# The limits of Antarctic conservation governance

**DOI:** 10.1038/s41467-026-77606-w

**Published:** 2026-10-06

**Authors:** Zachary T. Carter, Larissa Lubiana Botelho, Steven L. Chown, Parsa Yaryab, Nadiah P. Kristensen, Kerrie A. Wilson, Daniela Liggett, Michael Bode

**Affiliations:** 1 School of Mathematical Sciences, Queensland University of Technology, Gardens Point, Brisbane, QLD Australia; 2 Securing Antarctica’s Environmental Future, School of Mathematical Sciences, Queensland University of Technology, Gardens Point, Brisbane, QLD Australia; 3 Securing Antarctica’s Environmental Future, School of Biological Sciences, The University of Adelaide, North Terrace, Adelaide, SA Australia; 4 Securing Antarctica’s Environmental Future, School of Biological Sciences, Monash University, Melbourne, Victoria Australia; 5 Securing Antarctica’s Environmental Future, School of Biology and Environmental Science, Queensland University of Technology, Gardens Point, Brisbane, QLD Australia; 6 School of Earth and Environment, University of Canterbury, Christchurch, New Zealand

**Keywords:** Climate-change policy, Conservation biology, Social sciences

## Abstract

The Antarctic region exerts a profound influence on the Earth system, making its conservation fundamental to sustaining planetary health and human wellbeing. The Antarctic Treaty System (ATS) holds near-exclusive authority for environmental protection across the region, yet questions remain regarding its capacity to address a growing range of conservation threats. We investigate this by analysing over 90,000 scientific and policy documents (1961–2023) to examine how conservation threats are discussed across science and ATS policy deliberations. We find that threats originating more locally show clearer evidence of progression through the ATS’s decision-making system. Threats originating within the Antarctic Treaty Area show repeated progression towards governance action, whereas external threats, including climate change, remain under prolonged deliberation or receive limited engagement altogether. These patterns suggest that the ATS has demonstrated capacity to translate sustained engagement into governance action for threats within its remit, while comparable action remains less evident for threats originating beyond it. This distinction is increasingly consequential because some of Antarctica’s greatest threats originate outside the region. Strengthening coordination between the ATS and broader global governance frameworks may therefore be critical to addressing them.

## Abstract (web summary)

Many of Antarctica’s conservation threats originate beyond the region. This analysis of 90,000+ documents, finds that issues arising within Antarctica more clearly progress from discussion to governance action, while external threats often remain under deliberation.

## Introduction

The Antarctic region holds both intrinsic and global significance. It is one of Earth’s largest, most pristine biodiversity sanctuaries^1^ and plays a vital role in regulating the global climate and ocean systems^2,3^. The region’s conservation is therefore fundamental to achieving ambitious sustainability objectives that seek to maintain planetary health and human well-being, including the Kunming-Montreal Global Biodiversity Framework^4^ and the Paris Climate Agreement^5^. Failure to protect the Antarctic will unequivocally result in negative ecological and societal consequences that are likely to be large-scale and irreversible^6–8^.

At the heart of Antarctica’s protection lies a single integrated governance regime—the Antarctic Treaty System (ATS)—which exercises near-exclusive authority over how conservation is enacted across the region^9,10^. The ATS develops collective environmental responses through formal processes of deliberation and decision-making established under the Antarctic Treaty (via Article IX(1)). For environmental matters, these processes operate primarily through the comprehensive conservation framework established by the Protocol on Environmental Protection to the Antarctic Treaty (the ‘Protocol’; advised by the Committee for Environmental Protection [CEP]). Within this framework, the Antarctic Treaty Consultative Meeting (ATCM) serves as the ATS’s primary decision-making forum, where Treaty Parties come together to deliberate on and negotiate collective decisions. Scientific and technical advice is formally embedded in these negotiations, making the relationship between science and policy central to the regime’s design^11,12^.

Despite the ATS’s structured pathway for conservation policy, its capacity to achieve substantive outcomes has long been contested^13,14^. Across its more than 60-year history, the regime has been both lauded as an exemplar of environmental governance in a shared international space^15,16^ yet also criticised as a system in paralysis, no longer capable of delivering substantive outputs^17–20^. Resolving this debate has become increasingly important as conservation threats facing the Antarctic region continue to diversify and intensify^21,22^. These threats differ markedly in their origin, scale of potential impact, and underlying drivers, ultimately placing different demands on the ATS^1,23–25^. This complicates efforts to assess or improve the regime’s performance^26,27^: apparent underperformance may reflect system-wide institutional failure, weaknesses in specific contexts, or the limits of what the regime can reasonably be expected to achieve^16,28,29^. Clarifying this position is critical to the Antarctic region’s preservation^30^, as it defines whether future efforts to strengthen conservation should focus on internal reform, strengthening coordination with external governance frameworks, or maintaining existing approaches^20^.

To address this issue, we quantitatively examine how the ATS engages with conservation threats through its deliberative and decision-making processes. We focus specifically on the regime’s capacity to recognise, deliberate on, and develop responses to conservation threats. Accordingly, our analysis centres on deliberative dynamics, rather than treating the regime’s institutional architecture or policy outputs alone as evidence of capacity. Institutional architecture reveals the authorities, obligations, and procedures available to the ATS, but not how these mechanisms are deployed in practice. Likewise, policy outputs provide evidence of governance responses but reveal comparatively little about the deliberations through which those responses emerged. Each perspective illuminates a different dimension of the regime’s design and performance^20^, but neither captures the full process through which conservation threats are considered and addressed^31^. This distinction is critical because major policy interventions often result from prolonged periods of negotiation and deliberation that are not apparent from the outputs alone^32,33^. We therefore examine changes in deliberative processesengagement and interpret them alongside the ATS’s institutional architecture and policy outputs to assessm the regime’s capacity to address conservation threats.

The ATS provides a rare opportunity to investigate these deliberative processes because agenda-setting, policy deliberation, and decision-making are formally structured around documents submitted to the ATCM^34^. These submissions create a documentary record of how conservation threats enter and move through the regime’s deliberative processes. Because environmental decisions are meant to be guided by the best available scientific and technical advice^12,35^, understanding these processes also requires consideration of the scientific discourse from which that advice is drawn. We therefore analyse more than 90,000 English-language scientific publications and ATCM policy documents that have been published since the ATS came into force (1961–2023, inclusive; hereafter the ‘corpus’).

## Results

**Fig. 1:**
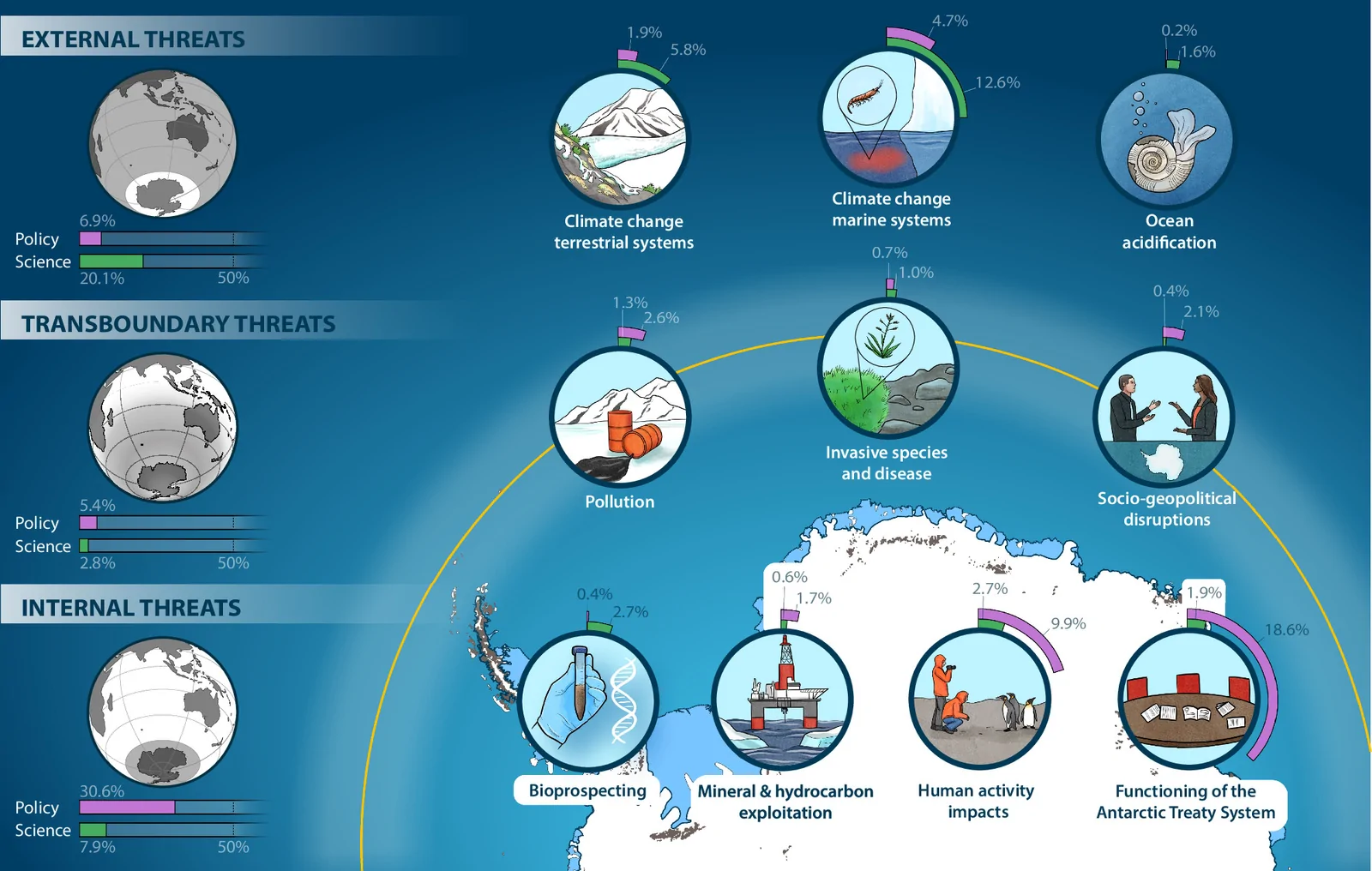
Overview of Antarctic conservation threats and their representation in science and policy. Illustrations depict the ten conservation threats analysed in this study and their representation across the scientific (*n* = 85,096) and ATCM policy (*n* = 5924) corpora (1961–2023). Threats are grouped according to where the processes generating them originate relative to the Antarctic Treaty Area (60° South latitude; yellow circular boundary), distinguishing between External, Transboundary, and Internal threats. Bar charts (left) show the aggregate prevalence of each threat category across the full corpus, while pie charts show the prevalence of individual conservation threats, expressed as percentages of all documents within the corresponding corpus (scientific or policy). Percentages do not sum to 100% because only topics assigned to conservation threats are shown; the remaining proportion corresponds to topics not assigned to conservation threats.

We examined ten conservation threats, grouped according to where the primary processes generating each threat originate relative to the Antarctic Treaty Area (60°S latitude): Internal, External, and Transboundary (Fig. 1). This distinction provides the analytical basis for comparing patterns of ATS engagement across conservation threats that place different demands on the regime. These threats were selected from a broader set identified through structured expert assessment as among the most pressing conservation challenges facing the Antarctic region^21,22,36^. Conceptually overlapping threats were consolidated (e.g., extreme precipitation within broader climate change impacts), while threats with limited representation in the scientific and policy literature (e.g., geoengineering and permanent human settlement) were excluded because they could not be meaningfully compared. Further details of threat identification, synthesis, and classification are provided in the Methods and Supplementary Note 1.

To examine how engagement with each threat changed over time, we analysed a corpus of 85,096 English-language scientific abstracts and 5924 policy documents submitted to the ATCM since the ATS entered into force in 1961. We used abstracts to enable large-scale, consistent comparison across the scientific literature, recognising that they capture the principal themes of each study but not its full nuance. For policy, we used whole documents and focused on Working Papers and Information Papers, which together provide the most comprehensive record of formal deliberations. Unlike policy outcomes (e.g., Decisions, Measures, or Resolutions), which reflect only a small subset of proposals able to achieve consensus, Working Papers and Information Papers capture the full spectrum of issues tabled for consideration, including proposals that did not result in a specific outcome. This offers a more comprehensive view of how conservation threats move through ATS deliberations than can be inferred from policy outcomes alone. The corpus does not include documents from the Convention for the Conservation of Antarctic Marine Living Resources (the CAMLR Convention). Further details of corpus construction, study scope, and the rationale for focusing on the ATCM are provided in the Methods.

We applied latent Dirichlet allocation (LDA) topic modelling to classify documents according to the conservation threats in Table 1^37^. We then fitted cubic splines to annual estimates of engagement with each conservation threat to track how engagement changed over time. We interpret statistically significant increases in a spline’s trajectory as indicating increasing engagement with a conservation threat, suggesting that the issue is becoming a more prominent subject of discourse. Conversely, declining engagement indicates that a threat is becoming less prominent and may reflect successful governance responses or constraints on the ATS’s capacity to act^34^. Importantly, low levels of engagement are inherently ambiguous and should not be interpreted as definitive evidence that the ATS lacks the capacity to address a particular threat. Comparing these temporal dynamics between science and policy provides insight into how conservation threats are reflected across the science-policy pathway that underpins ATS decision-making. Further methodological details are provided in the Methods and Supplementary Note 1, with validation analyses presented in Supplementary Note 4.

**Table 1:** Representative scientific and policy documents identified through topic modelling for each conservation threat

Category	Conservation Threat	Characteristic documents (classified by topic model)^a^
Internal threats	Functioning of the Antarctic Treaty System^b^	Scientific Committee on Antarctic Research (SCAR) meeting report^82^
		Review of Antarctic Treaty Consultative Meeting (ATCM) recommendations^83^
	Mineral and hydrocarbon exploitation	Criteria for Antarctic resource access^84^
		Mineral resources declaration of principles^85^
	Bioprospecting	Biotechnological applications of Antarctic microbes^86^
		Krill diet and atherosclerosis studies^87^
	Human activity impacts	Managing ship-based tourism impacts^88^
		Tourism and non-governmental activity guidelines^89^
External threats	Climate change impacts on terrestrial systems	Surface albedo feedback in climate models^90^
		Antarctic Climate Change and the Environment report update^91^
	Climate change impacts on marine systems	Ice sheet response to Southern Ocean warming^92^
		Climate change impacts on marine food webs^93^
	Ocean acidification	Ocean acidification in a high-CO₂ world^94^
		Southern Ocean acidification impacts^49^
Transboundary threats	Invasive alien species and disease	Invasive grass *Poa annua*^95^
		Aliens in Antarctica (International Polar Year outcomes)^96^
	Pollution	Microplastics in penguins^97^
		Removal of abandoned bases and waste^98^
	Socio-geopolitical disruptions	Australian Antarctic Territory and non-recognition^99^
		Jurisdictional issues in Antarctica^100^

^a^Documents are representative examples associated with topics assigned to each conservation threat. Topic assignments are probabilistic rather than exclusive.

^b^Refers to deliberations on the regime’s effectiveness and decision-making performance, where concerns raised by Treaty Parties are treated as an internal governance threat that can hinder conservation outcomes.

### Science and policy engage with different classes of conservation threats

The scientific and ATCM policy corpora differ in how they engage with conservation threats, with science engaging more strongly with external threats and policy more strongly with internal threats (Fig. 1). In the scientific corpus, external threats account for 20.1% of all discourse, compared to 7.9% for internal threats. Climate change impacts on marine systems was the largest contributor to the external category (12.6%), while bioprospecting and human activity impacts were the leading internal threats (2.7% each). The ATCM policy corpus shows the inverse pattern, with internal threats comprising 30.6% of all discourse and external threats only 6.9%. Discussion of the ATS’s functioning represents the largest share of policy content, accounting for 18.6% of all policy content. Transboundary threats were represented at comparable levels in both corpora, comprising 5.4% of policy discourse and 2.8% of scientific discourse. These patterns suggest that engagement within the ATCM has historically been concentrated on threats that can be addressed within the Antarctic region, whereas scientific engagement has been more strongly directed toward threats with global drivers.

**Fig. 2:**
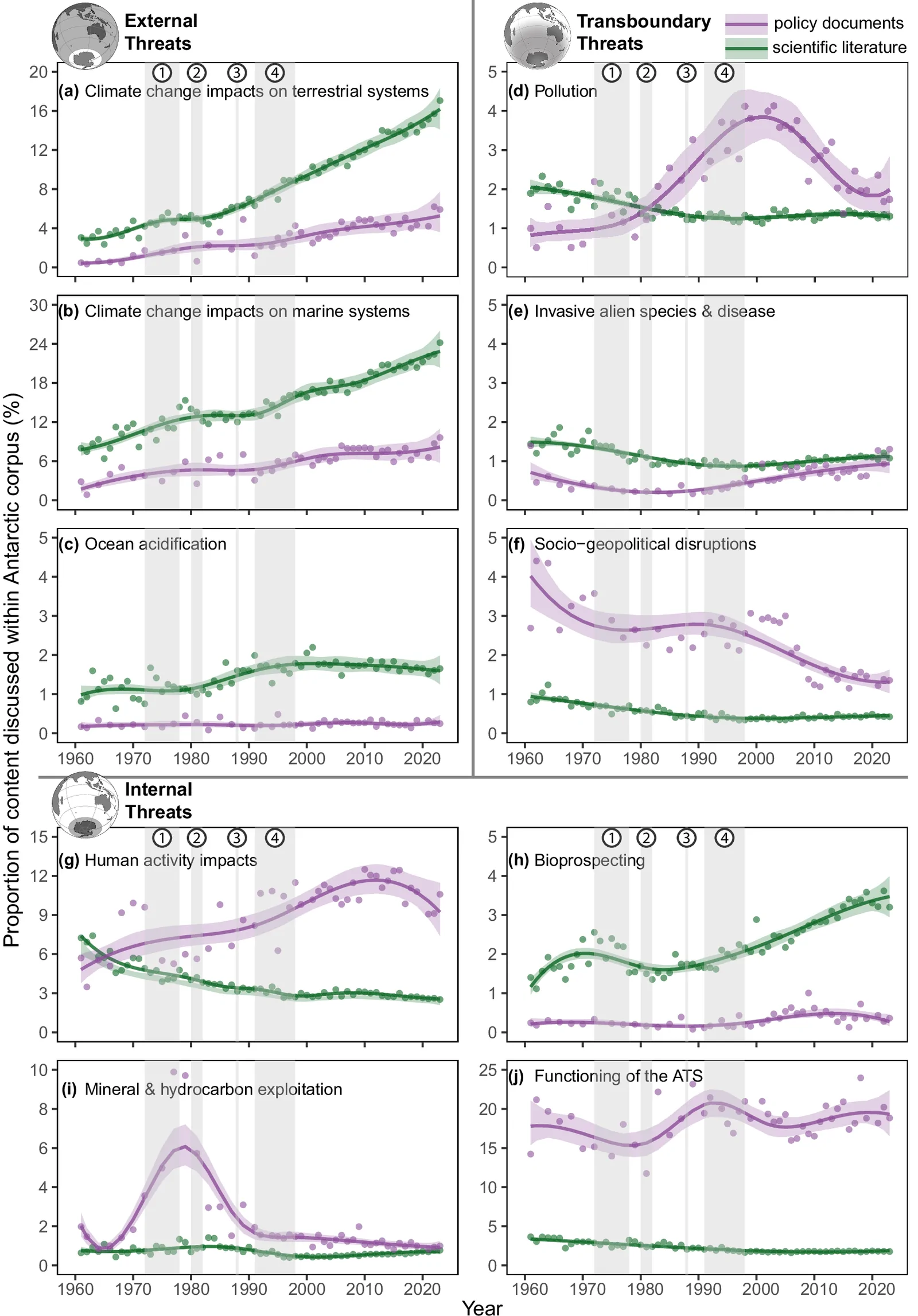
Changes through time in the proportional representation of scientific and policy engagement with major Antarctic conservation threats (1961–2023). Lines represent fitted cubic spline estimates, and shaded bands indicate pointwise 95% confidence intervals derived from 1000 parametric bootstrap replicates. Threats are grouped according to where the processes generating them originate, relative to the jurisdictional boundaries of the Antarctic Treaty Area: External Threats’ (panels **a**–**c**), 'Transboundary Threats’ (panels **d**–**f**), and ‘Internal threats’ (panels **g**–**j**). Major policy instruments are labelled: ① Convention on the Conservation of Antarctic Seals (CCAS; adopted 1972, entered into force 1978), ② Convention on the Conservation of Antarctic Marine Living Resources (CCAMLR; 1980, entered into force 1982), ③ Convention on the Regulation of Antarctic Mineral Resource Activities (CRAMRA; adopted 1988, never entered into force), ④ Protocol on Environmental Protection to the Antarctic Treaty (the ‘Protocol’, adopted 1991, entered into force 1998).

The spline fits indicate that this asymmetry has persisted through time (Fig. 2 and Supplementary Data 1). The only clear exception to this broader pattern is bioprospecting, the sole internal threat where scientific engagement has consistently exceeded policy engagement (Fig. 2g; proportional representation in the scientific corpus was 12 × higher than for policy in 2023). The ATCM’s permissive approach to bioprospecting, treated as a topic for ongoing deliberation rather than formal regulation (under Resolutions 7 [2005] and 9 [2009]), likely contributes to this disparity^38,39^. More than half of all national Antarctic programmes engage in bioprospecting^40^, investigating the use of bioactive compounds from psychrophilic (cold-adapted) organisms in pharmaceuticals, biotechnology, and industrial processes.

### Temporal dynamics differ systematically across threat categories

**Fig. 3:**
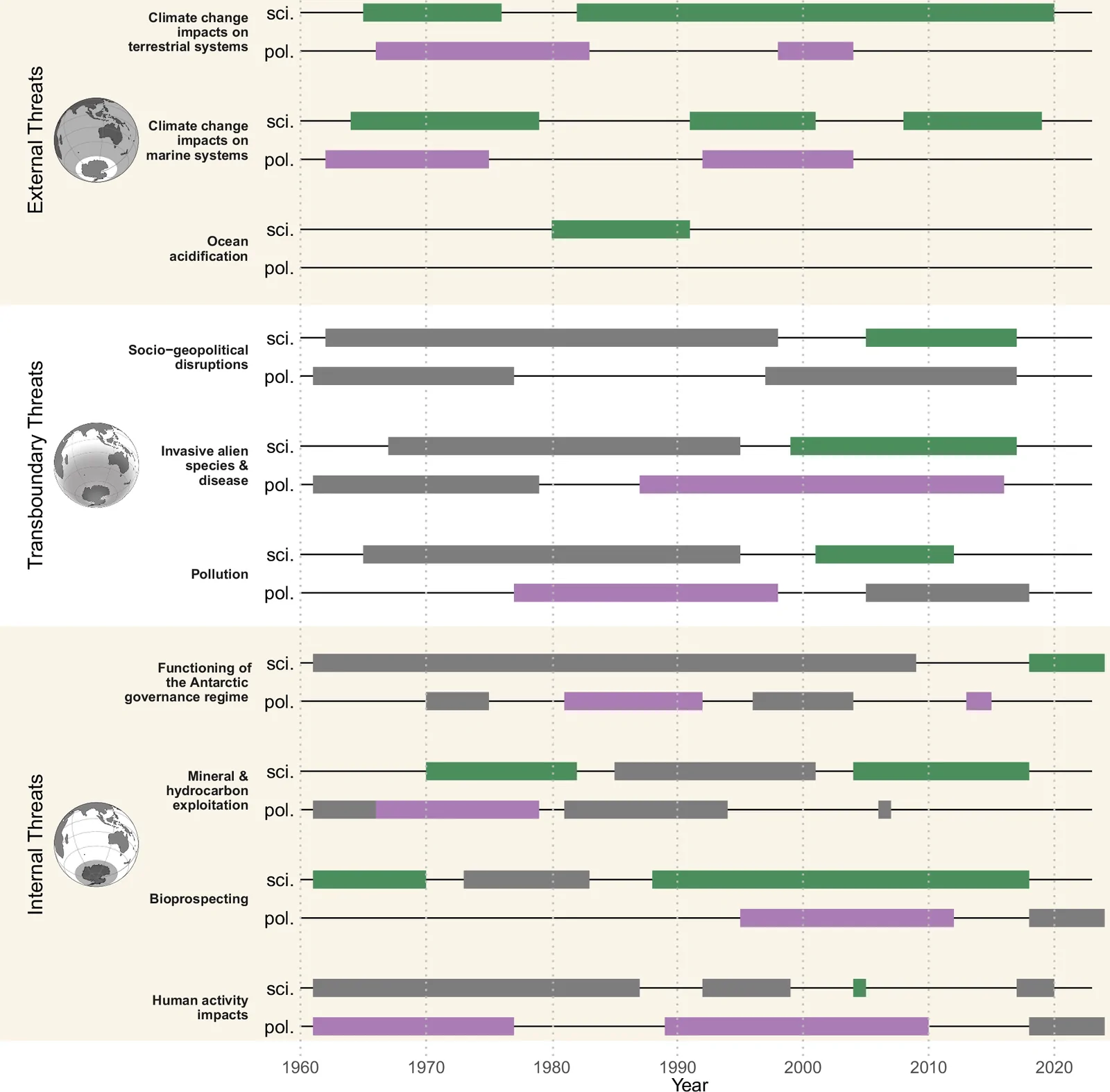
Periods of significant change in scientific and policy attention to major conservation threats, identified from the spline curves shown in Fig. 2 (1961–2023). Shaded intervals indicate periods during which engagement with a conservation threat increased or decreased significantly, providing a comparative overview of how engagement has changed through time across the scientific and policy corpora. Green () and purple () shading represent periods of significant growth within the scientific and ATCM policy corpora, respectively, while grey () shading indicates periods of significant decline. Threats are grouped according to where the processes generating them originate, relative to the jurisdictional boundaries of the Antarctic Treaty Area. ‘sci’ = science; ‘pol’ = policy.

While broad differences exist in how conservation threats are represented across science and policy, examining how those patterns change through time indicates systematic differences among threat categories (Fig. 3). Across the combined scientific and policy corpora, gradient analysis of the fitted splines indicated that the temporal dynamics of engagement differ systematically across threat categories (Kruskal–Wallis: H(2) = 14.90, *p* < 0.001; 95% CI = 0.0002–0.0012). Internal threats exhibited the greatest temporal variability, transboundary threats intermediate variability, and external threats the least (Jonckheere–Terpstra: *J* = 10.5, *p* < 0.001). In other words, engagement with internal threats was characterised by repeated episodes of increase and decline, whereas engagement with external threats followed more consistent trajectories. Importantly, this pattern was maintained when the scientific and policy corpora were analysed separately and was robust to changes in model parameterisation. Detailed statistical outputs, including pairwise comparisons for the combined and individual corpora, are provided in Supplementary Table 1. Sensitivity analyses are presented in Supplementary Note 4.

### Policy engagement reflects major developments in Antarctic governance

Changes in policy engagement often aligned with the negotiation and implementation of major ATS agreements for internal and transboundary threats (Figs. 2, 3). Across the ATS’s lifespan, engagement often increased during periods of active deliberation and subsequently declined once major governance outcomes had been achieved, although some issues later re-emerged as new concerns arose (see Supplementary Note 2 for complete details). For example, policy deliberations on mineral and hydrocarbon exploitation (Figs. 2f, 3) increased throughout the 1970s, peaking around 1981 as the ATCM established core principles for what would become the Convention on the Regulation of Antarctic Mineral Resource Activities (CRAMRA)^41,42^. At its peak, the issue accounted for 6.1% (95% confidence interval [CI] = 5.1–7.2%) of conservation-related discussions. Engagement subsequently declined during the protracted CRAMRA negotiations (1982–1988) and plateaued after the adoption of the 1991 Protocol, which prohibits commercial extraction of all non-living resources^43^.

By contrast, engagement with external threats associated with climate change impacts increased steadily through time (Figs. 2a, b 3). Since 1989, the ATCM has adopted 13 climate-related instruments (Recommendations, Decisions, and Resolutions), all of which are non-binding and stop short of imposing enforceable obligations or concrete mitigation targets^44–46^. Engagement with this threat continued to increase despite these governance responses, indicating that it remained a persistent subject of ATS deliberation, in contrast to the declining trajectories observed for many internal threats.

## Discussion

Whether the ATS possesses the capacity to address conservation threats remains unresolved. Because that capacity cannot be observed directly, we examine how issues emerge, persist, and recede within ATS deliberations. These patterns provide a measurable record of how threats move through the regime’s deliberative processes. We examined these dynamics across the ATS’s science-policy pathway to evaluate what they suggest about the regime’s capacity to recognise, deliberate on, and respond to different categories of conservation threat.

Our analysis suggests that the ATS’s jurisdictional boundaries shape how it engages with conservation threats. Internal threats were characterised by episodic engagement, with periods of increasing deliberation often followed by declining engagement after major governance responses. We interpret this rise-and-fall pattern as evidence of conservation issues progressing through the regime’s decision-making system: deliberative attention accumulates as a threat is recognised and debated, before declining following the development of governance responses. Under this interpretation, the observed decline in engagement is consistent with deliberative attention being translated into governance action, rather than simply indicating neglect or inaction. Such patterns provide evidence that sustained deliberation can culminate in governance action for threats that fall clearly within the ATS’s remit. This, in turn, suggests that the regime possesses the capacity to develop responses to regional conservation challenges through its formal decision-making processes.

By contrast, external threats exhibited markedly different trajectories, remaining either persistent subjects of deliberation throughout the ATS’s lifespan^6,47^ or receiving comparatively little engagement^48,49^. Unlike the rise-and-fall patterns observed for internal threats, neither trajectory provides a clear indication that issues are progressing through the regime’s decision-making system. Because the primary drivers of these threats originate beyond the Antarctic Treaty Area, their resolution often depends on actions taken outside the ATS itself. Sustained engagement may therefore reflect continued efforts to address unresolved challenges, while limited engagement may indicate either constraints on the regime’s ability to act or reliance on governance processes occurring elsewhere. The trajectories alone do not allow these possibilities to be distinguished, limiting what can be inferred about the ATS’s capacity to address threats whose primary drivers originate beyond its jurisdiction.

To better understand what these engagement dynamics suggest about the ATS's capacity to address conservation threats, we interpret them alongside the regime's institutional architecture and governance responses.

### Interpreting engagement dynamics

The ATCM’s science–policy pathway provides a comparative lens for examining how scientific priorities are reflected in policy deliberations. Science and policy emphasised different conservation threats, with science placing greater emphasis on global issues of broad significance, and ATCM deliberations concentrated on regionally confined issues where the regime holds clear authority to act. This concentration of policy attention on regional issues may partly reflect the historical origins of the ATS, which was initially established to manage issues arising within the Antarctic Treaty Area^50^. Yet despite these differences, both corpora exhibited similar temporal dynamics. Specifically, engagement with internal threats exhibited the greatest temporal variability, transboundary threats showed intermediate variability, and external threats exhibited the least variable trajectories in both science and policy. This correspondence suggests that scientific and policy discourse remain connected despite emphasising different conservation threats, consistent with the science-policy relationship envisaged under the Protocol^12^. However, the mechanisms through which this relationship operates appear more complex than simple alignment of priorities.

The episodic trajectories observed for many internal threats are consistent with conservation issues progressing through the ATCM’s decision-making system. Periods of intensifying deliberation were frequently associated with the development of governance responses. Declining engagement often followed the establishment of new regulatory mechanisms or policy frameworks, consistent with governance responses reducing the need for continued deliberation. This pattern is exemplified by the Protocol (signed 1991, entered into force 1998), which catalysed interest in some threats while contributing to the decline of others as regulatory mechanisms took effect. For example, discussions on human activity impacts intensified for roughly two decades following the signing of the Protocol (Figs. 2g, 3), reflecting the continued deliberations required under its Annexes I and V. Conversely, discussions of pollution, which rose steadily through the 1980s and early 1990s, declined after the Protocol entered into force (Figs. 2d, 3), as Annexes III and IV established regulatory mechanisms that reduced the need for continued debate.

The ATCM, however, has historically shown limited substantive engagement with bioprospecting (Fig. 2h), suggesting that the ATS has not translated its capacity into governance responses equally across all issues within its remit. This reluctance may partly reflect the commercial interests associated with Antarctic genetic resources, as well as unresolved questions regarding how intellectual property rights should be reconciled with the Treaty’s commitment to the free exchange of scientific information and research results (under Article III)^51^. Several Parties have themselves acknowledged this gap, calling for greater transparency and information exchange, and noting that without such measures the ATCM cannot adequately evaluate the implications of bioprospecting or determine appropriate governance responses^52^. The continued disparity between scientific and policy engagement suggests that bioprospecting remains an important area for future deliberation at the ATCM. (A full account of the dynamics across all internal and transboundary threats is provided in Supplementary Note 2).

Importantly, demonstrated capacity to address an issue should not be conflated with governance effectiveness. Our interpretation is based on the recurring pattern of deliberation, governance development, and subsequent decline in engagement observed across multiple internal and transboundary threats, rather than on the effectiveness of any individual governance outcome. This distinction is important because our analysis evaluates the ATCM’s capacity to develop responses to conservation threats rather than the effectiveness of the governance responses that emerge from those processes.

Recent accounts have criticised the ATCM for increasing reliance on soft-law instruments^20^ and for an adversarial atmosphere that undermines consensus even on seemingly minor policy decisions^53^. Our study is too coarse to evaluate such dynamics directly, underscoring the need for more nuanced work that links deliberations to specific outcomes. However, our results highlight that engagement with regional issues often unfolds over decades, with prolonged periods of negotiation and deliberation preceding major governance developments. These historical patterns suggest that apparent recent difficulties may reflect the long timescales of Antarctic decision-making rather than a permanent decline in the ATCM’s capacity to engage with regional conservation issues.

Unlike internal threats, the trajectories associated with external threats provide limited insight into whether these issues are progressing through the ATS’s decision-making system. Sustained or intensifying engagement indicates that Treaty Parties recognise their importance, but does not itself establish what these patterns imply about the ATS’s capacity to address them. Climate change, for example, has become an increasingly prominent subject of ATCM deliberations (Figs. 2, 3), reflecting widespread recognition of its significance to the Antarctic region. Yet legal scholarship indicates that Treaty Parties have approached the issue cautiously, prioritising institutional harmony and avoiding actions that could be perceived as extending beyond the ATS’s mandate^44,54,55^. Conversely, ocean acidification has received little attention at the ATCM despite being recognised globally as a major conservation threat^56,57^ (Fig. 2). Only seven dedicated Information Papers have been submitted on this threat, all by Observers rather than Consultative Parties, who are not directly responsible for decision-making^34^. While this may in part reflect a perception that ocean acidification falls under the remit of CAMLR to address^28^, the absence of any Party-led contributions nonetheless points to a reluctance to pursue the issue within the ATCM.

The observed trajectories alone do not allow us to determine whether the ATS lacks the capacity to address external threats or simply has not translated its available capacity into governance action. Interpreting these patterns, therefore, requires consideration of the regime’s institutional architecture. For external threats, this architecture is particularly important because their resolution requires coordination beyond the Antarctic Treaty Area^58^. The Antarctic Treaty provides for such engagement and offers a pathway to confront global challenges without exceeding its mandate^50,59^. In particular, Article III.2 encourages Parties to cooperate with United Nations (UN) Specialised Agencies and other international organisations with scientific or technical interests in Antarctica. This positions Antarctic governance within the wider international order. The Protocol reinforces this outward orientation by framing Antarctic protection as a global responsibility (preambular paragraph 8). Yet legal scholarship suggests that the ATS has often been reluctant to take this step, with Parties prioritising institutional autonomy over interaction with external institutions^55^.

The historical origins of the ATS may partly explain why these mechanisms for external engagement have not been fully utilised. The regime emerged at a time when Antarctica was comparatively isolated from wider geopolitical and economic processes and developed as a regionally oriented governance regime focused primarily on activities occurring within the Antarctic Treaty Area^13,60^. As conservation threats have increasingly been driven by processes operating beyond the Treaty Area^22^, this regional orientation may have fostered an institutional preference for regional solutions, leaving existing mechanisms for external engagement comparatively underutilised.

This legacy has important implications because external threats pose some of the most serious challenges facing Antarctic ecosystems^22^, yet they are also the issues for which the ATS provides the weakest evidence of progression through its deliberative processes. Several institutional reforms have been proposed to improve how the ATS addresses threats originating beyond the Antarctic Treaty Area, including restricting the decision-making influence of major greenhouse gas emitters, replacing consensus decision-making with majority voting, and strengthening coordination with other international institutions^61^. Many of these proposals would require substantive changes to the ATS’s institutional architecture. By contrast, fully exercising the Treaty’s existing mechanisms for external engagement may provide one of the most readily available opportunities to strengthen governance responses to threats originating beyond the Antarctic Treaty Area.

### Study limitations and considerations

These findings should, however, be interpreted in light of several methodological and scope limitations. Our modelling framework is most informative at the scale of broad conservation narratives and historically interpretable periods of change, rather than fine-grained temporal estimates. Sensitivity analyses indicated that changing model parameters affected the estimated magnitude of individual spline curves but had little influence on their overall shape or the broader temporal patterns they revealed. Accordingly, the framework is best suited to identifying persistent large-scale patterns of change and the relative prominence of conservation threats within scientific and policy discourse, rather than exact temporal estimates. Further details of the validation analyses are provided in the Methods and Supplementary Note 4.

While LDA enables holistic analysis of large corpora^37^, topics do not always correspond cleanly to individual conservation threats, particularly where threats share common terminology. The broad scientific search also retained a small proportion of false-positive records, potentially adding noise to the inferred topic structure. We addressed this through conservative topic assignment criteria, independent review, and validation analyses, which supported the overall robustness of the threat classifications. Nevertheless, some overlap between threat categories is unavoidable (see Supplementary Note 4 for complete details). In addition, LDA captures thematic prominence rather than the framing or sentiment of the discussion. Consequently, changes in engagement indicate how prominently a conservation threat is discussed, but not whether those discussions are supportive, critical, or otherwise framed. Distinguishing between descriptions of the ATS’s functioning versus dysfunction, for example, was not possible (see Supplementary Note 1 for details). Finally, our reliance on scientific abstracts and ATCM Working Papers and Information Papers limits access to the full nuance of debates and excludes informal or undocumented exchanges^9,62^. These sources nevertheless provide the most consistent and reproducible record of ATCM discourse.

Our analysis excludes CCAMLR documents, as CCAMLR constitutes a distinct decision-making body with different actors, institutional structures, and policy priorities^9^. This choice likely underestimates policy engagement with some marine-related conservation threats. Nevertheless, because the Protocol applies across the Antarctic Treaty Area, including marine environments south of 60°S, many Southern Ocean conservation threats are discussed across multiple ATS decision-making bodies rather than exclusively through CCAMLR^28^. Accordingly, our conclusions relate most directly to the Treaty and Protocol, while still providing insights into broader conservation dynamics across the ATS.

Despite its limitations, our application of LDA to Working Papers and Information Papers offers a systematic and reproducible way to analyse ATCM deliberations, providing insight into how conservation issues are negotiated within the ATS. While the Antarctic Treaty Secretariat maintains a comprehensive archive of official Meeting documents, it does not include transcripts, meaning previous assessments of negotiation dynamics have been largely limited to policy outputs rather than the deliberative record itself; e.g., see refs. ^63,64^. Here, our analyses support the use of Working Papers and Information Papers as a proxy for the substance of deliberations, with shifts in attention aligning closely with the negotiation and implementation of major environmental agreements. This approach, therefore, extends the study of Antarctic governance by revealing aspects of ATCM deliberations that would otherwise remain obscured.

### Pathways to strengthen the Antarctic Treaty System

Our analysis suggests that a central challenge facing the ATS in addressing conservation threats is not an absence of capacity, but how that capacity is translated into governance action when threats extend beyond the regime’s jurisdictional boundaries. For threats originating within the Antarctic Treaty Area, recurring cycles of deliberation, governance development, and declining engagement provide evidence that the regime can develop governance responses through sustained deliberation. External threats, however, present a different picture. Despite posing some of the greatest risks to Antarctic ecosystems^22^, the patterns of engagement provide comparatively little evidence that the ATS is translating available capacity into comparable governance action. Although the regime’s institutional architecture provides pathways for engaging with such threats, these mechanisms have not been fully leveraged^55^, with engagement instead characterised by either prolonged deliberation or limited attention. This finding is consistent with broader conclusions that many of Antarctica’s most significant conservation threats cannot be resolved in isolation^55,58^. For threats originating beyond the Antarctic Treaty Area, effective responses may necessarily depend on coordination across governance systems. Realising the full potential of the ATS may therefore require more deliberate links with global governance frameworks responsible for climate and ocean systems^44^. The regime already contains provisions for such coordination, and future progress may depend on how effectively these mechanisms are operationalised across governance systems.

Specific strategies for strengthening coordination across governance regimes have been explored elsewhere^65–67^. The most immediate opportunities lie in reinforcing engagement with the broader Antarctic regime complex^55,58^. This refers to the web of overlapping international institutions that influence the Antarctic region, including UN frameworks such as the UN Framework Convention on Climate Change (UNFCCC) and the UN Convention on the Law of the Sea (UNCLOS)^44,68^ (although see ref. ^54^ for a worthwhile critique). These linkages can be operationalised by positioning the ATS as a regional node within a wider governance network, where global rules are translated into Antarctic-specific measures and Antarctic policies inform global decision-making (see Supplementary Note 2 for additional considerations regarding the CCAMLR Convention). Operationalising these linkages requires acknowledging institutional overlap and deliberately managing it, ensuring that the ATS helps shape, rather than passively absorb, emerging global norms.

## Methods

**Fig. 4:**
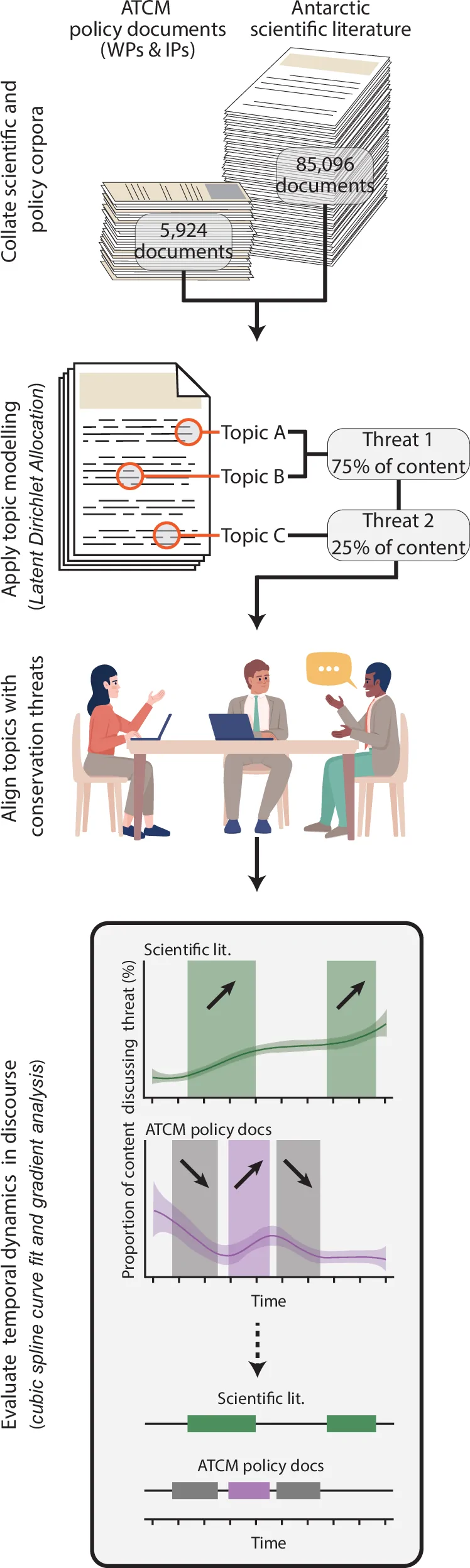
Illustrative summary of the methods used to identify trends in the Antarctic scientific literature and policy documents, 1961–2023. The workflow comprised four stages: collation of the scientific and policy corpora; application of latent Dirichlet allocation (LDA) topic modelling; alignment of LDA topics with conservation threats through structured qualitative review; and evaluation of temporal dynamics using cubic spline and gradient analyses. In the illustrative temporal plots, curves represent fitted cubic spline estimates and surrounding bands represent pointwise 95% confidence intervals; coloured intervals indicate periods of significant increase or decline identified from the spline gradients. ATCM = Antarctic Treaty Consultative Meeting; WPs = Working Papers; IPs = Information Papers.

Our approach to evaluating how the ATS engages with conservation threats comprised four main steps (Fig. 4). First, we compiled the corpus of English-language scientific abstracts and ATCM policy documents (Working Papers and Information Papers). Second, we applied LDA topic modelling to extract thematic structure from the corpus. Third, we used a structured qualitative review to align LDA topics with individual conservation threats. Finally, we fitted cubic spline curves to the annual proportion of documents discussing each threat, allowing us to track how engagement with threats changed over time. We analysed the gradients of these spline curves, using bootstrapped confidence intervals, to identify statistically significant periods of growth or decline in engagement. We begin by outlining the definitions and scope decisions that frame our analysis, after which we expand on each step in turn (see Supplementary Notes 1 and 3 for complete details).

### Conservation threat identification and categorisation

We compiled an initial list of conservation threats using refs. ^21^ and ^22^. We then refined this list in two steps: first, by consolidating conceptually similar phenomena where vocabularies and drivers overlapped (e.g., ‘heatwaves’, ‘surface melt’, and ‘glacier retreat’ were incorporated within the broader threat of ‘climate change impacts on terrestrial systems’); and second, by excluding threats identified in those studies as more distant concerns, whose limited presence in the corpus precluded robust analysis (e.g., ‘geoengineering’ or ‘agricultural expansion’). This process yielded ten conservation threats for inclusion in our study (see Supplementary Note 1 for full details).

To investigate how science and policy have engaged with these conservation threats, we grouped them according to the geographical location of the primary processes giving rise to them, relative to the Antarctic Treaty Area. This distinction reflects the expectation that threats may differ in the extent to which they can reasonably be addressed through ATS processes, and therefore provides a basis for comparing patterns of ATS engagement. We defined these categories relative to Article VI of the Antarctic Treaty, which applies south of 60°S latitude, including all ice shelves, without prejudice to the rights of any State under international law regarding the high seas within that area. We therefore define: (i) Internal threats as those whose threatening processes originate predominantly within the Antarctic Treaty Area; (ii) External threats as those whose threatening processes originate predominantly outside the Antarctic Treaty Area; and (iii) Transboundary threats as those whose threatening processes originate across both domains, such that neither internal nor external processes alone explain the threat. We emphasise that these categories are analytical rather than legal classifications and do not imply sovereign borders within Antarctica or take any position on territorial claims.

### Institutional background

The ATS is composed of four primary instruments: the Antarctic Treaty (signed 1959, entered into force 1961), the Convention for the Conservation of Antarctic Seals (CCAS; 1972/1978), the Convention on the Conservation of Antarctic Marine Living Resources (CAMLR Convention; 1980/1982), and the Protocol on Environmental Protection to the Antarctic Treaty (the ‘Protocol’; 1991/1998). The Treaty and the Protocol are further augmented by instruments adopted at the ATCMs, and the CAMLR convention is augmented by Conservation Measures adopted at the meetings of the Commission for the Conservation of Antarctic Marine Living Resources (CCAMLR). While both forums play central roles in Antarctic conservation, we focused our study on the ATCM because the Protocol constitutes the ATS’s comprehensive conservation agreement (Article II). By contrast, the CAMLR Convention defines conservation to include the rational use of marine living resources (Article II.2), giving it a distinct mandate, which differs from the focus of our study.

Our decision to analyse ATCM meeting documents, rather than those of CCAMLR, has two key implications. First, it situates our study within the Antarctic Treaty Area, rather than the broader CAMLR Convention Area, which extends to the Antarctic Convergence. Second, it narrows the scope of conservation-related policy discussions captured in our analysis, particularly those concerning the management of marine living resources. Although this decision limited our ability to capture every policy deliberation within the ATS, the Protocol addresses both terrestrial and marine environments, making ATCM discussions an adequate basis for assessing how the regime engages with conservation threats. Throughout this text, our references to the ATS should therefore be understood as focusing on the Treaty, the Protocol, and deliberations of the ATCM, rather than encompassing all instruments in equal measure.

At the ATCM, policy proposals and information exchange are conducted through meeting documents, which include Working Papers, Information Papers, Background Papers, and Secretariat Papers. Working Papers require discussion at the ATCM and, if adopted by consensus among Consultative Parties, can result in legally binding Measures or non-binding Decisions/Resolutions^9^. Information Papers provide background or supporting information to facilitate discussion, often in relation to issues raised in Working Papers, but do not in themselves require action or lead to binding outcomes^9^. Background Papers and Secretariat Papers are not presented orally at the Meeting and are subsidiary to policy deliberations^34^.

Observers and Experts play an important role in contributing scientific and technical advice to the ATCM^12^. Observers include intergovernmental and scientific organisations formally recognised under the Treaty framework (e.g., the Scientific Committee on Antarctic Research [SCAR]), which may submit both Working Papers and Information Papers. Experts comprise accredited non-governmental and industry bodies (e.g., the Antarctic and Southern Ocean Coalition [ASOC] and the International Association of Antarctica Tour Operators [IAATO]), which may submit Information Papers. While neither group has decision-making authority, their contributions broaden the knowledge base available to Parties and were captured in our analysis through the policy documents submitted to the ATCM.

### Measuring institutional engagement

Since Working Papers and Information Papers constitute the primary mechanism through which issues are introduced, discussed, and negotiated at the ATCM, they provide a systematic and observable record of deliberative activity within the ATS. We use the content of these documents as a proxy for institutional engagement with matters relevant to Antarctic conservation. Specifically, we quantify engagement as the proportion of policy text devoted to each conservation threat through time. We intentionally use the proportion of text devoted to a topic rather than the number of documents mentioning it for two reasons. First, considering the proportion of text devoted to a topic accounts for variation in the depth of discussion, which would be obscured if all documents contributed equally regardless of the amount of text on a particular issue. Second, this approach captures engagement with issues discussed within broader policy debates, even when they are not the primary focus of a document. Under this approach, greater textual attention is interpreted as indicating greater institutional engagement, while lower textual attention is interpreted as indicating lower engagement.

This approach assumes that changes in textual attention reflect changes in the degree to which the ATS engages with a particular issue. Under this assumption, temporal patterns in engagement may provide insight into the deliberative dynamics associated with a conservation threat. For example, periods of increasing attention followed by decline may be consistent with issue resolution, declining institutional interest, or recognition of constraints that limit further action. Conversely, sustained increases in attention without reprieve may indicate continued efforts to address an unresolved issue, the presence of institutional barriers, difficulties in reaching consensus, or the growing prominence of the threat itself. While these interpretations cannot be distinguished solely from the quantitative analysis we undertook, they provide a framework for understanding the patterns and dynamics of engagement observed in the data. While engagement should not be conflated with institutional capacity, patterns of engagement may provide insight into how that capacity is manifested through deliberative processes.

### Collation of scientific abstracts and ATCM policy documents

Our corpus comprised Antarctic-related scientific abstracts and ATCM policy documents that were published in the English-language since the ATS came into force (1961 to 2023, inclusive). We limited our investigation to English-language material for two reasons. First, English is one of the four official languages of the ATS (English, French, Spanish, and Russian), and all Working Papers are translated into each, ensuring that an English version is always available^34^. Information Papers may be submitted in any of the four languages, but most are written in English (see below). Second, the vast majority of published scientific literature, including the literature delimited to polar regions^69^, is communicated in the English language^70^. Although this limited our ability to explore perspectives embedded in other languages and cultures, we considered this subset of the primary literature and policy documents to be representative of the research taking place in the Antarctic region. Our final corpus comprised 85,096 scientific abstracts and 5924 policy documents (with 9 documents in total).

We collated Antarctic-related scientific abstracts following methodology described by Gray and Hughes^71^. Briefly, this involved a search of the Scopus scholarly database (www.scopus.com) with the following search query: (TITLE-ABS-KEY [antarct* OR “southern ocean” OR “ross sea” OR “Amundsen sea” OR “weddell sea”] AND NOT TITLE-ABS-KEY [candida OR “except Antarctica” OR “not antarctica”]) AND PUBYEAR > 1960 and PUBYEAR < 2024. The TITLE-ABS-KEY field code searched through articles based on their title, abstract and keywords. Qualifying terms were incorporated to reduce the number of false positive articles, such as papers on the yeast *Candida antarctica* or those explicitly excluding Antarctica (e.g., text with phrases similar to “every continent except Antarctica”). We extracted article metadata for analysis, including author(s), title, abstract, and publication year.

The Scopus scholarly database was selected over other platforms (e.g., Google Scholar) because it allows efficient web scraping to download large volumes of metadata. While Scopus may not capture all scientific literature, the ~ 85,000 abstracts retrieved are broadly representative of peer-reviewed research on Antarctic-related topics. We acknowledge, however, that abstracts provide only a condensed representation of individual articles and may omit methodological detail, contextual information, or secondary themes present within the full text. Nevertheless, abstracts are widely used in large-scale bibliometric and topic modelling analyses where full-text access is impractical^62^. Our analysis should therefore be interpreted as reflecting the dominant themes emphasised within the scientific literature rather than every aspect of the underlying article.

Approximately 92% of Working Papers and Information Papers produced since 1961 were available in English and suitable for analysis (5924 of 6422 documents), representing 96% of all Working Papers (2401 of 2494) and 90% of all Information Papers (3523 of 3928). Most were sourced from the Antarctic Treaty Database (www.ats.aq/devas/toolsandresources/antarctictreatydatabase), maintained by the Secretariat of the Antarctic Treaty, with additional material obtained from the University of Tasmania’s Antarctic Documents Database (www.utas.edu.au/library-resources/atadd) to fill gaps in early records. Documents were downloaded in their available formats (PDF, DOC, DOCX) and converted into machine-readable text for analysis. Earlier records were often low-quality scans of typewritten pages, for which we applied optical character recognition (OCR) using Meta’s Llama-4-Maverick-17B-128E-Instruct model^72^, a multimodal system capable of extracting text while preserving document layout and structure. OCR outputs were reviewed and corrected where necessary to remove artefacts and ensure consistency across the corpus. The remaining 8% (498 documents) could not be used because they were corrupted, missing content, available only in non-English versions, or inaccessible due to poor scan quality. Full details of the text-extraction and OCR workflow are provided in the associated repository (see the Data Availability statement for details).

### Topic modelling

We applied latent Dirichlet allocation (LDA) topic modelling to classify scientific abstracts and ATCM policy documents according to conservation threats^37^. We selected LDA because it represents each document as a mixture of topics, allowing conservation threats to be quantified as continuous topic proportions rather than mutually exclusive categories. Aggregating these proportions through time provides a quantitative basis for examining how engagement with different conservation threats changes across the corpus. Under the model, documents are represented as probabilistic mixtures of topics and topics as probabilistic distributions of words. The resulting document-topic distributions provide the basis for all subsequent analyses (see Supplementary Note 1 for full details)^73^.

All topic modelling was conducted in Python (version 3.10.12) using standard natural language processing libraries^74,75^. Texts were pre-processed by tokenising each document into individual words, lemmatising to reduce words to their base forms (e.g., “studies” to “study”), and removing stop words (e.g., “the”, “in”, “and”). To improve discriminatory power, we excluded the most frequent 15% and rarest 10% of terms, as very common words cannot differentiate between topics, and extremely rare words provide limited analytical value^76^. This filtering resulted in a vocabulary of 305,673 unique words.

After preprocessing, we applied LDA with *α* = 0.1 for the document–topic prior and *β* = 0.005 for the topic–word prior. These values encourage sparse distributions, concentrating documents on fewer topics and topics on fewer words, which improved the representation of under-represented policy documents^37^. We then trialled solutions for values of *n* = {10, 15, …, 95, 100} topics and measured the performance of each value using coherence and exclusivity metrics^76^.

The model with *n* = 90 achieved the highest overall coherence and exclusivity scores, indicating optimal topic separation and semantic consistency. We therefore used this 90-topic model for all subsequent analyses. The 90 topics chosen by the LDA analysis, and their 20 most-relevant terms, are provided in Supplementary Data 2, and the topic classification of all documents in Supplementary Data 3.

### Aligning LDA topics with conservation threats

We conducted a structured qualitative review to determine which LDA-derived topics aligned with specific conservation threats. This process involved a thematic analysis^77^ and was carried out in two stages: independent assessment of topic relevance followed by team-based consensus. First, each member of the research team independently examined the top 25 words for every topic to identify potential alignment with any threat. For topics deemed potentially relevant, reviewers then read the top 10 associated documents in full. A topic was retained only if all ten documents were judged to be substantively related to the threat in question. The team then convened to discuss individual selections and rejections. During group discussion, members could also review and reassess topics they had not initially selected but were brought forward by others. Deliberation continued until full agreement was reached on whether a topic clearly related to a particular threat. If consensus could not be reached, the topic was excluded. Only topics assigned to one or more conservation threats were retained for subsequent analyses of temporal dynamics. The final mapping of LDA topics to conservation threats is provided in Supplementary Note 3.

### Evaluating temporal dynamics in discourse

We analysed the annual topic proportions generated from our LDA model to examine how scientific and policy attention to conservation threats changed over time. For each document, the LDA model inferred a probabilistic distribution of topic proportions that summed to one across all topics. Annual topic percentages were then calculated by aggregating these document-level topic proportions within each year and expressing them as a proportion of the total number of documents published annually within each corpus. As such, each document contributed equally to the analysis irrespective of document length.

All model fitting and evaluation was conducted using the R statistical environment version 4.2.1^78^. We plotted the number of published Antarctic-related primary literature and policy documents on an annual basis to assess trends from 1961 to 2023, inclusive. We then characterised the behaviour of each annual plot using a cubic spline fit, which generates a smooth curve from piecewise polynomials joined end-to-end at connection points known as ‘knots’^79^. This approach provides a flexible representation of temporal patterns and is well suited to identifying periods of growth or decline through analysis of their first derivative (i.e., the ‘gradient’).

Our spline-fitting approach followed previously established methods^80^. Briefly, we selected the optimal number of equidistant knots for each spline curve using generalised cross-validation, which minimises prediction error in the fitted annual counts of scientific abstracts and policy documents^81^. Here, we assumed a lognormal scatter of the data about each curve. To quantify uncertainty, we generated pointwise 95% confidence intervals from 1000 parametric bootstrap replicates, each obtained by simulating a new dataset under the fitted error model and refitting the spline. Finally, we estimated the gradient of each spline over time to identify periods where attention to conservation threats intensified or diminished in the scientific and policy literature. Pointwise 95% confidence intervals for the gradient were obtained by taking the first derivative of each bootstrapped spline replicate. For each conservation threat, periods of significant growth or decline were identified throughout the ATS’s lifespan as years where the 95% confidence interval for the gradient did not include zero, with positive values indicating growth and negative values indicating decline.

We conducted statistical analyses on the identified periods of growth and decline to assess whether patterns in attention differed across the threat categories. Differences in the number of growth and decline periods were first assessed using the Kruskal-Wallis test, a nonparametric alternative to the one-way analysis of variance that does not assume normality. To ensure robust inference,* p*-values were estimated from 1000 parametric bootstrap replicates, each obtained by refitting the spline to resampled data. We then evaluated whether the number of significant periods followed an ordinal pattern across threat categories, using the Jonckheere-Terpstra test, a non-parametric method for detecting ordered differences among groups. In this case, the expectation was that the frequency of significant growth or decline periods would be highest for regionally confined (internal) threats, intermediate for transboundary threats, and lowest for global (external) threats.

### Technical validation

We evaluated the robustness of our analytical framework using a series of validation and diagnostic analyses spanning the full analytical workflow, from corpus construction to temporal modelling with cubic splines (Fig. 4). Specifically, we: (1) evaluated the precision of the scientific corpus retrieval strategy, noting that the policy corpus comprised all available ATCM Working Papers and Information Papers and therefore did not require equivalent retrieval validation; (2) tested whether imbalance between the scientific and policy corpora biased the inferred topic structure; (3) evaluated how alternative vocabulary filtering strategies influenced topic coherence and the retention of semantically meaningful terms; (4) quantified agreement between model-derived threat classifications and manually classified documents to assess the accuracy of our conservation threat assignment; and (5) tested the sensitivity of major temporal patterns identified in the analysis to five alternative model parameterisations.

Across these analyses, the validation results supported the reliability of the modelling framework while also identifying important analytical constraints. The scientific corpus retrieval strategy achieved high precision, corpus imbalance had little influence on the inferred topic structure, topic-to-threat classifications showed strong agreement with manually classified documents, and vocabulary sensitivity analyses supported the selected balance between semantic coherence and retention of domain-specific language. Moreover, the major temporal patterns identified in the analysis, including historically interpretable periods of institutional change, remained broadly robust to changes in model parameterisation. Spline magnitudes were, however, sensitive to parameter specification, and so exact spline estimates should be interpreted more cautiously than broader shifts in engagement through time.

Analytical limitations identified through these analyses are discussed further in the Discussion. Complete details are provided in Supplementary Note 4.

### Reporting summary

Further information on research design is available in the Nature Portfolio Reporting Summary linked to this article.

## Supplementary information

Supplementary Info

Description of Additional Supplementary File

Supplementary Data 1

Supplementary Data 2

Supplementary Data 3

Reporting Summary

Transparent Peer Review file

## Data Availability

The data supporting the findings of this study are available at https://doi.org/10.5281/zenodo.21972438. The OCR-processed ATCM policy papers, together with associated documentation and workflow details, are available at https://doi.org/10.5281/zenodo.17547450.

## Code availability

The code supporting the findings of this study is available at https://doi.org/10.5281/zenodo.21972438.
