# Supplementary material for: The limits of Antarctic conservation governance
Source: Nat Commun. 2026 Oct 6;17:11398. doi: 10.1038/s41467-026-77606-w (PMC13736897; doi:10.1038/s41467-026-77606-w)
Supplement: Supplementary file 1 [file 41467_2026_77606_MOESM2_ESM.pdf]

Description of Additional Supplementary Files

Supplementary dataset 1: Spline model outputs describing temporal patterns of scientific and policy engagement with each conservation threat from 1961–2023

Supplementary dataset 2: Latent Dirichlet allocation (LDA) topic model outputs, including the 90 inferred topics and their 20 most relevant terms.

Supplementary dataset 3: level topic classifications generated by the LDA model for the scientific and ATCM policy corpora.
