# Supplementary material for: The limits of Antarctic conservation governance
Source: Nat Commun. 2026 Oct 6;17:11398. doi: 10.1038/s41467-026-77606-w (PMC13736897; doi:10.1038/s41467-026-77606-w)
Supplement: Supplementary file 2 [file 41467_2026_77606_MOESM6_ESM.pdf]

nature portfolio

Corresponding author(s): Zachary T Carter

Last updated by author(s): 17/08/26

Reporting Summary

Nature Portfolio wishes to improve the reproducibility of the work that we publish. This form provides structure for consistency and transparency in reporting. For further information on Nature Portfolio policies, see our Editorial Policies and the Editorial Policy Checklist.

Statistics

For all statistical analyses, confirm that the following items are present in the figure legend, table legend, main text, or Methods section.

n/a	Confirmed	
☐	☒	The exact sample size (n) for each experimental group/condition, given as a discrete number and unit of measurement
☒	☐	A statement on whether measurements were taken from distinct samples or whether the same sample was measured repeatedly
☐	☒	The statistical test(s) used AND whether they are one- or two-sided<br>*Only common tests should be described solely by name; describe more complex techniques in the Methods section.*
☒	☐	A description of all covariates tested
☐	☒	A description of any assumptions or corrections, such as tests of normality and adjustment for multiple comparisons
☐	☒	A full description of the statistical parameters including central tendency (e.g. means) or other basic estimates (e.g. regression coefficient) AND variation (e.g. standard deviation) or associated estimates of uncertainty (e.g. confidence intervals)
☐	☒	For null hypothesis testing, the test statistic (e.g. F, t, r) with confidence intervals, effect sizes, degrees of freedom and P value noted<br>*Give P values as exact values whenever suitable.*
☒	☐	For Bayesian analysis, information on the choice of priors and Markov chain Monte Carlo settings
☒	☐	For hierarchical and complex designs, identification of the appropriate level for tests and full reporting of outcomes
☒	☐	Estimates of effect sizes (e.g. Cohen's d, Pearson's r), indicating how they were calculated

Our web collection on statistics for biologists contains articles on many of the points above.

Software and code

Policy information about availability of computer code

Data collection	Optical character recognition of historical ATCM policy documents was performed using Meta's Llama-4-Maverick-17B-128E-Instruct model. Custom Python scripts (Python v3.10.12) were used to retrieve, process, and compile scientific publication metadata and policy documents for analysis.
Data analysis	Text preprocessing and topic modelling were performed in Python v3.10.12 using standard natural language processing and machine learning libraries. Statistical analyses, spline modelling, bootstrap procedures, and data visualisation were conducted in the R statistical environment v4.2.1 using custom analysis scripts.

For manuscripts utilizing custom algorithms or software that are central to the research but not yet described in published literature, software must be made available to editors and reviewers. We strongly encourage code deposition in a community repository (e.g. GitHub). See the Nature Portfolio guidelines for submitting code & software for further information.

Data

Policy information about availability of data

All manuscripts must include a data availability statement. This statement should provide the following information, where applicable:

- Accession codes, unique identifiers, or web links for publicly available datasets
- A description of any restrictions on data availability
- For clinical datasets or third party data, please ensure that the statement adheres to our policy

Text preprocessing and topic modelling were performed in Python v3.10.12 using standard natural language processing and machine learning libraries. Statistical analyses, spline modelling, bootstrap procedures, and data visualisation were conducted in the R statistical environment v4.2.1 using custom analysis scripts.

Research involving human participants, their data, or biological material

Policy information about studies with human participants or human data. See also policy information about sex, gender (identity/presentation), and sexual orientation and race, ethnicity and racism.

Reporting on sex and gender	no human participants or human data were used in this study.
Reporting on race, ethnicity, or other socially relevant groupings	*Please specify the socially constructed or socially relevant categorization variable(s) used in your manuscript and explain why they were used. Please note that such variables should not be used as proxies for other socially constructed/relevant variables (for example, race or ethnicity should not be used as a proxy for socioeconomic status).*<br>*Provide clear definitions of the relevant terms used, how they were provided (by the participants/respondents, the researchers, or third parties), and the method(s) used to classify people into the different categories (e.g. self-report, census or administrative data, social media data, etc.)*<br>*Please provide details about how you controlled for confounding variables in your analyses.*
Population characteristics	*Describe the covariate-relevant population characteristics of the human research participants (e.g. age, genotypic information, past and current diagnosis and treatment categories). If you filled out the behavioural & social sciences study design questions and have nothing to add here, write "See above."*
Recruitment	*Describe how participants were recruited. Outline any potential self-selection bias or other biases that may be present and how these are likely to impact results.*
Ethics oversight	*Identify the organization(s) that approved the study protocol.*

Note that full information on the approval of the study protocol must also be provided in the manuscript.

Field-specific reporting

Please select the one below that is the best fit for your research. If you are not sure, read the appropriate sections before making your selection.

☐ Life sciences ☐ Behavioural & social sciences ☒ Ecological, evolutionary & environmental sciences

For a reference copy of the document with all sections, see nature.com/documents/nr-reporting-summary-flat.pdf

Ecological, evolutionary & environmental sciences study design

All studies must disclose on these points even when the disclosure is negative.

Study description	This study quantitatively examined how conservation threats are represented within scientific and policy discourse associated with the Antarctic Treaty System (ATS). Topic modelling was applied to two documentary corpora spanning 1961–2023 to quantify temporal patterns of engagement with conservation threats and compare these patterns between science and policy. This was an observational study of existing documentary records; no experimental treatments or interventions were applied.
Research sample	The scientific corpus comprised 85,096 Antarctic-related scientific abstracts retrieved from the Scopus database, while the policy corpus comprised all 5,924 English-language Working Papers and Information Papers submitted to Antarctic Treaty Consultative Meetings between 1961 and 2023. These corpora were selected because they provide a comprehensive record of scientific research and formal policy deliberations relevant to Antarctic environmental governance.
Sampling strategy	No statistical sample-size calculation was performed because this study analysed existing documentary corpora rather than experimental or observational samples. The policy corpus comprised all eligible English-language ATCM Working Papers and Information Papers available for the study period. The scientific corpus was retrieved using a predefined Scopus search strategy developed from established Antarctic bibliometric methods and subsequently evaluated through corpus retrieval validation. Sample sizes for validation analyses were determined to estimate retrieval precision within ±5% at the 95% confidence level.
Data collection	Scientific publication metadata were retrieved from the Scopus database using a predefined search strategy. ATCM Working Papers and Information Papers were obtained from the Antarctic Treaty Secretariat and the University of Tasmania Antarctic Documents Database. Historical policy documents requiring digitisation were converted to machine-readable text using optical character

	recognition before preprocessing and analysis. All data processing and compilation were performed by the authors using custom Python scripts.
Timing and spatial scale	Data were collected from documents published between 1961 and 2023 inclusive. Analyses were conducted at annual resolution. The study focused on documents relating to the Antarctic Treaty Area and the broader Antarctic region.
Data exclusions	The policy corpus comprised all available English-language Working Papers and Information Papers submitted to Antarctic Treaty Consultative Meetings between 1961 and 2023.
Reproducibility	Reproducibility was evaluated through multiple validation and sensitivity analyses spanning corpus retrieval, topic modelling, threat classification, and temporal modelling. These included corpus retrieval validation, vocabulary sensitivity analyses, topic classification validation, and sensitivity analyses examining alternative model parameterisations.
Randomization	Not applicable.
Blinding	Not applicable.

Did the study involve field work? ☐ Yes ☒ No

Reporting for specific materials, systems and methods

We require information from authors about some types of materials, experimental systems and methods used in many studies. Here, indicate whether each material, system or method listed is relevant to your study. If you are not sure if a list item applies to your research, read the appropriate section before selecting a response.

Materials & experimental systems

n/a	Involved in the study
☒	☐ Antibodies
☒	☐ Eukaryotic cell lines
☒	☐ Palaeontology and archaeology
☒	☐ Animals and other organisms
☒	☐ Clinical data
☒	☐ Dual use research of concern
☒	☐ Plants

Methods

n/a	Involved in the study
☒	☐ ChIP-seq
☒	☐ Flow cytometry
☒	☐ MRI-based neuroimaging

Plants

Seed stocks	*Report on the source of all seed stocks or other plant material used. If applicable, state the seed stock centre and catalogue number. If plant specimens were collected from the field, describe the collection location, date and sampling procedures.*
Novel plant genotypes	*Describe the methods by which all novel plant genotypes were produced. This includes those generated by transgenic approaches, gene editing, chemical/radiation-based mutagenesis and hybridization. For transgenic lines, describe the transformation method, the number of independent lines analyzed and the generation upon which experiments were performed. For gene-edited lines, describe the editor used, the endogenous sequence targeted for editing, the targeting guide RNA sequence (if applicable) and how the editor was applied.*
Authentication	*Describe any authentication procedures for each seed stock used or novel genotype generated. Describe any experiments used to assess the effect of a mutation and, where applicable, how potential secondary effects (e.g. second site T-DNA insertions, mosiacism, off-target gene editing) were examined.*
