# Supplementary material for: The limits of Antarctic conservation governance
Source: Nat Commun. 2026 Oct 6;17:11398. doi: 10.1038/s41467-026-77606-w (PMC13736897; doi:10.1038/s41467-026-77606-w)
Supplement: Supplementary file 3 [file 41467_2026_77606_MOESM7_ESM.pdf]

nature portfolio

Peer Review File

The limits of Antarctic conservation governance

Corresponding Author: Dr Zachary Carter

This file contains all reviewer reports in order by version, followed by all author rebuttals in order by version.

Version 0:

Reviewer comments:

Reviewer #1

(Remarks to the Author)
Overall comments:

Thank you for the opportunity to review this timely and important manuscript.

The authors of the manuscript present an analytically ambitious and methodologically innovative assessment of the institutional effectiveness of the Antarctic Treaty System (ATS) through examining how scientific research priorities align or diverge from policy deliberations at the Antarctic Treaty Consultative Meeting (ATCM). The use of more than 90,000 scientific and policy documents, coupled with large-scale computational/systematic analysis of textual data and their evolution over time, represents a substantial technical undertaking and addresses a long-standing gap in empirical analyses of ATS governance dynamics.

The manuscript is well written and addresses a topic of high salience to conservation governance, international law, political ecology, environmental law, and international relations. The large-scale systematic analysis is well established and the specific methodology appear appropriate and sound for the research (although I am not an expert in them).
Below you will find a few comments that the authors might consider for minor writing revisions of their manuscript, simply to capture nuance.

Specific comments:

1- Line 112: Use of the term ‘Transboundary’
Although the term has currency in other regional environmental regimes, applying ‘transboundary’ in the Antarctic context risks implying a boundary structure that does not exist under the Antarctic Treaty. It is also noteworthy that the Antarctic Treaty Area (ATA) is not defined by sovereign or jurisdictional boundaries in the sense ordinarily assumed by the concept of transboundary governance. Antarctica lacks internationally recognised sovereign borders, and the Treaty explicitly suspends territorial claims. Using ‘transboundary’ may inadvertently reinforce a territorial logic that cuts against the ATS’s legal architecture and may bias interpretation toward claimant-state narratives. A more appropriate framing would arguably be to describe these pressures as ‘broader-scope’ or ‘multi-scalar’ threats, which avoids unintended alignment with territorial assumptions, while better reflecting their functional drivers.

2- Line 115: Corpus imbalance and reliance on abstracts
According to the manuscript, more than 90 percent of the corpus consists of scientific abstracts, while policy documents account for only around 7 percent. This pronounced imbalance warrants closer scrutiny, as abstracts are necessarily concise, stylised, and lexically constrained, and differ fundamentally from ATCM Working Papers and Information Papers in both structure and purpose. Reliance on abstracts may therefore shape how themes are detected and prioritised, potentially amplifying the apparent prominence of globally framed scientific concerns relative to policy discourse within the ATS. The authors should more explicitly address how this imbalance affects the sensitivity and robustness of their text analysis, including whether topic identification is influenced by document length, how scientific and policy materials are comparatively weighted, and whether some observed divergences between science and policy may reflect differences in document structure rather than substantive disagreement and/or institutional misalignment.

3- Line 116: Exclusion of CCAMLR

The decision to exclude CCAMLR documents materially narrows the analytical scope of the study, while the manuscript nevertheless advances conclusions about the ‘effectiveness of the ATS’ as a whole. This is a consequential choice, as CCAMLR governs the largest spatial domain within the Antarctic regime complex and addresses many of the most acute conservation challenges, particularly those affecting marine ecosystems. The authors should therefore clarify whether their findings are intended to apply specifically to the ATCM and the Environmental Protocol, rather than to the ATS in its entirety, and discuss how the exclusion of CCAMLR may shape the assessment of threats whose primary impacts occur in marine systems. In addition, it would be helpful to consider whether excluding CCAMLR risks underestimating the extent to which the Antarctic governance system engages with global or multi-scalar pressures. Without such clarification, it may be difficult for readers to fully assess/understand how the manuscript’s conclusions about ATS institutional effectiveness relate to the empirical scope of the analysis.

4- Line 107: Basis for selecting the ‘ten conservation threats’
The manuscript states that the analysis ‘focused on ten conservation threats identified as among the most pressing for the Antarctic region’. While the supplementary material appears to provide additional context for this selection, the rationale remains underdeveloped in the main text. Given that the threat taxonomy anchors all subsequent analysis and interpretation, the authors should more clearly summarise in the manuscript itself the criteria used to identify these ten threats.
In particular, it would be helpful to clarify whether the designation of ‘most pressing’ reflects citation frequency, assessed ecological severity, governance relevance within the ATS, expert judgment, or a combination thereof, and to briefly indicate which categories of threats were considered but ultimately excluded.
Without a concise and explicit statement of these criteria in the main text, readers may find it difficult to assess the robustness and scope conditions of the analysis, even if further detail is available in the supplementary materials.

5- Line 138 (Table 1): Classification of SCAR documents under ‘Functioning of the ATS’
Table 1 classifies SCAR-related documents under the category “Functioning of the ATS,” which requires further clarification. SCAR is an independent scientific organisation and not an institutional component of the Antarctic Treaty System, and its activities do not constitute ATS functioning in a legal or governance sense. If SCAR documents are included within this category, the authors should clarify whether they are intended to serve as proxies for scientific advisory influence on ATS deliberations, or whether their inclusion reflects patterns of discursive interaction rather than institutional authority or decision-making. It seems that drawing a clearer conceptual boundary between scientific advisory functions/entities and treaty-based governance functions would strengthen the analytical coherence of the classification and avoid conflating distinct functions within the Antarctic governance landscape.

I hope the above comments are helpful to the authors.
With best regards,
Zia Madani

(Remarks on code availability)
I am not an expert in coding or related fields but I have reviewed the supplementary materials associated with the manuscript with respect to one of the issues that I have raised in my comments. The comment in question is the following:

Line 107: Basis for selecting the ‘ten conservation threats’
The manuscript states that the analysis ‘focused on ten conservation threats identified as among the most pressing for the Antarctic region’. While the supplementary material appears to provide additional context for this selection, the rationale remains underdeveloped in the main text. Given that the threat taxonomy anchors all subsequent analysis and interpretation, the authors should more clearly summarise in the manuscript itself the criteria used to identify these ten threats.
In particular, it would be helpful to clarify whether the designation of ‘most pressing’ reflects citation frequency, assessed ecological severity, governance relevance within the ATS, expert judgment, or a combination thereof, and to briefly indicate which categories of threats were considered but ultimately excluded.
Without a concise and explicit statement of these criteria in the main text, readers may find it difficult to assess the robustness and scope conditions of the analysis, even if further detail is available in the supplementary materials.

Reviewer #2

(Remarks to the Author)
General assessment

This manuscript addresses an important question: how conservation threats in Antarctica are reflected in scientific and policy discourses. The topic is relevant, and the authors should be commended for the clarity of their writing, the paper's overall structure, and the quality of the figures, which make the work accessible to a broad audience. The illustrations are well designed and visually compelling.

Antarctic conservation itself is not my primary area of expertise. My core expertise lies in systematic literature reviews, topic modelling, and natural language processing (NLP). My comments should therefore be interpreted primarily through a methodological lens. Also, I have an injury in both of my arms and can only voice type. Therefore, AI was used to polish the comments that I dictated to the app “Voicy”. However, the entire content is my own.

From this perspective, while the paper is promising, the current methodological framework does not yet support the strength of the conclusions drawn. In particular, there is a mismatch between what the methods used can reliably measure (patterns of attention or salience in text corpora) and what the paper claims to identify (prioritisation of conservation threats). Therefore, I recommend rejecting the paper.

1. Conceptual framing: prioritisation vs. attention

• The goal of the paper is to investigate how conservation threats have been prioritised in scientific and policy corpora (line 103). However, the applied methods primarily capture attention, salience, or frequency of discussion, not prioritisation in a normative or decision-making sense. This distinction is critical and should be reflected consistently throughout the paper.
• Line 114: the wording refers to “mining attention,” which is methodologically accurate and, in my view, better reflects what the analysis does.
• I recommend reformulating the research questions, framing, and conclusions to focus on attention, prominence, or thematic salience, unless additional evidence is introduced that directly supports claims about prioritisation.

2. Corpus construction and inclusion/exclusion criteria
• A major limitation of the study is the absence of clear inclusion and exclusion criteria for the documents included in both the scientific and policy corpora.
• The search query is very broad and is not systematically evaluated, making it difficult to assess what is actually contained in the corpus.
• Without knowing what proportion of documents are truly relevant (false positivies/negatives), it is hard to trust downstream topic modelling results.
• A sensitivity analysis of the search terms is essential. Using the same query, it is possible to retrieve documents that are clearly outside the intended scope. Examples of documents captured by the current query include:
o “7β-22 Dihydroxyhopane, Isolated from the Sub-Antarctic Lichen, Inhibits the Viability and Stemness in Glioma Stem Like Cells”
o “A Really Dark Landscape”: Cormac McCarthy's Blood Meridian and Modest Mouse's The Moon & Antarctica

These examples highlight the risk of false positives.
• I strongly suggest:
o Defining explicit inclusion/exclusion criteria,
o Reporting the expected rate of irrelevant documents (recall, precision),
o Potentially introducing a simple ML classification step (e.g. scientific BERT) after the keyword search to filter for genuinely relevant documents before topic modelling.

3. Topic modelling
• The use of LDA is a reasonable starting point, but it is an non-state-of the art model by current NLP standards and is known to be sensitive to preprocessing and hyperparameter choices. You will always obtain a topic solution with LDA; therefore, the key question is why this particular solution should be trusted.
• Line 479: LDA does not deterministically assign a topic to each word; it infers probabilistic topic–word and document–topic associations. The wording should be corrected accordingly.
• The choice to remove the most frequent 15% and rarest 10% of terms is non-trivial and appears arbitrary. Were alternative thresholds tested?
• Hyperparameter sensitivity for the LDA model is not explored.
• Without such testing, it is unclear whether the identified topics are robust or artefacts of specific modelling choices.
• A sensitivity analysis (e.g. varying vocabulary thresholds, α and β values, or number of topics) would substantially increase confidence in the results.
• The manuscript mentions qualitative validation, but this does not appear to have been conducted in a systematic manner. (e.g. what is the F1 score compared to manual validation)?
• Topic coherence and exclusivity are useful internal metrics, but they are insufficient on their own.
• I would strongly encourage:
o Labelling a subset of documents according to conservation threat categories,
o Comparing model outputs against these labels,
o Reporting standard evaluation metrics such as precision, recall, or F1 scores.

4. Interpretation of percentages and topic prevalence
• It is unclear how topic percentages are computed:
o Is each document weighted equally?
o Are percentages based on topic proportions per document, or on token-level aggregation across the corpus?
• This should be clarified explicitly, as it affects interpretation.
• Figure 1: the percentages shown are confusing. External, transboundary, and internal threats do not sum to 100. What is the denominator? How many documents are included in the scientific vs. policy corpora? Add to the legend For subclasses (e.g. transboundary threats), percentages should be shown relative to their parent category. The figure legend should clearly explain what the percentages refer to.

5. Other comments
• Line 41: the term “regime” is used without definition and should be clarified.
• The manuscript uses many acronyms (e.g. WPs, IPs), some of which have multiple meanings in other contexts. These should be written out in full where possible.

• It would be helpful to report the average length of WPs and IPs compared to abstracts, to assess whether topic distributions may be driven by document type rather than conservation content.
• Semantic variants (e.g. "outside Antarctica" vs. "beyond Antarctica") and intruder terms (e.g. bacteria, microbes) remain in the corpus and should be more systematically addressed.
• Line 120: the manuscript assumes that abstracts are broadly representative of full texts. Previous work (including my own) has shown that abstracts often omit nuance, methodological detail, and secondary themes that are present in full texts. While the use of abstracts is understandable for scalability, this limitation should be acknowledged more explicitly, and its implications for topic interpretation should be discussed.
• Figure 3, while visually appealing, is difficult to interpret. Clear patterns are not immediately apparent. I recommend reconsidering how this information is visualised, potentially through more aggregated or comparative representations that align more closely with the research questions.

(Remarks on code availability)

Reviewer #3

(Remarks to the Author)
This paper is an interesting read, and I salute the team for the underpinning work - which is substantial. But let me offer you a provocative reading of the paper rather than an endorsement.

Given the location of the team itself is there any intrinsic surprise that Australian and New Zealand-based academics would conclude ultimately that the ATS is doing a good job - "institutionally effective". There is in both countries a well-established academic-policy-media complex that would largely reproduce that over time in part because both countries are claimant states and original signatories - the treaty has been widely regarded as integral to national security as well as offer broader merit in terms of peace and cooperation. For both countries, the Southern Ocean/Antarctica is part of their backyards.

The paper's design and sources/methods cannot get at something rather important about the ATS and that is it foundational working culture - the treaty was designed at the onset to be geographically focussed, with a hope in 1959 that membership would remain limited. No one could imagine or wanted to imagine China being a consultative party. Recall the reaction to India back in the 1950s. The US and its special allies including both Australia and New Zealand assumed the ATS would be largely directed by their collective knowledge and commitment. The shift towards conservation in the 1960s was an experiment to see whether the ATS could expand its remit through new conventions on conservation-related matters, while retaining the all important consensus principle. The 1980s were pivotal in terms of really stressing and straining what John Heap described as the "Antarctic club". And it is no surprise that policy would focus on "internal threats" as that was hard-wired into the system right at the start. The ambivalence about the UN came out clearly in the 1980s.

Effectiveness travels together with legitimacy and I do wonder whether a great deal of what you describe is a system seeking to cope with a legitimacy crisis - the regional framing coming under pressure both politically and ecologically. Your focus on conservation threats could be seen as two types - the conservation of governance itself and (ecological) conservation governance. I am not sure I really understand your internal, external and transboundary classification - surely that depends on your judgement as you note but actually I would be more interested in how stakeholders made sense of it. Most of the issues blur internal/external and transboundary might also refer to connections with other places thought as global commons such as oceans and space.

In the discussion area, I would be tempted to temper this positive assessment of the ATS - since the negotiation of the Protocol there has been a marked reluctance to tackle bioprospecting, tourism and call out bad behaviour. There has been a loss of confidence and in part this has been brought on by both wider geopolitics but also diversity of membership and external scrutiny. If this capacity has not been realised then why would you conclude it is still fit for purpose. Truthfully, the Antarctic Treaty is an essential accomplice for claimant states and we need to prepare for a world that appears to be drifting towards Neo-imperialism where great powers might simply walk away from treaties. The Trump administration surely needs to be cited in that regard.

While you might think that there are options to strengthen the ATS, the prevailing context might prevail simply because attention is focused more on the Arctic. The failed attempts to expand the MPA network provide a salutary reminder about how difficult it will be to secure consensus - there are competing understandings of threats to Antarctica. We might not have sufficiently shared global norms to draw upon in the coming future and I do think the paper needs to be alert to this. In the end the most effective achievement of the Antarctic Treaty is ensuring that the Antarctic remains free of direct conflict and that there has been a track record of scientific collaboration but the record on the knotty issues such as resource management and environmental protection is patchy.

(Remarks on code availability)

Version 1:

Reviewer comments:

Reviewer #1

(Remarks to the Author)
The author/s have appropriately addressed/responded to all the issues/questions I raised in my previous review.
Thank you.

(Remarks on code availability)

Reviewer #2

(Remarks to the Author)
I would like to thank the authors for their comprehensive response to my review. I particularly appreciate that they grouped my comments into thematic areas, which made the response letter easier to follow. It is evident that the authors took the comments seriously and made a substantial effort to address them.

I also appreciate the revision of the terminology throughout the manuscript. Reframing the study from identifying "priorities" or "threats" to examining **patterns of engagement** much more accurately reflects what can be inferred from the analyses.

However, in the abstract, I would encourage rephrasing the statement that the study examines "how conservation threats progress through the ATS deliberative and decision-making process." I do not think that "progress" is the most appropriate term here, as the analyses do not directly track if progress in decision-making processes. Instead, I suggest wording such as "examines how conservation threats are discussed." I understand these are semantics, but I believe this additional precision would better align the wording with what is actually analysed.

I also appreciate the extensive validation analysis that has been added for corpus construction. The manual validation of the retrieved documents is a valuable addition, and I agree that, in this context, prioritizing precision over recall is the appropriate choice, as the main concern is the inclusion of irrelevant documents that could influence the topic modelling results. The transparent reporting of retrieval accuracy substantially increases my confidence in the results.

A remaining concern relates to the treatment of the false-positive documents. The validation shows that approximately 8% of the false positives are artefacts of the search strategy. While I understand the authors' decision to retain these documents, I still believe it would be preferable to remove these clearly noisy records prior to conducting the LDA topic modelling, as this would further improve the quality of the corpus and reduce the possibility that unrelated documents influence the inferred topics. I understand this would be additional work and that you prefer keeping the results as it is... However, maybe for the next time :)!

Overall, I believe the methodological aspects of the manuscript have improved since the previous version. I think the authors now provide sufficient guidance for many potential users to understand its strengths and limitations.

(Remarks on code availability)

Open Access This Peer Review File is licensed under a Creative Commons Attribution 4.0 International License, which permits use, sharing, adaptation, distribution and reproduction in any medium or format, as long as you give appropriate credit to the original author(s) and the source, provide a link to the Creative Commons license, and indicate if changes were made.
In cases where reviewers are anonymous, credit should be given to 'Anonymous Referee' and the source.
The images or other third party material in this Peer Review File are included in the article's Creative Commons license, unless indicated otherwise in a credit line to the material. If material is not included in the article's Creative Commons license and your intended use is not permitted by statutory regulation or exceeds the permitted use, you will need to obtain permission directly from the copyright holder.
To view a copy of this license, visit https://creativecommons.org/licenses/by/4.0/

Response to reviewers

The limits of Antarctic conservation governance

Zachary T. Carter, Larissa Lubiana Botelho, Steven L. Chown, Parsa Yaryab, Nadiah P. Kristensen, Kerrie A. Wilson, Daniela Liggett, Michael Bode

We thank the reviewers for their thoughtful and constructive feedback, and the editor for the opportunity to revise our manuscript. We were encouraged that the reviewers recognised the potential value of the study and the importance of quantitatively evaluating Antarctic governance dynamics. However, several substantive issues were identified requiring clarification and additional work. These included the need for stronger justification and validation of the modelling framework, clearer articulation of the assumptions underpinning the analysis, and more restrained interpretation of the results.

In response, we have extensively revised both the main text and Supplementary Information. The manuscript now includes an expanded validation framework and a revised conceptual framing of the study. We have also strengthened discussion of methodological limitations and revised the conclusions of our study accordingly. While these revisions resulted in extensive textual changes throughout the manuscript, the underlying analyses, results, and overall study design remain unchanged. We believe these revisions have improved the clarity, rigour, and transparency of the study.

To facilitate review, we first provide a point-by-point response to each reviewer comment. Following these responses, we provide a consolidated summary of the major revisions made to the manuscript. Hyperlinks have been included throughout the response letter to enable rapid cross-referencing between reviewer comments and the corresponding changes in the manuscript.

Reviewer Comments

Reviewer #1 3

Reviewer #2 8

Reviewer #3 18

Major Updates

Update 1 - Supplementary Information 4: Validation of the topic modelling pipeline 24

SI 4.1 Relevance validation 25

SI 4.2 Topic composition analysis 27

SI 4.3 Accuracy of topic-to-threat classification 30

SI 4.4 Sensitivity analysis 40

Update 2 – Revised conceptual framing 49

Revised Introduction excerpt (aims and objectives) 49

Revised Discussion excerpt (opening and interpretation of findings) 50

Update 3 – Expanded discussion of study limitations 51

Update 4 – Revised conclusions and recommendations 52

Reviewer #1

1. The authors of the manuscript present an analytically ambitious and methodologically innovative assessment of the institutional effectiveness of the Antarctic Treaty System (ATS) through examining how scientific research priorities align or diverge from policy deliberations at the Antarctic Treaty Consultative Meeting (ATCM). The use of more than 90,000 scientific and policy documents, coupled with large-scale computational/systematic analysis of textual data and their evolution over time, represents a substantial technical undertaking and addresses a long-standing gap in empirical analyses of ATS governance dynamics.
   The manuscript is well written and addresses a topic of high salience to conservation governance, international law, political ecology, environmental law, and international relations. The large-scale systematic analysis is well established and the specific methodology appear appropriate and sound for the research (although I am not an expert in them). Below you will find a few comments that the authors might consider for minor writing revisions of their manuscript, simply to capture nuance.

   Thank you for taking the time to review our manuscript and for your thoughtful and constructive feedback. We appreciate your recognition of the study's potential significance to conservation governance and related fields. Your comments were particularly helpful in refining the articulation of several assumptions and conceptual framings underpinning the analysis. We have carefully considered each point raised and made substantive revisions throughout the manuscript in response.

2. Line 112: Use of the term 'Transboundary' – Although the term has currency in other regional environmental regimes, applying 'transboundary' in the Antarctic context risks implying a boundary structure that does not exist under the Antarctic Treaty. It is also noteworthy that the Antarctic Treaty Area (ATA) is not defined by sovereign or jurisdictional boundaries in the sense ordinarily assumed by the concept of transboundary governance. Antarctica lacks internationally recognised sovereign borders, and the Treaty explicitly suspends territorial claims. Using 'transboundary' may inadvertently reinforce a territorial logic that cuts against the ATS's legal architecture and may bias interpretation toward claimant-state narratives. A more appropriate framing would arguably be to describe these pressures as 'broader-scope' or 'multi-scalar' threats, which avoids unintended alignment with territorial assumptions, while better reflecting their functional drivers.

   This is an important point, and we agree that the term "transboundary" may carry unintended legal or territorial connotations in the Antarctic context if left insufficiently defined. We have therefore clarified the rationale and definitions of these categories in the Methods, where the analytical framework is formally introduced. Specifically, we now state that they are analytical classifications based on the geographic origin of the primary processes generating each threat relative to the Antarctic Treaty Area, rather than legal categories implying sovereign borders or jurisdictional claims within Antarctica..

   The revised Methods now explicitly state (beginning on line 498 of the revised text):

We emphasise that these categories are analytical rather than legal classifications and do not imply sovereign borders within Antarctica or take any position on territorial claims.

3. Line 115: Corpus imbalance and reliance on abstracts – According to the manuscript, more than 90 percent of the corpus consists of scientific abstracts, while policy documents account for only around 7 percent. This pronounced imbalance warrants closer scrutiny, as abstracts are necessarily concise, stylised, and lexically constrained, and differ fundamentally from ATCM Working Papers and Information Papers in both structure and purpose. Reliance on abstracts may therefore shape how themes are detected and prioritised, potentially amplifying the apparent prominence of globally framed scientific concerns relative to policy discourse within the ATS. The authors should more explicitly address how this imbalance affects the sensitivity and robustness of their text analysis, including whether topic identification is influenced by document length, how scientific and policy materials are comparatively weighted, and whether some observed divergences between science and policy may reflect differences in document structure rather than substantive disagreement and/or institutional misalignment.

    We agree that corpus imbalance warrants careful consideration because the scientific and policy corpora differ substantially in both size and document structure. We deliberately fitted a single topic model across both corpora to enable direct comparison between scientific and policy discourse. Had the corpora been modelled separately, each would have generated its own topic structure, making direct comparison between science and policy considerably more difficult. This choice, however, raises the possibility that differences in corpus size or document structure could influence the inferred topic structure, and so we conducted additional analyses to assess whether such effects were evident.

    To evaluate this possibility, we compared the average representation of each topic across the scientific and policy corpora. If corpus imbalance were influencing the model, topics would be expected to exhibit consistently higher representation in the larger scientific corpus. However, we found no evidence of such a pattern across either the full topic set (paired Wilcoxon signed-rank test, $p = 0.99$) or the subset of conservation-threat topics used in the main analysis ($p = 0.29$). These results suggest that corpus imbalance did not systematically bias the inferred topic structure or the conclusions drawn from the model.

    The complete analysis can be found in Supplementary Information 4.2. We additionally summarise its principal findings in the main text through a new subsection of the Methods entitled "Technical validation" (beginning on line 705 of the revised text).

4. Line 116: Exclusion of CCAMLR—The decision to exclude CCAMLR documents materially narrows the analytical scope of the study, while the manuscript nevertheless advances conclusions about the 'effectiveness of the ATS' as a whole. This is a consequential choice, as CCAMLR governs the largest spatial domain within the Antarctic regime complex and addresses many of the most acute conservation challenges, particularly those affecting marine ecosystems. The authors should therefore clarify whether their findings are intended to apply specifically to the ATCM and

the Environmental Protocol, rather than to the ATS in its entirety, and discuss how the exclusion of CCAMLR may shape the assessment of threats whose primary impacts occur in marine systems. In addition, it would be helpful to consider whether excluding CCAMLR risks underestimating the extent to which the Antarctic governance system engages with global or multi-scalar pressures. Without such clarification, it may be difficult for readers to fully assess/understand how the manuscript's conclusions about ATS institutional effectiveness relate to the empirical scope of the analysis.

We agree that this is an important and highly salient consideration when interpreting the scope of our study and its conclusions. We therefore expanded the Discussion to clarify how excluding CCAMLR shapes the scope of the analysis and interpretation of the results.

We now state (beginning on line 410 of the revised text):

> Our analysis excludes CCAMLR documents, as CCAMLR constitutes a distinct decision-making body with different actors, institutional structures, and policy priorities[9]. This choice likely underestimates policy engagement with some marine-related conservation threats. Nevertheless, because the Protocol applies across the Antarctic Treaty Area, including marine environments south of 60°S, many Southern Ocean conservation threats are discussed across multiple ATS decision-making bodies rather than exclusively through CCAMLR[28]. Accordingly, our conclusions relate most directly to the Treaty and Protocol, while still providing insights into broader conservation dynamics across the ATS.

This clarification forms part of the expanded "Study limitations and considerations" section presented in Major Update #3.

5. Line 107: Basis for selecting the 'ten conservation threats'—The manuscript states that the analysis 'focused on ten conservation threats identified as among the most pressing for the Antarctic region'. While the supplementary material appears to provide additional context for this selection, the rationale remains underdeveloped in the main text. Given that the threat taxonomy anchors all subsequent analysis and interpretation, the authors should more clearly summarise in the manuscript itself the criteria used to identify these ten threats. In particular, it would be helpful to clarify whether the designation of 'most pressing' reflects citation frequency, assessed ecological severity, governance relevance within the ATS, expert judgment, or a combination thereof, and to briefly indicate which categories of threats were considered but ultimately excluded. Without a concise and explicit statement of these criteria in the main text, readers may find it difficult to assess the robustness and scope conditions of the analysis, even if further detail is available in the supplementary materials.

We agree that the rationale underpinning the selection and consolidation of the conservation threats required clearer explanation in the main text. We therefore revised this section to explicitly state that the threats were initially identified through structured expert assessment focused on Antarctic conservation challenges. We also clarified that the final set of ten threats reflects subsequent consolidation and filtering steps undertaken to support robust topic-modelling analysis, with complete details provided in Supplementary Information 1.

We now state (beginning on line 113):

These threats were selected from a broader set identified through structured expert assessment as among the most pressing conservation challenges facing the Antarctic region[21,22,36]. Conceptually overlapping threats were consolidated (e.g., extreme precipitation within broader climate change impacts), while threats with limited representation in the scientific and policy literature (e.g., geoengineering and permanent human settlement) were excluded because they could not be meaningfully compared. Further details of threat identification, synthesis, and classification are provided in the Methods and Supplementary Information 1.

6. Line 138 (Table 1) – Classification of SCAR documents under 'Functioning of the ATS' Table 1 classifies SCAR-related documents under the category "Functioning of the ATS," which requires further clarification. SCAR is an independent scientific organisation and not an institutional component of the Antarctic Treaty System, and its activities do not constitute ATS functioning in a legal or governance sense. If SCAR documents are included within this category, the authors should clarify whether they are intended to serve as proxies for scientific advisory influence on ATS deliberations, or whether their inclusion reflects patterns of discursive interaction rather than institutional authority or decision-making. It seems that drawing a clearer conceptual boundary between scientific advisory functions/entities and treaty based governance functions would strengthen the analytical coherence of the classification and avoid conflating distinct functions within the Antarctic governance landscape.

The reviewer raises an important point regarding the interpretation of the illustrative documents presented in Table 1. We agree that SCAR is an independent scientific organisation rather than an institutional component of the ATS. The inclusion of this particular SCAR meeting report within the *Functioning of the Antarctic Treaty System* conservation threat category does not reflect SCAR's institutional status within the ATS. Rather, the document was strongly associated with this topic because its substantive content focused extensively on Antarctic governance processes, including responses to ATCM recommendations, environmental management activities, and interactions between SCAR and Treaty Parties. Consequently, the document received a high probability for the topic that we subsequently interpreted as relating to the functioning of the ATS (please see below for the full report reference).

More broadly, this comment highlights an important feature of our topic-modelling approach. Conservation threats are assigned on the basis of document content rather than institutional affiliation. Each document receives a probability distribution across all identified topics, reflecting the extent to which it engages with multiple themes simultaneously. Consequently, different SCAR meeting reports may be associated with different conservation threats depending on their substantive content rather than the organisation from which they originated.

Because topic interpretation necessarily involves a degree of judgement, we also strengthened the revised manuscript by introducing an additional validation analysis evaluating agreement between model-derived conservation threat assignments and independent manual

classifications of the underlying documents. Overall, these analyses demonstrated strong agreement between the topic-model assignments and manual classifications, indicating that the assigned conservation threats generally reflected the substantive content of the documents. Where disagreement occurred, it was typically confined to conceptually related threats, reflecting overlap in their underlying drivers, governance mechanisms, or areas of scientific inquiry.

Complete details of this validation are provided in Supplementary Information 4.3. We additionally summarise its principal findings in the new “Technical validation” subsection of the Methods (beginning on line 705 of the revised text).

To further clarify the interpretation of Table 1, we have added a footnote to the "Characteristic documents (classified by topic model)" column. This note explains that the documents are representative examples associated with topics assigned to each conservation threat and that topic assignments are probabilistic rather than exclusive. We believe this clarification more accurately reflects the underlying analytical framework and helps distinguish topic-level interpretation from document-level classification.

Scientific Committee on Antarctic Research (SCAR). Fifteenth Meeting of Scar, Chamonix 16 to 26 May 1978. *Polar Record* **19**, 295-322, doi: https://doi.org/10.1017/S0032247400018416 (1978).

Reviewer #2

1. This manuscript addresses an important question: how conservation threats in Antarctica are reflected in scientific and policy discourses. The topic is relevant, and the authors should be commended for the clarity of their writing, the paper's overall structure, and the quality of the figures, which make the work accessible to a broad audience. The illustrations are well designed and visually compelling.
   Antarctic conservation itself is not my primary area of expertise. My core expertise lies in systematic literature reviews, topic modelling, and natural language processing (NLP). My comments should therefore be interpreted primarily through a methodological lens. Also, I have an injury in both of my arms and can only voice type. Therefore, AI was used to polish the comments that I dictated to the app "Voicy". However, the entire content is my own.
   From this perspective, while the paper is promising, the current methodological framework does not yet support the strength of the conclusions drawn. In particular, there is a mismatch between what the methods used can reliably measure (patterns of attention or salience in text corpora) and what the paper claims to identify (prioritisation of conservation threats). Therefore, I recommend rejecting the paper.

   Thank you for taking the time to review our manuscript and, in particular, for your detailed commentary on the NLP and topic modelling framework. We took these methodological concerns very seriously and made a concerted effort to address all of them throughout the revised manuscript and Supplementary Information.

   In response to your suggestions, we undertook two major sets of revisions. First, we conducted a series of additional validation and sensitivity analyses spanning all major stages of the analytical workflow. these analyses supported the reliability of the modelling framework while also identifying important analytical constraints that have now been incorporated into the revised manuscript. Second, we substantially revised the conceptual framing of the study to better align our conclusions with what can be directly inferred from the underlying data. In particular, we shifted the focus of the study from inferring prioritisation of conservation threats to examining patterns of engagement within the scientific and policy corpora. We accordingly also revised the interpretation of these patterns throughout the manuscript to ensure that our conclusions remain commensurate with the evidentiary limits of the analytical framework.

   The validation and sensitivity analyses are presented in Supplementary Information 4 and are described in full in Major Update #1. The revised conceptual framing extends throughout the manuscript and cannot be fully reproduced within the response letter; however, the principal changes are summarised in Major Update #2.

   We believe these revisions have substantially strengthened the robustness of the study and provide a stronger foundation for the conclusions drawn.

2. Conceptual framing: prioritisation vs. attention

- The goal of the paper is to investigate how conservation threats have been prioritised in scientific and policy corpora (line 103). However, the applied methods primarily capture attention, salience, or frequency of discussion, not prioritisation in a normative or decision-making sense. This distinction is critical and should be reflected consistently throughout the paper.
- Line 114: the wording refers to "mining attention," which is methodologically accurate and, in my view, better reflects what the analysis does.
- I recommend reformulating the research questions, framing, and conclusions to focus on attention, prominence, or thematic salience, unless additional evidence is introduced that directly supports claims about prioritisation.

We agree that this distinction is important and that our original framing placed greater interpretive weight on document frequencies than could be directly supported by the underlying data. We also agree that topic modelling is fundamentally a tool for identifying patterns of thematic attention and salience within text, rather than directly measuring priority-setting.

In response, we substantially revised the conceptual framing of the manuscript. Specifically, we no longer interpret document frequencies as evidence of conservation prioritisation. Instead, the revised manuscript examines how the scientific literature and the ATS engage with conservation threats through time, and interprets these engagement dynamics alongside the ATS's deliberative processes, institutional architecture, and policy outputs. This distinction is important because patterns of engagement can be observed directly through the documentary record, whereas prioritisation and institutional effectiveness require additional causal inferences that cannot be established from document frequencies alone.

This conceptual revision required substantial changes throughout the manuscript. We restructured the aims and objectives of the study, revised the interpretive framework used in the Discussion, and adopted more restrained conclusions that remain closely aligned with the evidence provided by the documentary record. These changes are summarised in Major Update #2 and carried through to the revised Conclusions in Major Update #4.

3. Corpus construction and inclusion/exclusion criteria
   - A major limitation of the study is the absence of clear inclusion and exclusion criteria for the documents included in both the scientific and policy corpora.
   - The search query is very broad and is not systematically evaluated, making it difficult to assess what is actually contained in the corpus.
   - Without knowing what proportion of documents are truly relevant (false positivies/negatives), it is hard to trust downstream topic modelling results.
   - A sensitivity analysis of the search terms is essential. Using the same query, it is possible to retrieve documents that are clearly outside the intended scope. Examples of documents captured by the current query include:
     - "7β-22 Dihydroxyhopane, Isolated from the Sub-Antarctic Lichen, Inhibits the Viability and Stemness in Glioma Stem Like Cells"

- "A Really Dark Landscape": Cormac McCarthy's Blood Meridian and Modest Mouse's The Moon & Antarctica.

These examples highlight the risk of false positives.

- I strongly suggest:
  - Defining explicit inclusion/exclusion criteria,
  - Reporting the expected rate of irrelevant documents (recall, precision),
  - Potentially introducing a simple ML classification step (e.g. scientific BERT) after the keyword search to filter for genuinely relevant documents before topic modelling.

We agree that the reliability of downstream topic modelling depends fundamentally on careful construction of the corpus, and that the original manuscript did not sufficiently evaluate our selected retrieval strategy. In response, we introduced a new relevance-validation analysis that explicitly evaluates the accuracy and limitations of the search strategy. This analysis was conducted only for the scientific corpus because the policy corpus was not assembled through keyword-based retrieval. Instead, it comprised the complete set of English-language Working Papers and Information Papers obtained directly from the Antarctic Treaty Secretariat (see the paragraph beginning on line 571 of the revised main text for complete details).

To summarise this analysis for the scientific corpus, we compared the two dominant retrieval approaches used throughout the Antarctic bibliometric literature: a more targeted strategy, which underpins our scientific corpus, and a broader wildcard-only approach. Precision was quantified through manual validation of randomly sampled abstracts drawn from each of these corpora to estimate precision within ±5% at the 95% confidence level (n = 384 abstracts per retrieval approach). This analysis showed that our targeted strategy achieved substantially higher precision (88%, 95% CI: 85–91%) than the broader wildcard-only approach (79%, 95% CI: 75–83%). Importantly, 91% of false positives were boundary cases with some degree of Antarctic relevance rather than completely unrelated records. This suggests that retrieval errors were dominated by ambiguous or peripheral cases rather than clearly irrelevant records.

We believe that this analysis addresses the reviewer's major concerns regarding corpus construction and retrieval uncertainty because it: (i) defines explicit inclusion and exclusion criteria regarding what constitutes a relevant scientific document; (ii) quantifies retrieval precision through formal validation analyses; and (iii) provides a transparent assessment of the nature and structure of retrieval errors.

Complete details of this analysis are provided in Supplementary Information 4.1. We additionally summarise the major findings in the new "Technical validation" subsection of the Methods (beginning on line 698 of the revised manuscript).

4. Topic modelling
   - The use of LDA is a reasonable starting point, but it is an non-state-of the art model by current NLP standards and is known to be sensitive to preprocessing and hyperparameter choices. You

will always obtain a topic solution with LDA; therefore, the key question is why this particular solution should be trusted.

- Line 479: LDA does not deterministically assign a topic to each word; it infers probabilistic topic–word and document–topic associations. The wording should be corrected accordingly.

We agree that newer topic-modelling approaches are available and that the robustness of any selected model should be demonstrated rather than assumed. Our objective, however, required a model that directly estimates document-topic mixtures which can be aggregated consistently across years and corpora to quantify temporal changes in engagement. LDA provides these probabilistic document-topic distributions as a primary model output and was therefore well suited to the aims of this study. We expanded the Methods (beginning on line 623 of the revised text) to clarify this rationale and added a new Supplementary Information section (SI 1.2.2) discussing the suitability of LDA relative to alternative topic-modelling approaches.

To evaluate the robustness of the inferred topic structure, we also introduced two complementary sensitivity analyses. The first examined the influence of alternative vocabulary construction strategies on the inferred topic structure, while the second evaluated the sensitivity of the major temporal patterns to alternative LDA hyperparameter specifications. These analyses showed that the principal temporal patterns identified in the study remained broadly consistent across alternative vocabulary specifications and LDA hyperparameters (see response to Comment 5 below for complete details).

Finally, we corrected the description of LDA throughout the manuscript to accurately reflect its probabilistic structure (see the revised Methods, beginning on line 623).

5. Topic modelling (continued)
   - The choice to remove the most frequent 15% and rarest 10% of terms is non-trivial and appears arbitrary. Were alternative thresholds tested?
   - Hyperparameter sensitivity for the LDA model is not explored. Without such testing, it is unclear whether the identified topics are robust or artefacts of specific modelling choices.
   - A sensitivity analysis (e.g. varying vocabulary thresholds, α and β values, or number of topics) would substantially increase confidence in the results.

We agree that LDA models can be sensitive to preprocessing decisions and hyperparameter specification, and that these sources of uncertainty were not adequately evaluated in the original submission. In response, we conducted two complementary sensitivity analyses examining the robustness of the modelling framework to alternative vocabulary specifications and model parameterisations. The first examined sensitivity to vocabulary construction by systematically varying the document-frequency thresholds used to define the model vocabulary. The second evaluated sensitivity to model specification through variation in key LDA hyperparameters, including α and β values.

To summarise these analyses:

- Sensitivity to vocabulary construction: We systematically varied the document-frequency thresholds used to define the model vocabulary and evaluated paired minimum–maximum thresholds of 5–95%, 10–90%, 15–85%, and 20–80% across multiple plausible α–β parameterisations. Our analysis showed that topic coherence increased monotonically with more restrictive vocabulary thresholds, but that increasingly restrictive trimming also removed progressively larger proportions of semantically meaningful vocabulary. We therefore retained the 15–85% threshold as a balanced configuration because it maintained strong topic coherence while limiting the loss of semantically meaningful vocabulary.

- Sensitivity to LDA hyperparameters: We systematically varied the Dirichlet priors α and β while holding both the vocabulary specification and number of topics constant. Specifically, we evaluated four alternative α–β parameterisations alongside the reference model used in the primary analysis to assess whether the inferred temporal dynamics of conservation threats remained robust to variation in model priors. For each parameterisation, we repeated the entire downstream analytical workflow, including topic assignment, spline fitting, and identification of significant periods of directional change. Across parameterisations, the principal temporal dynamics identified in the primary analysis remained broadly consistent, with similar statistically significant directional patterns recovered across models. However, the exact magnitudes of fitted spline responses were sensitive to parameterisation.

  These analyses indicate that the modelling approach is robust for identifying broad directional patterns in engagement with conservation threats through time, but that the exact temporal trajectories inferred for individual threats should not be interpreted as precise quantitative estimates. In other words, the framework can robustly identify periods in which engagement with a conservation threat increased or declined through time, but not the exact magnitude of those changes. This finding led us to explicitly acknowledge this limitation in the revised Discussion and to temper our interpretation of the fitted spline responses accordingly (see Major Update #3 for complete details in context).

  Complete details of both sensitivity analyses are provided in Supplementary Information 4.4. We additionally summarise the major findings in the new "Technical validation" subsection of the Methods (beginning on line 705 of the revised manuscript).

6. Topic modelling (continued)
   - The manuscript mentions qualitative validation, but this does not appear to have been conducted in a systematic manner. (e.g. what is the F1 score compared to manual validation)?
   - Topic coherence and exclusivity are useful internal metrics, but they are insufficient on their own.
   - I would strongly encourage:
     - Labelling a subset of documents according to conservation threat categories,
     - Comparing model outputs against these labels,
     - Reporting standard evaluation metrics such as precision, recall, or F1 scores.

We agree that the original manuscript did not provide a systematic evaluation of how accurately the topic-modelling framework classified documents into conservation threat categories. In response, we introduced a new validation analysis that quantitatively compares conservation threat classifications inferred from the topic model against manually labelled documents

To summarise this analysis, we manually assigned conservation threat labels to a validation subset of documents and compared these labels against classifications inferred through the topic modelling framework. We then quantified classification performance against these labels using standard evaluation metrics, including precision, recall, and F1 score, to assess how reliably the manually interpreted topic structure captured conservation threat categories across the scientific and policy corpora. Overall, the framework showed strong agreement with manual classification, with macro-averaged precision, recall, and F1-scores of 82.1%, 86.4%, and 83.3%, respectively. Classification performance was broadly consistent across scientific and policy corpora, and most misclassification arose among conceptually related conservation threats rather than completely unrelated categories. In this regard, many apparent false positives arose because the validation required each document to be assigned to a single dominant threat category, whereas the topic-modelling framework permits documents to retain substantial representation across multiple themes.

These analyses directly address the reviewer's recommendations by: (i) manually labelling a randomly selected validation subset of documents according to conservation threat categories; (ii) comparing these labels against classifications inferred through the topic modelling framework; and (iii) reporting standard classification metrics including precision, recall, and F1 score.

Complete details of this validation analysis are provided in Supplementary Information 4.3. We additionally summarise the major findings in the new "Technical validation" subsection of the Methods (beginning on line 705 of the revised manuscript).

7. Interpretation of percentages and topic prevalence
   - It is unclear how topic percentages are computed:
     - Is each document weighted equally?
     - Are percentages based on topic proportions per document, or on token-level aggregation across the corpus?
   - This should be clarified explicitly, as it affects interpretation.

We agree that the original manuscript did not clearly explain how annual topic prevalence percentages were calculated. To clarify, topic prevalence was calculated using the probabilistic topic composition inferred for each document by the LDA model. Consequently, each document contributed proportions of multiple topics that together summed to one, irrespective of document length. Annual topic percentages were then calculated by summing these document-level topic proportions within each year and expressing them relative to the total number of documents published annually within each corpus. As such, each document contributed equally to the annual totals irrespective of document length, although the

contribution of individual topics varied according to the topic proportions inferred for each document.

We have now added additional context to clarify this point, beginning on line 659 of the revised text. The paragraph now reads as follows:

> We analysed the annual topic proportions generated from our LDA model to examine how scientific and policy attention to conservation threats changed over time. For each document, the LDA model inferred a probabilistic distribution of topic proportions that summed to one across all topics. Annual topic percentages were then calculated by aggregating these document-level topic proportions within each year and expressing them as a proportion of the total number of documents published annually within each corpus. As such, each document contributed equally to the analysis irrespective of document length.

8. Interpretation of percentages and topic prevalence (continued) – Figure 1: the percentages shown are confusing. External, transboundary, and internal threats do not sum to 100. What is the denominator? How many documents are included in the scientific vs. policy corpora? Add to the legend For subclasses (e.g. transboundary threats), percentages should be shown relative to their parent category. The figure legend should clearly explain what the percentages refer to.

We agree that, without further clarification in the figure legend, the way in which we presented the percentages was confusing. Although the reviewer suggested expressing subclasses as percentages of their parent categories, we retained a common denominator because it allows direct comparison of threat prevalence both within and among the higher-level threat categories. The percentages therefore do not sum to 100% because Figure 1 displays only the subset of topics assigned to the conservation threats examined in this study. Topics not assigned to conservation threats are omitted from the figure and account for the remaining proportion of each corpus.

We have refined the figure legend to clarify the denominator used, report the number of documents included in each corpus, and explain how the percentages should be interpreted.

The figure legend now reads as follows (beginning on line 138 of the revised text):

> **Figure 1.** Overview of Antarctic conservation threats and their representation in science and policy. Illustrations depict the ten conservation threats analysed in this study and their representation across the scientific (n = 85,096) and ATCM policy (n = 5,924) corpora (1961–2023). Threats are grouped according to where the processes generating them originate relative to the Antarctic Treaty Area (60° South latitude; yellow circular boundary), distinguishing between External, Transboundary, and Internal threats. Bar charts (left) show the aggregate prevalence of each threat category across the full corpus, while pie charts show the prevalence of individual conservation threats, expressed as percentages of all documents within the corresponding corpus (scientific or policy). Percentages do not sum to 100% because only topics assigned to conservation threats are shown; the remaining proportion corresponds to topics not assigned to conservation threats.

9. Other comments

- Line 41: the term “regime” is used without definition and should be clarified.

  We agree that the term "regime" may be unfamiliar to some readers, particularly outside the governance literature. To improve clarity and accessibility, we revised the Abstract so that the Antarctic Treaty System (ATS) is introduced explicitly before the term "regime" is used. Throughout the revised manuscript, "regime" is used as shorthand for the Antarctic Treaty System once this has been clearly established.

- The manuscript uses many acronyms (e.g. WPs, IPs), some of which have multiple meanings in other contexts. These should be written out in full where possible.

  We agree that some acronyms may be unfamiliar to readers outside the Antarctic governance community. We therefore reviewed the manuscript to ensure that all acronyms are defined at first use and, where appropriate, replaced field-specific abbreviations with their full terms. In particular, we replaced WPs and IPs with their full terms (Working Papers and Information Papers) throughout the manuscript and clarified figure captions where abbreviations were used without accompanying definitions.

- It would be helpful to report the average length of WPs and IPs compared to abstracts, to assess whether topic distributions may be driven by document type rather than conservation content.

  We agree that scientific abstracts and ATCM policy documents differ substantially in length and structure, and that these differences could potentially influence inferred topic distributions. While reporting average document lengths would describe this difference, it would not directly evaluate whether document type influenced the inferred topic structure. We therefore used the revision process as an opportunity to explicitly examine whether topics showed systematic differences between scientific and policy documents.

  To summarise this analysis, we examined the composition of each topic across the scientific and policy corpora using the fitted LDA model. Specifically, we calculated the mean document-level topic proportion for each topic separately for scientific and policy documents and tested for consistent directional differences using a paired Wilcoxon signed-rank test. We found no evidence that the inferred topic structure was systematically skewed towards either scientific or policy documents across the full topic set ($p = 0.99$), with similar results when the analysis was restricted to topics included in the conservation-threat framework ($p = 0.29$). These findings suggest that differences between scientific abstracts and policy documents do not produce a consistent directional bias in the inferred topic structure and therefore provide little evidence that document type is driving the principal patterns reported in the manuscript.

  Complete details of this analysis are provided in Supplementary Information 4.2. We additionally summarise the major findings in the new "Technical validation" subsection of the Methods (beginning on line 705 of the revised manuscript).

- Semantic variants (e.g. "outside Antarctica" vs. "beyond Antarctica") and intruder terms (e.g. bacteria, microbes) remain in the corpus and should be more systematically addressed.

We agree that semantic ambiguity and intruder terms are an important consideration in keyword-based retrieval. To evaluate their influence on our corpus, we examined all false-positive records identified during corpus validation. Of the 384 records examined, 46 were classified as false positives, and only 4 of these records (8.7%) represented genuine lexical or indexing artefacts. The remaining false positives (42 of 46 records; 91.3%) were boundary cases in which Antarctic relevance was present but not primary, including comparative studies, incidental references to Antarctica, and studies focused on regions physically connected to Antarctica. Consequently, retrieval errors were driven primarily by documents lying near the boundary of the study scope rather than by semantic artefacts that could be readily removed through additional filtering. These findings suggest that semantic ambiguity and intruder terms are not a dominant feature of the corpus and are unlikely to exert a substantial influence on the broad patterns examined in this study.

Please see Supplementary Information 4.1 for complete details.

- Line 120: the manuscript assumes that abstracts are broadly representative of full texts. Previous work (including my own) has shown that abstracts often omit nuance, methodological detail, and secondary themes that are present in full texts. While the use of abstracts is understandable for scalability, this limitation should be acknowledged more explicitly, and its implications for topic interpretation should be discussed.

We thank the reviewer for this clarification and agree that the limitations associated with using abstracts were not sufficiently acknowledged in the original manuscript. Although the use of abstracts is indeed common in bibliometric and topic modelling studies, we agree that they do not fully capture the nuance, methodological detail, and secondary themes present within full-text articles. In response, we have amended the sentence identified by the reviewer, expanded the discussion of this limitation in the Methods, and added a supporting citation.

We have revised the sentence identified by the reviewer as follows (beginning on line 123 of the revised text):

> We used abstracts to enable large-scale, consistent comparison across the scientific literature, recognising that they capture the principal themes of each study but not its full nuance.

We now additionally state (beginning on line 595 of the revised text):

> The Scopus scholarly database was selected over other platforms (e.g., Google Scholar) because it allows efficient web scraping to download large volumes of metadata. While Scopus may not capture all scientific literature, the ~85,000 abstracts retrieved are broadly representative of peer-reviewed research on Antarctic-related topics. We acknowledge, however, that abstracts provide only a condensed representation of

individual articles and may omit methodological detail, contextual information, or secondary themes present within the full text. Nevertheless, abstracts are widely used in large-scale bibliometric and topic modelling analyses where full-text access is impractical[81]. Our analysis should therefore be interpreted as reflecting the dominant themes emphasised within the scientific literature rather than every aspect of the underlying article.

We had additionally added these details to our limitations section (beginning on line 406 of the revised text; see Major Update #3 for complete details):

> Finally, our reliance on scientific abstracts and ATCM Working Papers and Information Papers limits access to the full nuance of debates and excludes informal or undocumented exchanges[9,81]. These sources nevertheless provide the most consistent and reproducible record of ATCM discourse.

Citation: Atanassova, I., Bertin, M., & Larivière, V. (2016). On the composition of scientific abstracts. *Journal of Documentation, 72*(4), 636-647. doi:https://doi.org/10.1108/JDOC-09-2015-0111

- Figure 3, while visually appealing, is difficult to interpret. Clear patterns are not immediately apparent. I recommend reconsidering how this information is visualised, potentially through more aggregated or comparative representations that align more closely with the research questions.

We appreciate the reviewer's perspective regarding the interpretation of Figure 3 and agree that the figure can be challenging to interpret at first glance. We therefore reconsidered both the figure and its accompanying explanation. Although we considered alternative visualisations, we retained the original figure because it provides a compact overview of the timing and duration of significant changes in engagement across all conservation threats and both corpora within a single comparative framework. We concluded that the primary limitation may therefore relate to the accompanying explanation rather than the visualisation itself. Accordingly, we substantially revised the figure legend to clarify how periods of significant change were identified, what the shaded intervals represent, and how the figure should be interpreted.

The figure legend now reads as follows (beginning on line 239 of the revised text):

> **Figure 3.** Periods of significant change in scientific and policy attention to major conservation threats, identified from the spline curves shown in Figure 2 (1961–2023). Shaded intervals indicate periods during which engagement with a conservation threat increased or decreased significantly, providing a comparative overview of how engagement has changed through time across the scientific and policy corpora. Green (▬) and purple (▬) shading represent periods of significant growth within the scientific and ATCM policy corpora, respectively, while grey (▬) shading indicates periods of significant decline. Threats are grouped according to where the processes generating them originate, relative to the jurisdictional boundaries of the Antarctic Treaty Area. ‘sci’ = science; ‘pol’ = policy.

Reviewer #3

1. This paper is an interesting read, and I salute the team for the underpinning work - which is substantial. But let me offer you a provocative reading of the paper rather than an endorsement.

   We thank the reviewer for taking the time to review our manuscript and for providing such thoughtful and thought-provoking comments on the substance of our work. Indeed, these comments prompted us to reflect critically on the conceptual framing of the study and how our findings should be interpreted within broader debates surrounding Antarctic governance. Following these comments, we substantially revised the framing of the manuscript to better align our interpretations with the evidence generated by the analysis. These revisions extend throughout the manuscript, with particularly substantial changes to the Introduction, Discussion and Conclusions sections. We believe the revised manuscript presents a more balanced and defensible interpretation of our findings.

2. Given the location of the team itself is there any intrinsic surprise that Australian and New Zealand-based academics would conclude ultimately that the ATS is doing a good job - “institutionally effective”. There is in both countries a well-established academic-policy-media complex that would largely reproduce that over time in part because both countries are claimant states and original signatories - the treaty has been widely regarded as integral to national security as well as offer broader merit in terms of peace and cooperation. For both countries, the Southern Ocean/Antarctica is part of their backyards.

   The reviewer raises an important point. We recognise that questions concerning the effectiveness, legitimacy, and overall success of the ATS are inherently contested and may be interpreted differently by scholars operating in different political, geographical, and disciplinary contexts.

   Reflecting on this comment, we concluded that the reviewer's concern likely stemmed from how we framed the original manuscript. In the previous version, we used terms such as “institutional effectiveness” and “fit for purpose” to describe the implications of our findings. We now agree that these terms implied a broader evaluation of the ATS than our analysis could directly support. Our study does not assess whether the ATS is successful, legitimate, or effective as a system of environmental governance. Rather, it examines how conservation threats are incorporated into the ATS's deliberative processes and what those patterns may suggest about the regime's capacity to recognise, deliberate on, and develop responses to them.

   In response, we have substantially revised the conceptual framing of the manuscript. The revised version focuses on capacity and how that capacity is exercised across different categories of conservation threat. Specifically, rather than asking whether the ATS is effective, we examine how patterns of engagement vary across conservation threats and what those patterns reveal about the regime's capacity to recognise, deliberate on, and develop responses to them. This framing more closely aligns our conclusions with the evidence generated by the analysis and avoids conflating patterns of engagement with broader normative judgments regarding governance effectiveness or legitimacy.

Importantly, this revised framing does not lead us to conclude that the ATS is uniformly successful. Instead, our findings suggest that the regime engages differently with different categories of conservation threat. For threats that fall clearly within its jurisdiction, engagement trajectories are consistent with capacity being exercised through the deliberative processes envisaged by the regime's governance framework. For threats whose primary drivers originate beyond the Antarctic Treaty Area, however, the evidence suggests that the ATS is not fully exercising the capacity afforded by its governance framework despite the availability of mechanisms for broader external engagement. We believe this interpretation better reflects both the strengths and limitations revealed by our analysis.

We note that these revisions extend throughout the manuscript. For ease of reference, key excerpts illustrating the revised conceptual framing are summarised in Major Update #2. Because this reframing affected the interpretation of our findings, it also resulted in substantial revisions to the Conclusions and Recommendations section, which are provided in Major Update #4.

3. The paper's design and sources/methods cannot get at something rather important about the ATS and that is it foundational working culture - the treaty was designed at the onset to be geographically focussed, with a hope in 1959 that membership would remain limited. No one could imagine or wanted to imagine China being a consultative party. Recall the reaction to India back in the 1950s. The US and its special allies including both Australia and New Zealand assumed the ATS would be largely directed by their collective knowledge and commitment. The shift towards conservation in the 1960s was an experiment to see whether the ATS could expand its remit through new conventions on conservation-related matters, while retaining the all important consensus principle. The 1980s were pivotal in terms of really stressing and straining what John Heap described as the "Antarctic club". And it is no surprise that policy would focus on "internal threats" as that was hard-wired into the system right at the start. The ambivalence about the UN came out clearly in the 1980s.

We agree with the reviewer that the ATS emerged through the deliberate avoidance of sovereignty disputes and developed a diplomatic culture centred on controversy avoidance. These foundational characteristics have undoubtedly shaped how issues enter the regime's deliberative processes and the extent to which Parties are willing to engage with particular conservation challenges. We therefore revised the Discussion to place our findings within this broader historical and institutional context, suggesting that the ATS's original regional orientation may have contributed to its comparatively limited engagement with conservation threats whose primary drivers originate beyond the Antarctic Treaty Area.

The paragraph reads (beginning on line 367 of the revised main text):

> The historical origins of the ATS may partly explain why these mechanisms for external engagement have not been fully utilised. The regime emerged at a time when Antarctica was comparatively isolated from wider geopolitical and economic processes and developed as a regionally oriented governance regime focused primarily on activities occurring within the Antarctic Treaty Area[13,79]. As conservation threats have increasingly been driven by processes operating beyond the Treaty Area[22], this regional

orientation may have fostered an institutional preference for regional solutions, leaving existing mechanisms for external engagement comparatively underutilised.

We believe this historical perspective complements and strengthen our interpretation of the observed patterns. Indeed, if the ATS's historical regional orientation continues to shape contemporary governance, then expecting the ATS to independently resolve threats whose primary drivers originate beyond the Antarctic Treaty Area may be unrealistic. Under this interpretation, strengthening connections with external governance frameworks becomes important because it allows the ATS to remain focused on Antarctic affairs while drawing upon governance systems operating at broader scales. In this sense, functioning as a regional node within a wider governance network may allow the regime to address increasingly global conservation challenges without requiring a fundamental departure from the institutional characteristics that have shaped its development.

At the same time, we do not consider this historical perspective to fully explain the observed patterns, as a complementary body of scholarship emphasises the ATS's capacity for institutional adaptation through time. Indeed, a substantial body of scholarship describes the ATS as having undergone successive periods of consolidation, institutional change, and stabilisation, during which environmental protection became an increasingly important component of its mandate (e.g. see Scott 2003; Dudeney & Walton 2012; Rothwell 2021; Sampaio 2022). The 1980s are particularly instructive in this regard. Rather than simply exposing the limitations of the ATS's original design, this period demonstrated the regime's capacity to respond to emerging issues through institutional adaptation while retaining many of its underlying diplomatic norms and decision-making principles.

We therefore view the ATS as a system characterised by both continuity and change, with contemporary patterns of governance reflecting both the historical legacies emphasised by the Reviewer and decades of subsequent institutional development. Accordingly, we revised the Discussion to avoid privileging a single interpretation of these patterns where multiple explanations remain plausible. The trajectories associated with external threats are therefore interpreted as being consistent with several complementary explanations, including the historical dynamics identified by the reviewer.

This revised interpretation is reflected throughout the Discussion and is illustrated in the revised Discussion excerpt provided in the second half of Major Update #2.

References:

- Dudeney JR, Walton DWH. 2012. Leadership in politics and science within the Antarctic Treaty. Polar Research.
- Rothwell DR. 2021. The Antarctic Treaty at sixty years: Past, present and future. Melbourne Journal of International Law **22**:F1+.
- Sampaio DP. 2022. Diplomatic culture and institutional design: Analyzing sixty years of Antarctic Treaty governance. Anais da Academia Brasileira de Ciências **94**.
- Scott K. 2003. Institutional Developments within the Antarctic Treaty System. International and Comparative Law Quarterly **52**:473-487

4. Effectiveness travels together with legitimacy and I do wonder whether a great deal of what you describe is a system seeking to cope with a legitimacy crisis - the regional framing coming under pressure both politically and ecologically. Your focus on conservation threats could be seen as two types – the conservation of governance itself and (ecological) conservation governance. I am not sure I really understand your internal, external and transboundary classification - surely that depends on your judgement as you note but actually I would be more interested in how stakeholders made sense of it. Most of the issues blur internal/external and transboundary might also refer to connections with other places thought as global commons such as oceans and space.

The reviewer brings up multiple interesting points that warrant discussion. We first discuss the ATS's legitimacy crisis and then move to our classification of conservation threats.

The reviewer raises an important point regarding the relationship between capacity and legitimacy. Although we changed the framing of the manuscript from institutional effectiveness to capacity, we agree that legitimacy is closely related to the exercise of the capacity, particularly in the context of global governance institutions. To address this comment, we expanded the Discussion to consider how the historical regional framing of the ATS may have influenced its engagement with conservation threats generated beyond the Antarctic Treaty Area. (this paragraph begins on line 367 of the revised main text; please see our response to question #3, above, for additional context).

This discussion is then linked to a new paragraph considering the implications for externally generated conservation threats. This paragraph reads as follows (beginning on line 375 of the revised main text):

> This legacy has important implications because external threats pose some of the most serious challenges facing Antarctic ecosystems[22], yet they are also the issues for which the ATS provides the weakest evidence of progression through its deliberative processes. Several institutional reforms have been proposed to improve how the ATS addresses threats originating beyond the Antarctic Treaty Area, including restricting the decision-making influence of major greenhouse gas emitters, replacing consensus decision-making with majority voting, and strengthening coordination with other international institutions[80]. Many of these proposals would require substantive changes to the ATS's institutional architecture. By contrast, fully exercising the Treaty's existing mechanisms for external engagement may provide one of the most readily available opportunities to strengthen governance responses to threats originating beyond the Antarctic Treaty Area.

We consider this historical perspective to provide important context for interpreting our findings. If the ATS's original regional orientation continues to shape contemporary governance, then expecting the regime to independently resolve threats whose primary drivers originate beyond the Antarctic Treaty Area may be unrealistic. At the same time, we maintain that the ATS possesses existing institutional mechanisms through which it can engage with such threats. For example, Article III.2 of the Antarctic Treaty explicitly encourages cooperation with United Nations Specialised Agencies and other international

organisations with scientific or technical interests in Antarctica. Our interpretation is therefore that the comparatively limited engagement with external threats observed in our analysis may reflect the historical institutional orientation of the ATS and the resulting underutilisation of these existing mechanisms, rather than an absence of institutional capacity.

We also agree that the rationale underlying the classification of threats as internal, external, and transboundary was not sufficiently clear in the original manuscript. To address this, we retained the classification but substantially clarified the rationale and definitions used.

Specifically, we classified threats according to the principal geographical location of the processes that generate direct environmental pressures on Antarctica. Climate change, for example, generates environmental pressures through greenhouse gas emissions released largely outside the Antarctic Treaty Area, whereas mineral and hydrocarbon exploitation would generate environmental pressures through extraction activities occurring within Antarctica. Other threats, such as invasive species, involve processes occurring both within and beyond the Antarctic Treaty Area and were therefore classified as transboundary.

We adopted this framework because a central objective of the study was to examine whether ATS engagement differs among conservation threats that place different demands on the regime. Classifying threats according to the geographical location of their principal pressure-generating processes provides a consistent analytical basis for making this comparison while avoiding circularity with governance arrangements or institutional response.

We also agree with the reviewer that the boundaries between these categories are not absolute. Many threats include processes occurring both within and beyond the Antarctic Treaty Area. For example, although climate change is primarily generated by greenhouse gas emissions released outside Antarctica, emissions generated within the Treaty Area also contribute. For this reason, the classification was based on the principal geographical location of the pressure-generating processes rather than requiring categories to be mutually exclusive.

Finally, we agree that stakeholders may classify conservation threats differently. The conservation threats themselves were identified through expert elicitation, but the internal, external, and transboundary categories represent an analytical framework developed for the purposes of this study rather than a stakeholder-derived typology.

5. In the discussion area, I would be tempted to temper this positive assessment of the ATS - since the negotiation of the Protocol there has been a marked reluctance to tackle bioprospecting, tourism and call out bad behaviour. There has been a loss of confidence and in part this has been brought on by both wider geopolitics but also diversity of membership and external scrutiny. If this capacity has not been realised then why would you conclude it is still fit for purpose. Truthfully, the Antarctic Treaty is an essential accomplice for claimant states and we need to prepare for a world that appears to be drifting towards Neo-imperialism where great powers might simply walk away from treaties. The Trump administration surely needs to be cited in that regard.

We thank the reviewer for this thoughtful comment. We agree that the observation of unrealised institutional capacity raises important questions about whether the ATS should be

characterised as effective or “fit for purpose”. Upon reflection, we concluded that our analysis was not well positioned to answer those broader questions directly. Rather than defending the original terminology, we substantially revised the conceptual framing of the manuscript. This revised version focuses on how the ATS engages with conservation threats and what those patterns reveal about the exercise of institutional capacity, rather than treating them as direct evidence of governance effectiveness. Excerpts of these changes can be found in Major Update #2, while the revised Conclusions section is provided in Major Update #4.

This revised framing does not lead us to conclude that the ATS is uniformly successful. On the contrary, the revised Discussion explicitly acknowledges important limitations and areas where institutional capacity appears underutilised. While many internal threats exhibit trajectories consistent with institutional capacity being exercised through the regime's deliberative processes, this pattern is not universal. For example, we now highlight bioprospecting as a case where the ATCM has historically avoided substantive engagement despite repeated calls from Parties for greater transparency and information exchange. Similarly, for threats whose primary drivers originate beyond the Antarctic Treaty Area, we now conclude that the ATS provides comparatively weak evidence that its available institutional capacity is being fully exercised, despite the existence of mechanisms for broader external engagement.

Accordingly, the Conclusions section has been substantially revised. Rather than arguing that the ATS is “fit for purpose”, we now conclude that a central challenge facing the regime is not an absence of institutional capacity, but how that capacity is exercised across the jurisdictional boundaries that define Antarctic governance. We believe this revised framing better reflects both the strengths and limitations revealed by our analysis and more appropriately captures the concerns raised by the reviewer.

Update 1- Supplementary Information 4: Validation of the topic modelling pipeline

To evaluate the robustness of the analytical framework, we conducted five complementary validation and diagnostic analyses spanning corpus construction, topic modelling, classification performance, and temporal inference (**Figure S4.1**). Automated text analyses can be sensitive to modelling assumptions, preprocessing decisions, and parameter specification, making application-specific validation important when drawing substantive inferences from large corpora (Grimmer & Stewart 2013; Denny & Spirling 2018) Accordingly, these analyses assessed: (i) the precision of the scientific corpus retrieval strategy; (ii) the influence of imbalance between scientific and policy documents on inferred topic structure; (iii) the accuracy of topic-to-threat classifications; (iv) the sensitivity of topic structure to alternative vocabulary filtering strategies; and (v) the sensitivity of temporal dynamics to alternative model parameterisations.

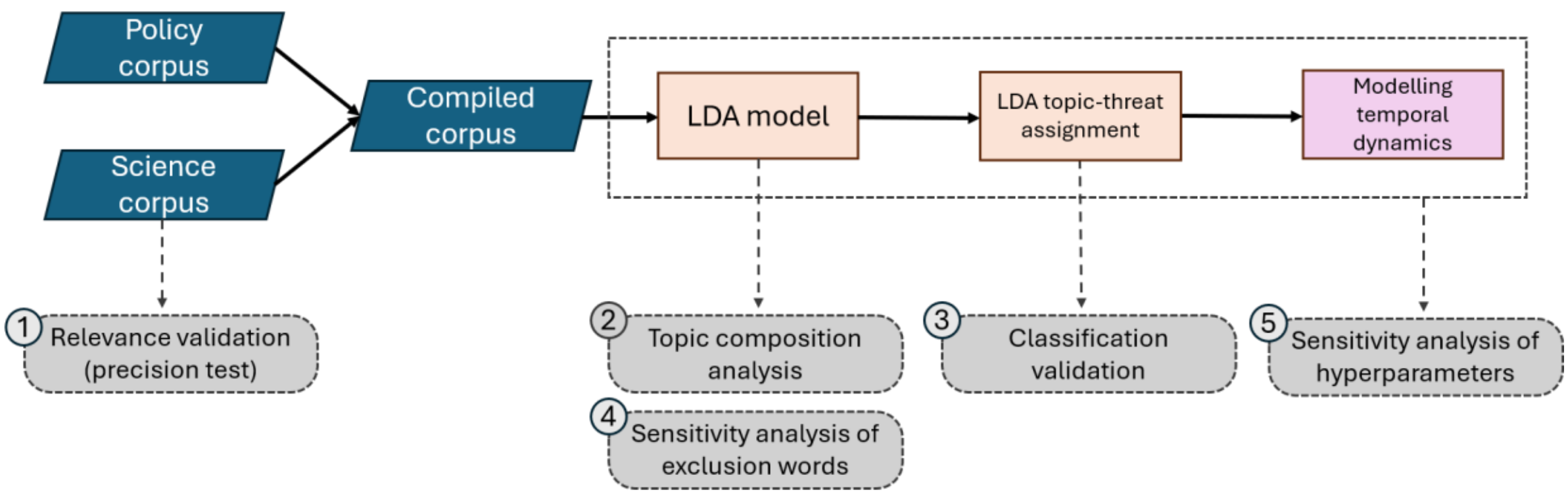

Figure S4.1. Validation and diagnostic assessment of the topic modelling pipeline. The workflow illustrates the progression from corpus compilation through topic modelling, topic-to-threat assignment, and modelling of temporal dynamics. Five validation and diagnostic analyses were conducted across the pipeline: (1) relevance validation, (2) topic composition analysis, (3) classification validation, (4) sensitivity analysis of exclusion words, and (5) sensitivity analysis of hyperparameters. Numbers correspond to the ordering in which analyses are presented in SI 4.

Overall, the validation results supported the reliability of the framework while also identifying important analytical constraints. The scientific corpus retrieval strategy achieved high precision (88%) and outperformed alternative approaches commonly used in Antarctic bibliometric research (e.g., see Dastidar 2007; Dastidar & Ramachandran 2008; Dudeney & Walton 2012; Ji et al. 2014). Despite substantial differences in corpus size, we found little evidence that imbalance between the scientific and policy corpora systematically biased inferred topic structure, either across the full model (paired Wilcoxon signed-rank test, $p = 0.99$) or within the subset of conservation threats examined in this study ($p = 0.29$). Vocabulary filtering analyses further identified a stable trade-off between semantic coherence and vocabulary retention, where increasingly restrictive filtering improved topic coherence but progressively removed larger numbers of meaningful terms. This supported selection of the 15–85% vocabulary threshold as a balanced configuration that improved interpretability while retaining nuanced domain-specific language.

Topic-to-threat classifications also showed good agreement with manually classified documents (macro-averaged F1 = 83.3%), consistent with strong performance for complex thematic classification tasks involving overlapping concepts (Pustejovsky & Stubbs 2013). Importantly, this validation

imposed stricter classification boundaries than the primary probabilistic framework by requiring each document to be assigned to a single dominant threat category, likely producing more conservative performance estimates overall. Finally, the major temporal patterns identified in the analysis remained robust to reasonable changes in model parameterisation, with alternative hyperparameter configurations preserving historically interpretable periods of change. Spline magnitudes were, however, sensitive to parameter specification and so exact spline estimates should therefore be interpreted more cautiously than broader shifts in attention through time.

Complete methodological details and validation results are provided in the following subsections. Associated validation datasets and analytical code are archived within the study's data repository (see the Data Availability Statement in the Main Text for details).

SI 4.1 Relevance validation

SI 4.1.1 Methods

Relevance validation was conducted for the scientific corpus only. The policy corpus consisted of all available ATCM Working Papers and Information Papers retrieved directly from the Antarctic Treaty Secretariat and therefore did not require keyword-based retrieval. In contrast, the scientific corpus could not be exhaustively retrieved a priori and instead required a keyword-based bibliometric retrieval strategy capable of balancing coverage and relevance. The Antarctic bibliometric literature reveals two dominant approaches to corpus construction that balance coverage and relevance in different ways (**Table S4.1**). The first employs targeted keyword strategies that combine "antarct*" with region-specific terms and exclusion criteria to improve specificity (e.g., Gray & Hughes 2016; Gray 2019). The second adopts a more generalist approach, relying solely on the wildcard term "antarct*" to maximise coverage of Antarctic-associated publications (e.g., Dastidar 2007; Dastidar & Ramachandran 2008; Dudeney & Walton 2012; Ji et al. 2014).

Table S4.1. Comparison of bibliometric search strategies used to identify Antarctic scientific literature.

Bibliometric approach	Keyword search[a]	References
1 (targeted keyword approach)	(TITLE-ABS-KEY(antarct* OR "southern ocean" OR "ross sea" OR "amundsen sea" OR "weddell sea")) AND (PUBYEAR > 1960) AND (PUBYEAR < 2024) AND NOT (TITLE-ABS-KEY(candida OR "except antarctica" OR "not antarctica")) AND (LIMIT-TO (LANGUAGE,"English"))	Gray (2019); Gray and Hughes (2016); Hughes et al. (2024)
2 (wildcard-only approach)	(TITLE-ABS-KEY(antarct*)) AND (PUBYEAR > 1960) AND (PUBYEAR < 2024) AND (LIMIT-TO (LANGUAGE,"English"))	Dastidar (2007); Dastidar and Ramachandran (2008); Dudeney and Walton (2012); Ji et al. (2014)

[a]Adjusted for the timeframe of this study and for English-language documents only

These approaches represent two widely used definitions of "Antarctic literature". Rather than constructing a bespoke search strategy, we compared these established retrieval approaches directly. Because our objective was to define a corpus that is comprehensive, transparent, and reproducible, we treated corpus construction as an explicit methodological decision and evaluated each approach empirically.

To determine which approach provided the most suitable corpus for our analysis, we quantified the precision of each retrieval strategy through manual validation of randomly sampled abstracts. Sample size was determined *a priori* to estimate classification precision within ±5% at 95% confidence under a binomial framework (Field 2012), using:

$$n = \frac{Z^2 p(1-p)}{E^2}$$

Where $Z = 1.96$ (critical value for 95% confidence), $E = 0.05$ (margin of error), and $p = 0.5$ (most conservative assumption in the absence of prior information). This yielded a target sample size of 384 abstracts. Given the large size of the corpus relative to the validation sample, finite population correction was not applied (Field 2012).

Abstracts were randomly sampled from each retrieval strategy and independently classified by two reviewers using a predefined relevance schema (see below). Each document was assigned to one of two categories: relevant or irrelevant. Inter-reviewer agreement was assessed prior to consensus using percent agreement and Cohen's kappa to evaluate consistency in the application of the relevance criteria. Disagreements were subsequently reviewed and resolved through discussion between the reviewers, and the consensus classifications were used to calculate the final precision estimates. For the targeted keyword approach, reviewers agreed on 97.1% of classifications ($\kappa = 0.87$). For the wildcard-only approach, agreement was 97.4% ($\kappa = 0.92$). These results indicate a high degree of consistency in the application of the relevance criteria, providing confidence in the reliability of the resulting precision estimates (Landis & Koch 1977).

Relevance criteria were defined *a priori* to ensure consistent and repeatable classification, drawing on established principles from systematic review methodology in conservation science (see Pullin & Stewart 2006). Documents were considered relevant if Antarctica constituted a primary focus of the study and the content focused on understanding environmental systems, ecological processes, human activities, or governance within the region. Documents were classified as irrelevant if Antarctica was mentioned only incidentally, used for comparison, or if the study did not directly engage with Antarctic systems or processes. In cases of ambiguity, classification was based on whether the study's primary research questions and conclusions were centred on the Antarctic region.

Using these classifications, precision was calculated for each retrieval strategy as:

$$Precision = \frac{TP}{TP + FP}$$

where *TP* is the number of abstracts correctly classified as relevant (true positives) and *FP* is the number incorrectly classified as relevant (false positives). This metric quantifies the proportion of retrieved documents that fall within the scope of the analysis. Confidence intervals for precision were calculated using the Wilson method (Brown et al. 2001).

SI 4.1.2 Interpretation

The targeted keyword approach achieved substantially higher precision (88%, 95% confidence interval [CI]: 85–91%) than the wildcard-only approach (79%, 95% CI: 75–83%), indicating that broader retrieval increases the proportion of irrelevant documents (**Table S4.2**). On this basis, the targeted keyword approach was selected to construct the corpus used in our study.

Table S4.2. Performance of bibliometric search strategies based on manual validation of document relevance.

Bibliometric approach	True Positive (TP)	False Positive (FP)	Precision (95% CI)
1 (targeted keyword approach)	338	46	88 (85 – 91) %
2 (wildcard-only approach)	303	80	79 (75 – 83) %

To provide additional transparency, we examined the nature of classification errors to characterise the limitations of the retrieval strategy through manual inspection of all false positive records. False positives were not randomly distributed, but instead clustered within seven recurring categories (**Table S4.3**). The largest proportion arose from studies in which Antarctica was included as a comparative reference (32.6%), followed by incidental lexical matches and studies focused on regions physically connected to Antarctica without being centred on the Antarctic region itself (both 19.6%). These categories capture cases in which Antarctic terminology is present but not central to the study, either through comparison, incidental mention, or broader regional context. Smaller contributions arose from studies in which Antarctica formed one component of broader multi-region or global analyses, rather than the primary focus of investigation (8.7%). Additional false positives arose from indexing or lexical artefacts (8.7%), references to Antarctic logistical infrastructure or field operations (6.5%), and studies using Antarctic-derived samples without substantively engaging with Antarctic systems (4.3%). Excluding indexing or lexical artefacts, which represent genuine retrieval mismatches (n = 4, 9%), the overwhelming majority of false positives (n = 42, 91%) reflect boundary cases in which Antarctic relevance is present but not primary.

Table S4.3. Classification of false positive documents by error type and frequency for the targeted keyword search strategy.

Category	Classification criteria	Error count	% of false positive	% of validation sample (n = 384)
Comparative reference	Studies in which Antarctica is mentioned as a point of comparison, analogy, or contextual reference, but is not central to the research question, analysis, or conclusions.	15	32.6	3.9
Incidental lexical match	Records in which Antarctic-related terms are present in the title, abstract, or metadata but do not reflect substantive engagement with Antarctic systems, processes, or contexts.	9	19.6	2.3
Oceanographic / geographic spillover	Studies focused on regions adjacent to, or dynamically connected with, Antarctica where Antarctic terminology is used in a broader geographic or oceanographic context without the study being centred on the Antarctic region itself.	9	19.6	2.3
Multi-system inclusion	Studies that include Antarctica as one component within a broader, multi-region or global analysis, where Antarctica contributes to the analysis but is not the primary focus.	4	8.7	1.0
Indexing / lexical artefact	Records retrieved due to artefacts of indexing, terminology, or naming conventions, including algorithm names, taxonomic labels, or ambiguous keyword combinations (e.g. “Arctic/Antarctic” terms).	4	8.7	1.0
Logistical presence	Studies in which Antarctica is referenced as a site of logistical activity (e.g. research stations, field sites, or launch locations) or operational infrastructure, without the scientific investigation being directed at Antarctic systems or processes.	3	6.5	0.8
Sample provenance	Studies in which materials, organisms, or samples originate from Antarctica, but the research question, analysis, or interpretation is not focused on Antarctic systems	2	4.3	0.5

SI 4.2 Topic composition analysis

SI 4.2.1 Methods

In our study, we combined the scientific and policy literature into a single corpus so that topics were inferred within a common semantic space. Because LDA derives topics from corpus-specific word co-occurrence structure, fitting separate models to different corpora can produce topic sets that are not inherently aligned, limiting direct comparison of thematic emphasis (Bystrov et al. 2022). Our construction of a unified corpus therefore enabled direct comparison between scientific and policy

documents. However, the combined corpus was strongly imbalanced. On a document basis, there were 5,924 ATCM policy documents and 85,096 scientific abstracts (a ratio of 1:14). Since LDA infers topics from aggregate word co-occurrence patterns, the dominant corpus may exert disproportionate influence on the resulting topic structure.

To assess whether corpus imbalance introduced a systematic bias towards the scientific literature, we compared the average representation of each topic between scientific and policy documents (i.e., the mean topic proportion assigned to documents within each corpus). In LDA, each document is assigned a proportion for every topic, representing how strongly each topic is reflected in the text. Averaging these values across documents therefore provides a measure of topic representation that is independent of the total number of documents within a corpus. Higher average proportions indicate that a topic contributes more strongly to documents within a corpus, whereas similar average proportions indicate comparable representation across corpora.

Differences in mean proportions were then calculated for each topic and evaluated using a paired Wilcoxon signed-rank test to assess whether one corpus consistently exhibited higher representation across topics (Wilcoxon 1945). If corpus imbalance were biasing the model, topics would be expected to exhibit systematically greater representation in scientific documents than in policy documents.

SI 4.2.2 Interpretation

Scientific documents exhibited higher mean topic proportions in 51 topics (56.7%), while policy documents exhibited higher proportions in 39 topics (43.3%) (**Figure S4.2**). Despite this imbalance in counts, differences in topic representation were not statistically significant (paired Wilcoxon signed-rank test, $p = 0.99$) and were negligible in magnitude ($r = 0.001$) (**Figure S4.3a**). This indicates no consistent directional bias in topic representation between corpora and provides little evidence that corpus imbalance resulted in a systematic bias towards the scientific literature. Repeating this analysis only for the subset of topics included in the conservation threat framework yielded the same result, with no significant differences in topic proportions between scientific and policy documents ($p = 0.29$) (**Figure S4.3b**). This provides further evidence that the subset of topics underpinning the study's conclusions is not disproportionately dominated by the scientific corpus.

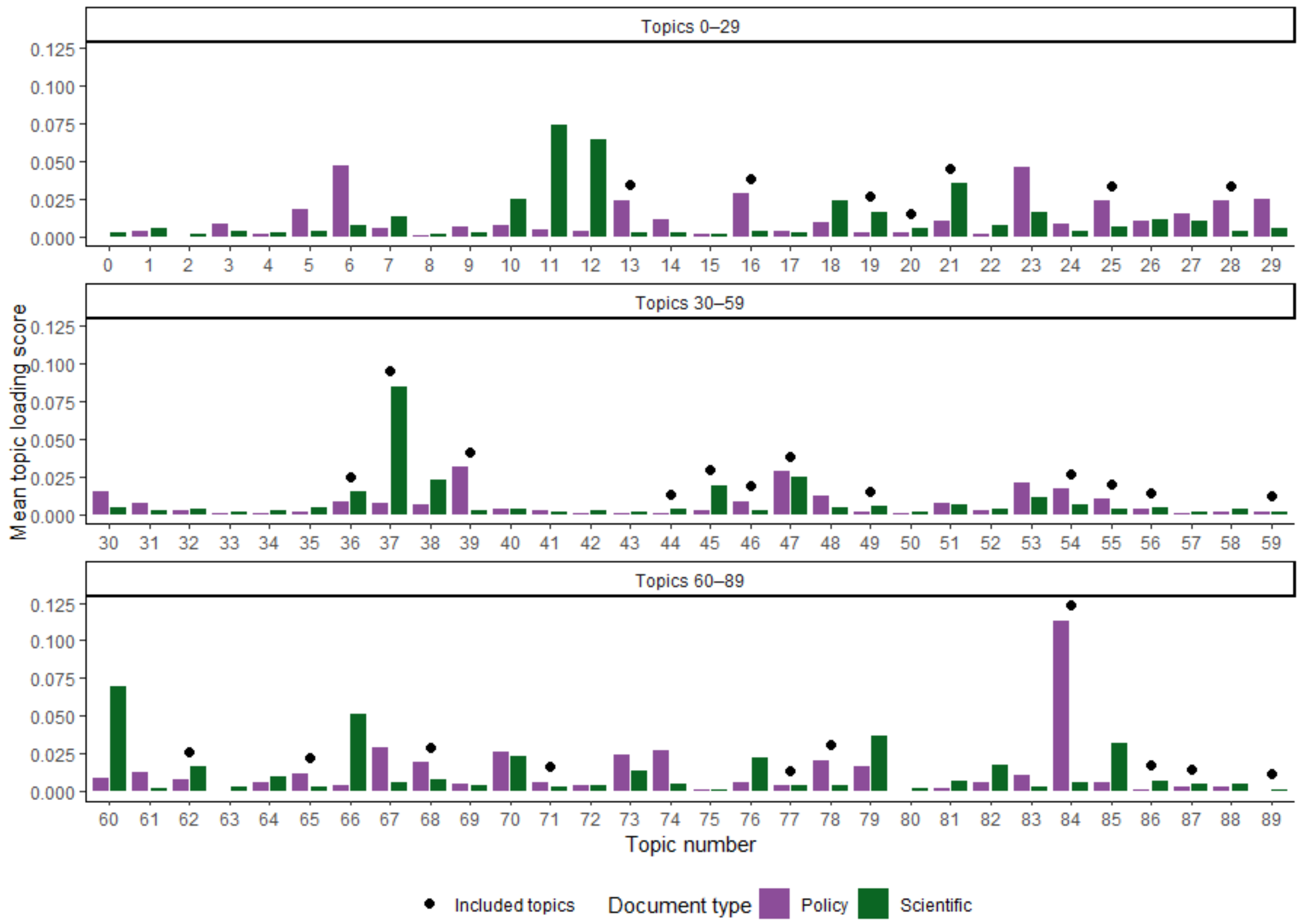

Figure S4.2. Comparison of mean topic loading scores between scientific and ATCM policy documents across all model-derived topics. Bars represent the mean loading score for each topic, calculated separately for scientific (green) and policy (purple) documents. Dots indicate topics that were included in the main analysis.

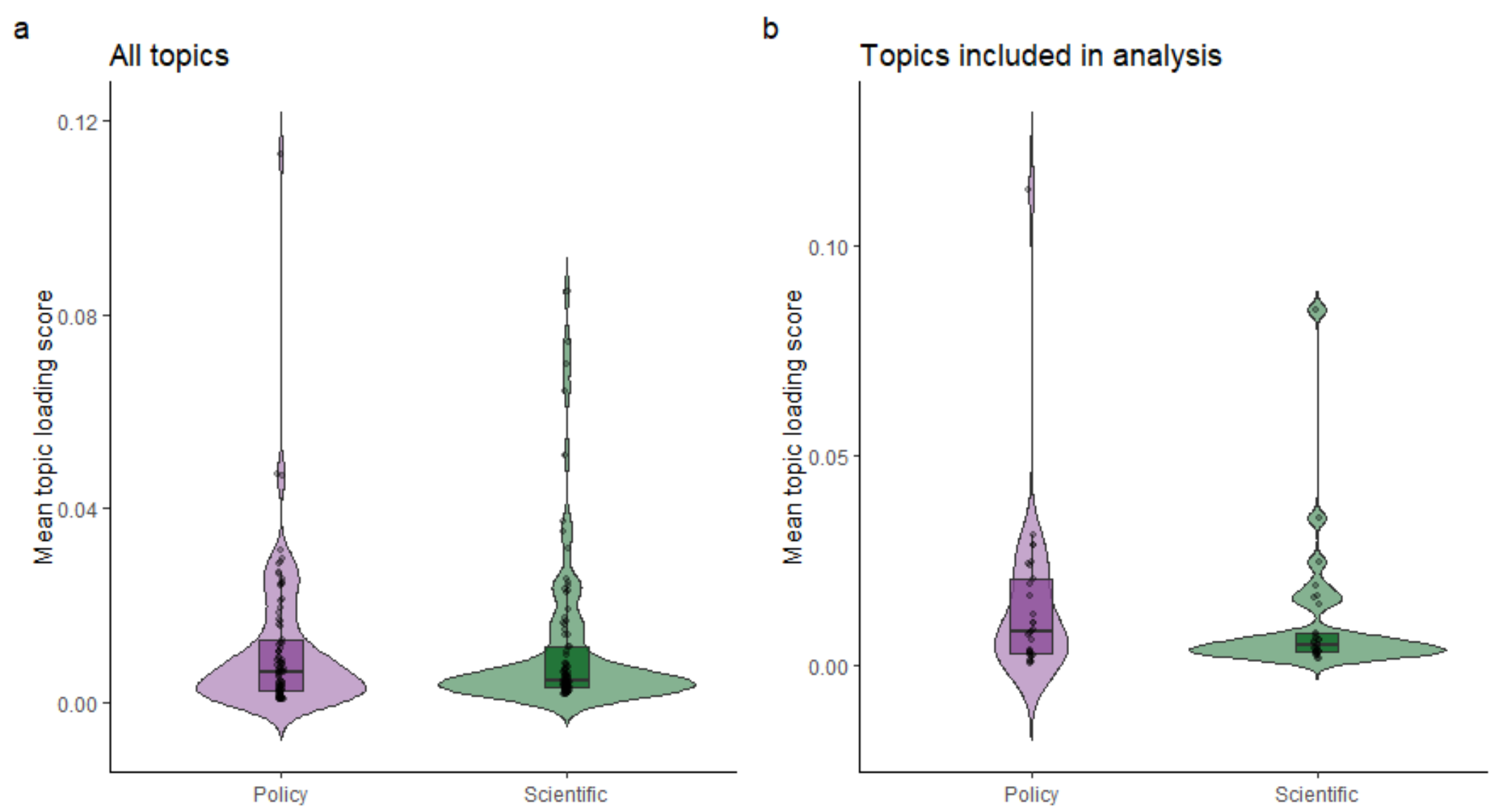

Figure S4.3. Distribution of mean topic loading scores across scientific and policy documents for all topics (a) and for the subset of topics included in the conservation threat framework (b). Each point represents the mean proportions for a single topic, with violin plots showing the overall distribution and embedded boxplots indicating median and interquartile range.

SI 4.3 Accuracy of topic-to-threat classification

SI 4.3.1 Methods

In the main analysis, LDA topics were assigned to conservation threats through structured thematic review and team consensus (see the Methods section of the main text and **Error! Reference source not found.** for complete detail). This process produced a mapping between LDA topics and conservation threat categories, whereby each topic was assigned either to a conservation threat or to a residual category of "no conservation threat". Because subsequent analyses relied on these assignments to infer conservation threats from topic-model outputs, we evaluated their consistency by comparing manually assigned conservation threat classifications with classifications derived from the topic model.

To operationalise this comparison, documents were assigned a model-derived conservation threat classification based on their dominant LDA topic (i.e., the topic with the highest probability). Documents inherited the threat classification associated with their dominant topic, whereas documents whose dominant topic was not assigned to a conservation threat were classified as "no conservation threat". This produced a single model-derived classification for each document. If the topic-to-threat mapping is internally consistent, documents assigned to a particular conservation threat by the topic model should be classified into the same threat category during manual review.

We constructed a manually classified reference dataset by randomly sampling documents from those assigned to each conservation threat based on the model-derived classification, as well as documents classified as "no conservation threat". For each category, we selected 40 documents from both the scientific and policy corpora. Under a binomial sampling framework, this sample size corresponds to an approximate margin of error of ±15 percentage points at the 95% confidence level (see Supplementary Information 4.1.1 for complete details). his level of precision was considered sufficient for evaluating the consistency of the topic-to-threat mapping while remaining feasible for manual review. Where fewer than 40 documents were available for a given category, all available documents were included (**Table S4.4**).

Table S4.4. Validation sample sizes by conservation threat and document type

Conservation threat	Number of sampled documents	
	Scientific	Policy (WP + IP)
Climate change	40	40
Ocean acidification	40	1
Socio-geopolitical disruptions	40	40
Invasive alien species and disease	40	25
Pollution	40	40
Functioning of the Antarctic governance regime	40	40
Mineral and hydrocarbon exploitation	40	40
Bioprospecting	40	19
Human activity impacts	40	40
No conservation threat	40	40

Each document in the validation sample was reviewed and assigned a conservation threat label based on its content, using abstracts for scientific papers and full texts for ATCM policy documents, following the classification criteria outlined in **Table S4.5**. Two reviewers independently classified each document, blinded to the model-derived classifications, with disagreements resolved by a third reviewer acting as an adjudicator, consistent with the procedure used in the relevance validation (SI 4.1.1 Methods). Inter-reviewer agreement between the primary reviewers was assessed using Cohen's κ to evaluate consistency in the application of the classification criteria (Landis & Koch

1977). Agreement was high (κ = 0.91). Disagreements were subsequently reviewed and resolved through discussion between the reviewers, and the consensus labels were treated as the reference standard against which model-derived classifications were evaluated.

To evaluate classification performance, we compared model-derived labels with the manually annotated reference dataset using standard multi-class classification metrics, including precision, recall, and F1-score (Sokolova & Lapalme 2009; Pustejovsky & Stubbs 2013). Precision was calculated as the proportion of correctly classified documents among those assigned to a given category (see SI 4.1.1 Methods for details). Recall was defined as the proportion of documents in the reference dataset assigned to a given category that were correctly identified by the model:

$$Recall = \frac{TP}{TP + FN}$$

where TP is the number of true positives and FN the number of false negatives. Precision therefore quantifies how often conservation threat classifications assigned by the model agreed with the manually annotated reference dataset, whereas recall measures how many documents belonging to a given conservation threat in the reference dataset were successfully identified by the model.

The F1-score, representing the harmonic mean of precision and recall, was used as the primary summary metric of classification performance:

$$F1 = 2 \times \frac{Precision \times Recall}{Precision + Recall}$$

Performance was assessed separately for the scientific and policy corpora, as well as for the combined dataset. Metrics were computed for each conservation threat category and summarised using macro-averaged values (Grandini et al. 2020; Opitz 2024), such that each category contributed equally to the overall performance regardless of the number of documents.

Table S4.5. Classification framework for Antarctic conservation threats.

Conservation threat	Operational definition	Include if…	Exclude if…	Ambiguity rules
Climate change (merged terrestrial + marine)	Anthropogenic climate forcing driving physical and/or ecological change in Antarctic systems	• Analyses atmospheric or ocean warming<br>• Examines sea ice extent, thickness, or variability<br>• Examines glacier, ice shelf, or ice sheet dynamics<br>• Assesses ecological responses explicitly linked to climate change<br>• Uses climate models, projections, or scenarios<br>• Explicitly attributes observed or projected changes to climate change or anthropogenic climate forcing	• Focuses primarily on ocean chemistry changes without broader climate context<br>• Examines direct human activities or local disturbances<br>• Describes environmental variability (seasonal, interannual, or decadal) without explicit attribution to anthropogenic climate forcing<br>• Presents physical or climatological analyses without analysing climate change as a driver or assessing ecological/conservation implications<br>• Mentions climate change or warming only as background context or a secondary explanation	Assign only if climate is a primary driver, not background context; resolve overlap with acidification based on analytical focus; exclude superficial mentions
Ocean acidification	Changes in ocean carbonate chemistry driven by anthropogenic CO_2 uptake, resulting in reduced pH and altered carbonate system dynamics with implications for marine organisms and ecosystems	• Analyses ocean pH, carbonate chemistry, or CO_2 uptake processes<br>• Examines aragonite or calcite saturation states<br>• Investigates biological or ecological responses to changing carbonate chemistry<br>• Uses experiments, observations, or models explicitly focused on acidification processes<br>• Attributes chemical or biological changes to ocean acidification or CO_2-driven shifts in carbonate chemistry<br>• Examines interactions between acidification and marine organisms (e.g. calcifiers, plankton, benthic species)	• Focuses primarily on ocean warming, sea ice, or physical climate processes without analysing carbonate chemistry<br>• Examines climate change broadly without specific treatment of acidification mechanisms<br>• Mentions acidification only as background context or a secondary co-stressor<br>• Analyses marine ecosystem change without explicitly linking outcomes to carbonate chemistry<br>• Describes general ocean biogeochemistry without clear focus on pH or carbonate system dynamics	Assign only if carbonate chemistry change is a primary analytical focus, not a secondary co-stressor; where both warming and acidification are analysed, assign based on the dominant mechanism examined; do not assign if acidification is inferred but not directly analysed or measured; prioritise this category when studies explicitly quantify pH, saturation state, or carbonate chemistry parameters
Socio-geopolitical disruptions	External geopolitical, economic, or societal processes, including strategic competition, legal contestation, or divergence in governance norms, that disrupt or influence Antarctic governance, cooperation, or decision-making, thereby affecting the capacity to manage conservation threats	• Examines geopolitical tensions, strategic competition, or shifts in international relations that influence Antarctic governance<br>• Analyses changes in global cooperation, diplomacy, or multilateral processes affecting ATS decision-making or coordination<br>• Investigates how external crises or socio-economic pressures (e.g. pandemics, economic shocks) impact Antarctic operations or governance<br>• Examines contestation, divergence, or overlap in governance systems (e.g. international law, competing frameworks, or liability and responsibility for environmental harm) that create tension or ambiguity in Antarctic decision-making	• Focuses on internal ATS governance, institutional design, or decision-making without external socio-political drivers<br>• Examines operational, logistical, or environmental processes without a geopolitical or societal mechanism<br>• Describes legal frameworks, cooperation, or governance arrangements without evidence of contestation or influence on decision-making<br>• Mentions geopolitical or global context only as background, without analysing its effect on Antarctic governance or conservation outcomes	Assign only when external socio-political processes actively influence Antarctic governance or decision-making. Do not assign where such processes are absent, implicit, or secondary to internal governance or environmental dynamics. In borderline cases, prioritise whether the primary mechanism of change is external pressure (assign here) or internal system capacity (assign to 'functioning of the Antarctic governance regime').

Invasive alien species and disease	The introduction, establishment, or spread of non-native species and anthropogenically mediated pathogens in the Antarctic region, including diseases introduced or facilitated by human activity, with potential impacts on native ecosystems and biodiversity	• Examines introduction, establishment, or spread of non-native species in Antarctica<br>• Investigates anthropogenically mediated pathogen introduction (e.g. COVID-19, avian influenza)<br>• Analyses biosecurity risks and pathways associated with human activity<br>• Examines mechanisms of spread linked to human movement, transport, or infrastructure<br>• Assesses management, prevention, or mitigation of invasive species or disease risks<br>• Links biological introductions to ecosystem impacts or biodiversity change	• Focuses on naturally occurring diseases or ecological interactions without human-mediated introduction<br>• Examines endemic pathogens or natural disease dynamics in Antarctic species<br>• Discusses climate-driven range shifts without explicit human-mediated introduction pathways<br>• Analyses general biodiversity change without reference to invasive species or introduced disease<br>• Mentions disease or species presence without identifying anthropogenic introduction or spread	Assign only where there is a clear anthropogenic pathway or mechanism; require explicit linkage between disease or species spread and human activity or global movement systems; include wildlife-mediated spread only where linked to human introduction or amplification; do not assign to vague references such as "disease risk" or "biosecurity concern" without a defined mechanism
Pollution	The introduction, transport, or accumulation of anthropogenic contaminants in the Antarctic environment, affecting air, land, water, ice, or biota.	• Examines contamination arising from human activity (e.g. plastics, hydrocarbons, heavy metals, sewage, persistent organic pollutants)<br>• Identifies a contaminant source or pathway, including local release (e.g. stations, waste) or long-range transport<br>• Assesses accumulation or ecological impacts of contaminants (e.g. bioaccumulation, environmental quality)<br>• Evaluates pollution mitigation, waste management, or contamination prevention	• Focuses on climate change or greenhouse gases without a contaminant pathway<br>• Examines natural geochemical processes without anthropogenic input<br>• Describes physical disturbance or human activity without contamination<br>• Focuses on invasive species or disease unless explicitly framed as contamination<br>• Mentions pollution only in general or rhetorical terms	Require identification of a contaminant (or class of contaminants) and an anthropogenic source or pathway; include long-range transport only when contaminants are explicitly specified classify as human activity impacts if focus is infrastructure or human presence without contaminants; classify based on emphasis if focus is governance or regulation (policy vs environmental contamination)
Functioning of the Antarctic governance regime	Processes, mechanisms, and institutional dynamics within the Antarctic Treaty System that maintain, enable, or influence its effective operation, including decision-making, coordination, compliance, and the implementation of environmental governance	• Examines internal ATS decision-making processes (e.g. consensus-based governance)<br>• Analyses coordination, cooperation, or institutional performance within the ATS<br>• Investigates compliance, enforcement, or implementation of ATS agreements<br>• Evaluates the effectiveness, capacity, or functioning of the ATS as a governance system<br>• Discusses institutional structures, procedures, or reforms aimed at maintaining or improving governance processes	• Focuses on external geopolitical dynamics or international relations beyond the ATS<br>• Examines specific environmental threats without discussing governance processes<br>• Discusses policy outcomes or environmental impacts without reference to institutional functioning<br>• Analyses human activities or pressures unless explicitly linked to governance processes<br>• Mentions governance in a general or descriptive way without evaluating its functioning or mechanism	Assign where the primary focus is on how the ATS operates (processes, mechanisms, or performance), rather than the specific issues it addresses; require explicit reference to institutional functioning; do not assign where governance is only background context; where both governance and a specific threat are discussed, classify based on the dominant focus
Mineral and hydrocarbon exploitation	The exploration, prospecting, or potential extraction of mineral and hydrocarbon resources in the Antarctic region, including activities, interests, or pressures	• Examines exploration, prospecting, or extraction of minerals or hydrocarbons in Antarctica<br>• Discusses interest in or potential development of Antarctic mineral or hydrocarbon resources<br>• Analyses technological, economic, or logistical feasibility of mineral or hydrocarbon extraction	• Examines environmental impacts without reference to mineral or hydrocarbon exploration or extraction<br>• Discusses geopolitical dynamics or international relations without explicit linkage to resource exploitation	Assign only where there is an explicit link to exploration, extraction, or utilisation of mineral or hydrocarbon resources; include both realised and hypothetical exploitation (e.g. future scenarios or economic interest); if both governance and exploitation are

	related to their development and use	• Investigates pressures or drivers associated with accessing mineral or hydrocarbon resources<br>• Evaluates risks, implications, or consequences of mineral or hydrocarbon exploitation<br>• Discusses historical or contemporary proposals related to Antarctic mineral or hydrocarbon resource use	• Analyses scientific research on Antarctic geology without connection to resource use or extraction<br>• Focuses on living or renewable resources (e.g. fisheries or biological resources)<br>• Mentions resources in a descriptive or background context only	discussed, assign based on the dominant focus; distinguish from human activities impacts by requiring a clear resource extraction or utilisation component
Bioprospecting	The search for, collection, and utilisation of Antarctic biological material for commercial, industrial, or biotechnological applications, including associated scientific, economic, or strategic interests	• Examines the discovery, collection, or use of Antarctic organisms or genetic material for commercial or industrial purposes<br>• Investigates biotechnological, pharmaceutical, or industrial applications derived from Antarctic biodiversity<br>• Discusses the potential or development of biological resources for commercial use<br>• Analyses economic, technological, or logistical aspects of accessing or utilising Antarctic biological material<br>• Evaluates implications arising from the use of Antarctic biological or genetic resources	• Focuses on basic scientific research without connection to commercial or applied use<br>• Examines ecological, biological, or genetic studies without reference to utilisation or application<br>• Discusses conservation, biodiversity, or ecosystem processes without linkage to resource use<br>• Examines mineral, hydrocarbon, or other non-biological resource exploitation<br>• Mentions biological resources in a descriptive or background context only	Require explicit linkage to utilisation, application, or commercial potential of biological material; do not assign where research is purely descriptive or exploratory without downstream use ; include early-stage or hypothetical applications where intent or potential use is clearly stated; distinguish from human activity impacts by requiring a biological resource use component, not just presence or activity
Human activity impacts	The direct physical impacts of human presence and activities in the Antarctic region, including infrastructure, transport, and operational activities that alter or disturb the environment	• Examines impacts arising from human presence (e.g. research stations, tourism, field operations)<br>• Investigates environmental disturbance from infrastructure development or expansion<br>• Analyses impacts of transport and logistics (e.g. shipping, aviation, overland travel)<br>• Examines direct physical disturbance to habitats, wildlife, or ecosystems (e.g. trampling, noise, site disturbance)<br>• Evaluates management or mitigation of impacts arising from human activities	• The primary mechanism is not direct physical disturbance from human activity<br>• Human activity is mentioned only as background context without assessing environmental impact	Assign only where there is a clear, direct link between human activity and environmental disturbance; if multiple mechanisms are present, assign based on the dominant impact pathway; use this category for direct disturbance not better explained by a more specific mechanism
No conservation threat	Document content that does not substantively engage with any of the defined Antarctic conservation threat categories, even if it concerns Antarctic science, logistics, policy, or environmental systems more broadly	• Analyses physical, biological, or environmental processes without linking them to conservation impacts or threats<br>• Examines variability or system dynamics without attributing change to a defined conservation threat<br>• Focuses on methods, models, monitoring, or data collection without interpreting results in a conservation context	• The document explicitly links processes or activities to environmental, ecological, or institutional impacts<br>• A defined conservation threat is a primary analytical focus, not background context<br>• The document frames findings in terms of risk, impact, vulnerability, or management relevance<br>• Human activities, environmental change, or governance are analysed in relation to their consequences for Antarctic systems	Assign to no conservation threat when the document contributes scientific, operational, or policy context without explicitly analysing a defined conservation threat. Prioritise the primary analytical focus over incidental mentions, and do not infer conservation relevance unless it is clearly established

SI 4.3.2 Interpretation

At the aggregate level, classification performance demonstrated good agreement between model-derived and manually assigned labels, with macro-averaged precision, recall, and F1-scores of 82.1%, 86.4%, and 83.3%, respectively (**Table S4.6**). Performance was broadly consistent across corpora, with scientific documents showing marginally higher precision (84.7%) and recall (88.3%) than policy documents (83.0% and 85.6%, respectively), resulting in a slightly higher F1-score for the scientific subset (85.3% vs 83.2%). Across all subsets, recall consistently exceeded precision, indicating that the model is more likely to identify documents containing conservation threats than to miss them, albeit at the cost of some over-classification of documents that do not substantively engage with these threats.

Table S4.6. Summary performance metrics for topic-to-threat classification across document types.

Sampled document type	Precision	Recall	F1-score
Overall	82.1	86.4	83.3
Scientific	84.7	88.3	85.3
Policy	83.0	85.6	83.2

Across categories, classification performance varied, with F1-scores ranging from 64% to 92% (**Table S4.7**; **Figure S4.4**). Several threats were classified with relatively high performance, including invasive alien species and disease (F1 = 90%), bioprospecting (92%), mineral and hydrocarbon exploitation (87%), and pollution (88%). By contrast, lower performance was observed for the functioning of the Antarctic governance regime (F1 = 78%), human activity impacts (76%), and the 'no conservation threat' category (64%). The observed variation in F1-scores across conservation threats reflects how distinctly each threat is represented within the topic structure. Threats characterised by well-separated thematic signals were more reliably captured by LDA topics, whereas those with broader or overlapping representations were more difficult to delineate. The mechanisms underlying these differences, including the distinct forms of misclassification observed across threat categories, are examined in detail below.

Table S4.7. Per-category classification performance for topic-to-threat mapping across the validation dataset

Conservation threat	True positive	False positive	False negative	Precision	Recall	F1
Climate change	71<br>[39, 32]	9<br>[1, 8]	5<br>[3, 2]	89 (80-94)[a]<br>[98, 80][b]	93 (86-97)<br>[93, 94]	91<br>[95, 86]
Ocean acidification	28<br>[27, 1]	13<br>[13, 0]	0<br>[0 0]	68 (53-80)<br>[68, 100]	100 (88-100)<br>[100, 100]	81 [81, 100]
Socio-geopolitical disruptions	64<br>[35, 29]	16<br>[5, 11]	3<br>[2, 1]	80 (70-87)<br>[88, 72]	96 (88-98)<br>[95, 97]	87<br>[91, 83]
Invasive alien species & disease	57<br>[33, 24]	8<br>[7, 1]	5<br>[2, 3]	88 (78-94)<br>[82, 96]	92 (82-97)<br>[94, 89]	90<br>[88, 92]
Pollution	67<br>[34, 33]	13<br>[6, 7]	5<br>[3, 2]	84 (74-90)<br>[85, 82]	93 (85-97)<br>[92, 94]	88<br>[88, 88]
Functioning of the Antarctic governance regime	62<br>[28, 34]	18<br>[12, 6]	18<br>[2, 16]	78 (67-85)<br>[70, 85]	78 (67-85)<br>[93, 68]	78<br>[80, 76]
Mineral and hydrocarbon exploitation	63<br>[37, 26]	17<br>[3, 14]	2<br>[2, 0]	79 (69-86)<br>[92, 65]	97 (89-99)<br>[95, 100]	87<br>[94, 79]
Bioprospecting	55<br>[37, 18]	4<br>[3, 1]	5<br>[4, 1]	93 (84-97)<br>[92, 95]	92 (82-96)<br>[90, 95]	92<br>[91, 95]
Human activity impacts	66<br>[32, 34]	14<br>[8, 6]	27<br>[8, 19]	82 (73-89)<br>[80, 85]	71 (61-79)<br>[80, 64]	76<br>[80, 73]
No conservation threat	65<br>[37, 28]	15<br>[3, 12]	57<br>[35, 22]	81 (71-88)<br>[92, 70]	53 (44-62)<br>[51, 56]	64<br>[66, 62]

[a] Values in parentheses correspond to 95% confidence intervals for performance metrics calculated across the combined validation dataset. Confidence intervals are shown only for combined precision and recall values.

[b] Values in brackets correspond to metrics for the scientific corpus (first value) and policy corpus (second value).

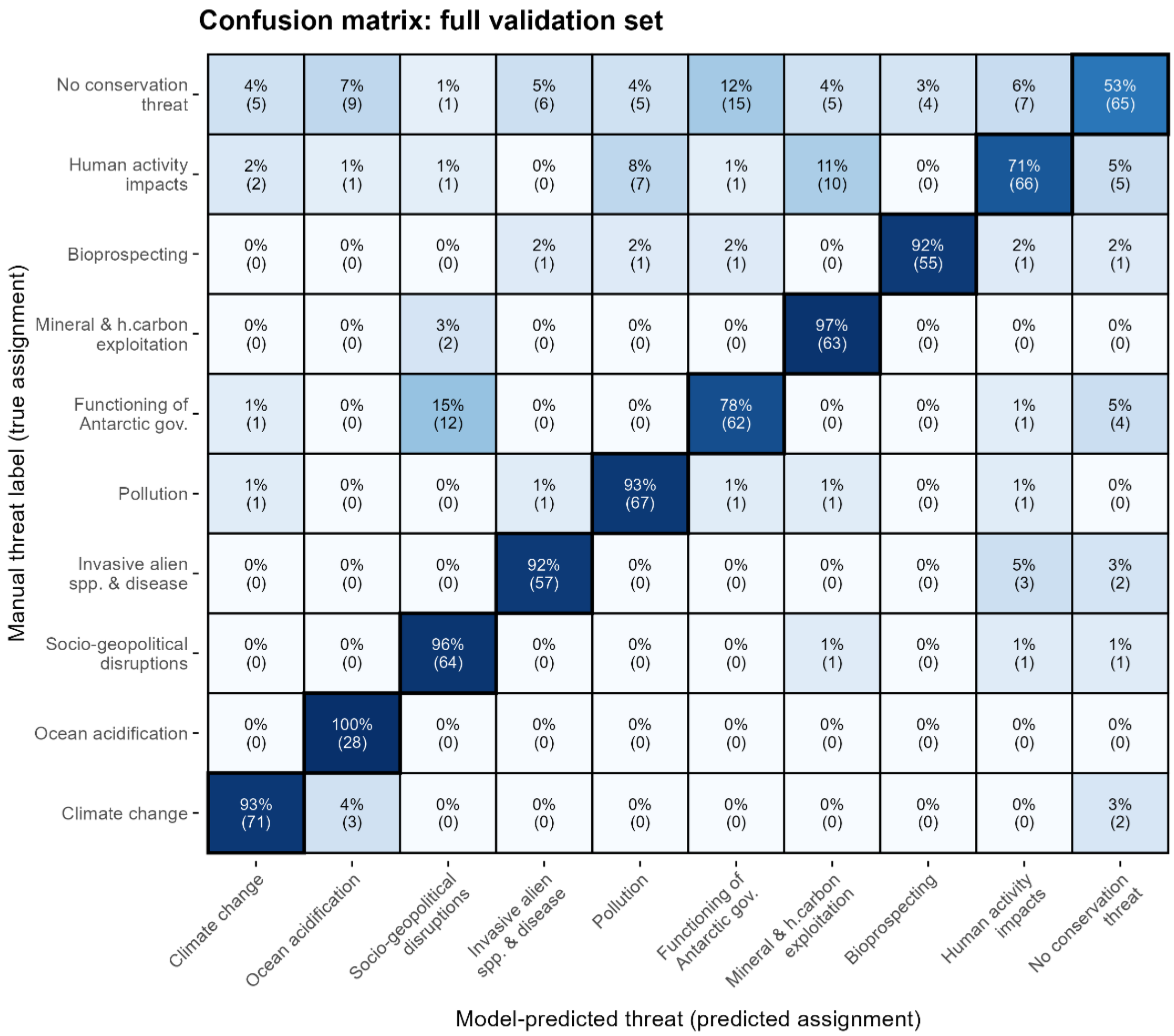

Figure S4.4. Row-normalised confusion matrix comparing model-derived and manually assigned conservation threat labels for the full validation dataset.

Misclassification arose from two primary sources: reassignment among conceptually related conservation threats and over-assignment of conservation threats to documents that did not substantively engage with them. Errors arising from reassignment among closely related threats were concentrated in a small number of categories. The most pronounced case was the functioning of the Antarctic governance regime, where 15% of manually classified documents were instead assigned to socio-geopolitical disruptions (n = 12), a pattern driven primarily by the policy corpus (20%; n = 10, compared with 7%; n = 2 in the scientific corpus) (**Figure S4.5**). This reflects overlap between procedural or institutional discussions and broader geopolitical themes. A similar, though more distributed, pattern was observed for human activity impacts, where documents were reassigned to mineral and hydrocarbon exploitation (11%; n = 10) and pollution (8%; n = 7), with misclassification again more pronounced in the policy corpus (Figure S4.5). In these cases, the content does describe human activities but is more strongly defined by specific pressures, leading it to be more consistently assigned to narrower threat categories.

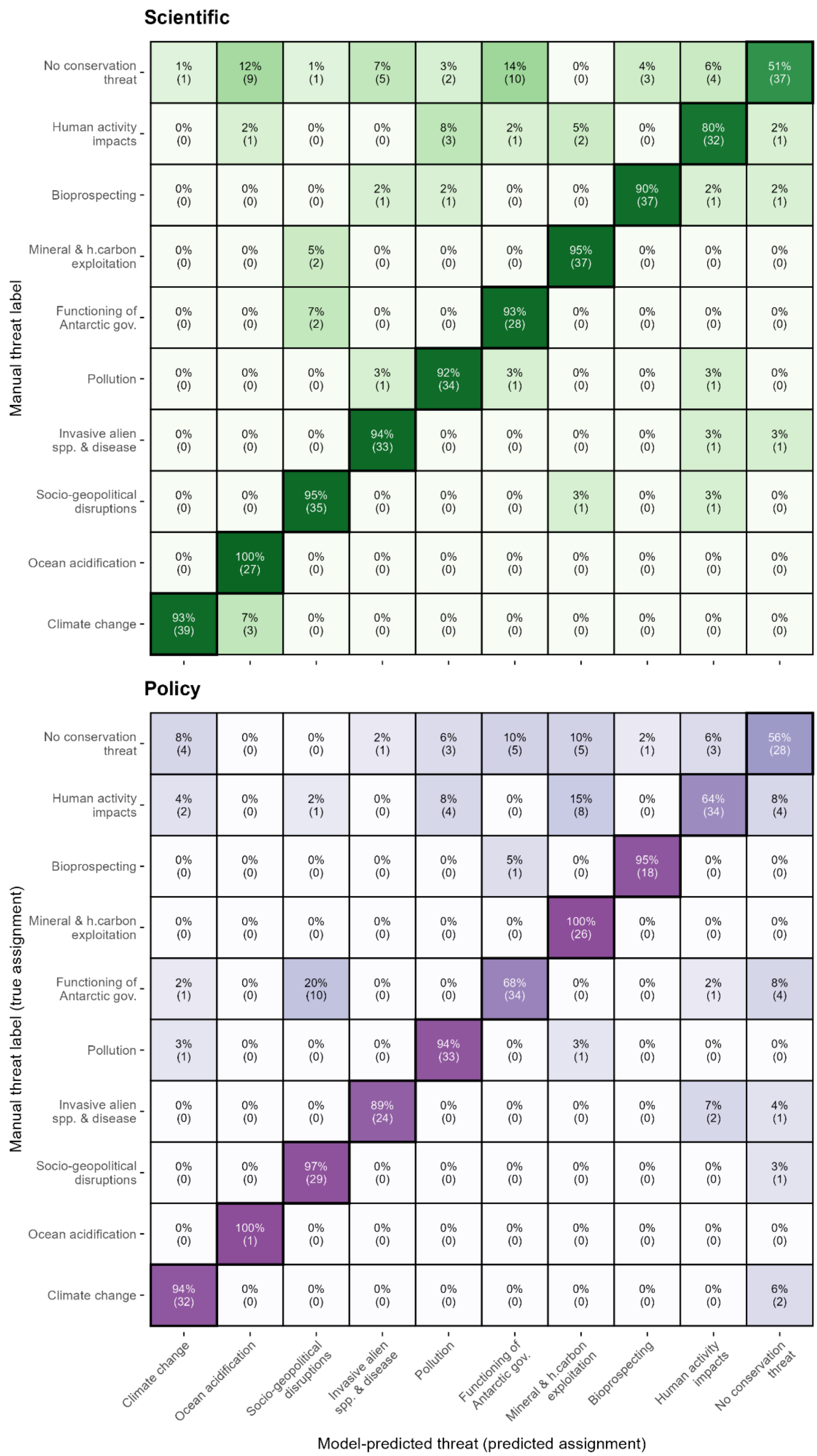

Figure S4.5. Row-normalised confusion matrices showing agreement between model-derived and manually assigned conservation threat labels for scientific and policy corpora.

Over-classification primarily arose from documents being assigned to conservation threat categories rather than classified as ‘no conservation threat’. Accordingly, the ‘no conservation threat’ category exhibited relatively high precision (81%) but substantially lower recall (53%), indicating that while assignments to this category were generally reliable, many documents without a clearly defined conservation threat were nevertheless reassigned to a conservation threat category. These misclassifications were distributed across multiple conservation threats and typically represented only a small proportion of documents assigned to each category. However, reassignment of ‘no conservation threat’ documents was more pronounced for ocean acidification (22%; 9 of 41 assigned documents), the functioning of the Antarctic governance regime (19%; 15 of 80 assigned documents), and, to a lesser extent, bioprospecting (7%; 4 of 59 assigned documents). These patterns reflect the presence of related thematic content that does not explicitly constitute a conservation threat.

Ocean acidification provides a clear example of this over-assignment pattern, with perfect recall (100%) but comparatively lower precision (68%). This indicates that all manually identified ocean acidification documents were successfully captured, but that the model also assigned additional documents to this category. These misclassifications appear to arise from overlap with broader ocean biogeochemistry, where closely related processes share similar thematic signals. As a result, the model captures a broader biogeochemical signal associated with ocean acidification, encompassing both acidification-specific studies and more general ocean biogeochemical dynamics.

Importantly, the validation framework used here differs from the probabilistic framework used in the primary analysis by forcing each document into a single dominant conservation threat category. In the primary analysis, however, documents retain their full probabilistic topic distributions and may contribute simultaneously to multiple conservation threats. This hard classification framework may therefore inflate apparent misclassification where documents contain overlapping or weaker thematic signals. To evaluate this possibility, we examined documents that were manually classified as ‘no conservation threat’ but were instead assigned by the model to a conservation threat category. We then assessed whether ‘no conservation threat’ remained the second-strongest model-derived signal within those documents.

Among the 57 documents manually classified as ‘no conservation threat’ but reassigned by the dominant-topic validation framework to a conservation threat category, 47 (82.5%) ranked ‘no conservation threat’ as the second-strongest model-derived signal. This suggests that many apparent false positives arise from the stringent nature of the hard classification framework rather than complete semantic misclassification. The probabilistic model assigns documents mixtures of thematic signals with varying strengths, but the validation framework forces each document into a single dominant category. Consequently, some documents were classified as errors even when substantial non-threat thematic structure remained strongly represented within the model output.

Interpreting these metrics requires consideration of the structure of the classification task. Documents are assigned to partially overlapping conservation threats based on probabilistic topic representations, so some degree of ambiguity is unavoidable (Pustejovsky & Stubbs 2013). There is also no universal threshold for acceptable performance in multi-class classification, and single summary metrics can mask important aspects of model behaviour (Grandini et al. 2020; Opitz 2024). These metrics therefore evaluate the consistency of the dominant-topic operationalisation used for validation, rather than the full probabilistic framework used in the primary corpus-level analyses. In this context, classification performance was strong and internally consistent, with macro-averaged precision, recall, and F1-scores of 82.1%, 86.4%, and 83.3%, respectively, and similar values across the scientific and policy corpora. With ten conservation threat categories, this level of agreement is substantially above chance expectations and indicates that the manually derived topic-to-threat alignment captures meaningful thematic structure across the corpus.

Over-assignment of conservation threats nevertheless introduces some inflation of individual categories, particularly where weak or diffuse thematic signals overlap with broader conservation topics. The additional diagnostic analyses suggest that many apparent false positives arise from the stringent hard-classification framework imposed during validation rather than complete semantic misclassification, particularly for documents manually classified as ‘no conservation threat’. Although it is not possible to determine directly whether this inflation was distributed uniformly through time, the major temporal patterns identified in the policy corpus correspond closely with well-documented periods of ATS negotiation, implementation, and institutional change (Figures 2–3, Main Text). This correspondence suggests that the dominant temporal dynamics primarily reflect underlying institutional processes rather than artefacts of classification. Nevertheless, conservation threats exhibiting lower precision, particularly ocean acidification, should be interpreted more cautiously than higher-performing categories.

SI 4.4 Sensitivity analysis

Natural language processing models are not uniquely determined by the underlying data, but depend on a range of modelling decisions, including preprocessing and parameter specification (Denny & Spirling 2018). To assess whether the patterns identified by our LDA model reflect meaningful structure in the corpus, rather than artefacts of these choices, we conducted two complementary sensitivity analyses. First, we investigated how alternative term-frequency thresholds for excluding rare and highly frequent terms altered the composition of the model vocabulary and the resulting topic structure. Second, we evaluated the sensitivity of the model to parameter specification by varying key hyperparameters and refitting the model under each configuration while holding the vocabulary constant. We conducted these analyses separately because changing term-frequency thresholds alters the vocabulary underlying the model, resulting in topic structures that are not directly comparable across configurations (Schofield et al. 2017). We therefore first defined a stable and interpretable vocabulary before evaluating sensitivity to model specification within this fixed feature space.

SI 4.4.1 Sensitivity to vocabulary construction

SI 4.4.1.1 Methods

To construct the model vocabulary, we applied document-frequency thresholds to exclude terms that were either too rare or too common across the corpus. Terms occurring in very few documents were removed to reduce the influence of sparse or potentially spurious tokens, whereas highly ubiquitous terms were excluded because they provide limited discriminative information for topic inference (see **Error! Reference source not found.** for implementation details). To evaluate sensitivity to vocabulary trimming, we systematically varied the proportion of rare and highly frequent terms excluded from the corpus and assessed the resulting changes in inferred topic structure. Because the effects of vocabulary trimming on topic structure may depend partly on model parameterisation (Bystrov et al. 2023), all vocabulary configurations were evaluated across a set of plausible LDA hyperparameter values rather than under a single fixed specification. This allowed us to assess whether the inferred topic structure remained stable across alternative vocabulary definitions and identify a balanced threshold configuration.

We evaluated a series of vocabulary configurations in which the minimum and maximum document-frequency thresholds were varied symmetrically. Specifically, we tested paired document-frequency thresholds of 5–95%, 10–90%, 15–85%, and 20–80%, corresponding to progressively more restrictive definitions of the model vocabulary (Schofield et al. 2017). For each configuration, the corpus was re-vectorised and the LDA model was refitted from scratch so that the inferred topic structure reflected the modified vocabulary. Each vocabulary specification was then evaluated across five hyperparameter configurations of the symmetric Dirichlet priors on document–topic (α) and topic–word (β) distributions: $\alpha = 0.1$, $\beta = 0.005$; $\alpha = 0.05$, $\beta = 0.005$; $\alpha = 0.1$, $\beta = 0.001$; $\alpha = 0.1$, $\beta = 0.01$; and $\alpha = 0.2$, $\beta = 0.005$. The purpose of varying hyperparameters within this subsection was not to optimise model specification for each vocabulary configuration, but to assess whether sensitivity to vocabulary trimming remained consistent across a reasonable range of modelling assumptions.

To evaluate how vocabulary trimming influenced the interpretability of inferred topics, we quantified topic coherence across all vocabulary configurations. Topic coherence was evaluated using Normalised Pointwise Mutual Information (NPMI), a co-occurrence-based metric that measures the semantic relatedness of high-probability words within topics, with higher values indicating more interpretable and internally coherent topic structure (Röder et al. 2015). NPMI is widely used in topic modelling because it corresponds more closely with human judgements of topic interpretability than unnormalised PMI-based measures (see **Error! Reference source not found.** for complete details) (Röder et al. 2015). For each fitted model, we calculated NPMI using the top 20 words per topic and summarised each model using the median NPMI across topics, consistent with the number of words

used for manual topic interpretation and assignment to conservation threats. Median NPMI values were then compared across vocabulary configurations to assess the trade-off between improved topic coherence and increasingly restrictive vocabulary trimming.

Selecting document-frequency thresholds in topic modelling involves balancing improved coherence against retention of semantically meaningful vocabulary, and higher coherence can partly reflect the removal of terms that increase topic heterogeneity (Bystrov et al. 2023). We therefore did not select the final vocabulary configuration solely by maximising NPMI. Instead, we complemented coherence-based evaluation with a quantitative inspection of the excluded vocabulary. Specifically, we classified removed terms as either dictionary-recognised words or non-dictionary tokens (e.g. typographical variants and non-standard terms) and quantified how the number and rate of removal of each category varied across document-frequency thresholds. This allowed us to assess whether increasingly restrictive thresholds disproportionately removed semantically meaningful terms relative to noise, potentially leading to the loss of less frequent or more specialised topics.

SI 4.4.1.2 Results

The distribution of excluded tokens was strongly skewed towards low term-frequency thresholds (**Figure S4.6**). At a 1% threshold, approximately 150,000 non-dictionary tokens (i.e. non-recognised words and typographical variants) were removed compared to ~5,000 dictionary words, indicating that early trimming primarily removes low-frequency, non-standard terms. As the threshold increased, the number of excluded tokens declined rapidly, with non-dictionary tokens decreasing by more than an order of magnitude between 1% and 5% (~150,000 to ~5,000), and to fewer than ~1,500 by 10%, while dictionary words declined more gradually (~5,000 to ~900 to ~450 over the same interval) (**Figure S4.6**).

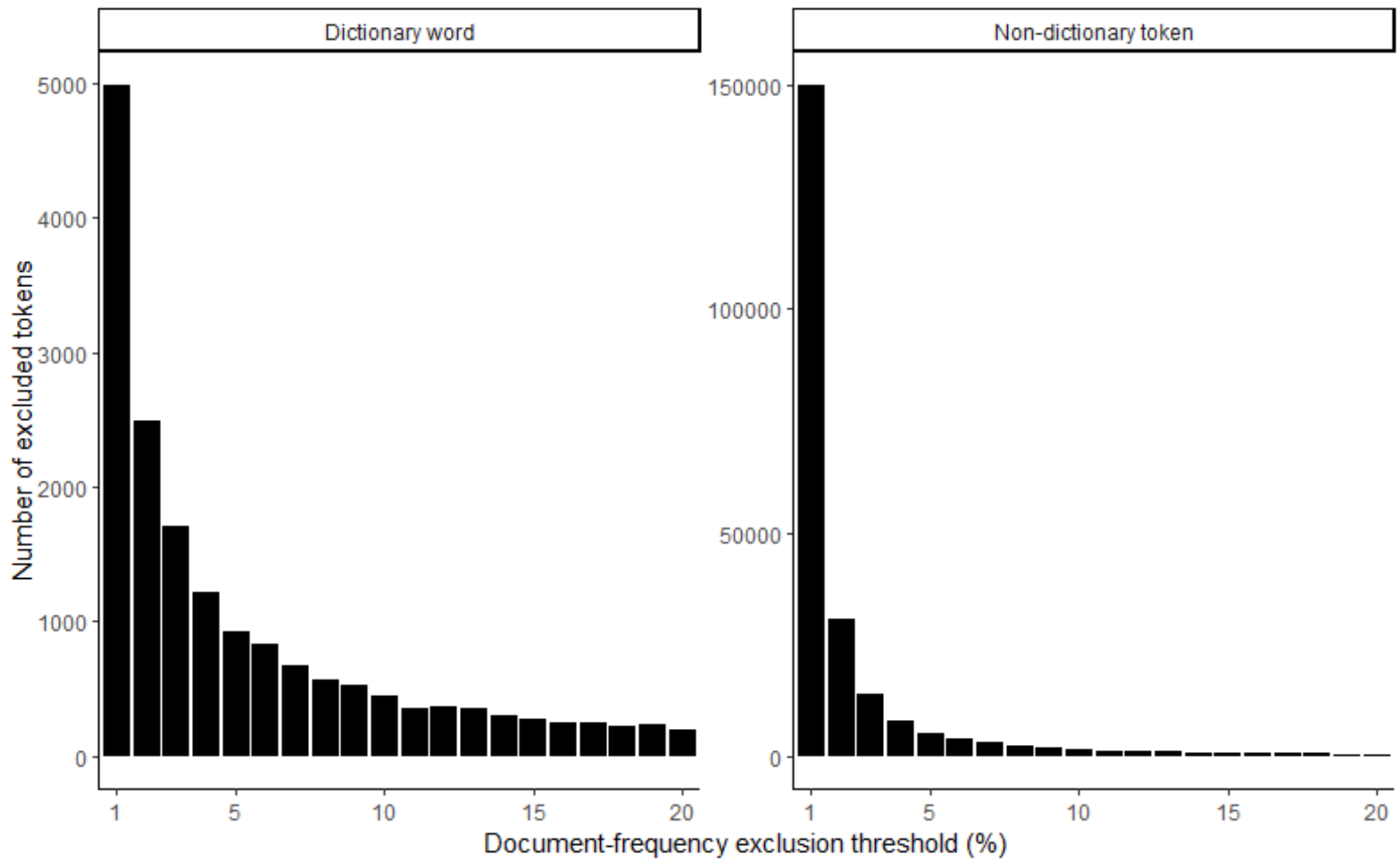

Figure S4.6. Number of excluded dictionary words (left) and non-dictionary tokens (right) across document-frequency thresholds.

The magnitude of these reductions decreased across successive thresholds. For non-dictionary tokens, percentage reductions fell from 96.6% between 1% and 5% to 72.3% between 5% and 10%, and to 54.0% and 40.6% across the 10–15% and 15–20% intervals, respectively. Dictionary words followed

a similar but less pronounced pattern (81.5%, 51.5%, 40.0%, and 29.4%; **Table S4.8**), indicating that reductions in non-dictionary tokens diminish more rapidly with increasing threshold, whereas the removal of dictionary words continues at a comparatively steady rate.

Table S4.8. Changes in excluded non-dictionary tokens and dictionary words across document-frequency thresholds

Interval	Non-dictionary tokens removed (start → end)	Δ (%)	Dictionary words removed (start → end)	Δ (%)
1 → 5%	150,103 → 5,090	-96.6	4,986 → 923	-81.5
5 → 10%	5,090 → 1,413	-72.3	923 → 448	-51.5
10 → 15%	1,413 → 650	-54.0	448 → 269	-40.0
15 → 20%	650 → 386	-40.6	269 → 190	-29.4

To assess how these changes in vocabulary composition affect model performance, we evaluated topic coherence across alternative trimming thresholds. Topic coherence increased consistently with more restrictive vocabulary definitions (**Figure S4.7**), with the lowest values observed under 5–95% thresholds (median NPMI = 0.112–0.139) and progressively higher values under 10–90% (0.133–0.150), 15–85% (0.159–0.179), and 20–80% configurations (0.184–0.195). The largest increase in coherence occurred between the 10–90% and 15–85% thresholds and was consistent across all parameterisations, distinguishing lower-coherence configurations (5–95% and 10–90%) from higher-coherence configurations (15–85% and 20–80%). We therefore excluded the 5–95% and 10–90% configurations from further consideration.

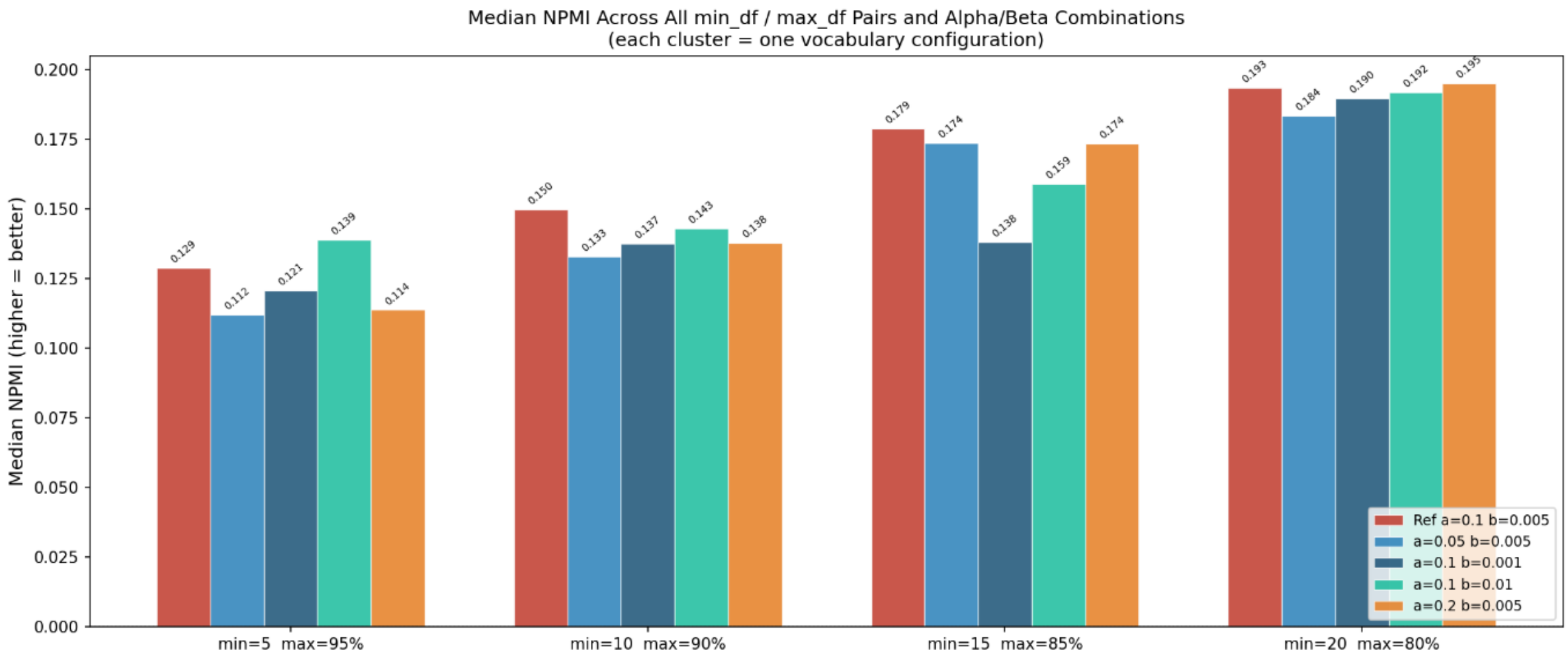

Figure S4.7. Median topic coherence (NPMI) across vocabulary configurations and hyperparameter settings. Bars show median NPMI values for models fitted using minimum–maximum document-frequency thresholds of 5–95%, 10–90%, 15–85%, and 20–80%. Colours denote different α–β parameterisations.

Comparison of the remaining thresholds showed that although the 20–80% configuration produced the highest median NPMI values. At the same time, analyses of excluded vocabulary showed that the proportion of excluded terms recognised as dictionary words exceeded 30% from thresholds ≥17% (**Figure S4.8**), while additional reductions in non-dictionary tokens were limited (**Figure S4.6**; **Table S4.8**). Because the analysis aims to capture nuanced conservation-related concepts, retaining interpretable vocabulary is essential. We therefore selected the 15–85% threshold as a balanced configuration that improves topic coherence while avoiding unnecessary loss of meaningful terms.

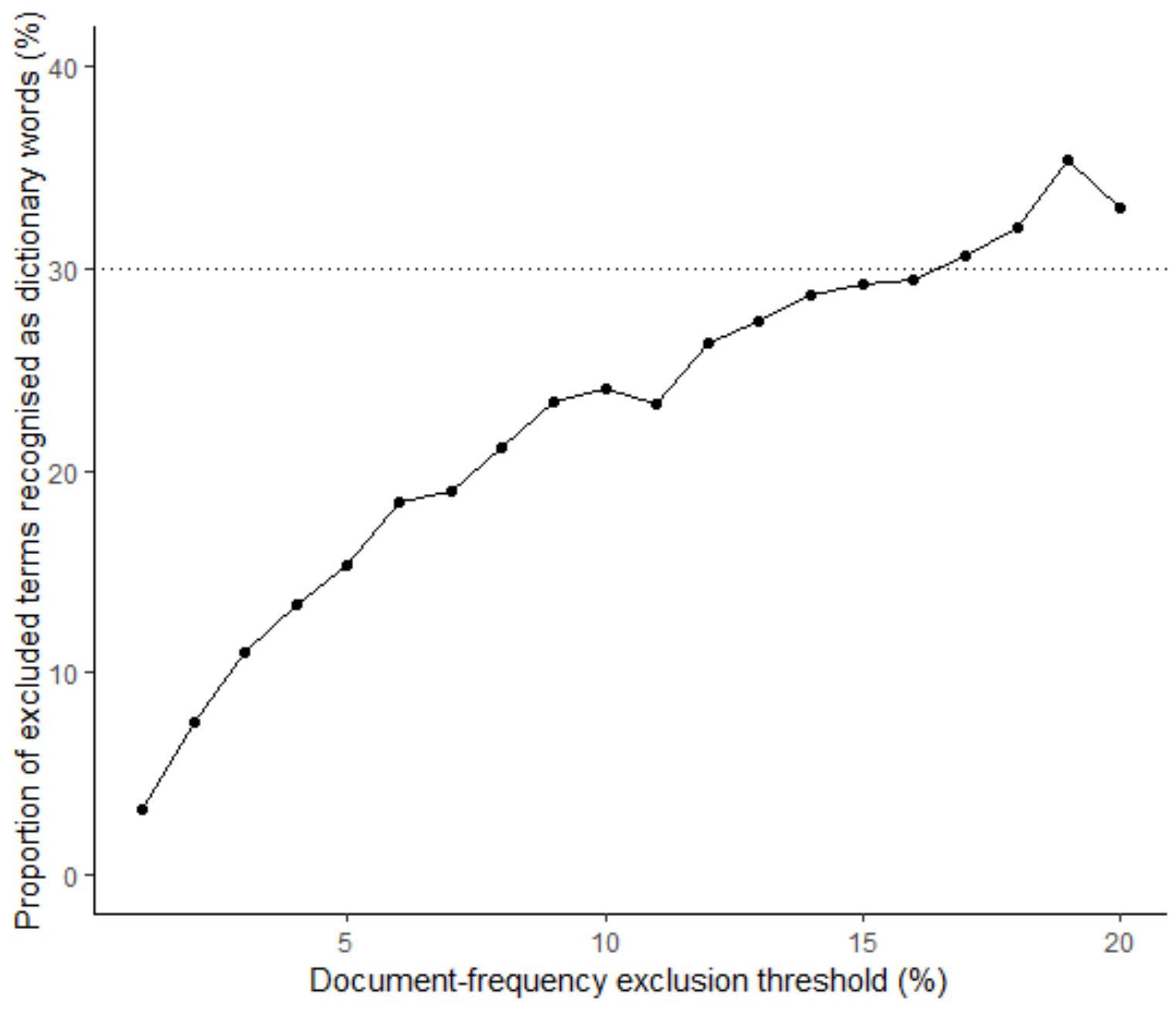

Figure S4.8. Proportion of excluded terms recognised as dictionary words across document-frequency thresholds. The dotted line indicates 30%.

SI 4.4.2 Sensitivity to model specification

SI 4.4.2.1 Methods

Following selection of the 15–85% document-frequency thresholds, we evaluated the sensitivity of the topic modelling framework to model specification. We varied the Dirichlet priors governing document–topic (α) and topic–word (β) distributions while holding the vocabulary specification and number of topics (K = 90) constant, the latter having previously been identified as the optimal topic resolution for the corpus (see **Error! Reference source not found.** for complete details). This allowed differences among model outputs to be attributed specifically to alternative α–β parameterisations rather than changes to the underlying representation or granularity of the corpus.

We evaluated four alternative α–β parameterisations alongside the reference model used in the primary analysis (**Table S4.9**). These parameterisations were selected to induce varying levels of sparsity and diffusivity in document–topic and topic–word distributions, spanning topic structures from highly specialised representations to more diffuse thematic mixtures.

Table S4.9. α–β parameterisations used in the sensitivity analysis of model specification.

Model	α	β	Expected effect
Reference	0.1	0.005	Baseline sparse representation, producing relatively specialised documents and topics
01-0001	0.1	0.001	More concentrated topic-word distributions, with topics defined by smaller sets of highly specialised words
01-001	0.1	0.01	More diffuse topic-word distributions, with broader and more overlapping vocabularies across topics
005-0001	0.05	0.001	More concentrated document-topic mixtures, with documents associated with fewer dominant topics
02-0005	0.2	0.005	More diffuse document-topic mixtures, with documents distributed across larger numbers of topics

After model fitting, topics were reassigned to conservation threats using the same structured review framework applied in the primary analysis (see **Error! Reference source not found.** for complete details). Briefly, this process involved reviewing representative documents and topic-word distributions to determine whether topics aligned with the predefined conservation threat categories. Because the objective of this analysis was to assess the robustness of downstream temporal inference to model specification, rather than independently validate each parameterisation, we did not repeat the full topic-to-threat classification accuracy assessment (outlined in SI 4.3.1 Methods). Following topic assignment, we repeated the downstream analytical workflow for each model configuration by fitting cubic splines to the temporal trajectories of each conservation threat and calculating periods of significant increase and decline using the same derivative-based framework applied in the primary analysis.

Robustness was assessed by comparing the temporal dynamics inferred under each α–β parameterisation with those of the reference model for each conservation threat. We first evaluated whether major historically interpretable periods of change identified in the primary analysis were consistently reproduced across parameterisations. For each model configuration, we quantified the number of directional reversals ("flips") between significant periods of increase and decline identified from the spline derivatives for each conservation threat. This approach is consistent with the primary analysis, which focused on broad temporal dynamics and historically interpretable periods of directional change rather than exact year-by-year correspondence. We then calculated the absolute deviation in the number of reversals relative to the reference model for each conservation threat. Exact temporal alignment of spline trajectories and fitted magnitudes was not expected across parameterisations because topic modelling is a stochastic and non-identifiable process in which alternative parameter settings can yield different but similarly plausible decompositions of semantic structure. Consequently, robustness was evaluated based on whether alternative model specifications reproduced similar overall frequencies and relative patterns of directional change rather than identical year-by-year trajectories. Differences in the number of directional reversals between sensitivity models and the reference model were assessed separately for scientific and policy corpora using paired Wilcoxon signed-rank tests. We expected parameterisations closer to the reference model to reproduce more similar temporal dynamics than more strongly perturbed configurations.

SI 4.4.2.2 Interpretation

Broad temporal dynamics were consistently reproduced across alternative α–β parameterisations, with both fitted spline trajectories (**Figure S4.9**) and periods of significant directional change (**Figure S4.10** and **Figure S4.11**) remaining qualitatively similar across models. Major historically interpretable periods of change identified in the primary analysis were likewise preserved across parameterisations. More strongly perturbed parameterisations produced modest shifts in topic structure, including broader thematic distributions in diffuse models and narrower representations in more concentrated models, resulting in some variation in spline magnitudes and the exact timing of directional transitions. However, these differences did not substantially alter the broader temporal narratives inferred from the corpus because comparable thematic content could still be reconstructed across parameterisations by adjusting the number of topics assigned to individual conservation threats. Consequently, the principal conservation dynamics identified in the primary analysis remained robust to reasonable perturbations of model specification.

Importantly, major historically interpretable periods of change identified in the primary analysis were likewise preserved across parameterisations within the policy corpus. For example, discussions related to mineral and hydrocarbon exploitation consistently increased through the 1970s and early 1980s before declining following negotiations surrounding the Convention on the Regulation of Antarctic Mineral Resource Activities (CRAMRA). Similarly, policy discussions concerning pollution increased throughout the 1980s and remained elevated until the implementation of the Protocol on

Environmental Protection to the Antarctic Treaty in the late 1990s. Discussions surrounding the functioning of the Antarctic Treaty System also showed consistent increases through the 1980s as additional Parties joined the Treaty and the governance framework expanded in complexity. Despite variation in topic structure among parameterisations, these broader historical dynamics remained qualitatively stable across models.

Ocean acidification represented the least stable conservation threat across parameterisations. Only two models (01_001 and 01_0001; **Figure S4.9**) recovered a distinct ocean acidification signal and, even in these cases, the inferred temporal dynamics differed from those identified in the primary analysis. This instability likely reflects the comparatively subtle and overlapping semantic structure of ocean acidification within the corpus, where discussions of acidification are frequently embedded within broader themes related to climate change, marine systems, and oceanographic processes. Consequently, the topic was often subsumed within neighbouring thematic structures rather than emerging as a consistently distinct conservation threat across parameterisations.

Quantitative comparisons of directional reversals likewise indicated strong agreement between sensitivity models and the reference configuration. In most cases, alternative parameterisations reproduced the same number of significant directional reversals ("flips") identified in the primary analysis, with at least half of all threat–corpus comparisons showing no deviation from the reference model. Even where differences occurred, they were generally small, with sensitivity models differing from the reference configuration by an average of only 0.24 flips and never by more than two flips. Consistent with this interpretation, paired Wilcoxon signed-rank tests identified no significant differences in the number of directional reversals between sensitivity models and the reference model when scientific and policy corpora were analysed together ($p = 0.43$) or separately (scientific: $p = 0.89$; policy: $p = 0.45$). These results indicate that perturbations to model priors produced only minor differences in directional change and did not substantially alter the broader temporal dynamics inferred from the corpus.

Overall, these results indicate that the principal temporal dynamics identified in the primary analysis are robust to reasonable perturbations of model specification, particularly at the scale of broad conservation narratives and historically interpretable periods of change. At the same time, the sensitivity analyses demonstrate that individual spline trajectories can vary among parameterisations because alternative topic models partition semantic structure differently across topics. Consequently, while broad directional patterns were consistently reproduced, the exact magnitudes of spline responses and the precise timing of directional transitions should not be interpreted as fixed temporal estimates. Rather, the modelling framework is most informative for identifying persistent large-scale patterns of change and the relative prominence of conservation threats within the scientific and policy corpora. Ocean acidification represented the primary exception to this broader pattern of robustness and should therefore be interpreted more cautiously than the remaining conservation threats. The lack of consistent temporal dynamics across parameterisations prevents strong temporal inference for this threat.

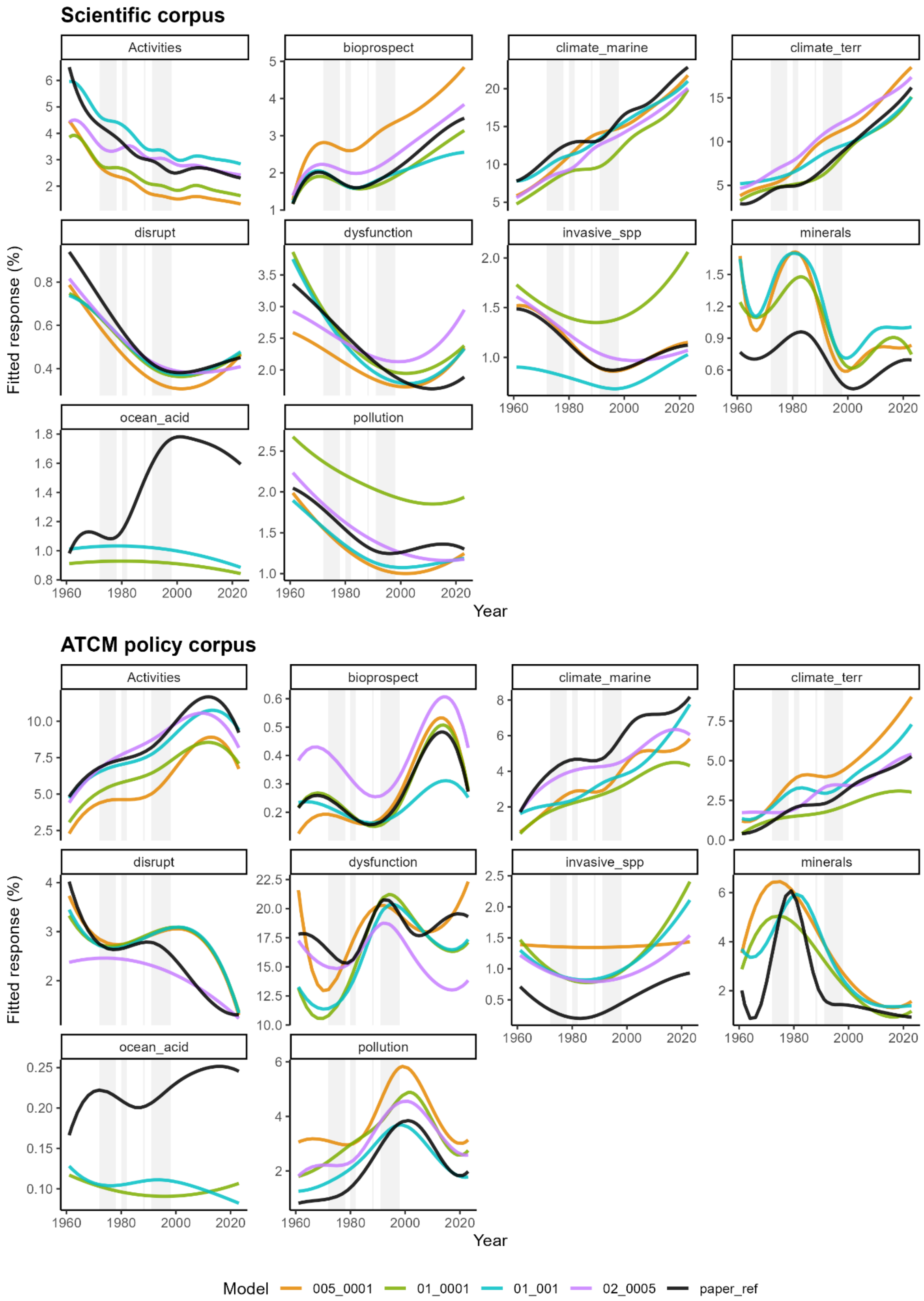

Figure S4.9. Sensitivity of fitted spline trajectories to alternative α–β parameterisations of the topic model. Fitted response curves are shown for each conservation threat across the scientific and ATCM policy corpora under the reference model and four perturbed parameterisations. Shaded regions indicate major historically relevant periods in Antarctic governance.

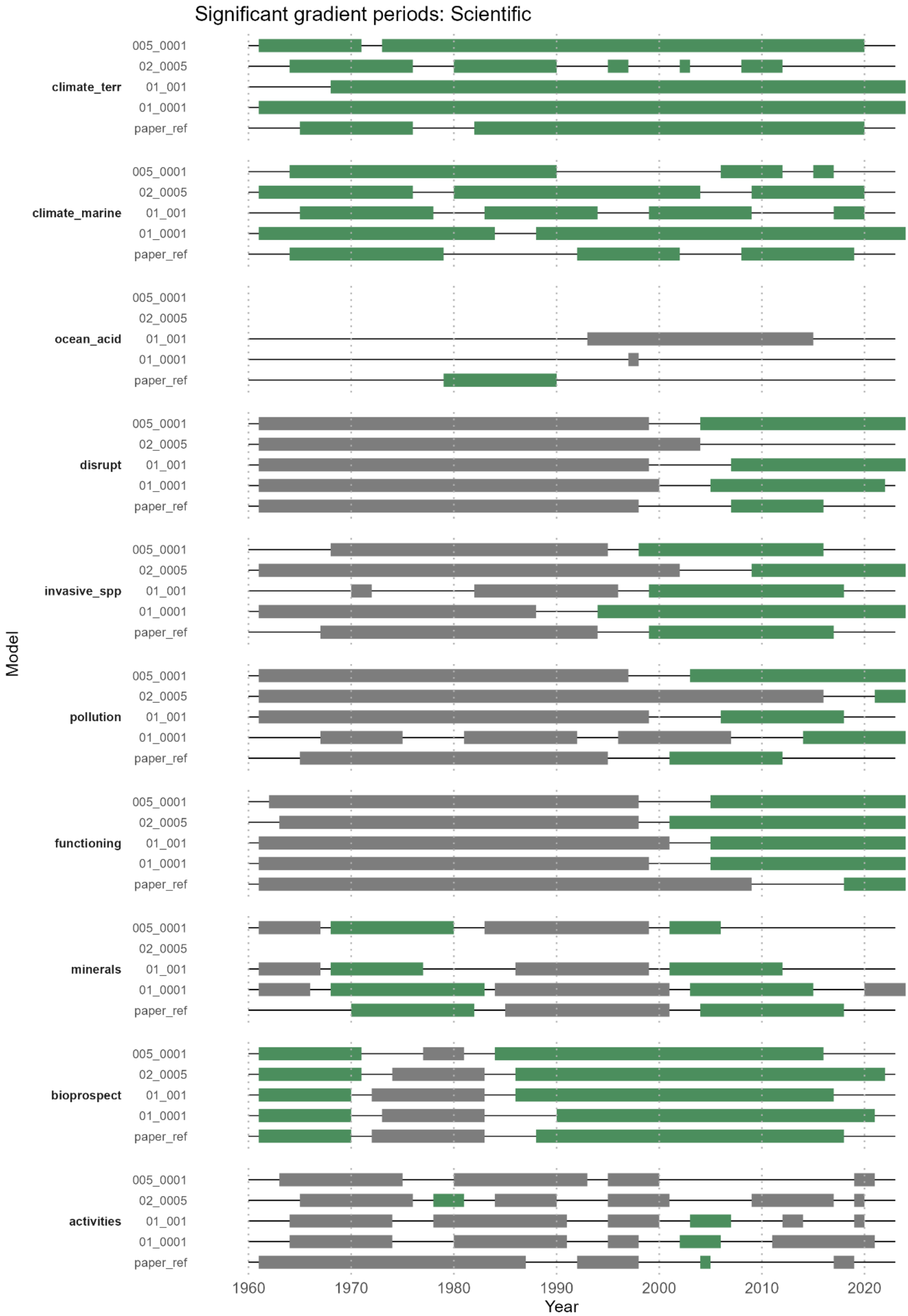

Figure S4.10. Significant periods of directional change identified from spline derivatives for the scientific corpus across alternative α–β parameterisations. Green segments indicate statistically significant increases in fitted response through time, whereas grey segments indicate significant declines.

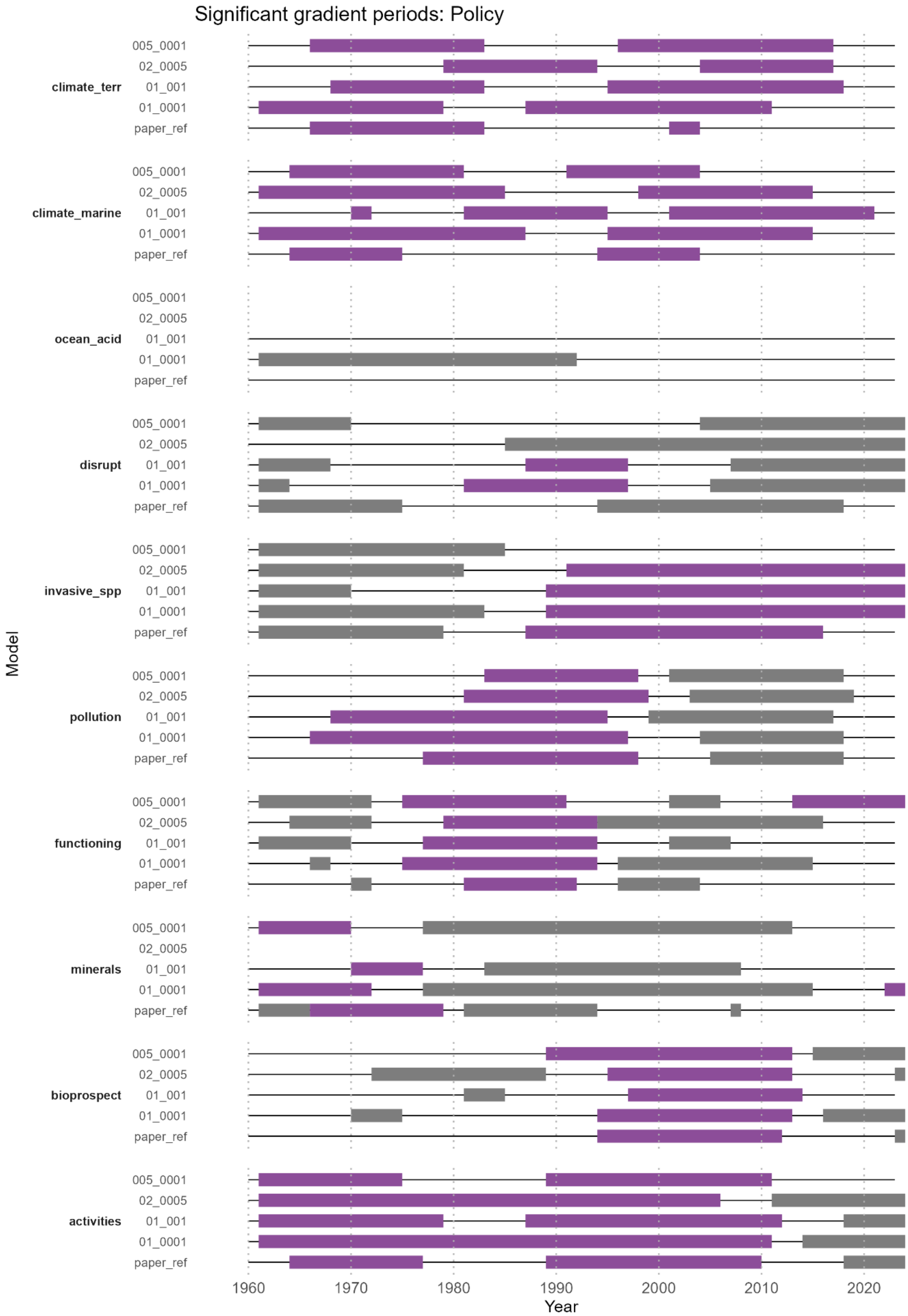

Figure S4.11. Significant periods of directional change identified from spline derivatives for the scientific corpus across alternative α–β parameterisations. Green segments indicate statistically significant increases in fitted response through time, whereas grey segments indicate significant declines.

Update 2 – Revised conceptual framing

In response to reviewer comments, we substantially revised the conceptual framing of the manuscript. These changes affected the Introduction, Methods, Discussion, and Conclusions. Selected excerpts are provided below to illustrate the nature and scope of these revisions; however, they do not represent all changes made to the manuscript.

Revised Introduction excerpt (aims and objectives)

Despite the ATS's structured pathway for conservation policy, its capacity to achieve substantive outcomes has long been contested[13,14]. Across its more than 60-year history, the regime has been both lauded as an exemplar of environmental governance in a shared international space[15,16] yet also criticised as a system in paralysis, no longer capable of delivering substantive outputs[17-20]. Resolving this debate has become increasingly important as conservation threats facing the Antarctic region continue to diversify and intensify[21,22]. These threats differ markedly in their origin, scale of potential impact, and underlying drivers, ultimately placing different demands on the ATS[1,23-25]. This complicates efforts to assess or improve the regime's performance[26,27]: apparent underperformance may reflect system-wide institutional failure, weaknesses in specific contexts, or the limits of what the regime can reasonably be expected to achieve[16,28,29]. Clarifying this position is critical to the Antarctic region's preservation[30], as it defines whether future efforts to strengthen conservation should focus on internal reform, strengthening coordination with external governance frameworks, or maintaining existing approaches[20].

To address this issue, we quantitatively examine how the ATS engages with conservation threats through its deliberative and decision-making processes. We focus specifically on the regime's capacity to recognise, deliberate on, and develop responses to conservation threats. Accordingly, our analysis centres on these processes rather than the regime's institutional architecture or policy outputs alone. Institutional architecture reveals the authorities, obligations, and procedures available to the ATS, but not how these mechanisms are deployed in practice. Likewise, policy outputs provide evidence of governance responses but reveal comparatively little about the deliberative processes through which those outputs emerged. Although both of these perspectives illuminate the regime's design and performance[20], they provide only partial insight into the deliberative processes through which conservation threats are considered and addressed[31]. This distinction is critical because major policy interventions often result from prolonged periods of negotiation and deliberation that are not apparent from the outputs alone[32,33]. We therefore examine the dynamics of these deliberative processes alongside the ATS's institutional architecture and policy outputs to provide a more holistic assessment of the regime's capacity to address conservation threats.

The ATS provides a rare opportunity to investigate these deliberative processes because agenda-setting, policy deliberation, and decision-making are formally structured around documents submitted to the ATCM[34]. These submissions create a documentary record of how conservation threats enter and move through the regime's deliberative processes. Because environmental decisions are meant to be guided by the best available scientific and technical advice[12,35], understanding these processes also requires consideration of the scientific

discourse from which that advice is drawn. We therefore analyse more than 90,000 English-language scientific publications and ATCM policy documents that have been published since the ATS came into force (1961—2023, inclusive; hereafter the 'corpus').

Revised Discussion excerpt (opening and interpretation of findings)

Despite the ATS's structured pathway for conservation policy, its capacity to achieve substantive outcomes has long been contested[13,14]. Across its more than 60-year history, the regime has been both lauded as an exemplar of environmental governance in a shared international space[15,16] yet also criticised as a system in paralysis, no longer capable of delivering substantive outputs[17-20]. Resolving this debate has become increasingly important as conservation threats facing the Antarctic region continue to diversify and intensify[21,22]. These threats differ markedly in their origin, scale of potential impact, and underlying drivers, ultimately placing different demands on the ATS[1,23-25]. This complicates efforts to assess or improve the regime's performance[26,27]: apparent underperformance may reflect system-wide institutional failure, weaknesses in specific contexts, or the limits of what the regime can reasonably be expected to achieve[16,28,29]. Clarifying this position is critical to the Antarctic region's preservation[30], as it defines whether future efforts to strengthen conservation should focus on internal reform, strengthening coordination with external governance frameworks, or maintaining existing approaches[20].

To address this issue, we quantitatively examine how the ATS engages with conservation threats through its deliberative and decision-making processes. We focus specifically on the regime's capacity to recognise, deliberate on, and develop responses to conservation threats. Accordingly, our analysis centres on these processes rather than the regime's institutional architecture or policy outputs alone. Institutional architecture reveals the authorities, obligations, and procedures available to the ATS, but not how these mechanisms are deployed in practice. Likewise, policy outputs provide evidence of governance responses but reveal comparatively little about the deliberative processes through which those outputs emerged. Although both of these perspectives illuminate the regime's design and performance[20], they provide only partial insight into the deliberative processes through which conservation threats are considered and addressed[31]. This distinction is critical because major policy interventions often result from prolonged periods of negotiation and deliberation that are not apparent from the outputs alone[32,33]. We therefore examine the dynamics of these deliberative processes alongside the ATS's institutional architecture and policy outputs to provide a more holistic assessment of the regime's capacity to address conservation threats.

The ATS provides a rare opportunity to investigate these deliberative processes because agenda-setting, policy deliberation, and decision-making are formally structured around documents submitted to the ATCM[34]. These submissions create a documentary record of how conservation threats enter and move through the regime's deliberative processes. Because environmental decisions are meant to be guided by the best available scientific and technical advice[12,35], understanding these processes also requires consideration of the scientific discourse from which that advice is drawn. We therefore analyse more than 90,000 English-

language scientific publications and ATCM policy documents that have been published since the ATS came into force (1961—2023, inclusive; hereafter the 'corpus').

Update 3 – Expanded discussion of study limitations

The modelling framework developed here is most informative at the scale of broad conservation narratives and historically interpretable periods of change, rather than fine-grained temporal estimates. Sensitivity analyses showed that changing model parameters affected the estimated magnitude of individual spline curves but had little influence on their overall shape or the broader temporal patterns they revealed. Accordingly, the framework is best suited to identifying persistent large-scale patterns of change and the relative prominence of conservation threats within scientific and policy discourse, rather than exact temporal estimates. Further details of the validation analyses are provided in the Methods and Supplementary Information 4.

While LDA enables holistic analysis of large corpora[57], topics do not always correspond cleanly to individual conservation threats, particularly where threats share common terminology. We addressed this through conservative topic assignment criteria, independent review, and validation analyses, which supported the overall robustness of the threat classifications. Nevertheless, some overlap between threat categories is unavoidable (see Supplementary Information 4 for complete details). Additionally, LDA captures thematic prominence rather than the framing or sentiment of discussion. Consequently, changes in engagement reveal how prominently a conservation threat is discussed, but not whether those discussions are supportive, critical, or otherwise framed. Distinguishing between descriptions of the ATS's functioning versus dysfunction, for example, was not possible (see Supplementary Information 1 for details). Finally, our reliance on scientific abstracts and ATCM Working Papers and Information Papers limits access to the full nuance of debates and excludes informal or undocumented exchanges[9,81]. These sources nevertheless provide the most consistent and reproducible record of ATCM discourse.

Our analysis excludes CCAMLR documents, as CCAMLR constitutes a distinct decision-making body with different actors, institutional structures, and policy priorities[9]. This choice likely underestimates policy engagement with some marine-related conservation threats. Nevertheless, because the Protocol applies across the Antarctic Treaty Area, including marine environments south of 60°S, many Southern Ocean conservation threats are discussed across multiple ATS decision-making bodies rather than exclusively through CCAMLR[28]. Accordingly, our conclusions relate most directly to the Treaty and Protocol, while still providing insights into broader conservation dynamics across the ATS.

Despite its limitations, our application of LDA to Working Papers and Information Papers offers a systematic and reproducible way to analyse ATCM deliberations, providing new insights into how conservation priorities are negotiated within the ATS. While the Antarctic Treaty Secretariat maintains a comprehensive archive of official Meeting documents, it does not include transcripts, meaning previous assessments of negotiation dynamics have been

largely limited to policy outputs rather than the deliberative record itself; e.g., see [82,83]. Here, we show that Working Papers and Information Papers provide a reliable proxy for the substance of deliberations, with shifts in attention aligning closely with the negotiation and implementation of major environmental agreements. This approach therefore extends the study of Antarctic governance by revealing aspects of ATCM deliberations that would otherwise remain obscured.

Update 4 – Revised conclusions and recommendations

Our analysis suggests that a central challenge facing the ATS in addressing conservation threats is not an absence of capacity, but how that capacity is exercised across the jurisdictional boundaries that define the regime. For threats originating within the Antarctic Treaty Area, recurring cycles of deliberation, governance development, and declining engagement provide evidence that the regime can translate sustained deliberation into governance action. External threats, however, present a different picture. Despite posing some of the greatest risks to Antarctic ecosystems[22], the patterns of engagement provide comparatively little evidence that the capacity available within the ATS is being fully exercised. Although the regime's institutional architecture provides pathways for engaging with such threats, these mechanisms have not been fully leveraged[74], resulting in either prolonged deliberation or limited engagement. This finding is consistent with broader conclusions that many of Antarctica's most significant conservation threats cannot be resolved in isolation[74,77]. For threats originating beyond the Antarctic Treaty Area, effective responses may necessarily depend on coordination across governance systems. Realising the full potential of the ATS will therefore require more deliberate links with global governance frameworks responsible for climate and ocean systems[64]. The regime already contains provisions for such coordination, and future progress may depend on how effectively these mechanisms are operationalised across governance systems.

Specific strategies for strengthening coordination across governance regimes have been explored elsewhere[84-86]. The most immediate opportunities lie in reinforcing engagement with the broader Antarctic regime complex[74,77]. This refers to the web of overlapping international institutions that influence the Antarctic region, including UN frameworks such as the UN Framework Convention on Climate Change (UNFCCC) and the UN Convention on the Law of the Sea (UNCLOS)[64,87] (although see [73] for a worthwhile critique). These linkages can be operationalised by positioning the ATS as a regional node within a wider governance network, where global rules are translated into Antarctic-specific measures and Antarctic policies inform global decision-making (see Supplementary Information 2 for additional considerations regarding the CCAMLR Convention). Operationalising these linkages requires acknowledging institutional overlap and deliberately managing it, ensuring that the ATS helps shape, rather than passively absorb, emerging global norms.
