# Supplementary material for: The limits of Antarctic conservation governance
Source: Nat Commun. 2026 Oct 6;17:11398. doi: 10.1038/s41467-026-77606-w (PMC13736897; doi:10.1038/s41467-026-77606-w)
Supplement: Supplementary file 4 [file 41467_2026_77606_MOESM1_ESM.docx]

**Supplementary Information**

**The limits of Antarctic conservation governance**

Zachary T. Carter^1,2,3,*^, Larissa Lubiana Botelho^1,2^, Steven L. Chown^4^, Parsa Yaryab^2^, Nadiah P. Kristensen^1,2^, Kerrie A. Wilson^5^, Daniela Liggett^6^, Michael Bode^1,2^

1. School of Mathematical Sciences, Queensland University of Technology, Gardens Point, Brisbane, QLD 4001, Australia
2. Securing Antarctica’s Environmental Future, School of Mathematical Sciences, Queensland University of Technology, Gardens Point, Brisbane, QLD 4001, Australia
3. Securing Antarctica’s Environmental Future, School of Biological Sciences, The University of Adelaide, North Terrace, Adelaide, SA 5005, Australia
4. Securing Antarctica’s Environmental Future, School of Biological Sciences, Monash University, Melbourne, Victoria 3800, Australia
5. Securing Antarctica’s Environmental Future, School of Biology and Environmental Science, Queensland University of Technology, Gardens Point, Brisbane, QLD 4001, Australia
6. School of Earth and Environment, University of Canterbury, Christchurch, 8041, New Zealand

*Corresponding author: [zach.carter@qut.edu.au](mailto:zach.carter@qut.edu.au)

Table of Contents

[Supplementary Figures	3](#_Toc238569824)

[Supplementary Tables	4](#_Toc238569825)

[Supplementary Notes	5](#_Toc238569826)

[Supplementary Note 1. Extended Materials and Methods	5](#_Toc238569827)

[Note 1.1. Identification and definition of conservation threats	5](#_Toc238569828)

[Note 1.2. Topic modelling	9](#_Toc238569829)

[Supplementary Note 2. Extended Discussion.	18](#_Toc238569830)

[Note 2.1. Spline fit oscillations and main Antarctic Treaty System Agreements	18](#_Toc238569831)

[Note 2.2. CCAMLR, science-based governance, and regime interactions.	22](#_Toc238569832)

[Supplementary Note 3. Aligning topics with conservation threats.	23](#_Toc238569833)

[Note 3.1. Topic selection for External Threats.	24](#_Toc238569834)

[Note 3.2. Topic selection for Transboundary Threats.	30](#_Toc238569835)

[Note 3.3. Topic selection for Internal Threats.	34](#_Toc238569836)

[Supplementary Note 4. Validation of the topic modelling pipeline.	44](#_Toc238569837)

[Note 4.1. Corpus retrieval validation.	45](#_Toc238569838)

[Note 4.2. Topic composition analysis.	48](#_Toc238569839)

[Note 4.3. Accuracy of topic-to-threat classification.	50](#_Toc238569840)

[Note 4.4. Sensitivity analysis.	62](#_Toc238569841)

[Supplementary References	73](#_Toc238569842)

# Supplementary Figures

**Supplementary Figure 1.** Simplified decision-making structure of the ATS. Scientific and technical advice enters the system, primarily through WPs and IPs submitted to the ATCM. Many proposals are first reviewed by the CEP, though the ATCM may also deliberate directly on conservation matters. Outcomes are adopted by consensus and may result in binding or non-binding instruments. Coloured boxes illustrate the core assumptions of our analysis regarding how scientific topics of varying priority are translated into ATCM discussions and potential policy outcomes. SCAR = Scientific Committee on Antarctic Research; ASOC = Antarctic and Southern Ocean Coalition. See Methods for complete details.

# Supplementary Tables

**Supplementary Table 1.** Summary of statistical tests used to evaluate differences in the number of spline gradient category shifts across conservation threat categories (‘External,’ ‘Transboundary,’ and ‘Internal’).

|  | Statistical Test | Description | Outcome | | Interpretation |
| --- | --- | --- | --- | --- | --- |
|  |  |  | Scientific Literature | Policy Documents |  |
| 1 | Kruskal-Wallis test^a^ with bootstrapped confidence intervals (n = 1000 iterations) | Non-parametric analysis of variance (ANOVA) to test for differences between conservation threat categories (External, Transboundary, Internal) and the number of spline gradient category shifts (from positive to negative and vice versa) | H(2) = 7.88, *p* = .019  [95CI = 0.011, 0.024] | H(2) = 6.54, *p* = .037  [95CI = 0.02, 0.042] | Significant differences were observed for both the scientific literature and policy documents (*p*<0.05) indicating that the number of spline gradient category shifts differs among conservation threat categories. |
| 2 | Dunn test | Post hoc pairwise comparison to identify which conservation threat categories differ in the number of gradient category shifts. | Internal-External:  *z* = 2.81, *p* = 0.005, *p*_adj_ = 0.015  Internal-Transboundary:  *z* = 1.20, *p* = 0.229, *p*_adj_ = 0.687  External-Transboundary:  *z* = -1.5, *p* = .133, *p*_adj_ = 0.400 | Internal-External:  *z* = 2.65, *p* = 0.010, *p*_adj_ = 0.032  Internal-Transboundary:  *z* = 1.31, *p* = 0.189, *p*_adj_ = 0.568  External-Transboundary:  *z* = -0.825, *p* = 0.248, *p*_adj_ = 0.744 | Internal threats had significantly more spline gradient category shifts compared to External threats for both the scientific literature and policy documents. Other pairwise comparisons were not statistically significant. |
| 3 | Jonckheere-Terpstra test | Post hoc test to assess whether the number of gradient category shifts follows an ordinal trend across conservation threat categories (where Internal > Transboundary > External) | *J* = 1.5, *p* = .004 | *J* = 3.5, *p* = .012 | A significant trend was detected for both the scientific literature and policy documents (*p*<0.05), indicating that the number of gradient category shifts increases in an ordinal manner, with greater shifts observed as the focus transitions from External to Transboundary to Internal threats. |

# Supplementary Notes

## Supplementary Note 1. Extended Materials and Methods

### Note 1.1. Identification and definition of conservation threats

The conservation threats examined in this study were identified through systematic horizon scanning ^1,2^, using large, diverse expert groups to elicit, refine, and prioritise key challenges. This process generated a broad and representative inventory of threats to Antarctic conservation and formed the foundation for our study sensu ^3^. In total, approximately 22 distinct threats were identified and grouped into six thematic categories based on their primary drivers (**Supplementary Table 2**; see ^2^ for details on the derivation of these categories).

**Supplementary Table 2** Conservation threats facing the Antarctic region, identified through expert horizon scanning and grouped into thematic categories based on primary drivers.

| **Direct climate/environmental change** | |
| --- | --- |
| 1 | Ocean acidification |
| 2 | Climate change impacts on marine systems |
| 3 | Climate change impacts on terrestrial systems |
| 4 | Extreme precipitation events |
| **Indirect climate/environmental change** | |
| 5 | Invasive alien species |
| 6 | Geoengineering |
| 7 | Emerging animal pathogens |
| **Effectiveness of the Antarctic governance regime** | |
| 8 | Regulatory failure |
| 9 | Collapse of the Antarctic Treaty System |
| 10 | Militarisation of the Antarctic region |
| 11 | Changes in environmental protection policies |
| 12 | Reduced cooperation among Antarctic Treaty Parties |
| **Global Disruptions** | |
| 13 | War among Antarctic Treaty Parties |
| 14 | Security threats and illicit activities |
| 15 | Large-scale epidemic or pandemic of the human population |
| **Resource exploitation and economic interests** | |
| 16 | Mineral extraction and hydrocarbon exploration |
| 17 | Bioprospecting |
| 18 | Iceberg harvesting |
| **Human footprint and activity expansion** | |
| 19 | Habitat alteration and activity impacts |
| 20 | Pollution |
| 21 | Permanent human settlement |
| 22 | Agricultural expansion |

Here, we adopt the definition of a ‘conservation threat’ proposed by ^2^: any political, legal, economic, social, technological, or environmental factor, activity, or process—whether local or global—that may negatively affect, either directly or indirectly, (1) any aspect of the Antarctic natural environment and/or (2) the planning, implementation, or management of conservation actions relating to it.

To ensure that the final list of conservation threats could be meaningfully analysed using topic modelling, we employed a two-step filtering process (**Supplementary Figure 2**). This process was necessary to (1) minimise artificial separation of conceptually similar threats and (2) ensure sufficient representation in the policy corpus to enable robust analysis. First, we consolidated related threats that were likely to be grouped under the same topic using Latent Dirichlet Allocation (LDA) topic modelling. Given that LDA is based on a bag-of-words approach, which captures term frequency without accounting for word order or semantic nuance, conceptually similar threats with overlapping vocabularies would be difficult to distinguish ^4^. We therefore merged these threats to avoid artificial separation during topic assignment. Second, we excluded any remaining threats that were not represented in the Antarctic Treaty Consultative Meeting (ATCM) documents—the primary venue through which conservation policy is developed, debated, and implemented under the Antarctic Treaty System (ATS) ^5^. Threats with little or no presence in this corpus were excluded, as their absence precluded a meaningful assessment of how the ATS has engaged with them over time. While a lack of policy discussion may itself be interpreted as a signal of institutional avoidance or inaction, the excluded threats were largely hypothetical or highly emergent, making it difficult to robustly identify their presence in the policy corpus or interpret trends with confidence.

To illustrate the two-stage filtering process, we use extreme precipitation events as an example of a consolidated threat (stage 1) and geoengineering as an example of an excluded threat (stage 2). Extreme precipitation events were consolidated with broader climate change impacts on terrestrial systems due to their overlapping drivers and vocabulary. While they represent a more precise ecological concern—particularly in relation to biodiversity impacts ^6,7^ and atmospheric river dynamics ^8^—they are conceptually and semantically embedded within the wider discourse on climate change. Merging these threats helped prevent artificial separation during topic modelling. On the other hand, geoengineering has generated considerable recent debate within the scientific community due to its uncertain effectiveness, ecological risks, and geopolitical and ethical implications e.g., see ^9,10^. However, it was formally raised for the first time at the 46th ATCM in 2024 ^11^ and, as such, provides little basis for assessing whether the ATS is equipped to address the threat overall.

Following this filtering process, we identified ten conservation threats to carry forward into our analysis (**Supplementary Table 3**). To support systematic interpretation, we organised these threats using the Driver–Pressure–State–Impact–Response (DPSIR) framework ^12^. This structure provided a conceptual basis to capture the breadth of ecological, social, and governance dimensions associated with each threat. It also allowed us to clearly categorise the component topics detected through our LDA topic modelling (see **Supplementary Note 1.2**, below, for complete details).

**Supplementary Figure 2**. Flow diagram illustrating the filtering process used to refine the initial list of conservation threats into the final set used for analysis.

.

|  | Conservation Threat | Management scope | Environmental drivers | System-level changes | Ecological and conservation impacts | References |
| --- | --- | --- | --- | --- | --- | --- |
| 1 | Climate change impacts on terrestrial systems | Extrinsic | Anthropogenic GHG emissions | Loss of ice mass and localised ice melt  Increased air temperatures  Soil drying and reduced water availability  Changes in vegetation dynamics | Stress and loss of native terrestrial biodiversity  Poleward shifts in terrestrial biodiversity  Altered ecosystem structure and habitat suitability | ^7,13-15^ |
| 2 | Climate change impacts on marine systems | Extrinsic | Anthropogenic GHG emissions | Ocean warming and sea ice loss  Increased ocean stratification and surface freshening  Altered primary productivity | Poleward shifts in marine biodiversity  Disruption of predator-prey relationships  Food web disruptions | ^16-18^ |
| 3 | Ocean acidification | Extrinsic | CO_2_ absorption by oceans | Decreased ocean pH  Reduced availability of free carbonate ions  Altered silica and carbonate chemistry | Disrupted development of calcifying organisms  Changes in community composition | ^18-20^ |
| 4 | Socio-geopolitical disruptions | Extrinsic | Global crises (e.g., war, human pandemics, crime) | Reduced cooperation and collaboration  Priority shifts  Gaps in long-term datasets and monitoring | Reduced capacity for evidence-based conservation  Diminished ability to respond to environmental challenges  Loss of scientific insights | ^21-23^ |
| 5 | Invasive alien species and disease | Transboundary | Human activity  Poleward eco-region shifts | Establishment of non-native species  Spread of pathogens | Changes in species composition  Altered ecosystem functioning | ^24-26^ |
| 6 | Pollution | Transboundary | Local human activity  Long-range pollutant transport | Rising contaminant load across ecosystems | Bioaccumulation in food webs  Diminished species fitness  Increased vulnerability to compounding stressors | ^27-29^ |
| 7 | Functioning of the Antarctic governance regime | Intrinsic | Diverging Party interests  Geopolitical pressures  Limitations of existing governance structures | Reduced cooperation and collaboration  Changes in environmental protection policies  Loss of relevance of the Antarctic governance regime | Diminished ability to respond to environmental challenges  Breakdown in coordinating conservation actions | ^22,30-32^ |
| 8 | Mineral & hydrocarbon exploitation | Intrinsic | Resource demands  Economic interests | Erosion of ATS authority  Physical habitat alteration  Localised pollution | Ecosystem degradation  Wildlife disturbance  Reframing of the region as commercially valuable | ^33,34^ |
| 9 | Bioprospecting | Intrinsic | Commercial interests  Scientific research | Erosion of ATS authority  Growing tension over benefit-sharing  Expansion of biological sampling activities | Loss of genetic diversity  Reframing of the region as commercially valuable | ^35-38^ |
| 10 | Human activity | Intrinsic | Human footprint | Disturbance to wildlife  Physical habitat alteration | Behavioural changes to wildlife  Ecosystem degradation | ^39-41^ |

**Supplementary Table 3**. Conservation threats grouped by management scope and DPSIR category. Threats included here were used as the basis for our study; groupings occurred based on the Drivers-Pressures-States-Impacts-Response (DPSIR) framework.

### Note 1.2. Topic modelling

In this section, we provide a brief overview of Latent Dirichlet Allocation topic modelling, followed by an in-depth explanation of how it was implemented and interpreted in our analysis.

#### 1.2.1 Background to Latent Dirichlet Allocation

We adopt the notation and approach outlined in ^4^ and ^42^.

Latent Dirichlet Allocation (LDA) is a method that uses probabilistic inference to discover the underlying topic structure in document collections (also referred to as a ‘corpus’). LDA assumes a generative model where each word is sampled from a topic’s word distribution, with the topic itself sampled from the document’s topic distribution (detailed below; **Supplementary Table 4**). This flexible framework allows words to appear in multiple topics with different probabilities, capturing different meanings in different contexts (e.g., ‘play ball’ versus ‘Shakespear’s play’). The generative model corresponds to a Bayesian network, which can be solved using statistical inference methods. As input, the method takes a document corpus cleaned of common functional words (e.g., ‘the’, ‘and’, ‘is’), where each document is represented as a ‘bag of words’ — an unordered collection of word frequencies. The outputs are: topic distributions for each document (*θ*); word distributions for each topic (*ϕ*); and topic assignments for each word (*z*). LDA performs well in practice because typically each document in a corpus tends to cover only a few topics, and each topic tends to be distinguished by a relatively small set of words.

1. For each topic *k* of *K*:
   (a) Draw the distribution of words for topic *k*

*ϕ_k_* ∼ Dirichlet(*β*)*.*

2. For each document *d* of *M*:
   (a) Draw the topic mix of document *d*

*θ_d_* ∼ Dirichlet(*α*)*.*

(b) Draw the number of words in document *d*

*N_d_* ∼ Poisson(*ξ*)*.*

The Poisson distribution assumed is not critical to the method, and more realistic assumptions can be made.

(c) For each *n*-th word of *N_d_*:
    i. Draw the topic of the *n*-th word

*z_d,n_* ∼ Categorical(*θ_d_*)*.*

ii. Given the *n*-th word’s topic, draw the word

*w_d,n_* ∼ Categorical(*ϕ_zd,n_*)*.*

**Supplementary Table 4**. Latent Dirichlet allocation notation.

| Symbol | Description |
| --- | --- |
| Counts: | |
| *K* | Number of topics covered by the corpus. The value is assumed known and fixed. *M* number of documents in the corpus. |
| *N_d_* | Number of words in the document *d*. |
| *V* | Number of words in the vocabulary |
| Variables: | |
| *D* | Corpus. A collection of documents, D = ^17wm^ |
| *θ_d_* | Topic distribution of document *d*. Takes values in the (K – 1)-simplex. |
| *ϕ_k_* | Word distribution of topic *k*. Takes values in the (*V* – 1)-simplex. |
| *w* | Word. A standard basis vector of length *V* indicating word position in vocabulary, i.e., a one-hot encoding (e.g., word 3 of 5 is *w*^3^ = (0,0,1,0,0)). |
| *w_d_* | Document *d.* A sequence of *N_d_* words, *w_d_* = (*w_1,_ w_2_,…*, $w_{N_{d}})$ |
| *z_d,n_* | Topic of word *w*_d,n_ (the *n*-th word in document *d*) *z_d,n_* $\in$ |
| Parameters: | |
| *α* | Dirichlet prior of the per-document topic distribution. A single value used equally for all topics. Smaller values mean each document contains fewer topics. |
| *β* | Dirichlet prior of the per-topic word distribution. A single value used equally for all words. Smaller values mean each topic concentrates on fewer words. |

The generative model has a geometric interpretation ^42^. Each document’s word frequencies and each topic *ϕ_k_* corresponds to a point in the (*V* − 1)-dimensional word simplex. The convex combination of all topics forms a (*K* −1)-dimensional topic-simplex (e.g., *K* = 2 forms a line segment and *K* = 3 a triangle), which is typically of much lower dimension than the word simplex (*K* ≪ *V*). Therefore, the method projects each document’s location from word space to topic space. For each document, its position in topic space is given by topic distribution *θ_d_*, which is the location that best explains its word frequencies in the probabilistic sense by maximising the likelihood of generating the corpus of documents.

The only observed variables are the words of the documents *w_d_*. The topics *ϕ_k_*, their proportions in each document *θ_d_*, and the assignments of each word to each topic *z_d,n_* are latent variables.

The LDA analysis amounts to posterior inference of *P*[*ϕ*_1:_*_K_,θ*_1:_*_M_,z*_1:_*_M,_*_1:_*_N_* | *D*]. While this full posterior includes topic assignments for each word, *z*_1:_*_M,_*_1:_*_N_*, in practice, we marginalise over these assignments to obtain the quantities of primary interest: the topic-word distributions *ϕ_k_* and the document-topic distributions *θ_d_*.

To obtain the posterior estimates of interest, we used the Python machine-learning module scikit-learn ^43^, which implements an online variational Bayes algorithm ^44^, which is a form of stochastic variational inference ^45^. Stochastic variational inference builds on variational inference, which solves high-dimensional optimisation problems using a coordinate ascent algorithm e.g., ^4^. The coordinate ascent algorithm is inefficient for large data sets because, in order to estimate the gradient of the objective function, it makes a full pass of all the data at each iteration. Stochastic variational inference improves efficiency by using a noisy estimate of the gradient obtained by subsampling only part of the data.

#### 1.2.2 Justification for selecting LDA

The choice of topic-modelling framework was guided by the analytical objectives of this study and the characteristics of the underlying corpus. Our aim was not simply to identify thematic clusters within a collection of documents, but to examine how major conservation threats emerged, persisted, and changed through time across scientific and policy discourse. Accordingly, our selection of LDA was guided by three considerations:

1. **Representation of documents as mixtures of topic** – Scientific abstracts and policy documents frequently discuss multiple conservation issues simultaneously. LDA was explicitly designed to represent documents as mixtures of topics ^4^, allowing individual documents to contribute probabilistically to multiple thematic areas rather than being assigned exclusively to a single cluster. This characteristic closely aligns with the structure of both scientific and policy documents and provides a natural framework for representing the thematic complexity of the corpus
2. **Interpretability of governance-relevant themes** – A central component of our workflow involved interpreting model-derived topics and assigning them to conservation threats relevant to Antarctic governance. This required a modelling framework that produced coherent and readily interpretable topics that could be systematically inspected, validated, and linked to substantive conservation challenges ^46^.
3. **Suitability for large-scale corpus analysis** – Our corpus comprised more than 90,000 scientific and policy documents spanning six decades. LDA was specifically developed to identify latent thematic structure within large document collections ^4^ and has been widely applied to similarly large-scale scientific and policy corpora. Its probabilistic framework therefore provides a natural and well-established approach for analysing patterns of discourse across extensive documentary records

These considerations made LDA a natural choice for the objectives of the study. Nevertheless, the field of topic modelling has expanded considerably since the introduction of LDA, with numerous alternative approaches developed to address different analytical objectives and document types e.g., see ^47,48,49^.Contemporary topic-modelling frameworks prioritise different aspects of topic quality and often involve trade-offs among semantic representation, interpretability, and other evaluation criteria ^46^. Consequently, no topic-modelling framework is universally superior ^50^; rather, the suitability of a given approach depends on the research objective and the characteristics of the underlying corpus.

Among contemporary alternatives, BERTopic was particularly relevant to our study because it combines topic modelling with transformer-based semantic embeddings while retaining interpretable topic representations ^51^. However, while both LDA and BERTopic can assign a document to multiple topics, the resulting values are generated in different ways. In LDA, document-topic mixtures are a primary model output and form the basis of the modelling framework. BERTopic can also generate topic probabilities, but these are derived following the clustering process rather than being estimated directly as part of the underlying model. Because our analysis relied on quantifying and aggregating document-topic mixtures across years and corpora to estimate the prevalence of conservation threats through time, the outputs generated by LDA were more directly aligned with the objectives of the study.

#### 1.2.3 LDA for Antarctic threats

##### Text pre-processing

For each document in the corpus (policy and scientific documents), we performed following pre-processing steps:

1.	Clean text.

2.	Tokenise text.

3.	Remove stop-words.

4.	Lemmatise tokens.

5.	Remove tokens with low and high document frequency.

We explain each of these pre-processing steps, below.

1. **Clean text.** We removed all characters that were not letters nor whitespace; replaced consecutive whitespace characters with a single whitespace; and converted all characters to lower case.
2. **Tokenise text.** Tokenisation refers to the process of breaking down text into smaller units called tokens, which typically represent words or other meaningful elements of the text. We used the word tokenize() function from the Python Natural Language Toolkit (NLTK) (Bird et al., 2009), NLTK’s recommended word tokeniser.
3. **Remove stop-words.** Stop-words are common words (e.g., “the”, ‘is’, ‘at’) that carry little meaningful content alone and are therefore removed from the texts before analysis. To create our stop-words list, we combined the standard NLTK list of English stop-words with the words the additional stop-words listed in Box 1 and Box 2, and further removed all tokens of length 1.

The additional stop-words were chosen by an iterative process, in consultation with the experts, who reviewed the top topic words each time the model was run. The words were chosen to ensure that the model would not focus on administrative or diplomatic aspects.

4. **Lemmatise tokens.** Lemmatisation is a process that reduces words to their base form (e.g., ‘studied’ and ‘studies’ become ‘study’). To perform lemmatisation, we used NLTK’s WordNetLemmatizer in its default setting, which treats every word as a noun. If the word is not found in WordNet’s dictionary, it returns the original word.
5. **Remove tokens with low and high document frequency.** We removed words that occurred in a very low and very high proportion of documents. Very frequent words are typically not informative for distinguishing between topics. For example, they might be domain-specific words that are not included in the standard stop-word lists, such as ‘study’ and ‘results’ in scientific papers. Very rare words may represent noise rather than meaningful topics, such as typographical errors. Alternatively, rare terms might be indicative of a topic. However, because the model relies on co-occurrence patterns across multiple documents to identify topics, they are not useful to the statistical model. Removing rare words also reduces the dimensionality of the problem.

We chose minimum and maximum word document frequencies of 0.01 and 0.7, respectively. These values were chosen based on iterative testing and manual validation against known pol- icy priorities and conservation threats in Antarctica. The maximum document frequency of 0.7 helped remove overly common terms that appeared in most documents but didn’t contribute to topic differentiation. The minimum document frequency of 0.01 ensured we retained terms that, while relatively rare, were important indicators of specific conservation threats and policy concerns. We validated these thresholds by checking whether key policy documents remained well-represented in the topic distributions and by confirming that known conservation threats were being captured in coherent topics with relevant associated terminology. This manual validation process helped ensure the model maintained sensitivity to important but potentially less frequent policy and conservation discussions while filtering out noise from common administrative language. In particular, we chose a low minimum frequency (0.01) to capture topics particular to capture ATS policy documents. Policy documents constituted a much smaller proportion of documents in the corpus compared to scientific papers, 5717 Policy documents Versus 85096 Scientific documents.

The removal of low- and high-document-frequency tokens was performed as part of the count vectorisation step in the code. We used scikit-learn’s CountVectorizer() to create a dictionary of words and their frequencies, which was then converted into a sparse matrix of tokens counts (i.e., D in matrix form), which are the observations used by the LDA algorithm. This reflects the ‘bag of words’ assumption of LDA — documents are treated as an unordered collection of word frequencies.

##### Topic extraction

To perform the LDA, we used the fit() method of the LatentDirichletAllocation() function from scikit-learn ^43^.

Because the corpus was large, we used the online variational Bayes’ algorithm (Hoffman et al., 2010) to obtain the posteriors (option learning method="online"). We chose a learning offset of 50, which means how quickly the model updates its parameters during training. This was chosen because the offset of 50.0 would have enough initial iterations with higher learning rates to capture diverse vocabulary, and with our topic number (75) and vocabulary size, this offset would allow for sufficient exploration of topic-word distributions and the appropriate time for specialized policy topics to emerge. We left the learning decay parameter at its default value of 0.7, which is in the range of values recommended in the documentation to guarantee convergence. We set the maximum number of passes over the training data to 10, and verified convergence by plotting the log-likelihood and perplexity metrics per iteration (**Supplementary Figure 3**). The log-likelihood curve shows diminishing returns after iteration 7, the perplexity decrease becomes minimal (< 1 per cent change) in the final iterations, and both metrics show stable, asymptotic behaviour in the final iterations.

LDA assumes the number of topics (K) is known and fixed. To choose the number of topics for the final results, we trialled the algorithm for values K ∈ [10, 20, . . . , 80, 100] ^52^, and we selected K = 90 based on both quantitative coherence metrics and expert assessment.

For our quantitative evaluation, we used Normalised Pointwise Mutual Information (NPMI) coherence ^53^, which measures how often the top words in each topic co-occur in documents compared to what would be expected if they were distributed independently. Higher NPMI scores indicate that the top words in topics frequently co-occur, suggesting the topics are semantically coherent and meaningful. This approach to measuring topic quality aligns well with human judgment, as validated by ^54^. The NPMI calculation for a pair of top words in the same topic *w*_1_ and *w*_2_ is

$$NPMI(\omega_{1},\omega_{2})=\frac{log(\frac{P(\omega_{1},\omega_{2})}{P(\omega_{1})P(\omega_{2})}}{-log P(\omega_{1},\omega_{2})}$$

**Supplementary Figure 3**. The perplexity score and log-likelihood versus iteration.

where P (wi) is the probability of the top-word i occurring in a document, P (ω_1_, ω_2_) is the probability of words 1 and 2 co-occurring, and the probabilities are calculated empirically from their frequencies from the corpus. We used the top 20 words for each topic, and calculated overall coherence for each topic as the average NPMI across all pairs of these top words, then averaged across all topics to evaluate each model.

We selected *K =* 90 topics for the final analysis (**Supplementary Figure 4**) based on both quantitative coherence metrics and expert assessment. The 90-topic model achieved the highest overall coherence score, indicating strong semantic consistency among top words, and subject-matter experts confirmed that this level of granularity provided the most useful and interpretable set of topics for our purposes. We used an iterative process in which experts reviewed topic–word distributions across different values of $K$and provided feedback to identify the most conceptually meaningful representation. Through this process, $K=90$ was affirmed as the most appropriate balance between quantitative coherence and thematic interpretability, providing sufficient granularity to capture distinct themes across scientific and policy discourse.

**Supplementary Figure 4**. Topic coherence versus number of topics (*K*).

To choose the α and β priors, we manually tested with combinations of lower than default α and β. Lower α (document topic prior = 0.1) makes each document’s topic distribution more sparse (concentrated on fewer topics) and encourages documents to be strongly associated with just a few topics rather than spread across many. This helped under-represented policy documents stand out because it allowed them to have strong associations with specific topics rather than being divided across many topics. Lower β (topic word prior = 0.005) makes each topic’s word distribution more sparse (concentrated on fewer words), and allows topics to be more specific and distinct from each other. This helped capture specialised vocabulary and themes from policy documents even if they are less frequent in the corpus. By decreasing the α and β values after each run, we came to the number stated above, which created a more specialised and sparse representation to capture under-represented policy content.

## Supplementary Note 2. Extended Discussion.

### Note 2.1. Spline fit oscillations and main Antarctic Treaty System Agreements

Internal and Transboundary threats often display oscillatory spline patterns, with attention rising, peaking, and subsequently declining over time. In this section, we examine the temporal trajectories of these threats in greater detail and assess how each ‘boom-and-bust’ cycle aligns with the negotiation, adoption, and implementation of major agreements within the history of the Antarctic Treaty System (ATS). Our primary focus is on Antarctic Treaty Consultative Meeting (ATCM) policy discussions, though we also highlight relevant patterns in the scientific corpus where appropriate.

#### 2.1.1 Internal Threats

##### Functioning of the Antarctic Treaty System

The Antarctic Treaty (AT) was originally established with geopolitical motivations, aiming to resolve conflicts over territorial claims in the region ^55,56^. From its entry into force in 1961 until 1970, attention focused primarily on the Treaty’s implementation, with numerous discussions concerning its functioning, including the establishment of procedural rules. After the Treaty’s initial implementation phase and establishment of its institutional framework, debates about the functioning of the Antarctic Treaty System (ATS) significantly declined between 1970 and 1975 (through reaching a local minimum in 1979, comprising 15.3% [95% CIs = 13.9—17.0%] of ATCM policy literature at this time). However, challenges to the Treaty’s operation resurfaced in the 1980s, a period characterized by a rapid increase in the number of Antarctic Treaty Consultative Parties ^57^. This expansion was driven largely by two major factors: contentious debates over mineral exploitation in Antarctica and Malaysia’s criticisms of the Treaty at the United Nations General Assembly ^56^. The prospect of resource exploitation attracted several developing nations, such as Brazil, China, and Uruguay, to join the Treaty ^58,59^. In addition, as a response to Malaysia’s criticisms —which highlighted concerns about inclusivity and the growing influence of developing nations—the rules governing ascension to Consultative status were relaxed. Consequently, the Treaty faced an unprecedented increase in the number of Parties, prompting a notable rise in discussions about the ATS’s functioning from 1982 to 1992 (reaching a global maximum of 21.5% [95% CIs = 19.0—22.4%] of ATCM policy literature at this time). These issues were partially addressed with the signing of the Protocol and the banning of non-living resources exploitation, which led to a subsequent and marked decline in such debates.

##### Mineral and hydrocarbon exploitation

Interest in Antarctic mineral resources first appeared on the agenda of the Antarctic Treaty Consultative Meeting (ATCM) in 1970 ^60^. However, the possibility of mineral resource exploration was a divisive topic, sparking intense debate and resulting on a significant increase in interest in the topic throughout the 1970s and early 1980s. During this period, a coalition of developing countries advocating for exploitation emerged ^61^, while other Parties—particularly industrialized nations and original Treaty signatories such as France and Australia—voiced strong opposition, citing concerns over environmental impacts ^62^. These debates are reflected in a significant increase in interest in the topic between 1967 and 1981 and culminated in the negotiation of the Convention on the Regulation of Antarctic Mineral Resource Activities (CRAMRA), which began in 1982 ^58,59^. Discussions on this topic gradually decreased during the subsequent CRAMRA negotiations (with a significant decrease between 1982 and 1991), which is expected as the Parties addressed their concerns, eventually culminating in the signing of CRAMRA at the Fourth Special Antarctic Treaty Consultative Meeting on Antarctic Mineral Resources in 1988. However, despite six years of negotiations, significant doubts about CRAMRA persisted. When CRAMRA was formally adopted, some Consultative Parties, including the United Kingdom, openly expressed reservations regarding the treaty’s efficacy ^60^. Ultimately, these doubts contributed to the Convention’s failure to be ratified.

##### Bioprospecting

With respect to the ATS, bioprospecting is defined as:

Any activity of search, identification, description, collection, survey, monitoring, cultivation, replication, or any other scientific investigation processes, performed on indigenous biological species, carried on within the area defined in Article VI of the Antarctic Treaty, with the initial intention to consider potential industrial or commercial derived products or applications, notably through the development of patentable material or process ^63^

Despite the potential implications, Treaty Parties have given limited consideration to bioprospecting, treating it as a subject for ongoing discussion rather than adopting formal regulation ^64,65^. This permissive stance has resulted in minimal representation of the threat within the ATCM policy corpus. However, a small but sustained increase on the topic occurred between 1995 and 2012, after which a plateau occurred (this threat comprised approximately 0.5% [95% CIs = 0.4—0.6%] of ATCM policy literature in 2012). This increased interest likely coincided with the adoption of the Protocol Environmental Protection to the Antarctic Treaty (adopted 1991, entered into force 1998), which raised questions about how bioprospecting might be managed within the ATS framework ^66^. These discussions have largely remained exploratory, though, with little substantive regulation emerging and only limited engagement from Treaty Parties overall ^67^.

##### Human Activity Impacts

Human activity impacts are an inherent concern as long as humans are present in the Antarctic region. Although new measures or treaties can mitigate these impacts, and such interventions may temporarily reduce the prominence of the issue in discussions, this is a challenge that cannot be fully resolved. This topic was heavily discussed during the period of implementation of the Treaty ^68^, reflecting the need to define governance principles for a newly regulated space, but also during the beginning of the discussions about mineral exploitation ^56^ (which can be observed in the significant increase that happened between 1967 and 1981). Following this period, discussions remained relatively stable until 1988, when they increased sharply. This rise aligns with the conclusion of the Convention on the Regulation of Antarctic Mineral Resource Activities (CRAMRA), which, despite being signed, faced persistent scepticism from several states ^60^. The refusal of Australia and France to ratify CRAMRA in 1989 ^69^ marked a decisive turning point, as environmental concerns began to outweigh the perceived benefits of resource development. This shift in priorities led to the negotiation of the Protocol on Environmental Protection to the Antarctic Treaty (the ‘Protocol’), adopted in 1991. The Protocol, a landmark addition to the Treaty, elevated conservation to a central objective of Antarctic governance. Its adoption triggered a sustained increase in discussions on human activity impacts, a trend that continued until 2012. This sustained significant increase in debates about human activity impacts indicates that the Protocol not only codified environmental protection in law but also entrenched it as a persistent and prominent theme in Antarctic governance.

#### 2.1.2 Transboundary threats

##### Invasive alien species & disease

Invasive species and disease represent a transboundary threat because they can be introduced from outside the Antarctic region and also spread among its distinct biogeographic areas. The movement of people and equipment between stations facilitates both inter- and intra-regional transfer, potentially disrupting local endemism and biogeographic structure ^70,71^. ATCM policy discourse on invasive species and disease began to rise from 1987 and has shown a generally upward trajectory through to 2023, with the steepest increases occurring prior to 2015.The initial growth coincided with negotiations leading to the Protocol (adopted in 1991, entered into force in 1998), which established invasive species management as a formal conservation obligation under Annex II (Conservation of Antarctic Fauna and Flora). Subsequent increases were reinforced by concern over expanding tourism and logistics ^24,72^ and by the Committee for Environmental Protection (CEP), which identified invasive species and wildlife disease as priority management issues in its multi-year strategic reports. These include the CEP’s Five-Year Work Plan ^73^, which emphasises biosecurity coordination among Treaty Parties, and the Non-native Species Manual ^25^, developed to provide best-practice guidelines for preventing, detecting, and responding to incursions across diverse operational contexts. Even so, the issue has remained peripheral within the policy arena, accounting for only around 1% (95% CIs= 0.7%—1.3%) of ATCM discussions at its peak in 2023. This limited engagement likely reflects the region’s historical protection from non-native species, conferred by its isolation, extreme environment, and low human presence ^74^. However, as human activity intensifies and environmental barriers continue to weaken, this level of attention may no longer be commensurate with the growing risk.

##### Pollution

Pollution is classified as a Transboundary threat because pollutants can reach the Antarctic region in various ways, including from diffuse sources at the global scale and locally through point sources. Long-range atmospheric and oceanic transport delivers pollutants such as black carbon and persistent organic compounds from lower latitudes, while local contamination arises primarily from wastewater discharge, fuel spills, and hydrocarbon leakage associated with research stations and coastal infrastructure ^27-29^. Policy discourse on pollution began to intensify in the late 1970s, when early ATCM discussions focused on oil contamination and liability, particularly in relation to ship-borne discharges ^75^. The spline trajectory first showed a significant positive gradient in 1977 (occupying 1.2% [95% CIs = 0.9—1.5%] of ATCM policy literature), reflecting growing policy interest in pollution and environmental liability. This heightened attention persisted for two decades, peaking in 1997 at 3.7% (95% CI = 3.0–4.4%) as the Protocol entered into force and formally codified pollution control through its environmental annexes. ATCM discussions declined thereafter, as Annex III (Waste Disposal and Waste Management) and Annex IV (Prevention of Marine Pollution) established clear regulatory mechanisms that reduced the need for continued debate. Discussions plateaued around 2013 and have begun to rise again, reflecting renewed concern over marine plastic pollution ^76^.

##### Socio-geopolitical disruptions

Socio-geopolitical disruptions comprise externally driven stressors, such as war or pandemics, that undermine international cooperation in the Antarctic region ^21,22^. The Antarctic Treaty was founded on this idea, reflecting Cold War concerns that Antarctica might become a site of geopolitical rivalry ^77^, which explains the prominence of these issues in ATCM discussions during the 1960s (4.0% [95% CIs = 3.2—5.0%] in 1961). Policy attention to this threat declined over time, stabilising in the 1980s and 1990s with debates over ATS membership expansion and liability arrangements, before plateauing again during the 2010s. Reasons for this second plateau off are varied. They include discussions prompted by the COVID-19 pandemic and the Russian invasion of Ukraine, as well as more diffuse debates such as the designation of Antarctic Specially Protected Areas (ASPAs) and Managed Areas (ASMAs). Debates on some protected areas have become increasingly politicised and framed through legal-interpretive disputes ^78^, for example whether prohibiting entry to certain areas is compatible with Treaty principles of scientific freedom and inspection ^79^. As a result, seemingly routine designations have failed to pass, and progress has slowed markedly, with only nine ASPAs designated since 2012 and no new ASMAs since 2009 ^80^.

### Note 2.2. CCAMLR, science-based governance, and regime interactions.

We did not include policy discussions from the Convention on the Conservation of Antarctic Marine Living Resources (CCAMLR Convention) or conservation-related decisions made by its governing body, the Commission for the Conservation of Antarctic Marine Living Resources (CAMLR Commission), in our analysis. Our study therefore refrains from drawing conclusions about how effectively CCAMLR addresses marine conservation threats or contributes to the broader performance of the ATS. Instead, we provide complementary context from the existing literature on CCAMLR to illustrate how its experience both parallels and informs the themes identified in our analysis. We first consider scholarship that evaluates CCAMLR’s internal decision-making processes and the challenges of maintaining science-based consensus among Parties. We then discuss CCAMLR’s external interactions with emerging global governance arrangements, such as the Biodiversity Beyond National Jurisdiction (BBNJ) Agreement.

Our analysis shows that, while the best available science informs policy within the ATS, scientific and policy priorities are not always aligned in the ATCM. Similar patterns are evident within the CAMLR Commission, where political dynamics have constrained science-based management across the Southern Ocean ^81^. These constraints have arisen from the use of consensus rules to block or dilute conservation measures, divergent interpretations of “rational use” to justify continued fishing, and the growing politicisation of decision-making ^82^. Such dynamics have reduced CCAMLR’s ability to act on scientific advice and weakened trust in its governance processes. This is exemplified by the protracted negotiations and reduced scope of the Ross Sea and East Antarctic MPA proposals, where political resistance repeatedly overrode strong scientific consensus ^83^. The experience of CCAMLR offers a cautionary perspective for the ATS, demonstrating that even well-institutionalised science-based governance can be undermined by political divergence and procedural inertia.

Despite these internal challenges, CCAMLR also provides an instructive example of how Antarctic institutions can engage with emerging global governance frameworks. Drawing on its pioneering role in establishing high-seas marine protected areas (MPAs), including the South Orkney Islands Southern Shelf (2009) and Ross Sea region (2016) MPAs, CCAMLR has served as a reference point for developing the BBNJ Agreement’s framework for area-based management tools ^84^. In particular, CCAMLR’s conservation measures (e.g., CMs 91-04 and 91-05), scientific review processes, and institutional architecture have informed how the BBNJ’s scientific and compliance mechanisms might operate. CCAMLR’s ongoing contribution to global negotiations demonstrates that engagement beyond the ATS’s jurisdiction is both feasible and valuable.

## Supplementary Note 3. Aligning topics with conservation threats.

To align LDA topics with conservation threats, we conducted a structured qualitative review involving thematic analysis ^85^. Team members first independently assessed the relevance of each topic and then deliberated collectively to determine whether it should be retained as related to a specific threat. Complete details are provided in the main text. Below, we provide detailed information on the topics considered for each threat and outline the rationale for their inclusion or exclusion in the subsequent spline analysis.

### Note 3.1. Topic selection for External Threats.

Climate change impacts on marine and terrestrial systems (combined threats)

| **Topic 4** | | | | | | |
| --- | --- | --- | --- | --- | --- | --- |
|  | Year | Document Type | Title | Substantively Related? | Retained Topic? | Notes |
| 1 | 2021 | SCI | Declining citation accuracy in polar research | N | N | topic focuses on chlorofluorocarbon trends and monitoring, which are relevant to atmospheric science and ozone depletion but do not address ecological impacts on terrestrial or marine systems driven by climate change |
| 2 | 1989 | SCI | Substitutes and alternatives of CFC-113 based cleaning - an overview | N |  |  |
| 3 | 1988 | SCI | CFC/OZONE/GREENHOUSE ISSUES. | N |  |  |
| 4 | 2020 | SCI | Quantifying contributions of chlorofluorocarbon banks to emissions and impacts on the ozone layer and climate | N |  |  |
| 5 | 2001 | SCI | Study on the abundance of CFCs varying with the latitude at the bottom of the troposphere in the southern hemisphere | N |  |  |
| 6 | 2011 | SCI | Interannual variability of CFC-11 absorption by the ocean: An offline model study | N |  |  |
| 7 | 2009 | SCI | Simultaneous determination of CFC-11, CFC-12 and CFC-113 in seawater samples using a purge and trap gas-chromatographic system | N |  |  |
| 8 | 1991 | SCI | Halocarbons in the Atmosphere: Trends and Vertical Profiles | N |  |  |
| 9 | 1999 | SCI | Significance of selected halocarbons monitoring in air samples collected in the Terra Nova Bay region (northern Victoria Land, Antarctica) | N |  |  |
| 10 | 2010 | SCI | Atlantic CFC data in CARINA | N |  |  |
| **Topic 21 (Terrestrial systems only)** | | | | | | |
|  | Year | Document Type | Title | Substantively Related? | Retained Topic? | Notes |
| 1 | 2016 | SCI | Validation of the Antarctic Snow Accumulation and Ice Discharge basal stress boundary of the southeastern region of the Ross Ice Shelf, Antarctica | Y | Y | Topic focuses on how climate change is altering the dynamics of Antarctica’s terrestrial cryosphere, including ice sheets, glaciers, and snow accumulation patterns |
| 2 | 1994 | SCI | Seasonal changes of sea-ice characteristics off east Antarctica | Y |  |  |
| 3 | 2020 | SCI | Formation of sea ice ponds from ice-shelf runoff, adjacent to the McMurdo Ice Shelf, Antarctica | Y |  |  |
| 4 | 1987 | SCI | Fast glacier flow: ice streams, surging, and tidewater glaciers | Y |  |  |
| 5 | 2006 | SCI | Polesapart: Global warming and the Antarctic ice sheets | Y |  |  |
| 6 | 2017 | SCI | Regional Changes in Icescape Impact Shelf Circulation and Basal Melting | Y |  |  |
| 7 | 1979 | SCI | The dynamics of marine ice sheets. | Y |  |  |
| 8 | 2009 | SCI | Large-Scale Dynamics of Ice Shelves | Y |  |  |
| 9 | 2006 | SCI | Ice cores \| Dynamics of the east antarctic ice sheet | Y |  |  |
| 10 | 2016 | SCI | Modeling ice shelf/ocean interaction in Antarctica: A review | Y |  |  |
| **Topic 36 (Terrestrial systems only)** | | | | | | |
|  | Year | Document Type | Title | Substantively Related? | Retained Topic? | Notes |
| 1 | 1999 | SCI | Accumulation and proxy-temperature variability in Dronning Maud Land, Antarctica, determined from shallow firn cores | Y | Y | topic focuses on paleoclimate reconstruction and accumulation variability from ice cores, offering key context for understanding climate-driven changes to Antarctica’s terrestrial cryosphere |
| 2 | 2008 | SCI | Preliminary evidence indicating Dome A (Antarctica) satisfying preconditions for drilling the oldest ice core | Y |  |  |
| 3 | 2012 | SCI | A detailed 2840 year record of explosive volcanism in a shallow ice core from Dome A, East Antarctica | Y |  |  |
| 4 | 2014 | SCI | High-resolution 900 year volcanic and climatic record from the Vostok area, East Antarctica | Y |  |  |
| 5 | 1994 | SCI | High resolution climatic information from short firn cores, western Dronning Maud Land, antarctica | Y |  |  |
| 6 | 1998 | SCI | A chronology for the Dome C deep ice-core site through radio-echo layer correlation with the Vostok ice core, Antarctica | Y |  |  |
| 7 | 2002 | IP | Deep ice core drilling project II at Dome Fuji, Antarctica | Y |  |  |
| 8 | 1981 | SCI | The chemical stratigraphy of polar ice sheets - a method of dating ice cores. | Y |  |  |
| 9 | 2007 | SCI | The EDC3 chronology for the EPICA Dome C ice core | Y |  |  |
| 10 | 2006 | SCI | Gas age-ice age differences and the chronology of the Vostok ice core, 0-100 ka | Y |  |  |
| **Topic 37** | | | | | | |
|  | Year | Document Type | Title | Substantively Related? | Retained Topic? | Notes |
| 1 | 2019 | SCI | The Southern Annular Mode and Southern Ocean Surface Westerly Winds in E3SM | Y | Y | Topic examines large-scale climate dynamics and circulation changes that influence Antarctic sea ice, precipitation, and energy balance |
| 2 | 2014 | SCI | Comparison of surface albedo feedback in climate models and observations | Y |  |  |
| 3 | 2019 | SCI | On the role of the atmospheric energy transport in 2Ã—3CO2-induced polar amplification in CESM1 | Y |  |  |
| 4 | 2001 | SCI | Biases in AMIP model simulations of the east China monsoon system | Y |  |  |
| 5 | 2020 | SCI | Evolution of the meridional shift of the subtropical and subpolar westerly jet over the Southern Hemisphere during the past 21,000 years | Y |  |  |
| 6 | 2016 | SCI | Net precipitation of Antarctica: Thermodynamical and dynamical parts of the climate change signal | Y |  |  |
| 7 | 2023 | SCI | Meridional Heat Transport in the DeepMIP Eocene Ensemble: Non-CO2 and CO2 Effects | Y |  |  |
| 8 | 2020 | SCI | Breakdown of the linear relationship between the southern hemisphere hadley cell edge and jet latitude changes in the last glacial maximum | Y |  |  |
| 9 | 1987 | SCI | Sea-ice and the Antarctic winter circulation. | Y |  |  |
| 10 | 2006 | SCI | A physical mechanism of the atmospheric response over Antarctica to decadal trends in tropical SST | Y |  |  |
| **Topic 44 (Marine systems only)** | | | | | | |
|  | Year | Document Type | Title | Substantively Related? | Retained Topic? | Notes |
| 1 | 2020 | SCI | Brief communication: Evaluating Antarctic precipitation in ERA5 and CMIP6 against CloudSat observations | Y | Y | This topic primarily focuses on climate model evaluation and projected changes in sea ice, sea-level rise, and Southern Ocean dynamics |
| 2 | 2020 | SCI | CO2 Increase Experiments Using the CESM: Relationship to Climate Sensitivity and Comparison of CESM1 to CESM2 | Y |  |  |
| 3 | 2008 | SCI | Cystacanths of acanthocephala in notothenioid fish from the Beagle Channel (sub-Antarctica) | N |  |  |
| 4 | 2021 | SCI | Differential credibility of climate modes in cmip6 | Y |  |  |
| 5 | 2016 | SCI | The leading modes of decadal SST variability in the Southern Ocean in CMIP5 simulations | Y |  |  |
| 6 | 2020 | SCI | Assessment of Sea Ice Extent in CMIP6 With Comparison to Observations and CMIP5 | Y |  |  |
| 7 | 2021 | SCI | Future Sea Level Change Under Coupled Model Intercomparison Project Phase 5 and Phase 6 Scenarios From the Greenland and Antarctic Ice Sheets | Y |  |  |
| 8 | 1999 | SCI | Dolichiscus spinosetosus n. sp., a new arcturid isopod from the Weddell Sea, Antarctica | N |  |  |
| 9 | 2006 | SCI | Zonally symmetric normal modes associated with the AO/NAM under a seasonally varying background climatology | Y |  |  |
| 10 | 2015 | SCI | An intercomparison of CMIP5 and CMIP3 models for interannual variability of summer precipitation in Pan-Asian monsoon region | Y |  |  |
| **Topic 47** | | | | | | |
|  | Year | Document Type | Title | Substantively Related? | Retained Topic? | Notes |
| 1 | 2017 | SCI | The ecological value of long-term studies of birds and mammals in Central America, South America and Antarctica | Y | Y | topic examines the ecological consequences of climate change across Antarctic ecosystems, including benthic communities, microbes, and vegetation. |
| 2 | 2022 | SCI | Interactive Effects of Warming and Pollutants on Marine and Freshwater Invertebrates | Y |  |  |
| 3 | 2018 | SCI | Predicting ecological responses in a changing ocean: the effects of future climate uncertainty | Y |  |  |
| 4 | 2018 | SCI | Linking ross sea coastal benthic communities to environmental conditions: Documenting baselines in a spatially variable and changing world | Y |  |  |
| 5 | 2023 | SCI | Antarctica's vegetation in a changing climate | Y |  |  |
| 6 | 2019 | SCI | Insights on the environmental impacts associated with visible disturbance of ice-free ground in Antarctica | Y |  |  |
| 7 | 2012 | SCI | Possible effects of global environmental changes on Antarctic benthos: A synthesis across five major taxa | Y |  |  |
| 8 | 2020 | SCI | Antibiotic resistance in minimally human-impacted environments | Y |  |  |
| 9 | 2015 | SCI | The Southern Ocean ecosystem under multiple climate change stresses - an integrated circumpolar assessment | Y |  |  |
| 10 | 2023 | SCI | Measuring the effect of climate change in Antarctic microbial communities: toward novel experimental approaches | Y |  |  |
| **Topic 62 (Terrestrial systems only)** | | | | | | |
|  | Year | Document Type | Title | Substantively Related? | Retained Topic? | Notes |
| 1 | 2000 | SCI | Utilization of automatic weather station data for forecasting high wind speeds at Pegasus Runway, Antarctica | Y | Y | This topic consists of meteorological monitoring and climatology studies, which inform terrestrial climate research |
| 2 | 1990 | SCI | The effect of wind direction and orography on air temperature at the "Arctowski' Station | Y |  |  |
| 3 | 1993 | SCI | Climatology of the east Antarctic Ice Sheet (100Â°E to 140Â°E) derived from automatic weather stations | Y |  |  |
| 4 | 2015 | SCI | Climate conditions at perennially ice-covered lake Untersee, East Antarctica | Y |  |  |
| 5 | 2004 | SCI | Meteorological conditions of the Basen nunatak in western Dronning Maud Land, Antarctica, during the years 1989-2001 | Y |  |  |
| 6 | 2016 | SCI | The surface climatology of the Ross Ice Shelf Antarctica | Y |  |  |
| 7 | 2008 | SCI | Meteorological observations at Syowa Station in 2004 by the 45th Japanese Antarctic Research Expedition | Y |  |  |
| 8 | 2022 | SCI | Analysis of extreme wind events in the Weddell Sea region (Antarctica) at Belgrano II Station | Y |  |  |
| 9 | 1984 | SCI | Surface meteorological data during oversnow traverse in 1982-1983 (Shirase Glacier). | Y |  |  |
| 10 | 1985 | SCI | Meteorological data at Mizuho Station, Antarctica, in 1983. | Y |  |  |
| **Topic 82** | | | | | | |
|  | Year | Document Type | Title | Substantively Related? | Retained Topic? | Notes |
| 1 | 2004 | SCI | Antarctic stratospheric ozone from the assimilation of occultation data | N | N | Topic focuses on stratospheric ozone dynamics and monitoring, which are atmospheric processes distinct from the ecological and physical impacts associated with climate change impacts |
| 2 | 2005 | SCI | Vertical structure of the anomalous 2002 Antarctic ozone hole | N |  |  |
| 3 | 1988 | SCI | Antarctic measurements of ozone by SAGE II in the spring of 1985, 1986, and 1987 | N |  |  |
| 4 | 1986 | SCI | Antarctic springtime measurements of ozone, nitrogen dioxide, and aerosol extinction by Sam II, Sage, and Sage II | N |  |  |
| 5 | 1990 | SCI | Sixty-four years of regular ozone measurements | N |  |  |
| 6 | 1997 | SCI | Effect of stratospheric ozone variations on UV radiation and on tropospheric ozone at high latitudes | N |  |  |
| 7 | 2009 | SCI | Height resolved ozone hole structure as observed by the Global Ozone Monitoring Experiment-2 | N |  |  |
| 8 | 2011 | SCI | Forecasts and assimilation experiments of the Antarctic ozone hole 2008 | N |  |  |
| 9 | 2021 | SCI | Simulation of Record Arctic Stratospheric Ozone Depletion in 2020 | N |  |  |
| 10 | 2005 | SCI | Objective evaluation of the impact of ral GOME ozone profiles on met office 3D-VAR analyses | N |  |  |
| **Topic 85** | | | | | | |
|  | Year | Document Type | Title | Substantively Related? | Retained Topic? | Notes |
| 1 | 1995 | SCI | Formation and discharge of deep and bottom water in the northwestern Weddell Sea | N | N | This topic comprises largely descriptive studies of deep and intermediate water masses, with limited Antarctic focus |
| 2 | 1986 | SCI | Ra-228 in the deep Indian Ocean | N |  |  |
| 3 | 1995 | SCI | On the synoptic hydrography of intermediate and deep water masses in the Iceland Basin | N |  |  |
| 4 | 1968 | SCI | The continuity of water masses along the western boundary of the tasman and coral seas | N |  |  |
| 5 | 2000 | SCI | Deep and bottom water of the Bransfield Strait eastern and central basins | N |  |  |
| 6 | 1961 | SCI | The subsurface water masses in the Western South pacific ocean | N |  |  |
| 7 | 2003 | SCI | Intermediate water masses off south-southwest Portugal: Chemical tracers | N |  |  |
| 8 | 1994 | SCI | Western boundary current variability off French Guiana as observed from moored current measurements | N |  |  |
| 9 | 1984 | SCI | DEEP CORE DRILLING: ELECTRO-MECHANICAL OR THERMAL DRILL? | N |  |  |
| 10 | 1995 | SCI | Oxygen isotope study of water masses in the Princess Elizabeth Trough, Antarctica | N |  |  |
| **Topic 87 (terrestrial systems only)** | | | | | | |
|  | Year | Document Type | Title | Substantively Related? | Retained Topic? | Notes |
| 1 | 1984 | SCI | Glacier flexure and the position of grounding lines: measurements by tiltmeter on Rutford ice stream, Antarctica. | Y | Y | This topic focuses on grounding line dynamics, glacier flow, and ice marginal processes |
| 2 | 2013 | SCI | Sediment deposition at the modern grounding zone of Whillans Ice Stream, West Antarctica | Y |  |  |
| 3 | 2018 | SCI | Grounding lines detecting using Landsat8 Oli and CryoSat-2 data fusion | Y |  |  |
| 4 | 1969 | SCI | A meridional surface wind speed profile in MacRobertson Land, Antarctica | Y |  |  |
| 5 | 2007 | SCI | Ice flow estimation of Shirase Glacier by using JERS-1/SAR image correlation | Y |  |  |
| 6 | 1995 | SCI | Pliocene-Pleistocene radiolarian biostratigraphy and palaeoceanography of the North Atlantic | N |  |  |
| 7 | 2005 | SCI | Assessing the ability of numerical ice sheet models to simulate grounding line migration | Y |  |  |
| 8 | 2010 | SCI | Grounding-line basal melt rates determined using radar-derived internal stratigraphy | Y |  |  |
| 9 | 2014 | SCI | Use of L-band SAR data for monitoring of ice sheet marginal zone on East Antarctica | Y |  |  |
| 10 | 1984 | SCI | Studies of ice formation and ice fabric on the Law Dome, Antarctica. | Y |  |  |

Ocean acidification

| **Topic 12** | | | | | | | |
| --- | --- | --- | --- | --- | --- | --- | --- |
|  | Year | Document Type | Title | Substantively Related? | Retained Topic? | Notes |  |
| 1 | 2000 | SCI | Photoacclimation by the Antarctic flagellate Pyramimonas sp. (Prasinophyceae) in response to iron limitation | N | N | This topic focuses on phytoplankton production and nutrient limitation, particularly in relation to iron availability |  |
| 2 | 2001 | SCI | Phytoplankton processes. Part 2: Rates of primary production and factors controlling algal growth during the Southern Ocean Iron RElease Experiment (SOIREE) | N |  |  |  |
| 3 | 2000 | SCI | Photoacclimation by the antarctic flagellate pyramimonas sp. (prasinophyceae) in response to iron limitation | N |  |  |  |
| 4 | 1991 | SCI | New production in polar regions: the role of environmental controls | N |  |  |  |
| 5 | 1979 | SCI | 13c and15n uptake by Phytoplankton in the Antarctic Upwelling Area: Results from the Antiprod I Cruise in the Indian Ocean Sector | N |  |  |  |
| 6 | 1992 | SCI | Major elements, nutrients, and plankton biomass in the ice edge and an offshore region of the Indian Ocean sector of the Southern Ocean | N |  |  |  |
| 7 | 2015 | SCI | Phytoplankton growth rates in the Ross Sea, Antarctica | N |  |  |  |
| 8 | 2010 | SCI | Iron-binding ligand production and copper speciation in an incubation experiment of Antarctic Peninsula shelf waters from the Bransfield Strait, Southern Ocean | N |  |  |  |
| 9 | 2002 | SCI | Abundance of Antarctic picophytoplankton and their response to light and nutrient manipulation | N |  |  |  |
| 10 | 2003 | SCI | Ratios of Si, C and N uptake by microplankton in the Southern Ocean | N |  |  |  |
| **Topic 19** | | | | | | | |
|  | Year | Document Type | Title | Substantively Related? | Retained Topic? | Notes |  |
| 1 | 2006 | SCI | Potential of combined U-Pb geochronology and Hf isotope geochemical studies on zircon to aid sedimentary provenance within Antarctica: Examples from West Antarctica | Y | Y | This topic includes research on carbonate chemistry and calcifying organisms relevant to ocean acidification, alongside geochemical studies that reflect broader biogeochemical methods |  |
| 2 | 2011 | SCI | Biometry of detached Emiliania huxleyi coccoliths along the Patagonian Shelf | Y |  |  |  |
| 3 | 2023 | SCI | Zircon geochronology and geochemistry of felsic gneisses from Harvey Nunatak, Napier Complex in East Antarctica | Y |  |  |  |
| 4 | 2005 | SCI | Search for psychrophilic methylotrophic bacteria in biotopes of the Antarctica | Y |  |  |  |
| 5 | 2022 | SCI | Emiliania huxleyi biometry and calcification response to the Indian sector of the Southern Ocean environmental gradients | Y |  |  |  |
| 6 | 2006 | SCI | The source of granitic gneisses and migmatites in the Antarctic Peninsula: A combined U-Pb SHRIMP and laser ablation Hf isotope study of complex zircons | Y |  |  |  |
| 7 | 2013 | SCI | The 2008 Emiliania huxleyi bloom along the Patagonian Shelf: Ecology, biogeochemistry, and cellular calcification | Y |  |  |  |
| 8 | 2011 | SCI | Characterization of magma from inclusions in zircon: Apatite and biotite work well, feldspar less so | Y |  |  |  |
| 9 | 1986 | SCI | Geochemistry and sedimentation. | Y |  |  |  |
| 10 | 2015 | SCI | Carbonate saturation state of surface waters in the Ross Sea and Southern Ocean: Controls and implications for the onset of aragonite undersaturation | Y |  |  |  |

### Note 3.2. Topic selection for Transboundary Threats.

Socio-geopolitical disruptions

| **Topic 6** | | | | | | | |
| --- | --- | --- | --- | --- | --- | --- | --- |
|  | Year | Document Type | Title | Substantively Related? | Retained Topic? | Notes |  |
| 1 | 1961 | IP | Speech by R.O. Nordland, Leader of the Norwegian Delegation, at the opening session of the Meeting. 10 July 1961 | N | N | Topic consists primarily of ceremonial speeches and meeting logistics, which do not substantively address geopolitical dynamics or institutional disruptions |  |
| 2 | 1964 | WP | Opening statement from the Head of the Japanese delegation | N |  |  |  |
| 3 | 1979 | WP | Declaration on the twentieth anniversary of the Antarctic Treaty | N |  |  |  |
| 4 | 1970 | IP | Closing Speech by Ambassador A. Matsui, the Chairman of the Sixth Consultative Meeting, on 31 October 1970 | N |  |  |  |
| 5 | 1962 | WP | Place and date of the next meeting | N |  |  |  |
| 6 | 1997 | IP | Opening address by Mr Aliocha Nedeltchev, Head of the delegation of the Republic of Bulgaria | N |  |  |  |
| 7 | 1970 | IP | Invitation to a Sunday Excursion | N |  |  |  |
| 8 | 1970 | IP | Speech by Mr. A. Matsui, the Chairman of the 6th Consultative Meeting | N |  |  |  |
| 9 | 1981 | WP | Opening statement by the Head of the delegation of the Federal Republic of Germany, Ambassador Hans Guenter Sulinima | N |  |  |  |
| 10 | 1995 | IP | Opening address by the representative of Greece | N |  |  |  |
| **Topic 78** | | | | | | | |
|  | Year | Document Type | Title | Substantively Related? | Retained Topic? | Notes |  |
|  | 1992 | IP | Annex on liability and compensation for Antarctic activities | Y | Y | Topic reflects legal and institutional tensions within the ATS that are shaped by broader geopolitical dynamics, including debates over liability, state responsibility, and territorial recognition. Documents in this group address unresolved negotiations around the Liability Annex, questions of adherence to conservation conventions, and conflicting interpretations of international law. |  |
|  | 1991 | IP | Annex on liability and compensation for Antarctic activities | Y |  |  |  |
|  | 2013 | SCI | To apply or to declare, or both? Links between the two types of intervention under the ICJ statute | Y |  |  |  |
|  | 1987 | IP | Report submitted by the Depositary Government (UK) in accordance with Recommendation XIII-2, Paragraph 2(d) relating to the Convention for the Conservation of Antarctic Seals | Y |  |  |  |
|  | 1994 | IP | Liability reference guide for the Working Group to strengthen the Liability Annex for the Environmental Protection Protocol to the Antarctic Treaty System | Y |  |  |  |
|  | 1996 | IP | International convention on liability and compensation for damage in connection with the carriage of hazardous and noxious substances by sea, 1996 | Y |  |  |  |
|  | 2023 | IP | Report by the United Kingdom as Depositary Government for the Convention for the Conservation of Antarctic Seals (CCAS) in Accordance with Recommendation XIII-2, Paragraph 2(d) 2021/22 | Y |  |  |  |
|  | 2022 | IP | Report by the United Kingdom as Depositary Government for the Convention for the Conservation of Antarctic Seals (CCAS) in Accordance with Recommendation XIII-2, Paragraph 2(d) 2020/21 | Y |  |  |  |
|  | 1999 | WP | Liability | Y |  |  |  |
|  | 2002 | WP | Liabililty: informal contact group work on "operator", scope and actions for compensation | Y |  |  |  |

Invasive alien species and pathogens

| **Topic 20** | | | | | | | |
| --- | --- | --- | --- | --- | --- | --- | --- |
|  | Year | Document Type | Title | Substantively Related? | Retained Topic? | Notes |  |
| 1 | 1981 | SCI | Virological studies of Adelie Penguins (Pygoscelis adeliae) in Antarctica. | Y | Y | none |  |
| 2 | 2008 | SCI | Viral antibodies in south polar skuas around Davis Station, Antarctica | Y |  |  |  |
| 3 | 2023 | SCI | A Review of Pathogens Transmitted by the Container-Inhabiting Mosquitoes, Aedes Albopictus, A Global Public Health Threat | Y |  |  |  |
| 4 | 2020 | SCI | West Nile Virus (Flaviviridae) | Y |  |  |  |
| 5 | 1971 | SCI | Studies of respiratory viruses in personnel at an Antarctic base | Y |  |  |  |
| 6 | 1985 | SCI | Viral infections of little blue penguins (Eudyptula minor) along the southern coast of Australia. | Y |  |  |  |
| 7 | 2018 | SCI | Multi-year serological evaluation of three viral agents in the Adelie Penguin (Pygoscelis adeliae) on Ross Island, Antarctica | Y |  |  |  |
| 8 | 1980 | SCI | Serologic evidence for parainfluenzavirus infection during isolation at south pole station, Antarctica | Y |  |  |  |
| 9 | 1988 | SCI | Viruses in Macquarie Island birds | Y |  |  |  |
| 10 | 1981 | SCI | A survey of virus infection in Sub-Antarctic penguins on Macquarie Island, Southern Ocean | Y |  |  |  |
| **Topic 56** | | | | | | | |
|  | Year | Document Type | Title | Substantively Related? | Retained Topic? | Notes |  |
| 1 | 2018 | SCI | Seed germination and invasion success of Poa annua L. In Antarctica | Y | Y | n/A |  |
| 2 | 2010 | SCI | The establishment of a new ecological guild of pollinating insects on sub-Antarctic South Georgia | Y |  |  |  |
| 3 | 2010 | SCI | Impact of anthropogenic transportation to Antarctica on alien seed viability | Y |  |  |  |
| 4 | 2023 | SCI | The Trojan Horse strategy: Seed fungal endophyte symbiosis helps to explain the invasion success of the grass, Poa annua, in Maritime Antarctica | Y |  |  |  |
| 5 | 1981 | SCI | Larval development of Paralomis Ganulosa (Jaquinot) under laboratory conditions (Decapoda, Anomura, Lithodidae) | Y |  |  |  |
| 6 | 1971 | SCI | The Phyllosoma Larvae of Slipper Lobsters from the Hawaiian Islands and Adjacent Areas | Y |  |  |  |
| 7 | 1993 | SCI | Entrapment of long-distance transported pollen grains by various moss species in coastal Victoria Land, Antarctica | Y |  |  |  |
| 8 | 2007 | IP | Aliens in Antarctica | Y |  |  |  |
| 9 | 2011 | IP | Colonisation status of known non-native species in the Antarctic terrestrial environment (updated 2011) | Y |  |  |  |
| 10 | 1978 | SCI | Biology of Australian seagrasses: Pollen development and submarine pollination in Amphibolis antarctica and Thalassodendron ciliatum (Cymodoceaceae) | Y |  |  |  |

Pollution

| **Topic 25** | | | | | | |
| --- | --- | --- | --- | --- | --- | --- |
|  | Year | Document Type | Title | Substantively Related? | Retained Topic? | Notes |
| 1 | 2014 | SCI | Investigation of fuel reduction effect of the antarctic syowa base microgrid by introduction of local-supply-and-local-consumption energy | Y | Y | n/A |
| 2 | 1999 | IP | Waste Management at Syowa Station | Y |  |  |
| 3 | 2000 | SCI | Recent energy production at Japanese Antarctic stations | Y |  |  |
| 4 | 2020 | SCI | Study of a Clean Microgrid for the Japanese Antarctica Showa Base | Y |  |  |
| 5 | 2001 | IP | Planning of waste disposal at the Russian Antarctic stations and ships | Y |  |  |
| 6 | 2016 | SCI | Reducing fossil fuel consumption: Renewable energy in antarctica | Y |  |  |
| 7 | 2004 | IP | The removal and clean-up of abandoned British bases and waste dumps in Antarctica | Y |  |  |
| 8 | 1972 | IP | Post Conference tour to Antarctica | Y |  |  |
| 9 | 2015 | SCI | Reducing fossil fuel consumption: Renewable energy in antarctica | Y |  |  |
| 10 | 2004 | SCI | Incineration of waste at Casey Station, Australian Antarctic Territory | Y |  |  |
| **Topic 49** | | | | | | |
|  | Year | Document Type | Title | Substantively Related? | Retained Topic? | Notes |
| 1 | 2007 | SCI | Occurrence of organochlorine pollutants in the eggs and dropping-amended soil of Antarctic large animals and its ecological significance | Y | Y | n/A |
| 2 | 2023 | SCI | Levels of persistent organic pollutants (POPs) in the Antarctic atmosphere over time (1980 to 2021) and estimation of their atmospheric half-lives | Y |  |  |
| 3 | 2003 | SCI | Distribution of polychlorinated biphenyls (PCBs) and organochlorine pesticides in soils from the east Antarctic coast | Y |  |  |
| 4 | 2008 | SCI | Persistent organic pollutants in soils and sediments from James Ross Island, Antarctica | Y |  |  |
| 5 | 2014 | SCI | Fingerprint of persistent organic pollutants in tissues of Antarctic notothenioid fish | Y |  |  |
| 6 | 2018 | SCI | A 50-year retrospective of persistent organic pollutants in the fat and eggs of penguins of the Southern Ocean | Y |  |  |
| 7 | 2002 | SCI | Determination of organochlorine pesticides in ABALONE samples from antarctic | Y |  |  |
| 8 | 1994 | SCI | Pesticide Residues in the Sediments from the Lakes of Schirmacher Oasis, Antarctica | Y |  |  |
| 9 | 2011 | SCI | Spatial distribution of HCH and DDT in soil from the Wuyi Mountains southern, Fujian, Southeast China | Y |  |  |
| 10 | 1983 | SCI | DDTs and PCB isomers and congeners in Antarctic fish | Y |  |  |
| **Topic 89** | | | | | | |
|  | Year | Document Type | Title | Substantively Related? | Retained Topic? | Notes |
| 1 | 2017 | SCI | Red-pink pigmented hymenobacter coccineus sp. nov., hymenobacter lapidarius sp. nov. and hymenobacter glacialis sp. nov., isolated from rocks in Antarctica | Y | Y | All documents (except 1) discuss pollution to some degree (e.g., doc.1 discusses how the bacteria genus being researched can be found in polluted soils |
| 2 | 2022 | WP | The Implementation of the Climate Change Response Working Programme | N |  |  |
| 3 | 2023 | SCI | Microplastics in gastrointestinal tracts of gentoo penguin (Pygoscelis papua) chicks on King George Island, Antarctica | Y |  |  |
| 4 | 2018 | SCI | Turning microplastics into nanoplastics through digestive fragmentation by Antarctic krill | Y |  |  |
| 5 | 2016 | SCI | Rufibacter ruber sp. Nov., isolated from fragmentary rock | Y |  |  |
| 6 | 2023 | SCI | Evaluation of the Deterioration of Untreated Commercial Polystyrene by Psychrotrophic Antarctic Bacterium | Y |  |  |
| 7 | 2023 | SCI | Traits and distribution of microplastics in stomach and intestinal tract of Pleuragramma antarcticum around the South Shetland Islands | Y |  |  |
| 8 | 2023 | SCI | Quantifying microplastics in fjords along the Western Antarctic Peninsula | Y |  |  |
| 9 | 2022 | SCI | Current status and future perspectives of microplastic pollution in typical cryospheric regions | Y |  |  |
| 10 | 2020 | SCI | Biodeterioration of untreated polypropylene microplastic particles by antarctic bacteria | Y |  |  |

### Note 3.3. Topic selection for Internal Threats.

Functioning of the Antarctic Treaty System

| **Topic 13** | | | | | | |
| --- | --- | --- | --- | --- | --- | --- |
|  | Year | Document Type | Title | Substantively Related? | Retained Topic? | Notes |
| 1 | 2017 | IP | Ratification of Protocol on Environmental Protection to the Antarctic Treaty by Turkey | Y | Y | Related to the ratification and implementation of the Environmental Protocol, reflecting the legal mechanisms underpinning the ATS’s institutional function. |
| 2 | 2013 | IP | Norway’s Implementing Legislation: Annex VI of the Protocol on Environmental Protection to the Antarctic Treaty and Measure 4 (2004) | Y |  |  |
| 3 | 2022 | IP | Report of the Depositary Government of the Antarctic Treaty and its Protocol in accordance with Recommendation XIII-2 | Y |  |  |
| 4 | 2021 | IP | Report of the Depositary Government of the Antarctic Treaty and its Protocol in accordance with Recommendation XIII-2 | Y |  |  |
| 5 | 2006 | IP | Report of the Depositary Government of the Antarctic Treaty and its Protocol (USA) in accordance with Recommendation XIII-2 | Y |  |  |
| 6 | 1998 | IP | Report of the USA with respect to Article 13(1) of the Protocol on Environmental Protection to the Antarctic Treaty | Y |  |  |
| 7 | 2023 | IP | Report of the Depositary Government of the Antarctic Treaty and its Protocol in accordance with Recommendation XIII-2 | Y |  |  |
| 8 | 2005 | IP | Report of the depository government of the Antarctic Treaty and its Protocol (USA) in accordance with Recommendation XIII-2 | Y |  |  |
| 9 | 2018 | IP | Report of the Depositary Government of the Antarctic Treaty and its Protocol in accordance with Recommendation XIII-2 | Y |  |  |
| 10 | 2011 | IP | Report of the Depositary Government of the Antarctic Treaty and its Protocol in accordance with Recommendation XIII-2 | Y |  |  |
| **Topic 15** | | | | | | |
|  | Year | Document Type | Title | Substantively Related? | Retained Topic? | Notes |
| 1 | 1994 | SCI | The effect of low temperature on BOD in Antarctic seawater | N | N | Related to Paleoclimate and Earth system history research |
| 2 | 2005 | IP | ANDRILL – The Approved McMurdo Sound Portfolio Projects - Final CEE update | N |  |  |
| 3 | 1987 | SCI | Parmales, a new order of marine Chrysophytes, with descriptions of three new genera and seven new species | N |  |  |
| 4 | 1989 | SCI | The quantitative and qualitative use of the Adaptability Questionnaire (ADQ). | N |  |  |
| 5 | 2003 | SCI | The palaeoclimatic significance of Cenozoic marine macrofossil assemblages from Cape Roberts Project drillholes, McMurdo Sound, Victoria Land Basin, East Antarctica | N |  |  |
| 6 | 2016 | SCI | Whole-genome sequence of Rummeliibacillus stabekisii strain PP9 isolated from Antarctic soil | N |  |  |
| 7 | 2022 | SCI | A recursive matched-filter to systematically explore volcanic long-period earthquake swarms | N |  |  |
| 8 | 2014 | SCI | Early Miocene Mollusca from McMurdo Sound, Antarctica (ANDRILL 2A drill core), with a review of Antarctic Oligocene and Neogene Pectinidae (Bivalvia) | N |  |  |
| 9 | 2017 | SCI | Variable thermal loading and flexural uplift along the Transantarctic Mountains, Antarctica | N |  |  |
| 10 | 2001 | SCI | History of Oligocene erosion, uplift and unroofing of the Transantarctic Mountains deduced from sandstone detrital modes in CRP-3 drillcore, Victoria Land Basin, Antarctica | N |  |  |
| **Topic 30** | | | | | | |
|  | Year | Document Type | Title | Substantively Related? | Retained Topic? | Notes |
| 1 | 1961 | WP | Exchange of information concerning expeditions and stations | Y | N | This topic primarily contains procedural and operational reports related to expedition activities, station logistics, and Treaty-mandated inspections |
| 2 | 1972 | WP | Exchange of information | Y |  |  |
| 3 | 1991 | SCI | Cold climate building research at Wasa base, Antarctica | N |  |  |
| 4 | 2002 | SCI | Activities of the summer party of the 42nd Japanese Antarctic Research Expedition in 2000-2001 | N |  |  |
| 5 | 2006 | SCI | Georg von Neumayer Station (GvN) and Neumayer Station II (NM-II) German research stations on Ekström Ice Shelf, Antarctica | N |  |  |
| 6 | 2021 | IP | Australian Antarctic Treaty and Environmental Protocol inspections: January/February 2020 | Y |  |  |
| 7 | 2009 | IP | Inspection undertaken by Norway in accordance with Article VII of the Antarctic Treaty | Y |  |  |
| 8 | 1980 | SCI | Seismological Bulletin 1978: October, November, December. | N |  |  |
| 9 | 2002 | SCI | Activities of the summer party of the 38th Japanese Antarctic Research Expedition, 1996-1997 | N |  |  |
| 10 | 2008 | SCI | Activities of the summer season of the 48th Japanese Antarctic Research Expedition in 2006-2007 | N |  |  |
| **Topic 31** | | | | | | |
|  | Year | Document Type | Title | Substantively Related? | Retained Topic? | Notes |
| 1 | 2005 | IP | Annual List of any Initial Environmental Evaluations prepared in accordance with Annex I, Article 2, of the Protocol (Annex I, Article 6, lit. b, of the Protocol) and also ATCM Resolution 6 (1995) 2004/2005 Season | Y | N | Two topics present – environmental assessment procedures and astrophysics/neutrino science |
| 2 | 2005 | SCI | Exploring the high-energy neutrino universe from the south pole - Results from amanda and status of icecube | N |  |  |
| 3 | 1996 | IP | A Review of Environmental Impact Assessments (EIAs) prepared for proposed activities in Antarctica | Y |  |  |
| 4 | 2005 | SCI | Neutrino astronomy with IceCube and AMANDA | N |  |  |
| 5 | 2007 | IP | Final Comprehensive Environmental Evaluation (CEE) for the Proposed Construction and Operation of Halley VI Research Station, and the Demolition and Removal of Halley V Research Station, Brunt Ice Shelf, Caird Coast, Antarctica | Y |  |  |
| 6 | 2004 | SCI | Latest results of AMANDA | N |  |  |
| 7 | 2024 | WP | Improvement to the CEE review procedures | Y |  |  |
| 8 | 2005 | SCI | New results from the AMANDA Neutrino telescope | N |  |  |
| 9 | 2005 | SCI | New results from the antarctic muon and neutrino detector array | N |  |  |
| 10 | 1995 | IP | Comprehensive Environmental Evaluation (CEE) Follow-up Procedures | Y |  |  |
| **Topic 39** | | | | | | |
|  | Year | Document Type | Title | Substantively Related? | Retained Topic? | Notes |
| 1 | 1987 | IP | Status of Antarctic Treaty Consultative Meeting Recommendations | Y | Y | Centred on the effective functioning of the ATS |
| 2 | 1994 | IP | Operation of the Antarctic Treaty System | Y |  |  |
| 3 | 1977 | IP | Review of the implementation of Article IX-4 of the Treaty | Y |  |  |
| 4 | 1992 | IP | Status of ATCM recommendations | Y |  |  |
| 5 | 1991 | WP | Status of Antarctic Treaty recommendations | Y |  |  |
| 6 | 1991 | WP | Draft Recommendation | Y |  |  |
| 7 | 1970 | WP | Permits for Entry to Specially Protected Areas | Y |  |  |
| 8 | 1966 | WP | Replacement of Recommendation III-VII | Y |  |  |
| 9 | 1966 | WP | Interim Guide Line for Annexes to Agreed Measures for the Conservation of Antarctic Fauna and Flora | Y |  |  |
| 10 | 1964 | WP | Recommendation by Great Britain and the United States of America | Y |  |  |
| **Topic 41** | | | | | | |
|  | Year | Document Type | Title | Substantively Related? | Retained Topic? | Notes |
| 1 | 2012 | SCI | Improvement of communication condition from lunar rover to deep space tracking station in antarctic Great-Wall station | N | N | Information on the EIES is relevant but others are not |
| 2 | 2024 | WP | Redesign of the Electronic Information Exchange System (EIES) | Y |  |  |
| 3 | 1984 | SCI | Tritium in Antarctic precipitation - information on global distribution. | N |  |  |
| 4 | 2014 | WP | Reviewing information exchange requirements | Y |  |  |
| 5 | 1988 | SCI | Fatty acid and sterol composition of the antarctic amphipod Themisto gaudichaudii guerin 1828 | N |  |  |
| 6 | 1992 | SCI | Acoustic tracking system for monitoring under-ice movements of polar seals | N |  |  |
| 7 | 2021 | SCI | The role of sea spray in atmospheric ocean gas exchange | N |  |  |
| 8 | 2012 | WP | Improving the Functioning of the Electronic Information Exchange System (EIES) for Non-Governmental Activities in Antarctica (Updated version) | Y |  |  |
| 9 | 2010 | WP | Antarctic Treaty Information Exchange via the Electronic Information Exchange System (EIES): Current state and improvements for a consistent use | Y |  |  |
| 10 | 1982 | SCI | Results of a combined hydroacoustic and midwater trawling survey of the Prince Edward Island group. | N |  |  |
| **Topic 55** | | | | | | |
|  | Year | Document Type | Title | Substantively Related? | Retained Topic? | Notes |
| 1 | 1983 | IP | Rules of procedures | Y | Y | This topic relates to the procedural foundations of the ATS and reflects its efforts to formalise and maintain proper institutional function. |
| 2 | 1961 | WP | Rules of procedure | Y |  |  |
| 3 | 1987 | IP | Rules of procedure | Y |  |  |
| 4 | 1961 | IP | Rules of Procedure (Adopted) | Y |  |  |
| 5 | 2008 | SCI | Inter-division IX-X / Working group encouraging the international development of Antarctic Astronomy | N |  |  |
| 6 | 2004 | WP | Revisions to the rules of procedure of the Antarctic Treaty | Y |  |  |
| 7 | 1997 | WP | Rules of procedure for ATCMs: Corrected version | Y |  |  |
| 8 | 2003 | WP | Amended Rules of Procedure of Antarctic Treaty Consultative Meetings -Establishment of the Secretariat- | Y |  |  |
| 9 | 1966 | WP | Draft Recommendation submitted by Australia and the United States under item Status of Meetings of Experts | Y |  |  |
| 10 | 1966 | WP | Meeting of experts | Y |  |  |
| **Topic 71** | | | | | | |
|  | Year | Document Type | Title | Substantively Related? | Retained Topic? | Notes |
| 1 | 2005 | IP | Corrigendum of Final Report of 27 ATCM (2004) Pages 170-175: Rules of Procedure | Y | Y | This topic relates to the procedural foundations of the ATS and reflects its efforts to formalise and maintain proper institutional function. |
| 2 | 1961 | IP | Rules of Procedure (Adopted) | Y |  |  |
| 3 | 1961 | WP | Rules of procedure | Y |  |  |
| 4 | 1983 | IP | Rules of procedures | Y |  |  |
| 5 | 1987 | IP | Rules of procedure | Y |  |  |
| 6 | 2004 | WP | Amended rules of procedure of Antarctic Treaty Consultative Meetings - Establishment of the Secretariat | Y |  |  |
| 7 | 1997 | WP | Rules of procedure for ATCMs: Corrected version | Y |  |  |
| 8 | 2004 | WP | Revisions to the rules of procedure of the Antarctic Treaty | Y |  |  |
| 9 | 1996 | IP | Possible errors in the Rules of Procedure | Y |  |  |
| 10 | 1983 | IP | Satellite telecommunication at the Antarctic meteorological centre, Vicecomodoro Marambio | Y |  |  |
| **Topic 84** | | | | | | |
|  | Year | Document Type | Title | Substantively Related? | Retained Topic? | Notes |
| 1 | 2023 | WP | Proposal for an Intersessional Process to Review the Use and Maintenance of the Multi-Year Strategic Work Plan | Y | Y | This topic reflects the internal coordination and strategic planning mechanisms that support the ATS’s institutional functioning and policy development. |
| 2 | 2008 | WP | Preparation for Scheduled CEP Discussions: Reviews of Past Activities | Y |  |  |
| 3 | 2012 | WP | The Development of a Multi-Year Strategic Work Plan for the Antarctic Treaty Consultative Meeting | Y |  |  |
| 4 | 2024 | WP | Report on the Intersessional Contact Group (ICG) to review the use and functioning of the Multi-Year Strategic Work Plan (MYSWP) | Y |  |  |
| 5 | 2017 | WP | Informal Intersessional Discussion: Implementation of the Climate Change Response Work Programme | Y |  |  |
| 6 | 2008 | WP | Report on Effectiveness of Trial Informal Group | Y |  |  |
| 7 | 2023 | WP | CEP strategic priorities and the 5-year work plan: Outcomes and recommendations from the intersessional discussions and workshop | Y |  |  |
| 8 | 2011 | WP | Report of the Intersessional Contact Group on Non-Native Species 2010-2011 | Y |  |  |
| 9 | 2019 | WP | Subsidiary Group on Management Plans Report of activities during the intersessional period 2018-2019 | Y |  |  |
| 10 | 1999 | WP | Operation of Working Group II | Y |  |  |

Mineral and hydrocarbon exploitation

| **Topic 54** | | | | | | | |
| --- | --- | --- | --- | --- | --- | --- | --- |
|  | Year | Document Type | Title | Substantively Related? | Retained Topic? | Notes |  |
| 1 | 1983 | SCI | Geologic data and its impact on the discussion on a regime for mineral resources (Antarctica). | Y | Y | Some documents discuss geopolitics but are related to non-renewable resource exploitation |  |
| 2 | 1979 | SCI | Note on assessment of mineral resources in the Antarctic. | Y |  |  |  |
| 3 | 1994 | SCI | Fragile ecosystems: preclusive restoration in the Antarctic | Y |  |  |  |
| 4 | 1986 | SCI | Prospecting in Antarctica? Geological, economic and political aspects | Y |  |  |  |
| 5 | 1984 | SCI | Acceptability criteria of an Antarctic minerals regime (Criterios de aceptabilidad en un regimen para los minerales antárticos) | Y |  |  |  |
| 6 | 1981 | SCI | Antarctic regime and the law of the sea convention: an Australian view. | Y |  |  |  |
| 7 | 1977 | WP | Declaration of principles concerning exploration and exploitation of Antarctic mineral resources | Y |  |  |  |
| 8 | 1974 | SCI | The endangered sea | Y |  |  |  |
| 9 | 1978 | SCI | Antarctica: conflict or compromise. | Y |  |  |  |
| 10 | 1978 | SCI | Thaw in international law? Rights on Antarctica under the law of common spaces. | Y |  |  |  |

Bioprospecting

| **Topic 45** | | | | | | |
| --- | --- | --- | --- | --- | --- | --- |
|  | Year | Document Type | Title | Substantively Related? | Retained Topic? | Notes |
| 1 | 2017 | SCI | Complete genome of Arthrobacter alpinus strain R3.8, bioremediation potential unraveled with genomic analysis | Y | Y | All papers explore the potential commercial use of psychrophilic organisms or their biomolecules, including applications such as enzyme production, genetic engineering, or bioremediation. |
| 2 | 2018 | SCI | Purification, characterization, and cloning of a cold-adapted protease from antarctic Janthinobacterium lividum | Y |  |  |
| 3 | 2009 | SCI | Identification of the csp gene and molecular modelling of the CspA-like protein from Antarctic soil-dwelling psychrotrophic bacterium Psychrobacter sp. B6 | Y |  |  |
| 4 | 2017 | SCI | Bioprospecting for extracellular enzymes from culturable Actinobacteria from the South Shetland Islands, Antarctica | Y |  |  |
| 5 | 2022 | SCI | Biosynthetic potential of uncultured Antarctic soil bacteria revealed through long-read metagenomic sequencing | Y |  |  |
| 6 | 2017 | SCI | Complete genome sequence of Pseudomonas antarctica PAMC 27494, a bacteriocin-producing psychrophile isolated from Antarctica | Y |  |  |
| 7 | 2015 | WP | Molecular cloning and characterization of alpha - Galactosidase gene from Glaciozyma antarctica | Y |  |  |
| 8 | 2019 | SCI | Genetic Transformation of the Filamentous Fungus Pseudogymnoascus verrucosus of Antarctic Origin | Y |  |  |
| 9 | 2008 | SCI | Cloning of glyceraldehyde-3-phosphate dehydrogenase from an Antarctic psychrophilic bacterium by inverse and splinkerette PCR | Y |  |  |
| 10 | 2008 | SCI | Cloning and expression of lipP, a gene encoding a cold-adapted lipase from Moritella sp.2-5-10-1 | Y |  |  |
| **Topic 86** | | | | | | |
|  | Year | Document Type | Title | Substantively Related? | Retained Topic? | Notes |
| 1 | 1996 | SCI | Lipid and fatty acid composition of intestinal mucosa of two Antarctic teleosts | Y | Y | Most papers examine the lipid and fatty acid composition of Antarctic organisms, aligning with bioprospecting through their focus on biological materials with potential commercial value, such as marine oils and omega-3s. |
| 2 | 2011 | SCI | Comparison of lipids in euphasia pacific and antarctic krill | Y |  |  |
| 3 | 1977 | SCI | Lipid class and fatty acid composition of Chorismus antarcticus (Pfeffer) (Crustacea: Decapoda) at South Georgia | Y |  |  |
| 4 | 1992 | SCI | Positional analyses of triacylglycerol fatty acids in the milk fat of the antarctic fur seal (Arctocephalus gazella) | Y |  |  |
| 5 | 2007 | SCI | Lipid content and fatty acid composition of 15 marine fish species from the southeast coast of Brazil | Y |  |  |
| 6 | 1999 | SCI | Fatty acid composition of the adipose tissue and yolk lipids of a bird with a marine-based diet, the Emperor Penguin (Aptenodytes forsteri) | Y |  |  |
| 7 | 1998 | SCI | Oils rich in docosahexaenoic acid in livers of sharks from temperate Australian waters | Y |  |  |
| 8 | 2020 | SCI | Identification and characterization of Pseudozyma antarctica Î”12 fatty acid desaturase and its utilization for the production of polyunsaturated fatty acids | Y |  |  |
| 9 | 2020 | SCI | Temperature Differentially Affects Gene Expression in Antarctic Thraustochytrid Oblongichytrium sp. RT2316-13 | Y |  |  |
| 10 | 1999 | SCI | Lipid and fatty acid composition of the Antarctic krill Euphausia superba | Y |  |  |

Human activity impacts

| **Topic 9** | | | | | | |
| --- | --- | --- | --- | --- | --- | --- |
|  | Year | Document Type | Title | Substantively Related? | Retained Topic? | Notes |
| 1 | 1988 | SCI | Antarctic airways: Antarctica's first commercial airline | Y | Y | Describes the expansion and intensification of human presence and infrastructure in Antarctica, particularly through scientific operations, aviation logistics, and tourism activities |
| 2 | 2011 | SCI | The polar particle hunter | N |  |  |
| 3 | 2017 | SCI | Performance highlights of NASA super pressure balloon mid-latitude flights | N |  |  |
| 4 | 2009 | SCI | Duration flight of the NASA super pressure balloon | N |  |  |
| 5 | 2011 | SCI | The 2011 NASA ~422,400 m3 super pressure balloon test flight | N |  |  |
| 6 | 2019 | SCI | Supertiger-2 2018 flight payload recovery and preliminary instrument assessment | Y |  |  |
| 7 | 1999 | SCI | Disturbance of emperor penguin Aptenodytes forsteri chicks by helicopters | Y |  |  |
| 8 | 2018 | IP | IAATO Overview of Antarctic Tourism: 2017-18 Season and Preliminary Estimates for 2018-19 Season | Y |  |  |
| 9 | 2017 | IP | IAATO Overview of Antarctic Tourism: 2016-17 Season and Preliminary Estimates for 2017-18 | Y |  |  |
| 10 | 2015 | SCI | Qualification of the NASA super pressure balloon | N |  |  |
| **Topic 16** | | | | | | |
|  | Year | Document Type | Title | Substantively Related? | Retained Topic? | Notes |
| 1 | 1992 | WP | Draft recommendation on the conduct of tourism and non-governmental activity in the Antarctic Treaty Area | Y | Y | Topic focuses on the environmental impacts and governance of tourism and non-governmental activity in Antarctica |
| 2 | 1994 | WP | Conduct of tourism and non-governmental activity in Antarctica | Y |  |  |
| 3 | 2003 | Scientific | Environmental impact management of ship based tourism to Antarctica | Y |  |  |
| 4 | 2004 | WP | Insurance cover for the costs of search and rescue, medical care and evacuation from Antarctica in the case of tourist and other non governmental activities | Y |  |  |
| 5 | 1988 | Scientific | The growth of Antarctic tourism | Y |  |  |
| 6 | 2002 | IP | ATCM papers, discussions and recommendations relating to tourism and non-governmental activities | Y |  |  |
| 7 | 2012 | IP | Environmental Aspects and Impacts of Tourism and Non-governmental Activities in Antarctica | Y |  |  |
| 8 | 1992 | Scientific | Antarctic tourism and environmental concerns | Y |  |  |
| 9 | 2016 | Scientific | Destination icy wilderness tourism in antarctica | Y |  |  |
| 10 | 2016 | WP | Benefits of Communication Among Competent Authorities for Tourism and Non-governmental Activities | Y |  |  |
| **Topic 28** | | | | | | |
|  | Year | Document Type | Title | Substantively Related? | Retained Topic? | Notes |
| 1 | 2013 | SCI | IMO regulations and ice class rules | Y | Y | Topic addresses international efforts to regulate ship-based activity in Antarctica |
| 2 | 2008 | IP | Rules governing ships in the Antarctic | Y |  |  |
| 3 | 2014 | IP | Status report on the development of the International Code for ships operating in Polar Waters (Polar Code) | Y |  |  |
| 4 | 2014 | WP | Supporting the Continued Development of the Polar Code | Y |  |  |
| 5 | 2014 | SCI | Progress towards the mandatory code for polar shipping | Y |  |  |
| 6 | 2017 | IP | Managing non-SOLAS vessels in the Southern Ocean | Y |  |  |
| 7 | 1998 | WP | The International Code of Safety for Ships in Polar Waters (Polar Code) | Y |  |  |
| 8 | 2011 | IP | Developing a Mandatory Polar Code “Progress and Gaps” | Y |  |  |
| 9 | 1970 | SCI | Linblad explorer. Cruise vessel for Antarctic voyages. | Y |  |  |
| 10 | 2011 | IP | Working group on the development of a mandatory code for ships operating in polar waters, IMO | Y |  |  |
| **Topic 43** | | | | | | |
|  | Year | Document Type | Title | Substantively Related? | Retained Topic? | Notes |
| 1 | 2016 | SCI | Installation of neutron monitor at the jang bogo station in antarctica | N | N | This topic primarily comprises engineering and technical development studies related to station infrastructure and UAV systems |
| 2 | 2017 | SCI | Effects of wind fences on the wind environment around Jang Bogo Antarctic Research Station | Y |  |  |
| 3 | 2011 | IP | The Draft Comprehensive Environmental Evaluation for the construction and operation of the Jang Bogo Antarctic Research Station, Terra Nova Bay, Antarctica | Y |  |  |
| 4 | 2021 | SCI | Development of Balloon-Assisted Gliding Unmanned Aerial Vehicle System for Atmospheric Observation and Spatiotemporal Aerosol Variations in Summer Troposphere over Syowa Station | N |  |  |
| 5 | 1972 | IP | List of delegates | N |  |  |
| 6 | 2013 | Scientific | Development and Operational Experiences of UAVs for Scientific Research in Antarctica | N |  |  |
| 7 | 2011 | WP | The Draft Comprehensive Environmental Evaluation for the construction and operation of the Jang Bogo Antarctic Research Station, Terra Nova Bay, Antarctica | Y |  |  |
| 8 | 2022 | SCI | Design and analysis of morphing wing UAV adopted to harsh environment based on 'Frigate bird' | N |  |  |
| 9 | 2019 | SCI | Targeted selection of amino acid residues to create variant libraries of Glaciozyma antarctica proline iminopeptidase | N |  |  |
| 10 | 1970 | IP | Personal History of Mr. Hiroshi Yokota, Secretary-General for the Sixth Antarctic Treaty Consultative Meeting | N |  |  |
| **Topic 46** | | | | | | |
|  | Year | Document Type | Title | Substantively Related? | Retained Topic? | Notes |
| 1 | 2015 | WP | Five-Year Work Plan adopted at the 17th meeting of the Committee for Environmental Protection (CEP XVII) | Y | Y | Topic describes strategic environmental planning and policy development by the CEP to manage and mitigate human impacts in Antarctica |
| 2 | 2019 | IP | Footprint in Antarctica | Y |  |  |
| 3 | 1982 | SCI | Environmental aspects of potential petroleum exploration and exploitation in Antarctica: forecasting and evaluating risks. | Y |  |  |
| 4 | 2022 | WP | Revisiting CEP strategic priorities and the CEP Five-Year Work Plan | Y |  |  |
| 5 | 2014 | WP | CEP Five-Year Work Plan adopted at the XVIth CEP Meeting in Brussels | Y |  |  |
| 6 | 2013 | WP | CEP Five-Year Work Plan adopted at the XVth CEP Meeting at Hobart | Y |  |  |
| 7 | 2020 | SCI | Human Error in Autonomous Underwater Vehicle Deployment: A System Dynamics Approach | Y |  |  |
| 8 | 1999 | WP | CEEs and the CEP | Y |  |  |
| 9 | 2018 | WP | Supporting the work of the Committee for Environmental Protection (CEP): A paper by the CEP Chair | Y |  |  |
| 10 | 2007 | WP | A Five-Year Work plan for the CEP: Report of the Intersessional Contact Group | Y |  |  |
| **Topic 59** | | | | | | |
|  | Year | Document Type | Title | Substantively Related? | Retained Topic? | Notes |
| 1 | 2016 | SCI | Design and antarctic testing of the Icefin vehicle | Y | Y | Topic reflects the operational use and environmental regulation of emerging technologies in Antarctica, including RPAS and vehicle cleaning protocols to minimise human impacts |
| 2 | 2009 | SCI | Biogeneration of chromophoric dissolved organic matter by bacteria and krill in the southern ocean | N |  |  |
| 3 | 2009 | WP | Procedures for vehicle cleaning to prevent transfer of non-native species into and around Antarctica | Y |  |  |
| 4 | 2023 | IP | Scientific use of Remotely Piloted Aircraft Systems (RPAS) in Antarctica: a review | Y |  |  |
| 5 | 2024 | IP | Operations and Scientific use of Remotely Piloted Aircraft Systems (RPAS) in Antarctica: a review | Y |  |  |
| 6 | 2010 | WP | Draft procedures for vehicle cleaning to prevent transfer of non-native species into and around Antarctica | Y |  |  |
| 7 | 2019 | SCI | Environmental guidelines for operation of Remotely Piloted Aircraft Systems (RPAS): Experience from Antarctica | Y |  |  |
| 8 | 2022 | WP | Do the Environmental Guidelines for operation of Remotely Piloted Air-craft Systems (RPAS) in Antarctica (v 1.1) need to be revised? | Y |  |  |
| 9 | 2019 | SCI | Icefin: Redesign and 2017 Antarctic Field Deployment | Y |  |  |
| 10 | 2009 | SCI | Biogeochemical and hydrographic controls on chromophoric dissolved organic matter distribution in the Pacific Ocean | N |  |  |
| **Topic 65** | | | | | | |
|  | Year | Document Type | Title | Substantively Related? | Retained Topic? | Notes |
| 1 | 2013 | IP | IAATO Overview of Antarctic Tourism: 2012-13 Season and Preliminary Estimates for 2013-14 Season | Y | Y | Topic provides detailed annual reporting on Antarctic tourism activity, a central component of human presence and impact in the region |
| 2 | 2016 | IP | IAATO Overview of Antarctic Tourism: 2015-16 Season and Preliminary Estimates for 2016-17 | Y |  |  |
| 3 | 2015 | IP | IAATO Overview of Antarctic Tourism | Y |  |  |
| 4 | 2012 | IP | IAATO Overview of Antarctic Tourism: 2011-12 Season and Preliminary Estimates for 2012-13 Season | Y |  |  |
| 5 | 2009 | IP | IAATO Overview of Antarctic Tourism: 2008-2009 Antarctic Season and Preliminary Estimates for 2009-2010 Antarctic Season | Y |  |  |
| 6 | 2014 | IP | IAATO Overview of Antarctic Tourism: 2013-14 Season and Preliminary Estimates for 2014-15 Season | Y |  |  |
| 7 | 2011 | IP | IAATO Overview of Antarctic Tourism: 2010-11 Season and Preliminary Estimates for 2011-12 Season | Y |  |  |
| 8 | 2010 | IP | IAATO Overview of Antarctic Tourism: 2009-10 Season and Preliminary Estimates for 2010-11 and Beyond | Y |  |  |
| 9 | 2008 | IP | IAATO Overview of Antarctic Tourism 2007-2008 Antarctic Season and Preliminary Estimates for 2008-2009 Antarctic Season | Y |  |  |
| 10 | 2007 | IP | IAATO Overview of Antarctic Tourism 2006-2007 Antarctic Season | Y |  |  |
| **Topic 68** | | | | | | |
|  | Year | Document Type | Title | Substantively Related? | Retained Topic? | Notes |
| 1 | 2012 | SCI | Edward Adrian Wilson (1872-1912): Polar explorer and artist | Y | Y | topic addresses the legacy and management of historical human sites in Antarctica |
| 2 | 2014 | SCI | The dogs of the Australasian Antarctic Expedition 1911-1914 | Y |  |  |
| 3 | 1968 | WP | Draft Recommendation: Historic Monuments | Y |  |  |
| 4 | 1972 | WP | Historic monuments | Y |  |  |
| 5 | 1972 | WP | Historic monuments | Y |  |  |
| 6 | 1970 | WP | Amalgamated List of Historic Monuments in Antarctica | Y |  |  |
| 7 | 1972 | WP | Historic monuments | Y |  |  |
| 8 | 2000 | SCI | Centennial of historic huts in Antarctica: A tourism attraction | Y |  |  |
| 9 | 2003 | SCI | Travels with mules: Antarctica 1912 | Y |  |  |
| 10 | 1968 | WP | New Zealand Proposal Regarding Historical Vestiges | Y |  |  |
| **Topic 77** | | | | | | |
|  | Year | Document Type | Title | Substantively Related? | Retained Topic? | Notes |
| 1 | 1992 | IP | Status of signatures and ratification of the Protocol on Environmental Protection to the Antarctic Treaty | Y | Y | Topic reflects legal and institutional mechanisms for tracking and managing human activity in Antarctica, particularly tourism flows and regulatory oversight. |
| 2 | 1999 | IP | Report by the Head of Delegation of Australia as the Representative of the Depositary Government for the Convention for the Conservation of Antarctic Marine Living Resources (CCAMLR) | Y |  |  |
| 3 | 1994 | IP | Signatures and Ratification of Protocol on Environmental Protection to the Antarctic Treaty | Y |  |  |
| 4 | 1997 | IP | Current status of adherence to the CCAMLR Convention and membership of the Commission | Y |  |  |
| 5 | 1996 | IP | Report submitted by the depositary government of the Convention on the Conservation of Antarctic Marine Living Resources (Australia) | Y |  |  |
| 6 | 1994 | IP | Convention on the Conservation of Antarctic Marine Living Resources (CCAMLR) | Y |  |  |
| 7 | 1977 | IP | Annex C of Recommendation VIII-9: Matters to be reported by tour organizers | Y |  |  |
| 8 | 1970 | IP | Provisional Schedule of Receptions, etc. during the Session of the Sixth Antarctic Treaty Consultative Meeting | N |  |  |
| 9 | 2003 | IP | Report on Antarctic tourism numbers through the Port of Ushuaia (2002-2003 Season) | Y |  |  |
| 10 | 2014 | IP | Preliminary report on Antarctic tourist flows and cruise ships operating in Ushuaia during the 2013/2014 Austral summer season | Y |  |  |

## Supplementary Note 4. Validation of the topic modelling pipeline.

To evaluate the robustness of the analytical framework, we conducted five complementary validation and diagnostic analyses spanning corpus construction, topic modelling, classification performance, and temporal inference (**Supplementary Figure 5**). Automated text analyses can be sensitive to modelling assumptions, preprocessing decisions, and parameter specification, making application-specific validation important when drawing substantive inferences from large corpora ^86,87^ Accordingly, these analyses assessed: (i) the precision of the scientific corpus retrieval strategy; (ii) the influence of imbalance between scientific and policy documents on inferred topic structure; (iii) the accuracy of topic-to-threat classifications; (iv) the sensitivity of topic structure to alternative vocabulary filtering strategies; and (v) the sensitivity of temporal dynamics to alternative model parameterisations.

**Supplementary Figure 5.** Distribution of robustness analyses throughout the topic modelling pipeline. The workflow illustrates the progression from corpus compilation through topic modelling, topic-to-threat assignment, and modelling of temporal dynamics. Five validation and diagnostic analyses were conducted across the pipeline.

To summarise, the validation results supported the reliability of the framework while also identifying important analytical constraints. The scientific corpus retrieval strategy achieved high precision (88%) and outperformed alternative approaches commonly used in Antarctic bibliometric research e.g., see ^55,88,89,90^. Despite substantial differences in the size of the scientific and policy corpora, we found little evidence that this imbalance systematically biased inferred topic structure, either across the full model or within the subset of conservation threats examined in this study. Vocabulary filtering analyses further identified a stable trade-off between semantic coherence and vocabulary retention, where increasingly restrictive filtering improved topic coherence but progressively removed larger numbers of meaningful terms. This supported selection of the 15–85% vocabulary threshold as a balanced configuration that improved interpretability while retaining nuanced domain-specific language.

Topic-to-threat classifications also showed good agreement with manually classified documents (macro-averaged F1 = 83.3%), consistent with strong performance for complex thematic classification tasks involving overlapping concepts ^91^. Importantly, this validation imposed stricter classification boundaries than the primary probabilistic framework by requiring each document to be assigned to a single dominant threat category, likely producing more conservative performance estimates overall. Finally, the major temporal patterns identified in the analysis remained robust to reasonable changes in model parameterisation, with alternative hyperparameter configurations preserving historically interpretable periods of change. Spline magnitudes were, however, sensitive to parameter specification and so exact spline estimates should therefore be interpreted more cautiously than broader shifts in attention through time.

Complete methodological details and validation results are provided in the following subsections. Associated validation datasets and analytical code are archived within the study’s data repository (see the Data Availability Statement in the Main Text for details).

### Note 4.1. Corpus retrieval validation.

#### 4.1.1 Methods

The scientific corpus was constructed using a keyword-based bibliometric retrieval strategy and therefore may include documents that fall outside the intended scope of the analysis. We therefore evaluated whether the retrieval approach produced a corpus that was sufficiently relevant to support subsequent analyses. Because the scientific literature could not be exhaustively retrieved a priori, corpus construction required a retrieval strategy capable of balancing coverage and relevance. The policy corpus, by contrast, consisted of all available ATCM Working Papers and Information Papers retrieved directly from the Antarctic Treaty Secretariat and therefore did not require keyword-based retrieval.

The Antarctic bibliometric literature contains two dominant approaches to corpus construction that balance coverage and relevance in different ways (**Supplementary Table 5**). The first employs targeted keyword strategies that combine “antarct*” with region-specific terms and exclusion criteria to improve specificity e.g., ^92,93^. The second adopts a more generalist approach, relying solely on the wildcard term “antarct*” to maximise coverage of Antarctic-associated publications e.g., ^55,88,89,90^.

**Supplementary Table 5**. Comparison of bibliometric search strategies used to identify Antarctic scientific literature.

| Bibliometric approach | Keyword search^a^ | References |
| --- | --- | --- |
| 1  (targeted keyword approach) | (TITLE-ABS-KEY(antarct* OR "southern ocean" OR "ross sea" OR "amundsen sea" OR "weddell sea")) AND (PUBYEAR > 1960) AND (PUBYEAR < 2024) AND NOT (TITLE-ABS-KEY(candida OR "except antarctica" OR "not antarctica")) AND ( LIMIT-TO ( LANGUAGE,"English" ) ) | Gray (2019); Gray and Hughes (2016); Hughes et al. (2024) |
| 2  (wildcard-only approach) | (TITLE-ABS-KEY(antarct* )) AND (PUBYEAR > 1960) AND (PUBYEAR < 2024) AND ( LIMIT-TO ( LANGUAGE,"English" ) ) | Dastidar (2007); Dastidar and Ramachandran (2008); Dudeney and Walton (2012); Ji et al. (2014) |

^a^Adjusted for the timeframe of this study and for English-language documents only

These approaches represent two widely used definitions of “Antarctic literature”. Rather than constructing an additional search strategy for this work, we compared these established retrieval approaches directly and chose the better of the two. Because our objective was to define a corpus that is comprehensive, transparent, and reproducible, we treated corpus construction as an explicit methodological decision and evaluated each approach empirically.

To determine which approach provided the most suitable corpus for our analysis, we quantified the precision of each retrieval strategy through manual validation of randomly sampled abstracts. Sample size was determined a priori to estimate classification precision within ±5% at 95% confidence under a binomial framework ^94^, using:

$$n=\frac{Z^{2}p(1-p)}{E^{2}}$$

Where Z is the critical value associated with a 95% confidence level (Z = 1.96), E is the desired precision (E = 0.05, ±5 percentage points), and p is the expected proportion of documents classified as relevant (p = 0.5). In the absence of prior information, p = 0.5 was used because it maximises the required sample size and therefore provides the most conservative sample size estimate. This yielded a target sample size of 384 abstracts. Given the large size of the corpus relative to the validation sample, finite population correction was not applied ^94^.

Abstracts were randomly sampled from each retrieval strategy and independently classified by two reviewers using a predefined relevance schema. Relevance criteria were defined a priori to ensure consistent and repeatable classification, drawing on established principles from systematic review methodology in conservation science see ^95^. Here, documents were considered relevant if Antarctica constituted a primary focus of the study and the research questions and conclusions were substantively centred on the Antarctic region, irrespective of disciplinary focus. Documents were classified as irrelevant if Antarctica was mentioned only incidentally, used for comparison, or if the study did not directly engage with Antarctic systems or processes. In cases of ambiguity, reviewers assessed whether the study’s primary research questions and conclusions were centred on the Antarctic region.

To verify that the relevance criteria could be applied consistently, inter-reviewer agreement was assessed using Cohen's kappa ^96^. Disagreements were subsequently reviewed and resolved through discussion between the reviewers, and the resulting consensus classifications were used to calculate the final precision estimates. Agreement was high for both the targeted keyword approach (κ = 0.87) and the wildcard-only approach (κ = 0.92), indicating that the relevance criteria were applied consistently ^97^.

The final consensus classifications were used to calculate precision for each retrieval strategy:

$$Precision= \frac{TP}{TP + FP}$$

where TP is the number of abstracts correctly classified as relevant (true positives) and FP is the number incorrectly classified as relevant (false positives). This metric quantifies the proportion of retrieved documents that fall within the scope of the analysis. Confidence intervals for precision were calculated using the Wilson method ^98^.

#### 4.1.2 Interpretation

The targeted keyword approach achieved substantially higher precision (88%, 95% confidence interval [CI]: 85–91%) than the wildcard-only approach (79%, 95% CI: 75–83%), indicating that broader retrieval increases the proportion of irrelevant documents (**Supplementary Table 6**). On this basis, the targeted keyword approach was selected to construct the corpus used in our study.

**Supplementary Table 6**. Performance of bibliometric search strategies based on manual validation of document relevance.

| Bibliometric approach | True Positive (TP) | False Positive (FP) | Precision (95% CI) |
| --- | --- | --- | --- |
| 1 (targeted keyword approach) | 338 | 46 | 88 (85 – 91) % |
| 2 (wildcard-only approach) | 303 | 80 | 79 (75 – 83) % |

To provide additional transparency, we examined the nature of classification errors to characterise the limitations of the retrieval strategy through manual inspection of all identified false positive records.

False positives were not randomly distributed, but instead clustered within seven recurring categories (**Supplementary Table 7**). The largest proportion arose from studies in which Antarctica was included as a comparative reference (32.6%), followed by incidental lexical matches and studies focused on regions physically connected to Antarctica without being centred on the Antarctic region itself (both 19.6%). These categories capture cases in which Antarctic terminology is present but not central to the study, either through comparison, incidental mention, or broader regional context. Smaller contributions arose from studies in which Antarctica formed one component of broader multi-region or global analyses, rather than the primary focus of investigation (8.7%). Additional false positives arose from indexing or lexical artefacts (8.7%), references to Antarctic logistical infrastructure or field operations (6.5%), and studies using Antarctic-derived samples without substantively engaging with Antarctic systems (4.3%). Excluding indexing or lexical artefacts, which represent genuine retrieval mismatches (n = 4, 9%), the overwhelming majority of false positives (n = 42, 91%) reflect boundary cases in which Antarctic relevance is present but not primary.

**Supplementary Table 7**. Classification of false positive documents by error type and frequency for the targeted keyword search strategy.

| Category | Classification criteria | Error count | % of false positive | % of validation sample  (n = 384) |
| --- | --- | --- | --- | --- |
| Comparative reference | Studies in which Antarctica is mentioned as a point of comparison, analogy, or contextual reference, but is not central to the research question, analysis, or conclusions. | 15 | 32.6 | 3.9 |
| Incidental lexical match | Records in which Antarctic-related terms are present in the title, abstract, or metadata but do not reflect substantive engagement with Antarctic systems, processes, or contexts. | 9 | 19.6 | 2.3 |
| Oceanographic / geographic spillover | Studies focused on regions adjacent to, or dynamically connected with, Antarctica where Antarctic terminology is used in a broader geographic or oceanographic context without the study being centred on the Antarctic region itself. | 9 | 19.6 | 2.3 |
| Multi-system inclusion | Studies that include Antarctica as one component within a broader, multi-region or global analysis, where Antarctica contributes to the analysis but is not the primary focus. | 4 | 8.7 | 1.0 |
| Indexing / lexical artefact | Records retrieved due to artefacts of indexing, terminology, or naming conventions, including algorithm names, taxonomic labels, or ambiguous keyword combinations (e.g. “Arctic/Antarctic” terms). | 4 | 8.7 | 1.0 |
| Logistical presence | Studies in which Antarctica is referenced as a site of logistical activity (e.g. research stations, field sites, or launch locations) or operational infrastructure, without the scientific investigation being directed at Antarctic systems or processes. | 3 | 6.5 | 0.8 |
| Sample provenance | Studies in which materials, organisms, or samples originate from Antarctica, but the research question, analysis, or interpretation is not focused on Antarctic systems | 2 | 4.3 | 0.5 |

### Note 4.2. Topic composition analysis.

#### 4.2.1 Methods

In our study, we combined the scientific and policy literature into a single corpus so that topics were inferred within a common semantic space. Because LDA derives topics from corpus-specific word co-occurrence structure, fitting separate models can produce topic sets that are not inherently aligned, limiting direct comparison of thematic emphasis across corpora ^99^. The unified corpus therefore enabled direct comparison between scientific and policy documents. However, the combined corpus was strongly imbalanced, comprising 5,924 ATCM policy documents and 85,096 scientific abstracts (approximately 1:14). Since LDA infers topics from aggregate word co-occurrence patterns, the dominant corpus may exert disproportionate influence on the resulting topic structure.

To assess whether this imbalance influenced the inferred topics, we compared topic proportions between scientific and policy documents. For each topic, we calculated the average document-level topic proportion separately for the scientific and policy corpora. In LDA, each document is assigned a proportion for every topic, representing how strongly each topic is reflected in the text. Averaging these values across documents therefore provides a measure of topic representation that is independent of the total number of documents within a corpus. For example, a topic with an average proportion of 0.10 across scientific abstracts and 0.05 across policy documents would be interpreted as having greater relative representation in the scientific corpus despite the substantially larger number of scientific documents overall.

Differences in mean proportions were then calculated for each topic and evaluated using a paired Wilcoxon signed-rank test to assess whether one corpus consistently exhibited higher representation across topics ^100^. If corpus imbalance were biasing the model, topics would be expected to show systematically higher representation in the larger corpus.

#### 4.2.2 Interpretation

Scientific documents exhibited higher mean topic proportions in 51 topics (56.7%), while policy documents exhibited higher proportions in 39 topics (43.3%) (**Supplementary Figure 6**). Despite this imbalance in counts, differences in topic representation were not statistically significant (paired Wilcoxon signed-rank test, p = 0.99) and were negligible in magnitude (r = 0.001) (**Supplementary Figure 7a**). This indicates no consistent directional bias in topic representation between corpora and provides little evidence that corpus imbalance resulted in a systematic bias towards the scientific literature. Repeating this analysis only for the subset of topics included in the conservation threat framework yielded the same result, with no significant differences in topic proportions between scientific and policy documents (p = 0.29) (**Supplementary Figure 7b**). This provides further evidence that the subset of topics underpinning the study's conclusions is not disproportionately dominated by the scientific corpus.

**Supplementary Figure 6**. Comparison of mean topic loading scores between scientific and ATCM policy documents across all model-derived topics. Bars represent the mean loading score for each topic, calculated separately for scientific (green) and policy (purple) documents. Dots indicate topics that were included in the main analysis.

**Supplementary Figure 7**. Distribution of mean topic loading scores across scientific and policy documents for all topics (**a**) and for the subset of topics included in the conservation threat framework (**b**). Each point represents the mean proportions for a single topic, with violin plots showing the overall distribution and embedded boxplots indicating median and interquartile range.

### Note 4.3. Accuracy of topic-to-threat classification.

#### 4.3.1 Methods

In the main analysis, LDA topics were assigned to conservation threats through structured thematic review and team consensus (see the Methods section of the main text and Supplementary Information 3 for complete detail). This process produced a mapping between LDA topics and conservation threat categories, whereby each topic was assigned either to a conservation threat or to a residual category of “no conservation threat”. Because subsequent analyses relied on these assignments to infer conservation threats from topic-model outputs, we evaluated their consistency by comparing manually classified conservation threat classifications with model-derived classifications. To do so, a reference dataset was manually classified by independent reviewers who were blinded to the model-derived classifications, and agreement between manual and model-derived classifications was subsequently evaluated.

To operationalise this comparison, documents were assigned a model-derived conservation threat classification based on their dominant LDA topic (i.e., the topic with the highest probability). Documents inherited the threat classification associated with their dominant topic, whereas documents whose dominant topic was not assigned to a conservation threat were classified as “no conservation threat”. This produced a single model-derived classification for each document. If the topic-to-threat mapping is internally consistent, documents assigned to a particular conservation threat by the topic model should be classified into the same threat category during manual review.

We constructed a manually classified reference dataset by randomly sampling documents from those assigned to each conservation threat based on the model-derived classification, as well as documents classified as “no conservation threat”. For each category, we selected 40 documents from both the scientific and policy corpora. Under a binomial sampling framework, this sample size corresponds to an approximate margin of error of ±15 percentage points at the 95% confidence level (see Supplementary Note 4.1.1 for complete details). This level of precision was considered sufficient for evaluating the consistency of the topic-to-threat mapping while remaining feasible for manual review. Where fewer than 40 documents were available for a given category, all available documents were included (**Supplementary Table 8**).

**Supplementary Table 8**. Validation sample sizes by conservation threat and document type.

| Conservation threat | Number of sampled documents | |
| --- | --- | --- |
|  | Scientific | Policy (WP + IP) |
| Climate change | 40 | 40 |
| Ocean acidification | 40 | 1 |
| Socio-geopolitical disruptions | 40 | 40 |
| Invasive alien species and disease | 40 | 25 |
| Pollution | 40 | 40 |
| Functioning of the Antarctic governance regime | 40 | 40 |
| Mineral and hydrocarbon exploitation | 40 | 40 |
| Bioprospecting | 40 | 19 |
| Human activity impacts | 40 | 40 |
| No conservation threat | 40 | 40 |

Each document in the validation sample was reviewed and assigned a conservation threat classification based on its content, using abstracts for scientific papers and full texts for ATCM policy documents, following the classification criteria outlined in **Supplementary Table 9**. Two reviewers independently classified each document, blinded to the model-derived classifications, with disagreements resolved by a third reviewer acting as an adjudicator, consistent with the procedure used in the relevance validation (**Supplementary Note 4.1.1**). Inter-reviewer agreement between the primary reviewers was assessed using Cohen’s κ to evaluate consistency in the application of the classification criteria ^97^. Agreement was high (κ = 0.91). Disagreements were subsequently reviewed and resolved through discussion between the reviewers, and the consensus classifications were treated as the reference standard against which model-derived classifications were evaluated.

To evaluate classification performance, we compared model-derived classifications with the manually classified reference dataset using standard multi-class classification metrics, including precision, recall, and F1-score ^91,101^. Precision was calculated as the proportion of documents assigned to a conservation threat by the topic model that received the same classification in the manually classified reference dataset (see **Supplementary Note 4.1.1** for details). Recall was defined as the proportion of documents assigned to a given conservation threat in the manually classified reference dataset that received the same classification from the topic model:

$$Recall = \frac{TP}{TP + FN}$$

where TP is the number of documents assigned to a given conservation threat in the manually classified reference dataset that received the same classification from the topic model; FN is the number of documents assigned to that conservation threat in the manually classified reference dataset that received a different classification from the topic model. Precision therefore quantifies how often conservation threat classifications assigned by the topic model agreed with the manually classified reference dataset, whereas recall measures how many documents assigned to a given conservation threat in the manually classified reference dataset received the same classification from the topic model.

The F1-score, representing the harmonic mean of precision and recall, was used as the primary summary metric of classification performance:

$$F1 = 2 \times\frac{Precision \times Recall}{Precision + Recall}$$

Performance was assessed separately for the scientific and policy corpora, as well as for the combined dataset. Metrics were computed for each conservation threat category and summarised using macro-averaged values ^102,103^, such that each category contributed equally to the overall performance regardless of the number of documents assigned to that category.

**Supplementary Table 9**. Classification framework for Antarctic conservation threats.

| Conservation threat | Operational definition | Include if… | Exclude if… | Ambiguity rules |
| --- | --- | --- | --- | --- |
| Climate change  (merged terrestrial + marine) | Anthropogenic climate forcing driving physical and/or ecological change in Antarctic systems | - Analyses atmospheric or ocean warming - Examines sea ice extent, thickness, or variability - Examines glacier, ice shelf, or ice sheet dynamics - Assesses ecological responses explicitly linked to climate change - Uses climate models, projections, or scenarios - Explicitly attributes observed or projected changes to climate change or anthropogenic climate forcing | - Focuses primarily on ocean chemistry changes without broader climate context - Examines direct human activities or local disturbances - Describes environmental variability (seasonal, interannual, or decadal) without explicit attribution to anthropogenic climate forcing - Presents physical or climatological analyses without analysing climate change as a driver or assessing ecological/conservation implications - Mentions climate change or warming only as background context or a secondary explanation | Assign only if climate is a primary driver, not background context; resolve overlap with acidification based on analytical focus; exclude superficial mentions |
| Ocean acidification | Changes in ocean carbonate chemistry driven by anthropogenic CO₂ uptake, resulting in reduced pH and altered carbonate system dynamics with implications for marine organisms and ecosystems | - Analyses ocean pH, carbonate chemistry, or CO₂ uptake processes - Examines aragonite or calcite saturation states - Investigates biological or ecological responses to changing carbonate chemistry - Uses experiments, observations, or models explicitly focused on acidification processes - Attributes chemical or biological changes to ocean acidification or CO₂-driven shifts in carbonate chemistry - Examines interactions between acidification and marine organisms (e.g. calcifiers, plankton, benthic species) | - Focuses primarily on ocean warming, sea ice, or physical climate processes without analysing carbonate chemistry - Examines climate change broadly without specific treatment of acidification mechanisms - Mentions acidification only as background context or a secondary co-stressor - Analyses marine ecosystem change without explicitly linking outcomes to carbonate chemistry - Describes general ocean biogeochemistry without clear focus on pH or carbonate system dynamics | Assign only if carbonate chemistry change is a primary analytical focus, not a secondary co-stressor; where both warming and acidification are analysed, assign based on the dominant mechanism examined; do not assign if acidification is inferred but not directly analysed or measured; prioritise this category when studies explicitly quantify pH, saturation state, or carbonate chemistry parameters |
| Socio-geopolitical disruptions | External geopolitical, economic, or societal processes, including strategic competition, legal contestation, or divergence in governance norms, that disrupt or influence Antarctic governance, cooperation, or decision-making, thereby affecting the capacity to manage conservation threats | - Examines geopolitical tensions, strategic competition, or shifts in international relations that influence Antarctic governance - Analyses changes in global cooperation, diplomacy, or multilateral processes affecting ATS decision-making or coordination - Investigates how external crises or socio-economic pressures (e.g. pandemics, economic shocks) impact Antarctic operations or governance - Examines contestation, divergence, or overlap in governance systems (e.g. international law, competing frameworks, or liability and responsibility for environmental harm) that create tension or ambiguity in Antarctic decision-making | - Focuses on internal ATS governance, institutional design, or decision-making without external socio-political drivers - Examines operational, logistical, or environmental processes without a geopolitical or societal mechanism - Describes legal frameworks, cooperation, or governance arrangements without evidence of contestation or influence on decision-making - Mentions geopolitical or global context only as background, without analysing its effect on Antarctic governance or conservation outcomes | Assign only when external socio-political processes actively influence Antarctic governance or decision-making. Do not assign where such processes are absent, implicit, or secondary to internal governance or environmental dynamics. In borderline cases, prioritise whether the primary mechanism of change is external pressure (assign here) or internal system capacity (assign to ‘functioning of the Antarctic governance regime’). |
| Invasive alien species and disease | The introduction, establishment, or spread of non-native species and anthropogenically mediated pathogens in the Antarctic region, including diseases introduced or facilitated by human activity, with potential impacts on native ecosystems and biodiversity | - Examines introduction, establishment, or spread of non-native species in Antarctica - Investigates anthropogenically mediated pathogen introduction (e.g. COVID-19, avian influenza) - Analyses biosecurity risks and pathways associated with human activity - Examines mechanisms of spread linked to human movement, transport, or infrastructure - Assesses management, prevention, or mitigation of invasive species or disease risks - Links biological introductions to ecosystem impacts or biodiversity change | - Focuses on naturally occurring diseases or ecological interactions without human-mediated introduction - Examines endemic pathogens or natural disease dynamics in Antarctic species - Discusses climate-driven range shifts without explicit human-mediated introduction pathways - Analyses general biodiversity change without reference to invasive species or introduced disease - Mentions disease or species presence without identifying anthropogenic introduction or spread | Assign only where there is a clear anthropogenic pathway or mechanism; require explicit linkage between disease or species spread and human activity or global movement systems; include wildlife-mediated spread only where linked to human introduction or amplification; do not assign to vague references such as “disease risk” or “biosecurity concern” without a defined mechanism |
| Pollution | The introduction, transport, or accumulation of anthropogenic contaminants in the Antarctic environment, affecting air, land, water, ice, or biota. | - Examines contamination arising from human activity (e.g. plastics, hydrocarbons, heavy metals, sewage, persistent organic pollutants) - Identifies a contaminant source or pathway, including local release (e.g. stations, waste) or long-range transport - Assesses accumulation or ecological impacts of contaminants (e.g. bioaccumulation, environmental quality) - Evaluates pollution mitigation, waste management, or contamination prevention | - Focuses on climate change or greenhouse gases without a contaminant pathway - Examines natural geochemical processes without anthropogenic input - Describes physical disturbance or human activity without contamination - Focuses on invasive species or disease unless explicitly framed as contamination - Mentions pollution only in general or rhetorical terms | Require identification of a contaminant (or class of contaminants) and an anthropogenic source or pathway; include long-range transport only when contaminants are explicitly specified  classify as human activity impacts if focus is infrastructure or human presence without contaminants; classify based on emphasis if focus is governance or regulation (policy vs environmental contamination) |
| Functioning of the Antarctic governance regime | Processes, mechanisms, and institutional dynamics within the Antarctic Treaty System that maintain, enable, or influence its effective operation, including decision-making, coordination, compliance, and the implementation of environmental governance | - Examines internal ATS decision-making processes (e.g. consensus-based governance) - Analyses coordination, cooperation, or institutional performance within the ATS - Investigates compliance, enforcement, or implementation of ATS agreements - Evaluates the effectiveness, capacity, or functioning of the ATS as a governance system - Discusses institutional structures, procedures, or reforms aimed at maintaining or improving governance processes | - Focuses on external geopolitical dynamics or international relations beyond the ATS - Examines specific environmental threats without discussing governance processes - Discusses policy outcomes or environmental impacts without reference to institutional functioning - Analyses human activities or pressures unless explicitly linked to governance processes - Mentions governance in a general or descriptive way without evaluating its functioning or mechanism | Assign where the primary focus is on how the ATS operates (processes, mechanisms, or performance), rather than the specific issues it addresses; require explicit reference to institutional functioning; do not assign where governance is only background context; where both governance and a specific threat are discussed, classify based on the dominant focus |
| Mineral and hydrocarbon exploitation | The exploration, prospecting, or potential extraction of mineral and hydrocarbon resources in the Antarctic region, including activities, interests, or pressures related to their development and use | - Examines exploration, prospecting, or extraction of minerals or hydrocarbons in Antarctica - Discusses interest in or potential development of Antarctic mineral or hydrocarbon resources - Analyses technological, economic, or logistical feasibility of mineral or hydrocarbon extraction - Investigates pressures or drivers associated with accessing mineral or hydrocarbon resources - Evaluates risks, implications, or consequences of mineral or hydrocarbon exploitation - Discusses historical or contemporary proposals related to Antarctic mineral or hydrocarbon resource use | - Examines environmental impacts without reference to mineral or hydrocarbon exploration or extraction - Discusses geopolitical dynamics or international relations without explicit linkage to resource exploitation - Analyses scientific research on Antarctic geology without connection to resource use or extraction - Focuses on living or renewable resources (e.g. fisheries or biological resources) - Mentions resources in a descriptive or background context only | Assign only where there is an explicit link to exploration, extraction, or utilisation of mineral or hydrocarbon resources; include both realised and hypothetical exploitation (e.g. future scenarios or economic interest); if both governance and exploitation are discussed, assign based on the dominant focus; distinguish from human activities impacts by requiring a clear resource extraction or utilisation component |
| Bioprospecting | The search for, collection, and utilisation of Antarctic biological material for commercial, industrial, or biotechnological applications, including associated scientific, economic, or strategic interests | - Examines the discovery, collection, or use of Antarctic organisms or genetic material for commercial or industrial purposes - Investigates biotechnological, pharmaceutical, or industrial applications derived from Antarctic biodiversity - Discusses the potential or development of biological resources for commercial use - Analyses economic, technological, or logistical aspects of accessing or utilising Antarctic biological material - Evaluates implications arising from the use of Antarctic biological or genetic resources | - Focuses on basic scientific research without connection to commercial or applied use - Examines ecological, biological, or genetic studies without reference to utilisation or application - Discusses conservation, biodiversity, or ecosystem processes without linkage to resource use - Examines mineral, hydrocarbon, or other non-biological resource exploitation - Mentions biological resources in a descriptive or background context only | Require explicit linkage to utilisation, application, or commercial potential of biological material; do not assign where research is purely descriptive or exploratory without downstream use ; include early-stage or hypothetical applications where intent or potential use is clearly stated; distinguish from human activity impacts by requiring a biological resource use component, not just presence or activity |
| Human activity impacts | The direct physical impacts of human presence and activities in the Antarctic region, including infrastructure, transport, and operational activities that alter or disturb the environment | - Examines impacts arising from human presence (e.g. research stations, tourism, field operations) - Investigates environmental disturbance from infrastructure development or expansion - Analyses impacts of transport and logistics (e.g. shipping, aviation, overland travel) - Examines direct physical disturbance to habitats, wildlife, or ecosystems (e.g. trampling, noise, site disturbance) - Evaluates management or mitigation of impacts arising from human activities | - The primary mechanism is not direct physical disturbance from human activity - Human activity is mentioned only as background context without assessing environmental impact | Assign only where there is a clear, direct link between human activity and environmental disturbance; if multiple mechanisms are present, assign based on the dominant impact pathway; use this category for direct disturbance not better explained by a more specific mechanism |
| No conservation threat | Document content that does not substantively engage with any of the defined Antarctic conservation threat categories, even if it concerns Antarctic science, logistics, policy, or environmental systems more broadly | - Analyses physical, biological, or environmental processes without linking them to conservation impacts or threats - Examines variability or system dynamics without attributing change to a defined conservation threat - Focuses on methods, models, monitoring, or data collection without interpreting results in a conservation context | - The document explicitly links processes or activities to environmental, ecological, or institutional impacts - A defined conservation threat is a primary analytical focus, not background context - The document frames findings in terms of risk, impact, vulnerability, or management relevance - Human activities, environmental change, or governance are analysed in relation to their consequences for Antarctic systems | Assign to no conservation threat when the document contributes scientific, operational, or policy context without explicitly analysing a defined conservation threat. Prioritise the primary analytical focus over incidental mentions, and do not infer conservation relevance unless it is clearly established |

#### 4.3.2 Interpretation

At the aggregate level, classification performance demonstrated good agreement between model-derived and manually assigned labels, with macro-averaged precision, recall, and F1-scores of 82.1%, 86.4%, and 83.3%, respectively (**Supplementary Table 10**). Performance was broadly consistent across corpora, with scientific documents showing marginally higher precision (84.7%) and recall (88.3%) than policy documents (83.0% and 85.6%, respectively), resulting in a slightly higher F1-score for the scientific subset (85.3% vs 83.2%). Across all subsets, recall consistently exceeded precision, indicating that the model is more likely to identify documents containing conservation threats than to miss them, albeit at the cost of some over-classification of documents that do not substantively engage with these threats.

**Supplementary Table 10**. Summary performance metrics for topic-to-threat classification across document types.

| Sampled document type | Precision | Recall | F1-score |
| --- | --- | --- | --- |
| Overall | 82.1 | 86.4 | 83.3 |
| Scientific | 84.7 | 88.3 | 85.3 |
| Policy | 83.0 | 85.6 | 83.2 |

Across categories, classification performance varied, with F1-scores ranging from 64% to 92% (**Supplementary Table 11**; **Supplementary Figure 8**). Several threats were classified with relatively high performance, including invasive alien species and disease (F1 = 90%), bioprospecting (92%), mineral and hydrocarbon exploitation (87%), and pollution (88%). By contrast, lower performance was observed for the functioning of the Antarctic governance regime (F1 = 78%), human activity impacts (76%), and the ‘no conservation threat’ category (64%). The observed variation in F1-scores across conservation threats reflects how distinctly each threat is represented within the topic structure. Threats characterised by well-separated thematic signals were more reliably captured by LDA topics, whereas those with broader or overlapping representations were more difficult to delineate. The mechanisms underlying these differences, including the distinct forms of misclassification observed across threat categories, are examined in detail below.

**Supplementary Table 11**. Per-category classification performance for topic-to-threat mapping across the validation dataset.

| Conservation threat | True positive | False positive | False negative | Precision | Recall | F1 |
| --- | --- | --- | --- | --- | --- | --- |
| Climate change | 71  [39, 32] | 9  [1, 8] | 5  [3, 2] | 89 (80-94)^a^  [98, 80]^b^ | 93 (86-97) [93, 94] | 91  [95, 86] |
| Ocean acidification | 28  [27, 1] | 13  [13, 0] | 0  [0 0] | 68 (53-80) [68, 100] | 100 (88-100) [100, 100] | 81 [81, 100] |
| Socio-geopolitical disruptions | 64  [35, 29] | 16  [5, 11] | 3  [2, 1] | 80 (70-87) [88, 72] | 96 (88-98) [95, 97] | 87  [91, 83] |
| Invasive alien species & disease | 57  [33, 24] | 8  [7, 1] | 5  [2, 3] | 88 (78-94) [82, 96] | 92 (82-97) [94, 89] | 90  [88, 92] |
| Pollution | 67  [34, 33] | 13  [6, 7] | 5  [3, 2] | 84 (74-90) [85, 82] | 93 (85-97) [92, 94] | 88  [88, 88] |
| Functioning of the Antarctic governance regime | 62  [28, 34] | 18  [12, 6] | 18  [2, 16] | 78 (67-85) [70, 85] | 78 (67-85) [93, 68] | 78  [80, 76] |
| Mineral and hydrocarbon exploitation | 63  [37, 26] | 17  [3, 14] | 2  [2, 0] | 79 (69-86) [92, 65] | 97 (89-99) [95, 100] | 87  [94, 79] |
| Bioprospecting | 55  [37, 18] | 4  [3, 1] | 5  [4, 1] | 93 (84-97) [92, 95] | 92 (82-96) [90, 95] | 92  [91, 95] |
| Human activity impacts | 66  [32, 34] | 14  [8, 6] | 27  [8, 19] | 82 (73-89) [80, 85] | 71 (61-79) [80, 64] | 76  [80, 73] |
| No conservation threat | 65  [37, 28] | 15  [3, 12] | 57  [35, 22] | 81 (71-88) [92, 70] | 53 (44-62) [51, 56] | 64  [66, 62] |

^a^ Values in parentheses correspond to 95% confidence intervals for performance metrics calculated across the combined validation dataset. Confidence intervals are shown only for combined precision and recall values.

^b^ Values in brackets correspond to metrics for the scientific corpus (first value) and policy corpus (second value).

**Supplementary Figure 8**. Row-normalised confusion matrix comparing model-derived and manually assigned conservation threat labels for the full validation dataset.

Misclassification arose from two primary sources: reassignment among conceptually related conservation threats and over-assignment of conservation threats to documents that did not substantively engage with them. Errors arising from reassignment among closely related threats were concentrated in a small number of categories. The most pronounced case was the functioning of the Antarctic governance regime, where 15% of manually classified documents were instead assigned to socio-geopolitical disruptions (n = 12), a pattern driven primarily by the policy corpus (20%; n = 10, compared with 7%; n = 2 in the scientific corpus) (**Supplementary Figure 9**). This reflects overlap between procedural or institutional discussions and broader geopolitical themes. A similar, though more distributed, pattern was observed for human activity impacts, where documents were reassigned to mineral and hydrocarbon exploitation (11%; n = 10) and pollution (8%; n = 7), with misclassification again more pronounced in the policy corpus (**Supplementary Figure 9**). In these cases, the content does describe human activities but is more strongly defined by specific pressures, leading it to be more consistently assigned to narrower threat categories.

**Supplementary Figure 9.** Row-normalised confusion matrices showing agreement between model-derived and manually assigned conservation threat labels for scientific and policy corpora.

Over-classification primarily arose from documents being assigned to conservation threat categories rather than classified as ‘no conservation threat’. Accordingly, the ‘no conservation threat’ category exhibited relatively high precision (81%) but substantially lower recall (53%), indicating that while assignments to this category were generally reliable, many documents without a clearly defined conservation threat were nevertheless reassigned to a conservation threat category. These misclassifications were distributed across multiple conservation threats and typically represented only a small proportion of documents assigned to each category. However, reassignment of ‘no conservation threat’ documents was more pronounced for ocean acidification (22%; 9 of 41 assigned documents), the functioning of the Antarctic governance regime (19%; 15 of 80 assigned documents), and, to a lesser extent, bioprospecting (7%; 4 of 59 assigned documents). These patterns reflect the presence of related thematic content that does not explicitly constitute a conservation threat.

Ocean acidification provides a clear example of this over-assignment pattern, with perfect recall (100%) but comparatively lower precision (68%). This indicates that all manually identified ocean acidification documents were successfully captured, but that the model also assigned additional documents to this category. These misclassifications appear to arise from overlap with broader ocean biogeochemistry, where closely related processes share similar thematic signals (e.g., studies of methane exchange, atmospheric greenhouse gases, microbial trace-gas oxidation, and marine nutrient cycling). As a result, the model captures a broader biogeochemical signal associated with ocean acidification, encompassing both acidification-specific studies and more general ocean biogeochemical dynamics.

Importantly, the validation framework used here differs from the probabilistic framework used in the primary analysis by forcing each document into a single dominant classification. In the primary analysis, however, documents retain their full probabilistic topic distributions and may contribute simultaneously to multiple conservation threats. This hard classification framework may therefore inflate apparent misclassification where documents contain overlapping or weaker thematic signals. To evaluate this possibility, we examined documents that were manually classified as ‘no conservation threat’ but were instead assigned by the model to a conservation threat category. We then assessed whether ‘no conservation threat’ remained the second-strongest model-derived signal within those documents.

Among the 57 documents manually classified as ‘no conservation threat’ but reassigned by the dominant-topic validation framework to a conservation threat category, 47 (82.5%) ranked ‘no conservation threat’ as the second-strongest model-derived signal. This suggests that many apparent false positives arise from the stringent nature of the hard classification framework rather than complete semantic misclassification. The probabilistic model assigns documents mixtures of thematic signals with varying strengths, but the validation framework forces each document into a single dominant category. Consequently, some documents were classified as errors even when substantial non-threat thematic structure remained strongly represented within the model output.

Interpreting these metrics requires consideration of the structure of the classification task. Documents are assigned to partially overlapping conservation threats based on probabilistic topic representations, so some degree of ambiguity is unavoidable ^91^. There is also no universal threshold for acceptable performance in multi-class classification, and single summary metrics can mask important aspects of model behaviour ^102,103^. These metrics therefore evaluate the consistency of the dominant-topic operationalisation used for validation, rather than the full probabilistic framework used in the primary corpus-level analyses. In this context, classification performance was strong and internally consistent, with macro-averaged precision, recall, and F1-scores of 82.1%, 86.4%, and 83.3%, respectively, and similar values across the scientific and policy corpora. With ten conservation threat categories, this level of agreement is substantially above chance expectations and indicates that the manually derived topic-to-threat alignment captures meaningful thematic structure across the corpus.

Over-assignment of conservation threats nevertheless introduces some inflation of individual categories, particularly where weak or diffuse thematic signals overlap with broader conservation topics. The additional diagnostic analyses suggest that many apparent false positives arise from the stringent hard-classification framework imposed during validation rather than complete semantic misclassification, particularly for documents manually classified as ‘no conservation threat’. Although it is not possible to determine directly whether this inflation was distributed uniformly through time, the major temporal patterns identified in the policy corpus correspond closely with well-documented periods of ATS negotiation, implementation, and institutional change (Figures 2–3, Main Text). This correspondence suggests that the dominant temporal dynamics primarily reflect underlying institutional processes rather than artefacts of classification. Nevertheless, conservation threats exhibiting lower precision, particularly ocean acidification, should be interpreted more cautiously than higher-performing categories.

### Note 4.4. Sensitivity analysis.

Natural language processing models are not uniquely determined by the underlying data, but depend on a range of modelling decisions, including preprocessing and parameter specification ^86^. To assess whether the patterns identified by our LDA model reflect meaningful structure in the corpus, rather than artefacts of these choices, we conducted two complementary sensitivity analyses. First, we investigated how alternative term-frequency thresholds for excluding rare and highly frequent terms altered the composition of the model vocabulary and the resulting topic structure. Second, we evaluated the sensitivity of the model to parameter specification by varying key hyperparameters and refitting the model under each configuration while holding the vocabulary constant. We conducted these analyses separately because changing term-frequency thresholds alters the vocabulary underlying the model, resulting in topic structures that are not directly comparable across configurations ^104^. We therefore first defined a stable and interpretable vocabulary before evaluating sensitivity to model specification within this fixed feature space.

#### 4.4.1 Sensitivity to vocabulary construction

##### Methods

Because document-frequency thresholds influence both the interpretability of inferred topics and the retention of semantically meaningful vocabulary, we evaluated a range of alternative threshold configurations to identify a balanced vocabulary specification. Terms occurring in very few documents were removed to reduce the influence of sparse or potentially spurious tokens, whereas highly ubiquitous terms were excluded because they provide limited discriminative information for topic inference (see Supplementary Note 1.2.2 for implementation details). To evaluate sensitivity to vocabulary trimming, we systematically varied the proportion of rare and highly frequent terms excluded from the corpus and assessed the resulting changes in inferred topic structure. Because the effects of vocabulary trimming on topic structure may depend partly on model parameterisation ^105^, all vocabulary configurations were evaluated across a set of plausible LDA hyperparameter values rather than under a single fixed specification. This allowed us to assess how vocabulary trimming influenced topic coherence and vocabulary retention across alternative vocabulary definitions and to identify a balanced threshold configuration.

We evaluated a series of vocabulary configurations in which the minimum and maximum document-frequency thresholds were varied symmetrically. Specifically, we tested paired document-frequency thresholds of 5–95%, 10–90%, 15–85%, and 20–80%, corresponding to progressively more restrictive definitions of the model vocabulary ^104^. For each configuration, the corpus was re-vectorised and a new LDA model was fitted so that the inferred topic structure reflected the modified vocabulary. Each vocabulary specification was then evaluated across five hyperparameter configurations of the symmetric Dirichlet priors on document–topic (α) and topic–word (β) distributions: α = 0.1, β = 0.005; α = 0.05, β = 0.005; α = 0.1, β = 0.001; α = 0.1, β = 0.01; and α = 0.2, β = 0.005. The purpose of varying hyperparameters within this subsection was not to optimise model specification for each vocabulary configuration, but to determine whether conclusions regarding vocabulary selection were sensitive to a reasonable range of modelling assumptions.

To evaluate how vocabulary trimming influenced the interpretability of inferred topics, we quantified topic coherence across all vocabulary configurations. Topic coherence was evaluated using Normalised Pointwise Mutual Information (NPMI), a co-occurrence-based metric that measures the semantic relatedness of high-probability words within topics, with higher values indicating more interpretable and internally coherent topic structure ^53^. NPMI is widely used in topic modelling because it corresponds more closely with human judgements of topic interpretability than unnormalised PMI-based measures (see Supplementary Note 1.2.2 for complete details) ^53^. For each fitted model, we calculated NPMI using the top 20 words per topic and summarised each model using the median NPMI across topics, consistent with the number of words used for manual topic interpretation and assignment to conservation threats. Median NPMI values were then compared across vocabulary configurations to assess the trade-off between improved topic coherence and increasingly restrictive vocabulary trimming.

Selecting document-frequency thresholds in topic modelling involves balancing improved coherence against retention of semantically meaningful vocabulary, and higher coherence can partly reflect the removal of terms that increase topic heterogeneity ^105^. We therefore did not select the final vocabulary configuration solely by maximising NPMI. Instead, we complemented coherence-based evaluation with a quantitative inspection of the excluded vocabulary. Specifically, we classified removed terms as either dictionary-recognised words or non-dictionary tokens (e.g. typographical variants and non-standard terms) and quantified how the number and rate of removal of each category varied across document-frequency thresholds. This allowed us to evaluate how vocabulary trimming affected topic coherence and vocabulary retention across a range of plausible model specifications, and to identify a threshold configuration that balanced these competing objectives.

##### Results

The distribution of excluded tokens was strongly skewed towards low term-frequency thresholds (**Supplementary Figure 10**). At a 1% threshold, approximately 150,000 non-dictionary tokens (i.e. non-recognised words and typographical variants) were removed compared to ~5,000 dictionary words, indicating that early trimming primarily removes low-frequency, non-standard terms. As the threshold increased, the number of excluded tokens declined rapidly, with non-dictionary tokens decreasing by more than an order of magnitude between 1% and 5% (~150,000 to ~5,000), and to fewer than ~1,500 by 10%, while dictionary words declined more gradually (~5,000 to ~900 to ~450 over the same interval) (**Supplementary Figure 10**).

**Supplementary Figure 10.** Number of excluded dictionary words (left) and non-dictionary tokens (right) across document-frequency thresholds.

The magnitude of these reductions decreased across successive thresholds. For non-dictionary tokens, percentage reductions fell from 96.6% between 1% and 5% to 72.3% between 5% and 10%, and to 54.0% and 40.6% across the 10–15% and 15–20% intervals, respectively. Dictionary words followed a similar but less pronounced pattern (81.5%, 51.5%, 40.0%, and 29.4%; **Supplementary Table 12**), indicating that reductions in non-dictionary tokens diminish more rapidly with increasing threshold, whereas the removal of dictionary words continues at a comparatively steady rate.

**Supplementary Table 12.** Changes in excluded non-dictionary tokens and dictionary words across document-frequency thresholds.

| Interval | Non-dictionary tokens removed  (start → end) | Δ (%) | Dictionary words removed  (start → end) | Δ (%) |
| --- | --- | --- | --- | --- |
| 1 → 5% | 150,103 → 5,090 | -96.6 | 4,986 → 923 | -81.5 |
| 5 → 10% | 5,090 → 1,413 | -72.3 | 923 → 448 | -51.5 |
| 10 → 15% | 1,413 → 650 | -54.0 | 448 → 269 | -40.0 |
| 15 → 20% | 650 → 386 | -40.6 | 269 → 190 | -29.4 |

To assess how these changes in vocabulary composition affect model performance, we evaluated topic coherence across alternative trimming thresholds. Topic coherence increased consistently with more restrictive vocabulary definitions (**Supplementary Figure 11**), with the lowest values observed under 5–95% thresholds (median NPMI = 0.112–0.139) and progressively higher values under 10–90% (0.133–0.150), 15–85% (0.159–0.179), and 20–80% configurations (0.184–0.195). The largest increase in coherence occurred between the 10–90% and 15–85% thresholds and was consistent across all parameterisations, distinguishing lower-coherence configurations (5–95% and 10–90%) from higher-coherence configurations (15–85% and 20–80%). We therefore excluded the 5–95% and 10–90% configurations from further consideration. The remaining comparison was thus between the 15–85% and 20–80% thresholds, which represented alternative trade-offs between topic coherence and retention of interpretable vocabulary.

**Supplementary Figure 11.** Median topic coherence (NPMI) across vocabulary configurations and hyperparameter settings. Bars show median NPMI values for models fitted using minimum–maximum document-frequency thresholds of 5–95%, 10–90%, 15–85%, and 20–80%. Colours denote different α–β parameterisations.

Comparison of the remaining thresholds showed that although the 20–80% configuration produced the highest median NPMI values, the increase in coherence relative to the 15–85% configuration was comparatively small. In contrast, analyses of excluded vocabulary showed that the proportion of excluded terms recognised as dictionary words exceeded 30% from thresholds ≥17% (**Supplementary Figure 12**), while additional reductions in non-dictionary tokens were limited (**Supplementary Figure 10**; **Supplementary Table 12**). Because the analysis aims to capture nuanced conservation-related concepts, retaining interpretable vocabulary is essential. We therefore selected the 15–85% threshold as a balanced configuration that improves topic coherence while avoiding unnecessary loss of meaningful terms.

**Supplementary Figure 12.** Proportion of excluded terms recognised as dictionary words across document-frequency thresholds. The dotted line indicates 30%.

#### 4.4.2 Sensitivity to model specification

##### Methods

Following selection of the 15–85% document-frequency thresholds, we evaluated the sensitivity of the topic modelling framework to model specification. We varied the Dirichlet priors governing document–topic (α) and topic–word (β) distributions while holding the vocabulary specification and number of topics (K = 90) constant, the latter having previously been identified as the optimal topic resolution for the corpus (see Supplementary Note 1.2.1 for complete details). This allowed differences among model outputs to be attributed specifically to alternative α–β parameterisations rather than changes to the underlying representation or granularity of the corpus.

We evaluated four alternative α–β parameterisations alongside the reference model used in the primary analysis (**Supplementary Table 13**). These parameterisations were selected to induce varying levels of sparsity and diffusivity in document–topic and topic–word distributions, spanning topic structures from highly specialised representations to more diffuse thematic mixtures.

**Supplementary Table 13**. α–β parameterisations used in the sensitivity analysis of model specification.

| Model | α | β | Expected effect |
| --- | --- | --- | --- |
| Reference | 0.1 | 0.005 | Baseline sparse representation, producing relatively specialised documents and topics |
| 01-0001 | 0.1 | 0.001 | More concentrated topic-word distributions, with topics defined by smaller sets of highly specialised words |
| 01-001 | 0.1 | 0.01 | More diffuse topic-word distributions, with broader and more overlapping vocabularies across topics |
| 005-0001 | 0.05 | 0.001 | More concentrated document-topic mixtures, with documents associated with fewer dominant topics |
| 02-0005 | 0.2 | 0.005 | More diffuse document-topic mixtures, with documents distributed across larger numbers of topics |

After model fitting, topics were reassigned to conservation threats by repeating the same structured review framework applied in the primary analysis (see Supplementary Note 3 for complete details). Briefly, this process involved reviewing representative documents and topic-word distributions to determine whether topics aligned with the predefined conservation threat categories. Following topic assignment, we repeated the downstream analytical workflow for each model configuration by fitting cubic splines to the temporal trajectories of each conservation threat and calculating periods of significant increase and decline using the same derivative-based framework applied in the primary analysis.

Robustness was assessed by comparing the temporal dynamics inferred under each α–β parameterisation with those of the reference model for each conservation threat. We first evaluated whether major historically interpretable periods of change identified in the primary analysis were consistently reproduced across parameterisations. For each model configuration, we quantified the number of directional reversals (“flips”) between significant periods of increase and decline identified from the spline derivatives for each conservation threat. This approach is consistent with the primary analysis, which focused on broad temporal dynamics and historically interpretable periods of directional change rather than exact year-by-year correspondence. We then calculated the absolute deviation in the number of reversals relative to the reference model for each conservation threat. Exact temporal alignment of spline trajectories and fitted magnitudes was not expected across parameterisations because topic modelling is a stochastic and non-identifiable process in which alternative parameter settings can yield different but similarly plausible decompositions of semantic structure. Consequently, robustness was evaluated based on whether alternative model specifications reproduced similar overall frequencies and relative patterns of directional change rather than identical year-by-year trajectories. Differences in the number of directional reversals between sensitivity models and the reference model were assessed separately for scientific and policy corpora using paired Wilcoxon signed-rank tests. We expected parameterisations closer to the reference model to reproduce more similar temporal dynamics than more strongly perturbed configurations.

##### Interpretation

Broad temporal dynamics were consistently reproduced across alternative α–β parameterisations, with both fitted spline trajectories (**Supplementary Figure 13**) and periods of significant directional change (**Supplementary Figure 14** and **Supplementary Figure 15**) remaining qualitatively similar across models. Major historically interpretable periods of change identified in the primary analysis were likewise preserved across parameterisations. More strongly perturbed parameterisations produced modest shifts in topic structure, including broader thematic distributions in diffuse models and narrower representations in more concentrated models, resulting in some variation in spline magnitudes and the exact timing of directional transitions. However, these differences did not substantially alter the broader temporal narratives inferred from the corpus because comparable thematic content could still be reconstructed across parameterisations by adjusting the number of topics assigned to individual conservation threats. Consequently, the principal conservation dynamics identified in the primary analysis remained robust to reasonable perturbations of model specification.

Importantly, major historically interpretable periods of change identified in the primary analysis were likewise preserved across parameterisations within the policy corpus. For example, discussions related to mineral and hydrocarbon exploitation consistently increased through the 1970s and early 1980s before declining following negotiations surrounding the Convention on the Regulation of Antarctic Mineral Resource Activities (CRAMRA). Similarly, policy discussions concerning pollution increased throughout the 1980s and remained elevated until the implementation of the Protocol on Environmental Protection to the Antarctic Treaty in the late 1990s. Discussions surrounding the functioning of the Antarctic Treaty System also showed consistent increases through the 1980s as additional Parties joined the Treaty and the governance framework expanded in complexity. Despite variation in topic structure among parameterisations, these broader historical dynamics remained qualitatively stable across models.

Ocean acidification represented the least stable conservation threat across parameterisations. Only two models (01_001 and 01_0001; **Supplementary Figure 13**) recovered a distinct ocean acidification signal and, even in these cases, the inferred temporal dynamics differed from those identified in the primary analysis. This instability likely reflects the comparatively subtle and overlapping semantic structure of ocean acidification within the corpus, where discussions of acidification are frequently embedded within broader themes related to climate change, marine systems, and oceanographic processes. Consequently, the topic was often subsumed within neighbouring thematic structures rather than emerging as a consistently distinct conservation threat across parameterisations.

Quantitative comparisons of directional reversals likewise indicated strong agreement between sensitivity models and the reference configuration. In most cases, alternative parameterisations reproduced the same number of significant directional reversals (“flips”) identified in the primary analysis, with at least half of all threat–corpus comparisons showing no deviation from the reference model. Even where differences occurred, they were generally small, with sensitivity models differing from the reference configuration by an average of only 0.24 flips and never by more than two flips. Consistent with this interpretation, paired Wilcoxon signed-rank tests identified no significant differences in the number of directional reversals between sensitivity models and the reference model when scientific and policy corpora were analysed together (p = 0.43) or separately (scientific: p = 0.89; policy: p = 0.45). These results indicate that perturbations to model priors produced only minor differences in directional change and did not substantially alter the broader temporal dynamics inferred from the corpus.

Overall, these results indicate that the principal temporal dynamics identified in the primary analysis are robust to reasonable perturbations of model specification, particularly at the scale of broad conservation narratives and historically interpretable periods of change. At the same time, the sensitivity analyses demonstrate that individual spline trajectories can vary among parameterisations because alternative topic models partition semantic structure differently across topics. Consequently, while broad directional patterns were consistently reproduced, the exact magnitudes of spline responses and the precise timing of directional transitions should not be interpreted as fixed temporal estimates. Rather, the modelling framework is most informative for identifying persistent large-scale patterns of change and the relative prominence of conservation threats within the scientific and policy corpora. Ocean acidification represented the primary exception to this broader pattern of robustness and should therefore be interpreted more cautiously than the remaining conservation threats. The lack of consistent temporal dynamics across parameterisations prevents strong temporal inference for this threat.

**Supplementary Figure 13.** Sensitivity of fitted spline trajectories to alternative α–β parameterisations of the topic model. Fitted response curves are shown for each conservation threat across the scientific and ATCM policy corpora under the reference model and four perturbed parameterisations. Shaded regions indicate major historically relevant periods in Antarctic governance.

**Supplementary Figure 14.** Significant periods of directional change identified from spline derivatives for the scientific corpus across alternative α–β parameterisations. Green segments indicate statistically significant increases in fitted response through time, whereas grey segments indicate significant declines.

**Supplementary Figure 15.** Significant periods of directional change identified from spline derivatives for the scientific corpus across alternative α–β parameterisations. Green segments indicate statistically significant increases in fitted response through time, whereas grey segments indicate significant declines.

## Supplementary References

1	Chown, S. L. *et al.* Challenges to the Future Conservation of the Antarctic. *Science* **337**, 158-159, doi:<https://doi.org/10.1126/science.1222821> (2012).

2	Carter, Z. T. *et al.* Emerging threats to Antarctic conservation. *Nature Ecology & Evolution*, doi:<https://doi.org/10.1038/s41559-025-02814-4> (2025).

3	Chown, S. L. in *Handbook on the politics of Antarctica*	(eds Klaus Dodds, A.D. Hemmings, & P. Roberts) 17 (Edward Elgar Publishing Limited, 2017).

4	Blei, D. M., Ng, A. Y. & Jordan, M. I. Latent dirichlet allocation. *Journal of Machine Learning Research* **3**, 993-1022 (2003).

5	Hughes, K. A., Lowther, A., Gilbert, N., Waluda, C. M. & Lee, J. R. Communicating the best available science to inform Antarctic policy and management: a practical introduction for researchers. *Antarctic Science* **35**, 438-472, doi:<https://doi.org/10.1017/S095410202300024X> (2023).

6	Vignon, É., Roussel, M.-L., Gorodetskaya, I. V., Genthon, C. & Berne, A. Present and Future of Rainfall in Antarctica. *Geophysical Research Letters* **48**, e2020GL092281, doi:<https://doi.org/10.1029/2020GL092281> (2021).

7	Ropert-Coudert, Y. *et al.* A complete breeding failure in an Adélie penguin colony correlates with unusual and extreme environmental events. *Ecography* **38**, 111-113, doi:<https://doi.org/10.1111/ecog.01182> (2015).

8	Maclennan, M. L., Lenaerts, J. T. M., Shields, C. & Wille, J. D. Contribution of Atmospheric Rivers to Antarctic Precipitation. *Geophysical Research Letters* **49**, e2022GL100585, doi:<https://doi.org/10.1029/2022GL100585> (2022).

9	Mankoff, K. *et al.* Exploratory Antarctic Ice Loss Intervention Workshop. 15 (Stanford, CA, 2024).

10	Siegert, M. *et al.* *Safeguarding the polar regions from dangerous geoengineering* (2024).

11	ASOC. ASOC report to the ATCM. Report No. IP 142, (Antarctic Treaty Consultative Meeting (ATCM), Kochi, India, 2024).

12	Tscherning, K., Helming, K., Krippner, B., Sieber, S. & Paloma, S. G. y. Does research applying the DPSIR framework support decision making? *Land Use Policy* **29**, 102-110, doi:<https://doi.org/10.1016/j.landusepol.2011.05.009> (2012).

13	Meredith, M. *et al.* in *IPCC Special report on the ocean and cryosphere in a changing climate*	(eds H.-O. Pörtner *et al.*) Ch. 3, 203-321 (Cambridge University Press, 2019).

14	Rignot, E. *et al.* Four decades of Antarctic Ice Sheet mass balance from 1979–2017. *Proceedings of the National Academy of Sciences* **116**, 1095-1103, doi:<https://doi.org/10.1073/pnas.1812883116> (2019).

15	Barrett, J. E. *et al.* Response of a Terrestrial Polar Ecosystem to the March 2022 Antarctic Weather Anomaly. *Earth's Future* **12**, e2023EF004306, doi:<https://doi.org/10.1029/2023EF004306> (2024).

16	Gunn, K. L., Rintoul, S. R., England, M. H. & Bowen, M. M. Recent reduced abyssal overturning and ventilation in the Australian Antarctic Basin. *Nature Climate Change* **13**, 537-544, doi:<https://doi.org/10.1038/s41558-023-01667-8> (2023).

17	Holland, P. R. The seasonality of Antarctic sea ice trends. *Geophysical Research Letters* **41**, 4230-4237, doi:<https://doi.org/10.1002/2014GL060172> (2014).

18	Griffiths, H. J., Cummings, V. J., Van de Putte, A., Whittle, R. J. & Waller, C. L. Antarctic benthic ecological change. *Nature Reviews Earth & Environment* **5**, 645-664, doi:<https://doi.org/10.1038/s43017-024-00583-5> (2024).

19	Figuerola, B. *et al.* A Review and Meta-Analysis of Potential Impacts of Ocean Acidification on Marine Calcifiers From the Southern Ocean. *Frontiers in Marine Science* **8**, doi:<https://doi.org/10.3389/fmars.2021.584445> (2021).

20	Hennige, S. J. *et al.* Crumbling Reefs and Cold-Water Coral Habitat Loss in a Future Ocean: Evidence of “Coralporosis” as an Indicator of Habitat Integrity. *Frontiers in Marine Science* **7**, doi:<https://doi.org/10.3389/fmars.2020.00668> (2020).

21	Liverpool, L. Russia’s war in Ukraine is disrupting Antarctic science. *Nature* **621**, 453-453, doi:<https://doi.org/10.1038/d41586-023-02764-6> (2023).

22	Liggett, D., Frame, B., Convey, P. & Hughes, K. A. How the COVID-19 pandemic signaled the demise of Antarctic exceptionalism. *Science Advances* **10**, eadk4424, doi:<https://doi.org/10.1126/sciadv.adk4424> (2024).

23	Graham, K. & Webb, J. Fit for purpose? A study of the political and legal aspects of CAMLR. 41 (New Zealand Centre for Global Studies, Auckland, New Zealand, 2022).

24	Bergstrom, D. M. Maintaining Antarctica's isolation from non-native species. *Trends in Ecology & Evolution* **37**, 5-9, doi:<https://doi.org/10.1016/j.tree.2021.10.002> (2022).

25	Committee for Environmental Protection (CEP). Non-native species manual (Revision 2019). Report No. DDC 578.6/2, 41 (Buenos Aires, Argentina, 2019).

26	Leihy, R. I., Peake, L., Clarke, D. A., Chown, S. L. & McGeoch, M. A. Introduced and invasive alien species of Antarctica and the Southern Ocean Islands. *Scientific Data* **10**, 200, doi:<https://doi.org/10.1038/s41597-023-02113-2> (2023).

27	Bennett, J. R. *et al.* Polar lessons learned: long-term management based on shared threats in Arctic and Antarctic environments. *Frontiers in Ecology and the Environment* **13**, 316-324, doi:<https://doi.org/10.1890/140315> (2015).

28	Stark, J. S. *et al.* Contamination of the marine environment by Antarctic research stations: Monitoring marine pollution at Casey station from 1997 to 2015. *PLOS ONE* **18**, e0288485, doi:<https://doi.org/10.1371/journal.pone.0288485> (2023).

29	Cabrerizo, A., Larramendi, R., Albar, J.-P. & Dachs, J. Persistent organic pollutants in the atmosphere of the Antarctic Plateau. *Atmospheric Environment* **149**, 104-108, doi:<https://doi.org/10.1016/j.atmosenv.2016.11.015> (2017).

30	Gardiner, N. B., Gilbert, N., Liggett, D. & Bode, M. Measuring the performance of Antarctic Treaty decision-making. *Conservation Biology*, e14349, doi:<https://doi.org/10.1111/cobi.14349> (2024).

31	Hemmings, A. D. Challenges to Substantive Demilitarisation in the Antarctic Treaty Area. *The Yearbook of Polar Law Online* **12**, 172-194, doi:<https://doi.org/10.1163/22116427_012010012> (2020).

32	Madani, Z. & Shibata, A. International law, climate change and the Antarctic Treaty System: re-contemplating governance questions apropos of the mounting challenges. *Antarctic Science* **35**, 374-389, doi:<https://doi.org/10.1017/S0954102023000226> (2023).

33	Dodds, K. J. Sovereignty watch: claimant states, resources, and territory in contemporary Antarctica. *Polar Record* **47**, 231-243, doi:<https://doi.org/10.1017/S0032247410000458> (2011).

34	Hemmings, A. D. & Stephens, T. Reconciling Regional and Global Dispensations: The Implications of Sub-Antarctic Extended Continental Shelf Penetration of the Antarctic Treaty Area. *The New Zealand Yearbook of International Law* **6**, 273-291 (2008).

35	SCAR. in *Antarctic Treaty Consultative Meeting (XLIII)*	11 (Secretariat of the Antarctic Treaty, Paris, France, 2021).

36	Netherlands. IP 29 Biological prospecting in the Antarctic Treaty Area. 21 (2018).

37	Danilovich, M. E., Sánchez, L. A., Acosta, F. & Delgado, O. D. Antarctic bioprospecting: in pursuit of microorganisms producing new antimicrobials and enzymes. *Polar Biology* **41**, 1417-1433, doi:<https://doi.org/10.1007/s00300-018-2295-4> (2018).

38	Ramasamy, K. P. *et al.* Comprehensive insights on environmental adaptation strategies in Antarctic bacteria and biotechnological applications of cold adapted molecules. *Frontiers in Microbiology* **14**, doi:<https://doi.org/10.3389/fmicb.2023.1197797> (2023).

39	Brooks, S. T., Jabour, J., van den Hoff, J. & Bergstrom, D. M. Our footprint on Antarctica competes with nature for rare ice-free land. *Nature Sustainability* **2**, 185-190, doi:<https://doi.org/10.1038/s41893-019-0237-y> (2019).

40	Convey, P. & Peck, L. S. Antarctic environmental change and biological responses. *Science Advances* **5**, eaaz0888, doi:<https://doi.org/10.1126/sciadv.aaz0888> (2019).

41	Leihy, R. I. *et al.* Antarctica’s wilderness fails to capture continent’s biodiversity. *Nature* **583**, 567-571, doi:<https://doi.org/10.1038/s41586-020-2506-3> (2020).

42	Murphy, K. P. *Machine Learning : A Probabilistic Perspective*. 1098 (MIT Press, 2012).

43	Pedregosa, F. *et al.* Scikit-learn: Machine learning in Python. *Journal of machine learning research* **12**, 2825-2830, doi:<https://doi.org/10.5555/1953048.2078195> (2011).

44	Hoffman, M., Bach, F. & Blei, D. in *Advances in neural information processing systems.*

45	Hoffman, M. D., Blei, D. M., Wang, C. & Paisley, J. Stochastic variational inference. *J. Mach. Learn. Res.* **14**, 1303–1347 (2013).

46	Chang, J., Gerrish, S., Wang, C., Boyd-Graber, J. & Blei, D. in *Advances in neural information processing systems.* (NeurIPS Proceedings).

47	Blei, D. M. & Lafferty, J. D. in *Proceedings of the 23rd international conference on Machine learning*	113–120 (Association for Computing Machinery, Pittsburgh, Pennsylvania, USA, 2006).

48	Cheng, X., Yan, X., Lan, Y. & Guo, J. BTM: Topic Modeling over Short Texts. *IEEE Transactions on Knowledge and Data Engineering* **26**, 2928-2941, doi:10.1109/TKDE.2014.2313872 (2014).

49	Dieng, A. B., Ruiz, F. J. R. & Blei, D. M. Topic Modeling in Embedding Spaces. *Transactions of the Association for Computational Linguistics* **8**, 439-453, doi:10.1162/tacl_a_00325 (2020).

50	Churchill, R. & Singh, L. The Evolution of Topic Modeling. *ACM Comput. Surv.* **54**, Article 215, doi:10.1145/3507900 (2022).

51	Grootendorst, M. BERTopic: Neural topic modeling with a class-based TF-IDF procedure. doi:10.48550/arxiv.2203.05794 (2022).

52	Hughes, K. A., Gray, A. D. & Ager, B. J. Attainment of consultative status by parties to the Antarctic Treaty: past, present and future. *The Polar Journal* **14**, 560-591, doi:10.1080/2154896X.2024.2414642 (2024).

53	Röder, M., Both, A. & Hinneburg, A. 399-408 (Association for Computing Machinery).

54	Newman, D., Lau, J. H., Grieser, K. & Baldwin, T. in *Human language technologies: The 2010 annual conference of the North American chapter of the association for computational linguistics.* 100-108 (Association for Computational Linguistics).

55	Dudeney, J. R. & Walton, D. W. H. Leadership in politics and science within the Antarctic Treaty. *Polar Research* **31**, 11075, doi:<https://doi.org/10.3402/polar.v31i0.11075> (2012).

56	Haward, M. & Jackson, A. Antarctica: geopolitical challenges and institutional resilience. *The Polar Journal* **13**, 31-48, doi:<https://doi.org/10.1080/2154896X.2023.2205237> (2023).

57	Sampaio, D. P. Diplomatic culture and institutional design: Analyzing sixty years of Antarctic Treaty governance. *Anais da Academia Brasileira de Ciências* **94**, doi:<https://doi.org/10.1590/0001-3765202220210539> (2022).

58	Beck, P. J. Convention on the Regulation of Antarctic Mineral Resource Activities: a major addition to the Antarctic Treaty System. *Polar Record* **25**, 19-32, doi:<https://doi.org/10.1017/S0032247400009943> (1989).

59	Joyner, C. C. The Antarctic Minerals Negotiating Process. *The American journal of international law* **81**, 888-905, doi:<https://doi.org/10.2307/2203415> (1987).

60	Walton, D. W. H. in *Science Diplomacy: Antarctica, science, and the governance of international spaces*	(eds Paul Arthur Berkman, Michael A. Lang, David W. H. Walton, & Oran R. Young) 14 (Smithsonian Institution, 2011).

61	Andersen, R. T. in *The Antarctic Treaty System in world politics*	(eds Arnfinn Jørgensen-Dahl & Willy Østreng) Ch. 7, 16 (Fridtjof Nansen Institute, 1991).

62	Hemmings, A. D., Dodds, K. & Roberts, P. in *Handbook on the Politics of Antarctica*	(eds A.D. Hemmings, Klaus Dodds, & Peder Roberts) 21 (Edward Elgar Publishing Limited, 2017).

63	Brazil. An enhanced definition of bioprospection in Antarctica. Report No. XLI ATCM, WP 27, 2 (Buenos Aires, Argentina, 2018).

64	Antarctic Treaty Consultative Meeting (ATCM). Resolution 9 (2009): Collection and use of Antarctic biological material. Report No. XXXII ATCM (CEP XII), (Buenos Aires, Argentina, 2009).

65	Antarctic Treaty Consultative Meeting (ATCM). Resolution 7 (2005): Biological prospecting. Report No. XXVIII ATCM, (Buenos Aires, Argentina, 2005).

66	Herber, B. P. Bioprospecting in Antarctica: the search for a policy regime. *Polar Record* **42**, 139-146, doi:<https://doi.org/10.1017/S0032247406005158> (2006).

67	Argentina, Chile, France & Norway. in *Antarctic Treaty Consultative Meeting XLI*	(Secretariat of the Antarctic Treaty, Buenos Aires, Argentina, 2018).

68	Antonello, A. Nature conservation and Antarctic diplomacy, 1959–1964. *The Polar Journal* **4**, 335-353, doi:<https://doi.org/10.1080/2154896X.2014.954882> (2014).

69	Waller, D. C. Death of a Treaty: The Decline and Fall of the Antarctic Minerals Convention Notes. *Vanderbilt Journal of Transnational Law* **22**, 631-668 (1989).

70	Hughes, K. A. *et al.* Human-mediated dispersal of terrestrial species between Antarctic biogeographic regions: A preliminary risk assessment. *Journal of Environmental Management* **232**, 73-89, doi:<https://doi.org/10.1016/j.jenvman.2018.10.095> (2019).

71	Onley, I. R. *et al.* Assessing ongoing risks and managing detections of non-native invertebrates in the Antarctic Region. *NeoBiota* **95**, 133-147 (2024).

72	Australia. in *XXVII Antarctic Treaty Consultative Meeting*	(Cape Town, South Africa, 2004).

73	CEP. in *Antarctic Treaty Consultative Meeting*	(Secretariat of the Antarctic Treaty, Helsinki, Finland, 2023).

74	Leihy, R. I. *et al.* Antarctic Biosecurity Policy Effectively Manages the Rates of Alien Introductions. *Earth's Future* **13**, e2024EF005405, doi:<https://doi.org/10.1029/2024EF005405> (2025).

75	Argentina & United Kingdom. in *Antarctic Treaty Consultative Meeting X*	(Secretariat of the Antarctic Treaty, London, UK, 1977).

76	Zhang, M., Haward, M. & McGee, J. Marine plastic pollution in the polar south: Responses from Antarctic Treaty System. *Polar Record* **56**, e36, doi:10.1017/S0032247420000388 (2020).

77	Hemmings, A. D. in *Polar Geopolitics? Kowledges, resources and legal regimes*	(eds Richard C. Powell & Klaus Dodds) Ch. 4, 18 (Edward Elgar Publishing, 2014).

78	Brooks, C. M., Press, T., Bloom, E., Goldsworthy, L. & Chen, J. in *Antarctica and the earth system*	Ch. 10, 30 (Routledge, 2025).

79	ATCM. in *Antarctic Treaty Consultative Meeting.* (Secretariat of the Antarctic Treaty).

80	ATS. *Status of Antarctic Specially Protected Area and Antarctic Specially Managed Area Management Plans.*, <<https://www.ats.aq/e/protected.html>> (2024).

81	Brooks, C. M. *et al.* Science-based management in decline in the Southern Ocean. *Science* **354**, 185-187, doi:doi:10.1126/science.aah4119 (2016).

82	Jacquet, J., Blood-Patterson, E., Brooks, C. & Ainley, D. ‘Rational use’ in Antarctic waters. *Marine Policy* **63**, 28-34, doi:<https://doi.org/10.1016/j.marpol.2015.09.031> (2016).

83	Brooks, C. M., Crowder, L. B., Österblom, H. & Strong, A. L. Reaching consensus for conserving the global commons: The case of the Ross Sea, Antarctica. *Conservation Letters* **13**, e12676, doi:<https://doi.org/10.1111/conl.12676> (2020).

84	Nocito, E. S. & Brooks, C. M. The influence of Antarctic governance on marine protected areas in the Biodiversity Beyond National Jurisdiction Agreement negotiations. *npj Ocean Sustainability* **2**, 13, doi:10.1038/s44183-023-00019-5 (2023).

85	Isoaho, K., Gritsenko, D. & Mäkelä, E. Topic Modeling and Text Analysis for Qualitative Policy Research. *Policy Studies Journal*, doi:<https://doi.org/10.1111/psj.12343> (2019).

86	Denny, M. J. & Spirling, A. Text Preprocessing For Unsupervised Learning: Why It Matters, When It Misleads, And What To Do About It. *Political Analysis* **26**, 168-189, doi:<https://doi.org/10.1017/pan.2017.44> (2018).

87	Grimmer, J. & Stewart, B. M. Text as Data: The Promise and Pitfalls of Automatic Content Analysis Methods for Political Texts. *Political Analysis* **21**, 267-297, doi:10.1093/pan/mps028 (2013).

88	Dastidar, P. G. National and institutional productivity and collaboration in Antarctic science: an analysis of 25 years of journal publications (1980-2004). *Polar Research* **26**, 175-180, doi:10.1111/j.1751-8369.2007.00017.x (2007).

89	Dastidar, P. G. & Ramachandran, S. Intellectual structure of Antarctic science: A 25-years analysis. *Scientometrics* **77**, 389-414, doi:10.1007/s11192-007-1947-x (2008).

90	Ji, Q., Pang, X. & Zhao, X. A bibliometric analysis of research on Antarctica during 1993–2012. *Scientometrics* **101**, 1925-1939, doi:10.1007/s11192-014-1332-5 (2014).

91	Pustejovsky, J. & Stubbs, A. *Natural language annotation for machine learning*. 1st edition edn, (O'Reilly, 2013).

92	Gray, A. D. (Lapin yliopisto).

93	Gray, A. D. & Hughes, K. A. Demonstration of “substantial research activity” to acquire consultative status under the Antarctic Treaty. *Polar Research* **35**, 34061, doi:10.3402/polar.v35.34061 (2016).

94	Field, A. *Discovering statistics using R*. (SAGE, 2012).

95	Pullin, A. S. & Stewart, G. B. Guidelines for Systematic Review in Conservation and Environmental Management. *Conservation Biology* **20**, 1647-1656, doi:10.1111/j.1523-1739.2006.00485.x (2006).

96	Cohen, J. A Coefficient of Agreement for Nominal Scales. *Educational and psychological measurement* **20**, 37-46, doi:10.1177/001316446002000104 (1960).

97	Landis, J. R. & Koch, G. G. The Measurement of Observer Agreement for Categorical Data. *Biometrics* **33**, 159-174, doi:10.2307/2529310 (1977).

98	Brown, L. D., Cai, T. T. & DasGupta, A. Interval Estimation for a Binomial Proportion. *Statistical Science* **16**, 101-133, 133 (2001).

99	Bystrov, V., Naboka, V., Staszewska-Bystrova, A. & Winker, P. Cross-Corpora Comparisons of Topics and Topic Trends. *Jahrbücher für Nationalökonomie und Statistik* **242**, 433-469, doi:10.1515/jbnst-2022-0024 (2022).

100	Wilcoxon, F. Individual Comparisons by Ranking Methods. *Biometrics Bulletin* **1**, 80-83, doi:10.2307/3001968 (1945).

101	Sokolova, M. & Lapalme, G. A systematic analysis of performance measures for classification tasks. *Information Processing & Management* **45**, 427-437, doi:10.1016/j.ipm.2009.03.002 (2009).

102	Grandini, M., Bagli, E. & Visani, G. (arXiv, 2020).

103	Opitz, J. A Closer Look at Classification Evaluation Metrics and a Critical Reflection of Common Evaluation Practice. *Transactions of the Association for Computational Linguistics* **12**, 820-836, doi:10.1162/tacl_a_00675 (2024).

104	Schofield, A., Magnusson, M. & Mimno, D. in *EACL 2017.* (eds Mirella Lapata, Phil Blunsom, & Alexander Koller) 432-436 (Association for Computational Linguistics).

105	Bystrov, V., Naboka-Krell, V., Staszewska-Bystrova, A. & Winker, P. Analysing the Impact of Removing Infrequent Words on Topic Quality in LDA Models. *arXiv*, 21, doi:<https://arxiv.org/abs/2311.14505> (2023).
